# Tungsten-anisole complex provides 3,6-substituted cyclohexenes for highly diversified chemical libraries

**DOI:** 10.1126/sciadv.adl0885

**Published:** 2024-02-16

**Authors:** Justin T. Weatherford-Pratt, Jeremy M. Bloch, Jacob A. Smith, Megan N. Ericson, Daniel J. Siela, Mason R. Ortiz, Mary H. Shingler, Sarah Fong, Jonathan A. Laredo, Ishaan U. Patel, Matt McGraw, Diane A. Dickie, W. Dean Harman

**Affiliations:** Department of Chemistry, University of Virginia, Charlottesville, VA 22904 USA.

## Abstract

Medicinal chemists use vast combinatorial molecular libraries to develop leads for new pharmaceuticals. The syntheses of these compounds typically rely on coupling molecular fragments through atoms with planar (sp^2^) geometry. These so-called flat molecules often lack the protein binding site specificity needed to be an effective drug. Here, we demonstrate a coupling strategy in which a cyclohexene is used as a linker to connect two diverse molecular fragments while forming two new tetrahedral (sp^3^) stereocenters. These connections are made with the aid of a tungsten complex that activates anisole toward an unusual double protonation, followed by sequential nucleophilic additions. As a result, either cis- or trans-disubstituted cyclohexenes can be prepared with a range of chemical diversity unparalleled by other dearomatization methods.

## Teaser

Three sequential nucleophilic additions provide cis and trans cyclohexenes with structural complexity.

## INTRODUCTION

**Fig. 1.**
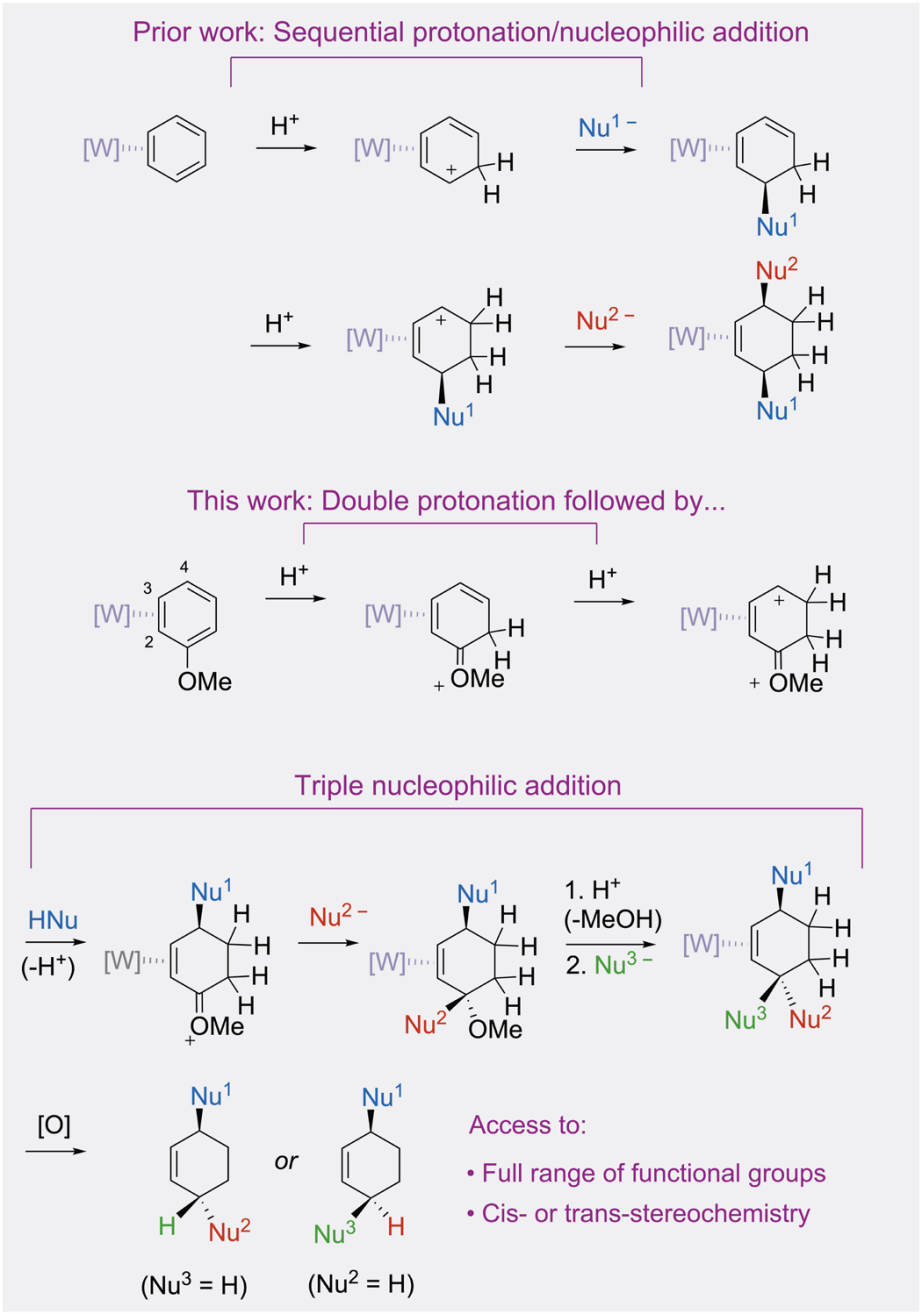
Overview of the synthetic design explored in this study. [W] = WTp(NO)(PMe_3_), where Tp = trispyrazolylborate. Nu = nucleophile. [O] = oxidant. The counterion is OTf^−^ (CF_3_SO_3_^−^). Either *cis*- or *trans*-3,6-cyclohexenes can be prepared by this method.

The development of a new drug typically requires the screening of millions of compounds. Because of this, medicinal chemists often prioritize quantity over structural complexity in producing molecular libraries for drug discovery (*1*, *2*). Such compounds are typically derived from high-throughput syntheses, which tend to use a small number of reliable chemical reactions. However, this narrow scope of synthetic methods has led to an overpopulation of certain types of molecular shapes and properties, to the exclusion of others (*3*). For example, “flat” compounds, rich in planar (sp^2^) carbons (*4*), often predominate these libraries, since they are easily generated from reliable aromatic ring-coupling strategies. Unfortunately, these structures have little in common with most naturally occurring, biologically active compounds, which tend to be rich in tetrahedral (sp^3^) carbon stereocenters. Because such molecules are better able to optimally fill regions of a targeted binding site, the fraction of sp^3^ carbons in a potential drug has been strongly correlated with its clinical success (*4*). Since the diversity of a chemical library is limited by the breadth of available chemical reactions, the challenge is to develop general synthetic approaches that lead to new compounds with both structural diversity and complexity. Libraries generated using such reactions could ultimately enhance the discovery of new medicines. Here, we describe an approach to cyclohexene-based compounds with adjustable relative and absolute stereochemistry and access to a wide range of functional groups. Central to this approach is an electron-rich tungsten reagent that can promote an unusual double protonation of anisole, thereby transforming the arene into a highly electrophilic intermediate, capable of undergoing up to three sequential addition reactions with nucleophiles (Fig. 1). While the “dearomatization” of benzene has been widely studied (*5**–**8*), the double protonation/triple nucleophilic addition sequence described herein allows for unparalleled access to structural variability. A portion of this study has been previously communicated (*9*).

## RESULTS

### Double protonation of anisole

**Fig. 2.**
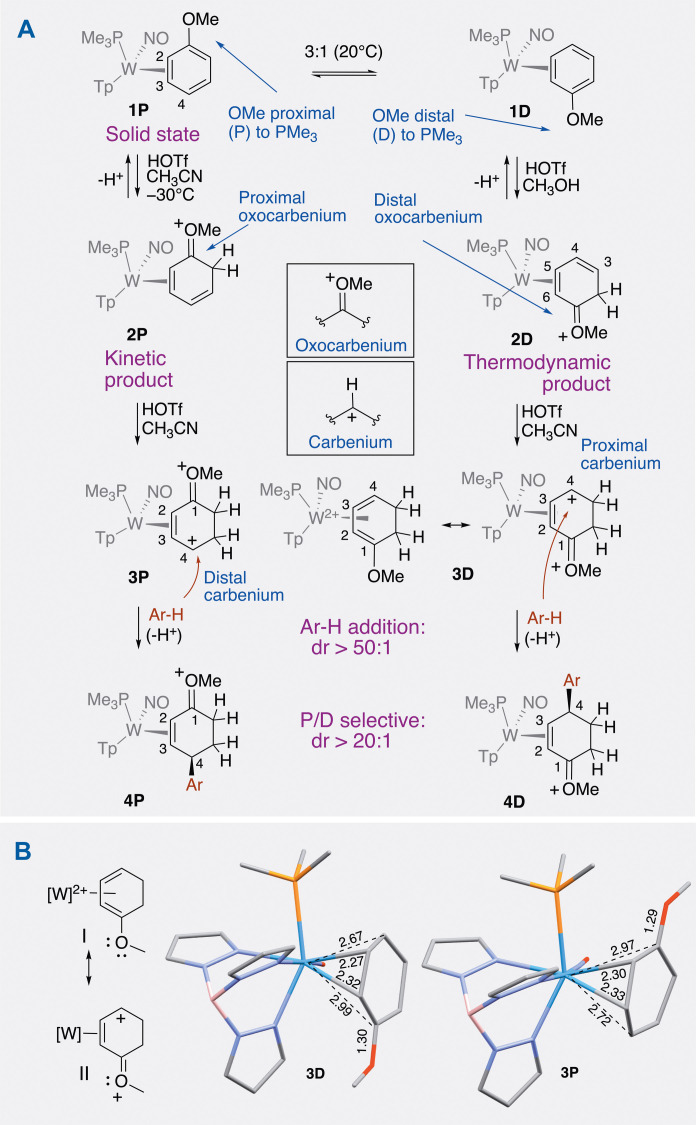
Formation of η^2^-enonium complexes from the double protonation of anisole. (**A**) This reaction sequence shows how an anisole complex is elaborated into an η^2^-enonium complex. Depending on which face of the anisole is coordinated, two different diastereomers can be prepared (**4P** or **4D**). (**B**) Resonance contributors for **3D** and the comparison of **3D** to **3P**. DFT calculations (SM) (*63*) demonstrate that the η^2^-enonium complex can be considered as a highly distorted W(II)-η^4^-methoxydiene complex in which the terminal carbons are only loosely coordinated. [W] = WTp(NO)(PMe_3_), where Tp = trispyrazolylborate.

The complex WTp(NO)(PMe_3_)(η^2^-anisole), (**1**, Fig. 2) prepared on a 45-g scale from W(CO)_6_ (*10*), (52% yield; optimized procedure reported in the Supplementary Materials) features the anisole ligand bound to tungsten at C2 and C3. Here, the metal-arene bond is stabilized in part by the interaction of a filled metal dπ orbital with a π* orbital of the aromatic ligand. This back-donation activates η^2^-bound arenes toward electrophilic addition or protonation (*11*, *12*). While this anisole complex has a half-life of roughly 45 min in solution saturated with air (*10*), it readily protonates at the uncoordinated ortho carbon with triflic acid/methanol (HOTf/MeOH) to form a 2H-anisolium complex (**2**) (*13*, *14*). Since {WTp(NO)(PMe_3_)} has a tungsten stereocenter, the corresponding η^2^-anisole complex exists as a mixture of coordination diastereomers **1D** and **1P** (*13*, *14*). While the equilibrium ratio of **1D**:**1P** is only 1:3, its conjugate acid, the 2H-anisolium analog **2D**, is thermodynamically favored by ~4 kcal/mol over its proximal isomer **2P**, and the diastereomeric ratio (dr) is >20:1 (**2D**:**2P**) under equilibrating conditions (*14*). Although the 2H-anisolium species formally features an oxocarbenium ion, the extensive π-backbonding from the tungsten stabilizes this moiety to the point that the complex may be precipitated from solution and stored. The C3-C6 fragment of **2D** now resembles an η^2^-1,3-diene complex and, as such, is moderately basic at C3 (*15*, *16*). Correspondingly, when a pure sample of **2D** is subjected to highly acidic conditions (HOTf/CH_3_CN), a second protonation occurs exclusively at the diene terminus C3 to form the dication **3D** (Fig. 2).

Attempts to induce precipitation of **3D** from a CH_2_Cl_2_ solution by addition of pentane were unsuccessful, and treating a CH_3_CN solution of **3D** with Et_2_O led to the regeneration of **2D**. However, observation and characterization of **3D** were possible by low temperature protonation of **2D** (0.030 g, 0.039 mmol; CD_3_CN) with an excess of triflic acid-*d_1_* (~5 drops) at −30°C. The diastereotopic hydrogen atoms of two different methylene groups exhibit signals between 2.7 and 3.8 parts per million (ppm), and the ^31^P-^183^W coupling constant drops to 240 Hz, a value notably lower than its precursors **2D** (*J*_WP_ = 280 Hz) or **1D** (*J*_WP_ = 310 Hz). This feature suggests an expanded coordination number for the tungsten in **3D** (*10*). Density functional theory (DFT) calculations (Fig. 2) indicate that the structure of **3D** resembles a highly distorted W(II)-η^4^-methoxydiene complex in which C1 (3.04 Å) and C4 (2.67 Å) are only weakly coordinated to the metal. By way of comparison, a single-crystal x-ray diffraction (SC-XRD) study of the analogous double-protonated dimethylaniline complex shows W-C1 = 3.13 Å and W-C4 = 2.63 Å (*15*). In contrast to the large difference in energy calculated for the 2H-anisolium diastereomers **2D** and **2P** (4.3 kcal/mol), **3P** is only 2.7 kcal/mol more stable than **3D** in CH_2_Cl_2_, but the latter appears to be kinetically stable, even at room temperature.

### Addition of the first nucleophile (Nu^1^ = Ar-H)

**Fig. 3.**
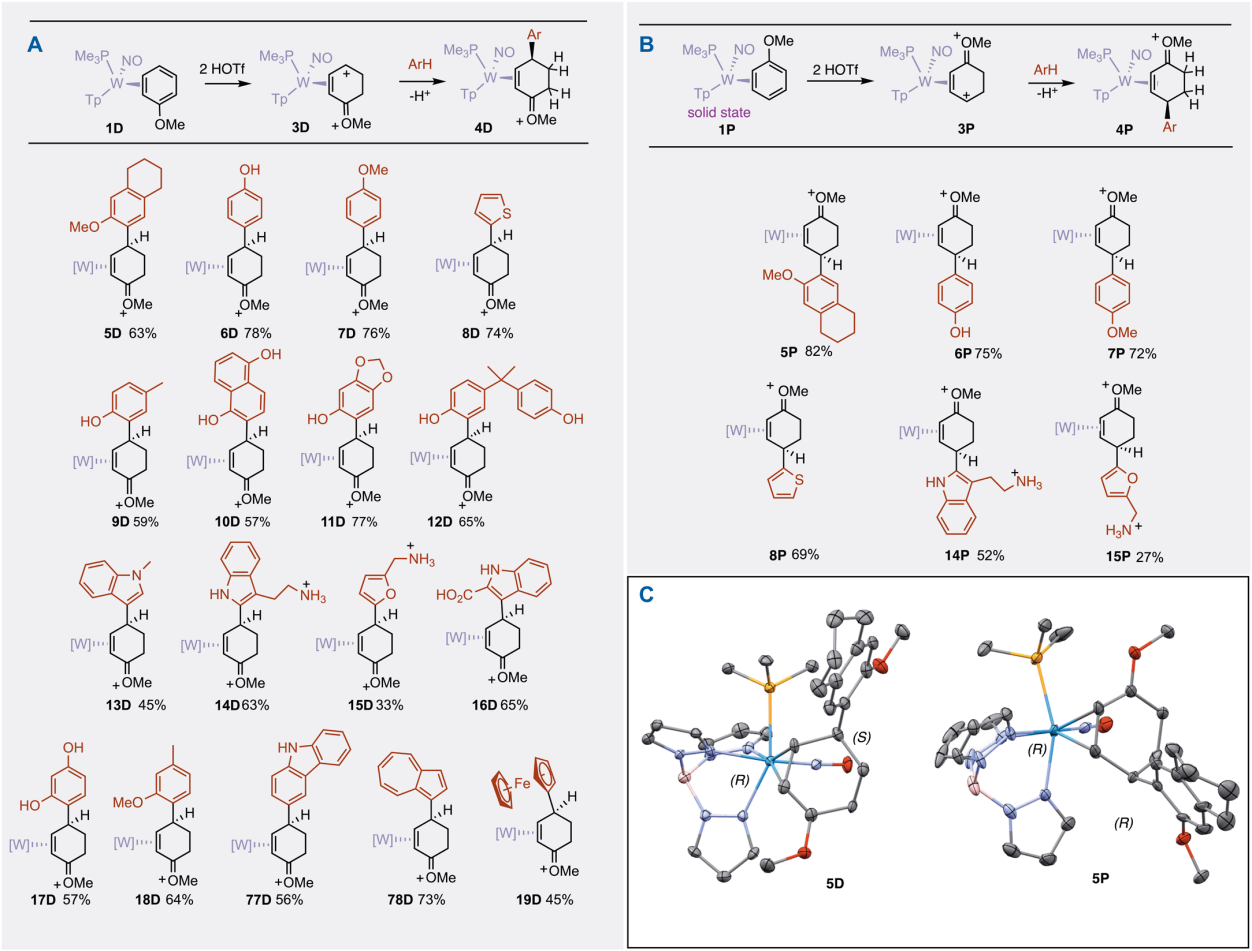
Examples of η^2^-enonium complexes. (**A**) Distal-oriented complexes: These complexes are prepared by a double protonation of the distal-oriented anisole complex (**1D**), which enables the ring coupling to Ar-H. [W] = WTp(NO)(PMe_3_), where Tp = trispyrazolylborate. (**B**) Proximal-oriented η^2^-enonium complexes: These complexes are prepared by a double protonation of the proximal-oriented anisole complex (**1P**), which enables the ring coupling of Ar-H. (**C**) A comparison of the molecular structures of **5D** and **5P**. These structures represent the distal and proximal diastereomers of the 6-methoxytetralin addition. Note how the new stereocenter can be controlled without altering the configuration of the tungsten complex. Only the R configuration of the tungsten is shown for the racemic mixture.

C4 of the η^4^-methoxydiene complex **3D** is highly electrophilic (Fig. 2) and, as such, was found to react with neutral, electron-rich π nucleophiles. Here, we focus our attention primarily on the reactions of **3D** with aromatic molecules. This compound was found to participate in electrophilic aromatic substitution (EAS) reactions to generate enonium complexes of the form **4D** (Fig. 2). In terms of Mayr’s nucleophilicity parameters (*17*), nucleophiles with an *N* parameter (*N*) ≥ ~ −1 are predicted to successfully add (e.g., anisole, *N* = −1.2; thiophene, *N* = −1.0). Aromatic molecules that successfully add to **3D** (Fig. 3) include phenols (**6D**, **9D**, and **12D**), anisoles (*N* ~ −1; **5D** and **7D**), 1,5-hydroxynaphthalene (**10D**), indoles (*N* ~ 7; **13D**, **14D**, and **16D**), furans (*N* ~ 1.3; **15D**), and carbazole (**77D**). Azulene (*18*) (*N* ~ 7; **78D**) and ferrocene (*N* ~ 2.5; **19D**) also cleanly undergo an EAS reaction with **3D**. In the case of thiophene and furan (**8D** and **15D**), the reaction predictably takes place at the α carbon (regioselectivity > 10:1). For unsubstituted phenol and anisole (**6D** and **7D**), the reaction selectively occurs at the para position (regioisomer ratio > 10:1). Care must be taken to keep the aromatic in excess to avoid the formation of a binuclear tungsten complex (the coupling of two tungsten-anisole complexes to one aromatic; see the Supplementary Materials). Benzene (*N* = −6.3), toluene (*N* = −4.5), and electron-deficient arenes fail to react with the η^4^-methoxydiene complex **3D**, as do anilines, which immediately protonate at the nitrogen under the acidic reaction conditions, thereby deactivating the aromatic nucleophile. In contrast, while remote amine groups such as those found in the preparation of **14D** or **15D** also protonate at nitrogen, the resulting ammonium groups are far enough from the aromatic ring as to avoid deactivation. In contrast to many EAS reaction types (e.g., Friedel-Crafts alkylation), remote amines, hydroxy groups, and carboxylic acids are tolerated without suppression of the reaction as no external Lewis acids are involved. Attempts to carry out the EAS reaction using substoichiometric amounts of acid (fewer than 2.0 equivalents of acid per anisole complex **1**) were unsuccessful.

**Fig. 4.**
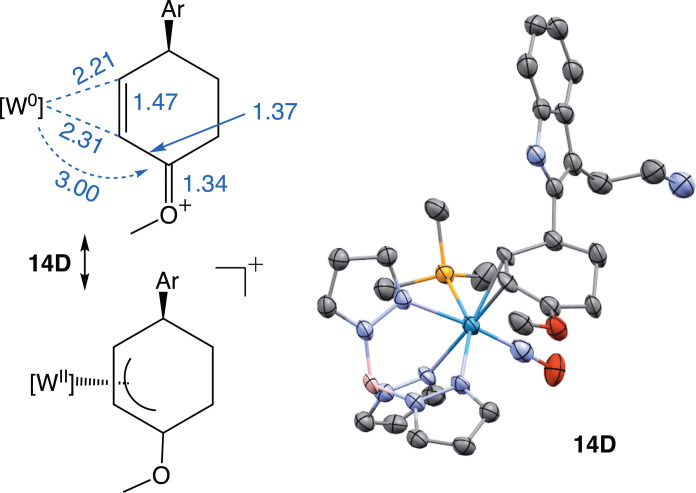
ORTEP diagram of tryptamine η^2^-enonium complex 14D. Determined from a single-crystal x-ray diffraction (SC-XRD) analysis (50% ellipsoids); distal orientation; Ar-H = tryptamine. Key bond lengths for **14D** are provided in angstroms (Å). [W] = WTp(NO)(PMe_3_), where Tp = trispyrazolylborate.

Crystals were obtained for several examples, including the addition of 6-methoxytetralin (**5D**) and tryptamine (**14D**). An Oak Ridge thermal ellipsoid plot (ORTEP) diagram of **14D** is shown in Fig. 4. A key spectral feature of the entire set of compounds (**5D** to **19D**; **77D** and **78D**) is that the methoxy signal for the oxocarbenium moiety is around 3 ppm, notably shifted upfield from its expected value of ~4.5 ppm (found for proximal isomers). This is attributed to a favored conformational isomer in which the methyl group is located in the pocket between two pyrazole rings (Fig. 4). This feature was earlier described for a series of rhenium complexes of the form ReTp(CO)(MeIm)(L), where L was an alkene (*19*). Compounds **5D** and **14D** appear to be rare cases of structurally characterized η^2^-enonium complexes. We draw a comparison to a compound described by the Liebeskind group as a “methoxy-substituted η^3^-cyclohexenyl complex” of MoTp(CO)_2_, which has an unusually long bond (~2.66 Å) between the metal and the methoxy-substituted allyl carbon (analogous to **14D** in Fig. 4) (*20*, *21*). By contrast, the analogous bond lengths (W to the oxocarbenium carbon) in **5D** and **14D** are both 3.00 Å, indicating that the latter structures are best characterized as η^2^-enonium complexes, with stabilization of the oxocarbenium group coming primarily from a π-interaction with its neighboring carbon (1.37 Å).

An SC-XRD analysis reveals that the solid (crystalline) state of anisole complex **1** contains only the proximal isomer (*22*). The conversion of η^2^-anisole complex to η^2^-enonium complex also can be carried out starting with the proximal coordination diastereomer of the anisole complex (**1P**) (the coordination diastereomer that is initially present in the crystalline state; Fig. 2) (*22*). Dissolving this material in a cold acidic solution generates the proximal form of the 2H-anisolium complex exclusively (**2P**), which can be protonated a second time (**3P**) and then combined with various aromatic nucleophiles to generate the proximal isomer of η^2^-enonium complex (type **4P**, Fig. 2). In Fig. 3, examples are provided that show several aromatic nucleophiles adding to the proximal double-protonated anisole complex (**3P**). We note here that **3P** appears to be notably slower to react than the distal form (**3D**). This observation is consistent with the fact that the carbenium carbon is more stable distal to the PMe_3_ than proximal to it (*15*). Note that **3P** derives its “P” designation from the methoxy group of the anisole it was derived from (i.e., **1P**); however, in **3P** the carbenium carbon is distal to the PMe_3_; see Fig. 2. In Fig. 3, two crystal structures are provided that compare the proximal and distal diastereomers of the 6-methoxytetralin addition. They illustrate that either hand of the new stereocenter created can be selectively obtained without changing the hand of the metal but rather solely by the manipulation of the η^2^-anisole substrate.

**Fig. 5.**
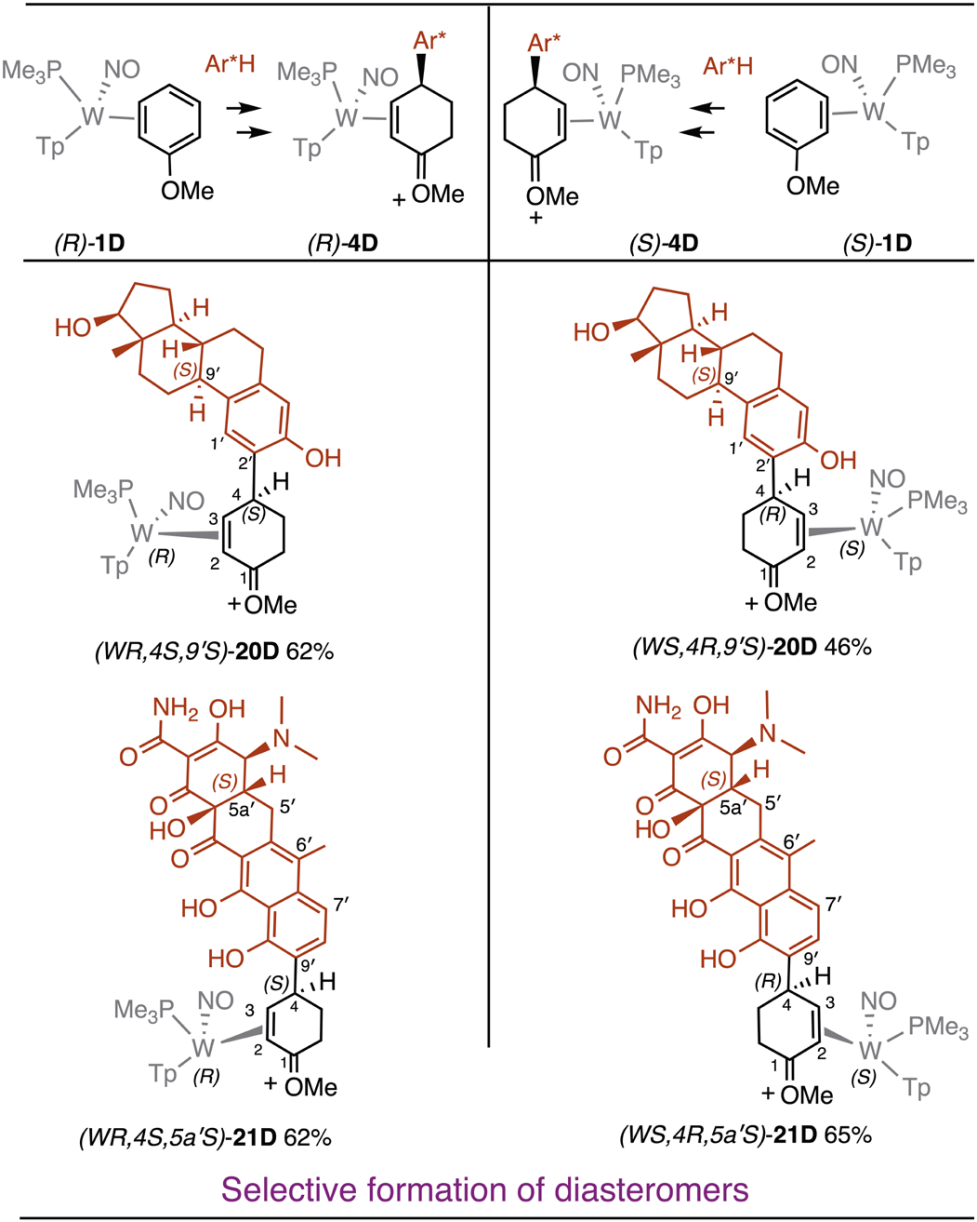
Selective formation of η^2^-enonium complex diastereomers. When the aromatic fragment (Ar^*^-H) is chiral, complimentary configurations of the tungsten stereocenter in WTp(NO)(PMe_3_)(η^2^-anisole) result in two different diastereomers of the coupled product.

When the aromatic ligand is chiral, two diastereomers are expected when using a racemic mixture of the tungsten complex **1**. Compound **1** can be prepared in enantioenriched form at multigram scale (optimized procedure reported in the Supplementary Materials) (*10*, *23*), and each isolated hand leads to a different diastereomer when treated with a chiral aromatic nucleophile (Ar^*^-H). Examples are provided for two systems of biological relevance in Fig. 5. Both β-estradiol and tetracycline contain fused phenolic rings, and both undergo reactions with **3P** or with **3D** ortho to the phenolic hydroxy group (C2′ for estradiol and C9’ for tetracycline). In the case of the tetracycline, the strongly acidic reaction conditions trigger loss of water to form the anhydrotetracycline derivative (*WR,4S,5a′S*)-**21D** or (*WS,4R,5a′S*)-**21D**. This hypothesis was confirmed by repeating the experiment with authentic anhydrotetracycline. High resolution mass spectrometry (HRMS) data for **21D** also support loss of water when tetracycline is used as a nucleophile (Ar^*^-H).

We next endeavored to carry out a similar reaction sequence to that shown in Fig. 2 starting from a methylated anisole. Unfortunately, our preliminary experiments were unsuccessful. While we were able to make complexes of both 4-methylanisole and 3-methylanisole, as well as protonate them, attempts to double-protonate (HOTf) these species and add a test nucleophile (dimethoxybenzene) did not generate any recognizable products. Attempts to bind 2-methylanisole failed altogether.

### Addition of the second nucleophile (Nu^2^ = H^−^)

**Fig. 6.**
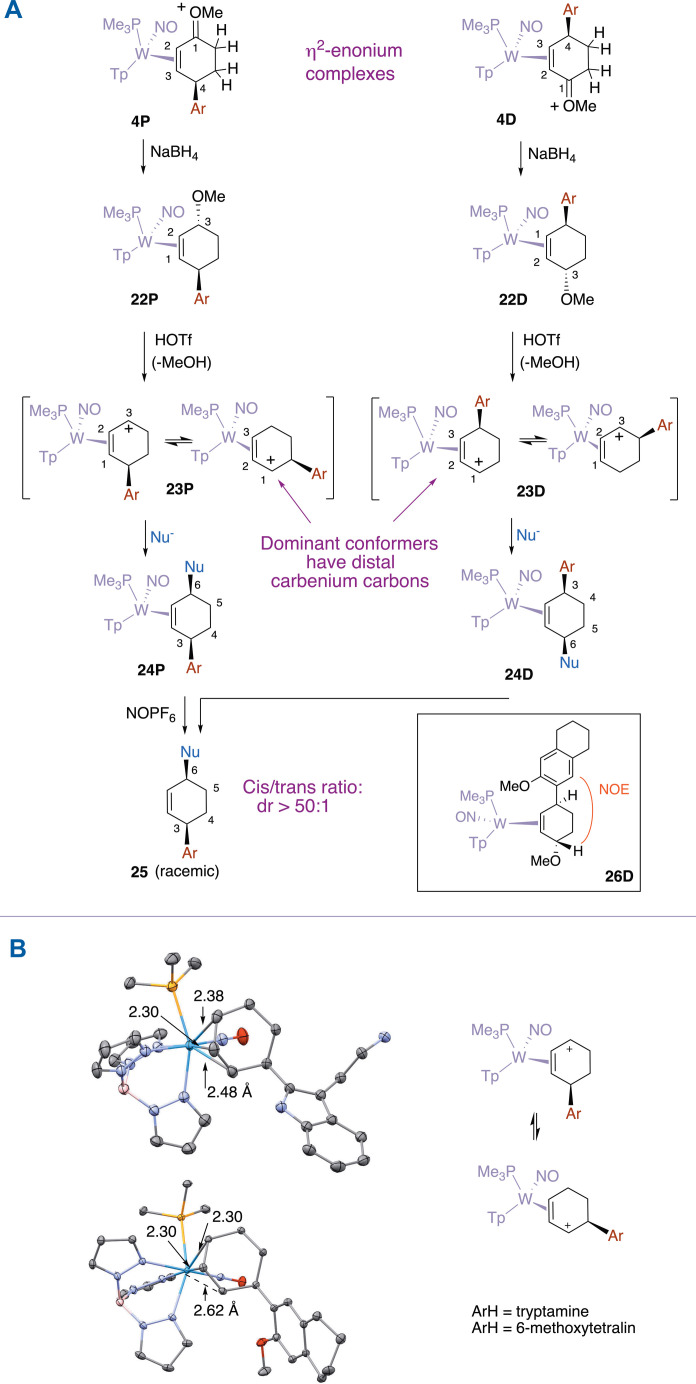
Preparation of cis-3,6-disubstituted cyclohexene complexes. (**A**) The conversion of the enonium species (**4P** and **4D**) to a π-allyl complex (**23P** and **23D**) and the subsequent addition of the third nucleophile provides two different diastereomers of the cyclohexene product that differ by the cyclohexene stereochemistry relative to the tungsten stereocenter. (**B**) Comparison of molecular structures for two proximal η^2^-allyl complexes **30P** and **31P** showing the long bond between the tungsten and the carbenium-like carbon (ORTEP diagrams; 50% ellipsoids).

We next endeavored to reduce the η^2^-enonium compounds (**4P** and **4D**; Fig. 6) with NaBH_4_ to allyl ethers (**22P** and **22D**), which in the presence of acid were anticipated to convert to aryl-substituted “η^2^-allyl” complexes of the form **23P** and **23D** (Fig. 6) (*15*). These two synthetic steps were combined into a single procedure in most cases, but with **26D** (vide infra), the allyl ether intermediate was isolated and fully characterized. For this compound, nuclear Overhauser effect spectroscopy (NOESY), correlation spectroscopy (COSY), heteronuclear single-quantum coherence (HSQC), and heteronuclear multiple-bond correlation (HMBC) data indicate that the hydride is delivered to the oxocarbenium carbon of type **4D** anti to metal coordination. This stereochemistry is consistent with earlier reports (*14*) [but regrettably is misrepresented in (*9*) due to a graphical error]. In particular, an NOE interaction is observed for **26D** between the distal allyl methine proton and a tetralin proton (see Fig. 6). More generally, the allyl ether complexes (type **22P** and **22D**; Fig. 6) are converted directly into their corresponding η^2^-allyl complexes (type **23P** or **23D**) (*15*).

**Fig. 7.**
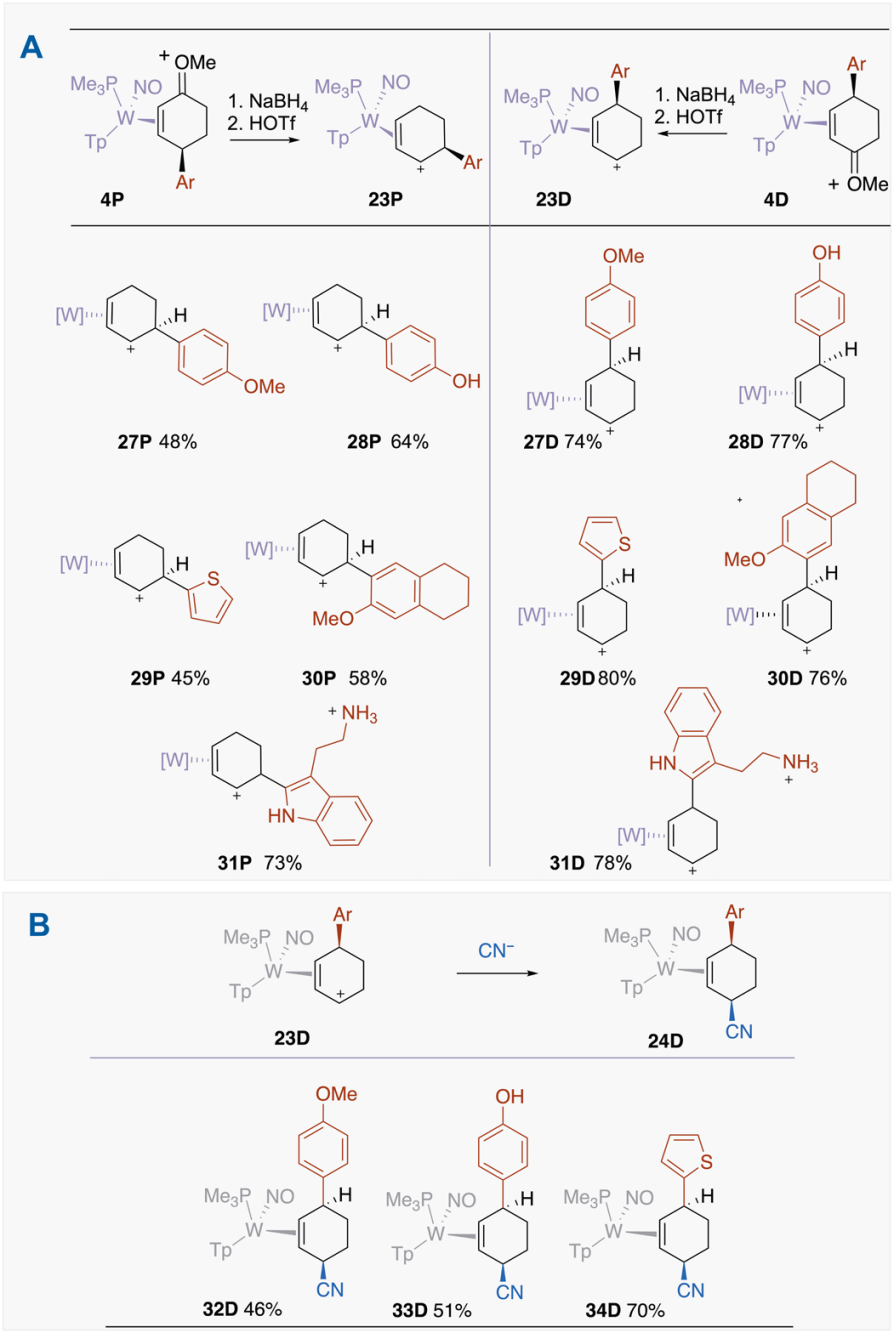
η^2^-allyl complexes and their conversion to cyclohexene complexes. (**A**) These P series and D series allyl complexes are direct precursors to proximal and distal *cis*-3,6-disubstituted cyclohexene complexes. [W] = WTp(NO)(PMe_3_), where Tp = trispyrazolylborate. (**B**) Formation of *cis*-3,6-disubstituted cyclohexene complex diastereomers prepared from the D series of allyl complexes.

Allyl complexes (Fig. 7) are represented herein as dihapto-coordinated (*15*), meaning that two of the three allyl carbons (~2.3 Å) are more tightly coordinated than the third. The “long-bond” can range anywhere from 2.5 to 3.0 Å (*15*). Furthermore, DFT calculations have demonstrated that these species exist as two distinct conformers (*15*), differing by which terminal carbon is elongated. Here, this is represented as a plus charge proximal or distal to the PMe_3_. We note that while the most common use of the term conformer refers to two species differing by rotation of a bond (i.e., rotational conformers), conformers can involve inversion of a bond angle (akamptisomers), trigonal pyramidal inversion, or other rearrangements (*24*). Allyl conformers of type P and D interconvert without bond breakage or rotation but are distinct structures, separated by a modest activation barrier (*15*). This type of distorted η^2^-allyl structure has been described previously in the literature but appears to be unusually pronounced in tungsten and molybdenum complexes containing a nitrosyl ligand (*13*, *25*). As described above, the {WTp(NO)(PMe_3_)} system shows a consistent preference to orient the carbenium carbon distal to the PMe_3_ (*15*). Consequentially, while the “D” series keeps the aryl-substituted cyclohexenyl ring oriented the same as the allyl ether precursor, the proximal series of allyl complexes undergoes an allyl shift, such that the carbenium carbon is adjacent to the aryl group (Figs. 6 and 7). Crystal structures of two allyl complexes from the P series (**30P** and **31P**) shown in Fig. 6 highlight this shift in geometry. This suggested that it might be possible to achieve different regioselectivities for the D and P allyl complexes, but to date, we have not been able to demonstrate this (vide infra). Although the dominant conformer for the proximal η^2^-allyl complexes places the carbenium carbon distal to the PMe_3_, assumed steric interactions with the aryl group inhibit reaction of nucleophiles with this species, and as a consequence, nucleophilic addition to the minor conformation is primarily observed. Thus, both the proximal and distal series of η^2^-allyl complexes deliver *cis*-3,6-disubstituted cyclohexene complexes, differing only in their orientation relative to the {WTp(NO)(PMe_3_)} fragment. The one exception we encountered was the addition of cyanide to **29P**, where the ratio of 3,6- to 3,4 addition is only 2:1 (*9*). In Fig. 7, these results are summarized for anisole-, phenol-, and thiophene-derived cyclohexylnitriles.

### Addition of the third nucleophile (Nu^3^ = C, N, O, S)

**Fig. 8.**
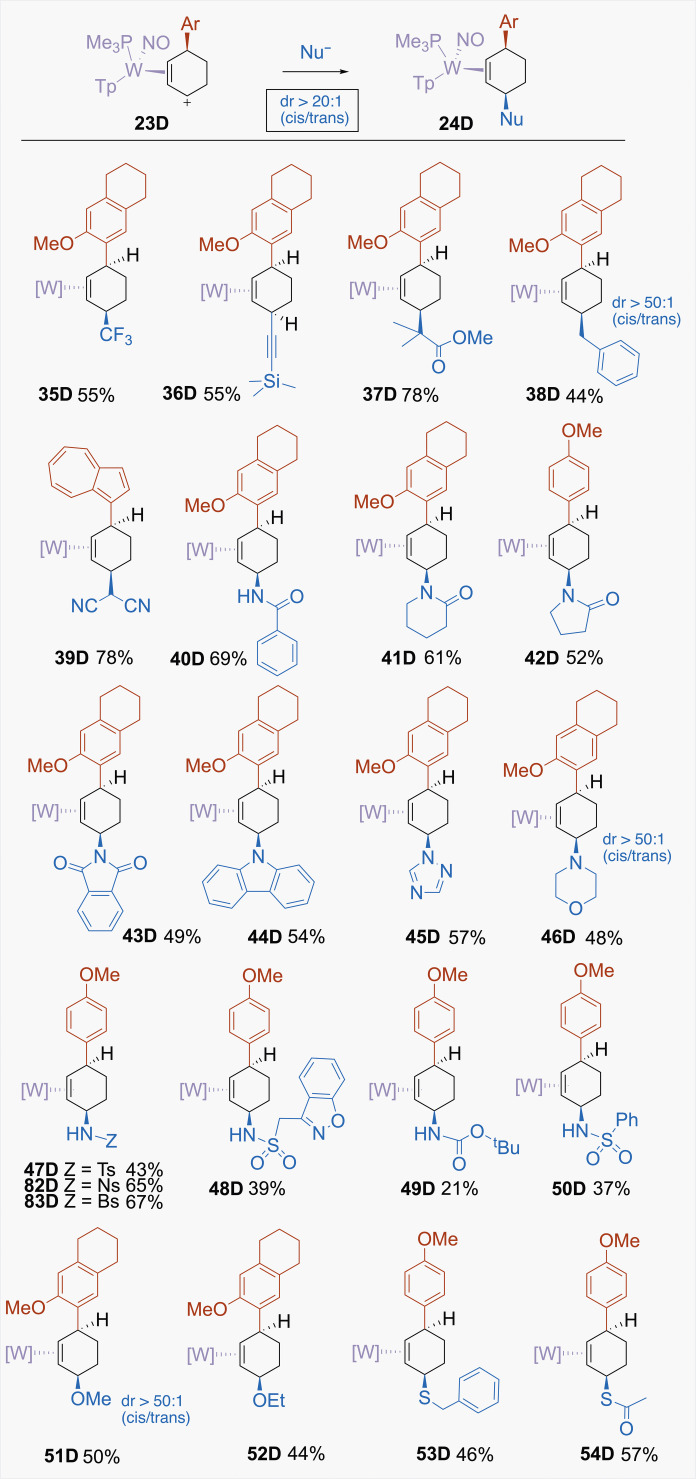
Selected examples of cis-3,6-disubstituted cyclohexene complexes. Examples include C-C, C-N, C-O, and C-S linkages. [W] = WTp(NO)(PMe_3_), where Tp = trispyrazolylborate. Ts = tosyl; Bs = brosyl; Ns = nosyl.

Using examples of η^2^-allyl complexes from the D series, the scope of the third nucleophile was explored in greater depth (Fig. 8). Nucleophiles that successfully add include all the major classes of carbon nucleophiles that have been used with the Cr(CO)_3_(η^6^-arene) system (*6*, *26*), such as the Ruppert-Prakash reagent (i.e., CF_3_TMS), ester enolates {e.g., [(1-methoxy-2-methylprop-1-en-1-yl)oxy]trimethylsilane}, and Grignard reagents. From this work and prior studies (*16*), π nucleophiles as weak as silyl enol ethers (*N* ~ 9)(*17*) or indoles (*N* ~ 6) (*17*) can add to cyclic allyl complexes such as [WTp(NO)(PMe_3_)(C_6_H_9_)]^+^. However, successful addition reactions were also observed for a broad array of nitrogen nucleophiles, which include amines {either “free” [**46D**] or protected [e.g., boc (**49D**), Bz (**40D**), and Ts (**47D**)]}, amides (**40D** to **42D**), phthalimide (**43D**), a triazole (**45D**), carbazole (**44D**), and sulfonamides (**47D**, **48D**, **50D**, **82D**, and **83D**), including the anticonvulsant zonisamide (Zonegran). While nucleophilic substitutions with amines are well established (*22*), the first report of dearomatizing benzene with nitrogen nucleophiles using {Cr(CO)_3_} or other η^6^-binding transition metal complexes has appeared only recently (*27*).

In all the cases we explored, the addition occurs anti to the tungsten, resulting in *cis*-3,6-cyclohexene complexes exclusively (dr > 20:1). An example of these structures is shown in Fig. 8 for the addition of 6-methoxytetralin and the Ruppert-Prakash reagent (**35D**). We note that, in most of these cases, the final nucleophile is added in anionic (i.e., deprotonated) form despite their basic nature, and addition usually occurs with minimal competition from deprotonation of the η^2^-cyclohexenylium species. In addition to the wide range of nitrogen and carbon nucleophiles, oxygen and sulfur nucleophiles could be added successfully, including alkoxides (**51D** and **52D**), thiols (**53D**), and even thioacetate (**54D**).

### Formation of compounds with trans-stereochemistry

**Fig. 9.**
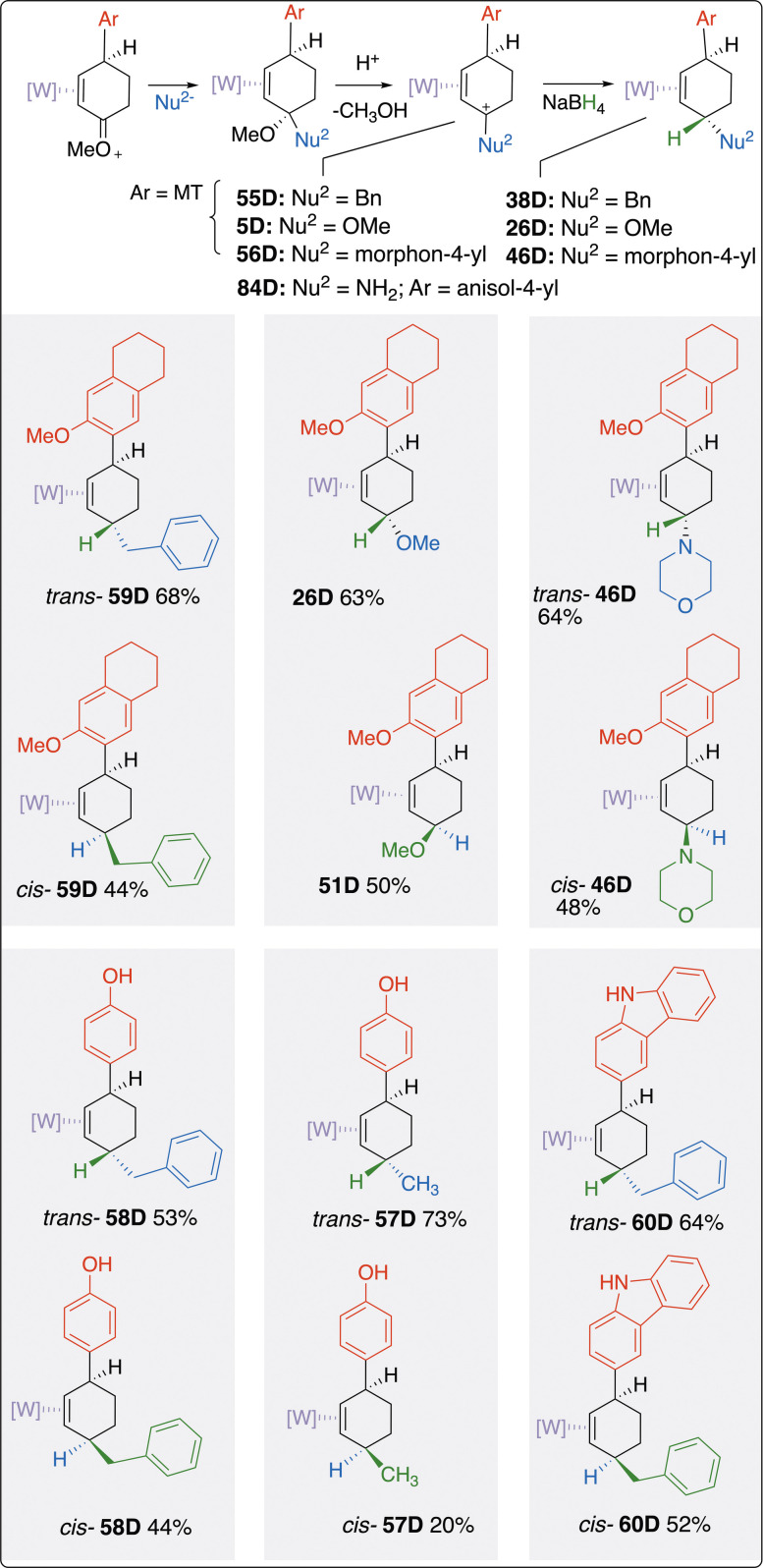
Selected examples of trans-3,6-disubstituted cyclohexene complexes. These complexes are shown with their *cis*-3,6-disubstituted counterparts for comparison. [W] = WTp(NO)(PMe_3_), where Tp = trispyrazolylborate. MT = 3-methoxy-5,6,7,8-tetrahydronaphthalen-2-yl. For the cis isomers, the cis/trans ratio: dr > 50:1. For the trans isomers, the trans/cis ratio is as follows: *trans*-**57D**: dr >20:1; *trans*-**58D**: dr >20:1; *trans*-**59D**: dr >10:1; *trans*-**60D**: dr >20:1; *trans*-**26D**: dr >50:1; *trans*-**46D**: dr >50:1. NBu_4_BH_4_ was used as a replacement for NaBH_4_ when THF was used as a solvent (SM) for better solubility.

As mentioned above, for virtually all η^2^-arene complexes, nucleophiles initially add anti to the face of metal coordination. This stereochemistry is reliable, but it represents a potential limitation to this tungsten-based dearomatization methodology in that the organic cyclohexenes produced in Fig. 9 are all cis-geometries. An attractive feature of the anisole chemistry described is the versatility provided by the oxocarbenium group. Not only can it be demethylated to generate cyclohexenones (*14*), or reduced to form 3-methoxycyclohexenes (vide infra), carbon or nitrogen nucleophiles can be added to the oxocarbenium carbon. The resulting allylic methoxy group can then be cleaved with acid (−MeOH) and then treated with a hydride, thereby forcing the “second nucleophile” in toward the metal (see Fig. 9). The result is a 3,6-disubstituted cyclohexene ligand with trans-stereochemistry. Several examples of this strategy are demonstrated in Fig. 9. In the first example, a benzyl Grignard is first added to the η^2^-enonium complex **5D**, followed by elimination of MeOH to form **55D**. [The analogous reaction was also run with Ar-H = phenol to form **85D**, whose structure was confirmed by an SC-XRD study (supporting materials.)] Subsequent hydride addition forms the *trans*-3,6-disubstituted substituted product *trans*-**59D**.Conversely, hydride addition, elimination of MeOH, and benzyl addition result in the cis stereochemistry, as was shown earlier (*cis*-**38D**). For amines, addition to the oxocarbenium results in an η^2^-eniminium system such as in **56D**, which upon hydride addition produces the *trans*-3,6-disubstituted cyclohexene *trans*-**46D**. SC-XRD studies confirm the structure of the η^2^-eniminium **56D** as well as the parent complex produced from ammonia (**84D**; Supplementary Materials). Last, as seen earlier, addition of hydride to the η^2^-enonium complex **5D** results in the *trans*-disubstituted allyl ether **26D**, while addition of methoxide at the η^2^-allyl stage delivers the cis-isomer **51D**. Additional examples of complementary cis- and trans-isomers are provided in Fig. 9.

### Release of the product cyclohexene

**Fig. 10.**
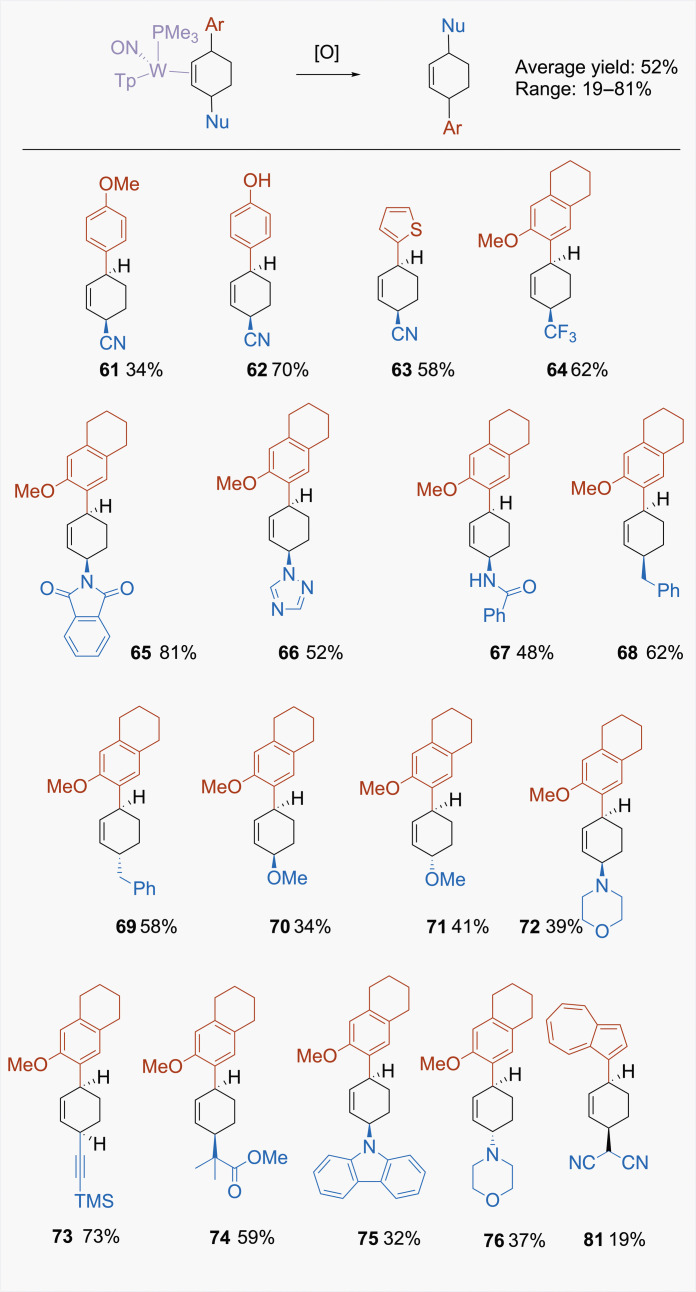
Selected examples of organic 3,6-disubstituted cyclohexenes. Prepared in racemic form. [O] = NOPF_6_, or [FeCp_2_]PF_6_, where Cp = cyclopentadienyl.

Treatment of complexes of the form WTp(NO)(PMe_3_)(cyclohexene) with moderate oxidants (E° > 0.5 V, NHE) such as NOPF_6_ or [FeCp_2_]PF_6_ [cyclopentadienyl (Cp)] results in decomplexation of the cyclohexene ligand (average yield: 52%). Limiting the equivalents of the oxidant used typically avoids any damage to the organic cyclohexene. In Fig. 10, results are summarized for several representative examples (**61** to **76**). Starting with a racemic mixture of the anisole complex (**1**), the organic compound isolated is identical, regardless of which pathway (P or D) it is derived from, and yields appear to be comparable. However, if the initial anisole complex is enantioenriched, then the P series and D series of reactions would yield opposite enantiomers (*9*). Unfortunately, in contrast to what is observed for racemic anisole complex (**1**) (vide supra), powders of enantioenriched **1** were often found to contain a mixture of **1P** and **1D** diastereomers. Hence, the best method for obtaining highly enantioenriched cyclohexenes is to use the **D** series and vary the hand of the metal, as we have previously demonstrated (*16*, *28*).

## DISCUSSION

This report describes a general procedure for both cis- and *trans*-3,6-disubstituted cyclohexenes. This method is compatible with an exceptionally broad range of suitable coupling partners. The disubstituted cyclohexenes shown in Fig. 10 and cyclohexanes similar to them have been shown to be valuable intermediates in the synthesis of natural products (*29*, *30*). Regarding similar approaches to functionalized cycloalkenes, Sarlah and his (*5*) group have recently developed a method to dearomatize benzenes via photocycloaddition with triazolinediones that can deliver *cis*-3,6-diaminocyclohexenes, cis-dihydroxylated cyclohexadienes, or *trans*-5,6-carboamiated cyclohexadienes. Procedures reported for synthesizing aryl-substituted cyclohexenes involve catalyzed allylic substitution (e.g., Tsuji-Trost) (*31*), oxidative allylic functionalization of cyclohexenes (*32*), and cross-coupling with cyclohexadienes (*33*, *34*). 3-Aryl cyclohexenes can also be prepared from coupling functionalized aryl groups (e.g., Grignards) with preexisting cyclohexadienes (*33*, *35*) or allylic ethers (*30*, *36*), but stereo- or regiocontrol can be an issue with any of these procedures. Other approaches to aryl-substituted cyclohexenes might involve construction of the carbocyclic ring. These include various carbocyclization reactions (*37*), intramolecular aldol reactions (*38*), ring-closing metathesis (*39*), and Diels-Alder reactions (*40*, *41*), but these methods lack the modular nature and broad scope demonstrated for the method described herein.

Alternatively, approaches typically used to couple aromatics to cycloalkanes involve cross-coupling reactions such as Negishi (*42*), Stille (*43*), Suzuki (*44*), and Hiyama (*45*), but such procedures are more challenging than sp^2^-sp^2^ coupling protocols and are often frustrated by elimination byproducts. Further, they usually require the use of precious metal catalysts and aryl halides or other suitable aryl precursors. Corey-House (*46*, *47*) and Kochi-Schlosser type couplings avoid precious metals (*48*). For cases where arenes are coupled to provide an sp^3^-sp^2^ linkage, strong Lewis-acid activators are typically required (Friedel-Crafts), and oligomerization is common (*49*). Electron-deficient transition-metal complexes and Brønsted acids have been used successfully in Friedel-Crafts alkylations in the case of benzyl electrophiles (*49*), where rearrangements of the carbocation intermediate are less of an issue. However, examples carried out with high stereoselectivity are rare owing to the fact that the active form of the electrophile is planar (*49*).

Unexpectedly, we were unable to find any strategies for the preparation of 6-substituted 3-arylcyclohexenes from η^6^-coordinated arene systems, as addition of carbon nucleophiles to Cr(CO)_3_(η^6^-benzene) leads to 1,3-cyclohexadienes or substituted arenes. Regarding other organometallic approaches germane to this study, the manganese system [(η^6^-arene)Mn(CO)_3_]^+^can undergo sequential nucleophilic additions to form a *cis*-5,6,-disubstituted 1,3-cyclohexadiene complex (*50*). This double nucleophilic addition approach requires highly reactive nucleophiles (e.g., butyllithium), which are not compatible with many other functional groups, and has not been widely applied to organic synthesis (*51*–*55*). Friedel-Crafts–type addition reactions have been observed with other highly electrophilic arene complexes (*56*), as well as dienyl complexes such as the [Fe(CO)_2_(η^5^-C_6_H_7_)]^+^ system (*57*). These organometallic complexes have been elaborated into aryl-substituted dienes, enones (*52*–*55*), and carbazoles (*58*, *59*). Limited examples of EAS reactions have also appeared in our own work, in the synthesis of γ-substituted enones (*60*) and tetrahydroindolines (*61*), but before our preliminary report of this study (*9*), we had been unable to couple these reactions to a second or third nucleophilic addition. To our knowledge, the double protonation strategy demonstrated herein has not been used previously for arene complexes with any transition metal, aside from tungsten and only in the case of anilines (*19*, *61*).

Last, we note that in prior studies, we have demonstrated the ability to carry out dearomatization reactions with enantioenriched tungsten complexes (*16*, *28*). Key to this success was the observation that arene exchange, with subsequent protonation and addition of nucleophiles does not cause epimerization of the tungsten center. Although we did not focus on the preparation of enantioenriched materials in the present study, we used this methodology to prepare two different diastereomers of β-estradiol (**20D**) and anhydrotetracycline (**21D**) derivatives with dr > 20:1.

The highly electron-rich tungsten system {WTp(NO)(PMe_3_)} enables the double protonation of an anisole ligand, rendering it exceptionally electrophilic. The addition of a soft or π-nucleophile such as an electron-rich arene, followed by reduction and subsequent acid-promoted loss of MeOH, and addition of a more-traditional “hard nucleophile” (typically anionic) results in a highly regio- and stereospecific formation of *cis*-3,6-disubstituted cyclohexenes. Reversing the order of the second and third nucleophilic additions can provide the complementary trans-stereochemistry. The range of accessible functionalities is exceptional, for both soft and hard nucleophiles, allowing the formation of a library of biologically relevant molecules linked through a cyclohexene.

## MATERIALS AND METHODS

Nuclear magnetic resonance spectra were obtained on 500-, 600-, or 800-MHz spectrometers. Chemical shifts are referenced to tetramethylsilane using residual ^1^H or ^13^C signals of the deuterated solvents as internal standards. Infrared spectra were recorded on a spectrometer as a glaze on a diamond anvil attenuated total reflectance (ATR) assembly. All synthetic reactions were performed in a glovebox under a dry nitrogen atmosphere unless otherwise noted. All solvents were sparged with nitrogen before use. Deuterated solvents were used as received from Cambridge Isotopes. Reagents were purchased from commercial vendors and used as received without purification. Compounds WTp(NO)(PMe_3_)(Br), **1**, **2**, and [WTp(NO)(PMe_3_)(5,6-η^2^-4H-1,3-dimethoxybenzenium)](*L*-DBTH) were prepared according to previous literature procedures with some modifications(*10*, *22*, *62*). Compounds **5D-8D**, **13D**, **15D**, **78D**, **19D**, **8P**, **28P**, **27D-30D**, **32D-37D**, *cis*-**38D**, *cis*-**46D**, **51D**, **61-65**, **70**, and **72-74** have previously been published (*9*). Synthetic details, DFT calculations, and spectroscopic and crystallographic characterizations of compounds are provided in the Supplementary Materials.

## Supplementary Materials

### This PDF file includes:

Materials and Methods Figs. S1 to S75 Tables S1 to S6 Legends for data S1 and S2 References
