# Supplementary material for: Tungsten-anisole complex provides 3,6-substituted cyclohexenes for highly diversified chemical libraries
Source: Sci Adv. 2024 Feb 16;10(7):eadl0885. doi: 10.1126/sciadv.adl0885 (PMC10871534; doi:10.1126/sciadv.adl0885)
Supplement: Supplementary file 1 — Materials and Methods Figs. S1 to S75 Tables S1 to S6 Legends for data S1 and S2 References [file sciadv.adl0885_sm.pdf]

Science Advances
AAAS

# Supplementary Materials for

## Tungsten-anisole complex provides 3,6-substituted cyclohexenes for highly diversified chemical libraries

Justin T. Weatherford-Pratt *et al.*

Corresponding author: W. Dean Harman, wdh5z@virginia.edu

*Sci. Adv.* **10**, eadl0885 (2024)
DOI: 10.1126/sciadv.adl0885

**The PDF file includes:**

Materials and Methods
Figs. S1 to S75
Tables S1 to S6
Legends for data S1 and S2
References

**Other Supplementary Material for this manuscript includes the following:**

Data S1 and S2

**Materials and Methods**

Experimental Procedures & Characterizations

**General Methods:** NMR spectra were obtained on 500, 600, or 800 MHz spectrometers. Chemical shifts are referenced to tetramethylsilane (TMS) utilizing residual $^{1}$H or $^{13}$C signals of the deuterated solvents as internal standards. Infrared (IR) spectra were recorded on a spectrometer as a glaze on a diamond anvil ATR assembly. All synthetic reactions were performed in a glovebox under a dry nitrogen atmosphere unless otherwise noted. All solvents were sparged with nitrogen prior to use. Deuterated solvents were used as received from Cambridge Isotopes. Reagents were purchased from commercial vendors and used as received without purification. Compounds WTp(NO)($PMe_3$)(Br), **1**, **2**, and [WTp(NO)($PMe_3$)(5,6-$\eta^2$-4H-1,3-dimethoxybenzenium)](*L*-DBTH) were prepared according to previous literature procedures with some modifications.[10, 19, 62] Compounds **3P**, **3D**, **5D-8D**, **13D**, **15D**, **78D**, **19D**, **8P**, **29P**, **27D-30D**, **34P**, **32D-37D**, ***cis*-38D**, ***cis*-46D**, **51D**, **61-64**, **68**, **70**, and **72-74** have previously been published.[9] The transition-metal fragment WTp(NO)($PMe_3$), where Tp = trispyrazolylborate, is abbreviated as [W].

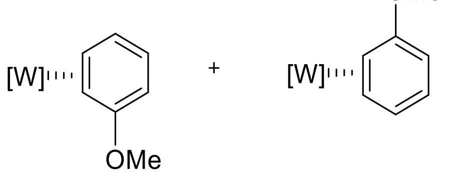


**1 (1D + 1P)**

To a 1 L round bottom flask were added a stir bar, WTp(NO)($PMe_3$)(Br) (45.7298 g, 78.4576 mmol), and anisole (700 mL). To this stirring green mixture was added a sodium dispersion in toluene (33.1286 g, 33% w/w, 475.530 mmol) followed by anisole (300 mL). After stirring for 18 h, the reaction mixture was filtered through a Celite™ plug (600 mL coarse porosity fritted funnel filled 1/3rd with Celite™ set in anisole). The Celite™ plug was rinsed with anisole (~600 mL) and the filtrate was loaded onto a silica column (600 mL coarse porosity fritted funnel filled 3/4th with silica set in anisole). A green band was then eluted with $Et_2O$ (1 L) and discarded. A yellow band was eluted with $Et_2O$ (4 L) and concentrated *in vacuo* to a volume of ~700 mL before adding pentane (1 L). A yellow precipitate was collected on a 150 mL fine porosity fritted funnel and desiccated under static vacuum overnight to yield **1** (24.8400 g, 52%) as a 3:1 ratio of **1P** to **1D**. Characterization of **1** has been reported previously.[10]

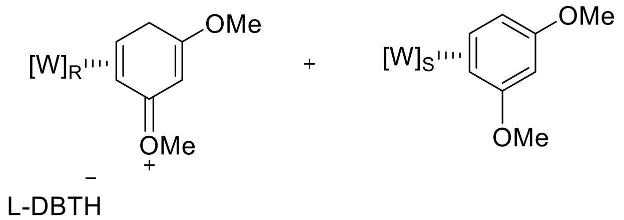


**[*R*-WTp(NO)(PMe$_3$)(5,6-η$^2$-4H-1,3-dimethoxybenzenium)](*L*-DBTH) and *S*-WTp(NO)(PMe$_3$)(5,6-η$^2$-1,3-dimethoxybenzene)**

To a 500 mL round bottom flask were added a stir bar, WTp(NO)($PMe_3$)(5,6-η$^2$-4H-1,3-dimethoxybenzenium)](*L*-DBTH) (14.9565 g, 14.9651 mmol), and 2-butanone (160 mL). To this solution was added a solution of DI $H_2O$ (1.0280 g, 57.063 mmol) in 2-butanone (28 mL). After ~1 min of stirring, a golden precipitate formed. The reaction mixture was allowed to stir for 3 h before the golden precipitate was collected on a 150 mL medium porosity fritted funnel. The filtrate was transferred to a 300 mL Erlenmeyer flask and reserved. The golden precipitate that was collected was washed with 2-butanone (30 mL), $Et_2O$ (100 mL), and pentane (100 mL) before desiccating under static vacuum overnight to yield **[*R*-WTp(NO)(PMe$_3$)(5,6-η$^2$-4H-1,3-dimethoxybenzenium)](*L*-DBTH)** (6.3318 g, 85%). A stir bar and basic alumina (80 mL) were added to the previously reserved filtrate. This mixture was allowed to stir for 5 min before eluting through a basic alumina plug (150 mL coarse porosity fritted funnel filled 1/4th with basic alumina set in THF). The basic alumina plug was rinsed with THF (200 mL) and the golden filtrate was concentrated *in vacuo* to a volume of ~10 mL. Pentane (1 L) was added and a yellow precipitate was collected on a 60 mL medium porosity fritted funnel and washed with 60 mL of pentane (3 x 20 mL). Desiccated under static vacuum overnight to yield ***S*-WTp(NO)(PMe$_3$)(5,6-η$^2$-1,3-dimethoxybenzene)** (2.3537 g, 49%). Characterization of the WTp(NO)($PMe_3$)(5,6-η$^2$-1,3-dimethoxybenzene) and [*R*-WTp(NO)($PMe_3$)(5,6-η$^2$-4H-1,3-dimethoxybenzenium)](*L*-DBTH) has been reported previously.[10, 62]

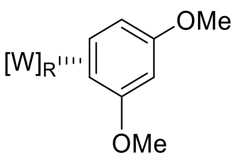


***R*-WTp(NO)(PMe$_3$)(5,6-η$^2$-1,3-dimethoxybenzene)**

To a 300 mL Erlenmeyer flask were added a stir bar, [*R*-WTp(NO)(PMe$_3$)(5,6-η$^2$-4H-1,3-dimethoxybenzenium)](*L*-DBTH) (2.8839 g, 2.8856 mmol), THF (200 mL), and basic alumina (50 mL). This mixture was allowed to stir for 15 min before eluting through a basic alumina plug (350 mL coarse porosity fritted funnel filled 1/4$^{th}$ with basic alumina set in THF). The basic alumina plug was rinsed with THF (300 mL) and the golden filtrate was concentrated *in vacuo* to a volume of ~20 mL. Pentane (500 mL) was added and the solution was concentrated *in vacuo* to a volume of ~200 mL before adding more pentane (300 mL). A yellow precipitate was collected on a 60 mL medium porosity fritted funnel and washed with 60 mL of pentane (3 x 20 mL). Desiccated under static vacuum overnight to yield ***R*-WTp(NO)(PMe$_3$)(5,6-η$^2$-1,3-dimethoxybenzene)** (1.1161 g, 60%). Characterization of the WTp(NO)(PMe$_3$)(5,6-η$^2$-1,3-dimethoxybenzene) has been reported previously.[10]

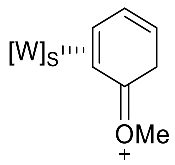


***S*-2D**

To a 4-dram vial were added a stir bar, *S*-WTp(NO)(PMe$_3$)(5,6-η$^2$-1,3-dimethoxybenzene) (1.2095 g, 1.8865 mmol), anisole (5 mL), and THF (5 mL). After stirring for 18 h, the reaction mixture was loaded onto a silica column (350 mL coarse porosity fritted funnel filled 3/4$^{th}$ with silica set in pentane). Once loaded, the column was rinsed with pentane (~300 mL) and the filtrate was discarded. A yellow band was then eluted with THF (~250 mL) and concentrated *in vacuo* to a yellow oil. A chilled (-30 °) solution of HOTf (0.3221 g, 2.146 mmol) in DME (1 mL) was added and after stirring for ~1 min, the resulting red solution was added to stirring $Et_2O$ (1 L). An orange precipitate was collected on a 150 mL medium porosity fritted funnel and washed with 300 mL of $Et_2O$ (2 x 150 mL). Desiccated under static vacuum overnight to yield ***S*-2D** (0.4819 g, 34%). Characterization of **2D** has been reported previously.[19]

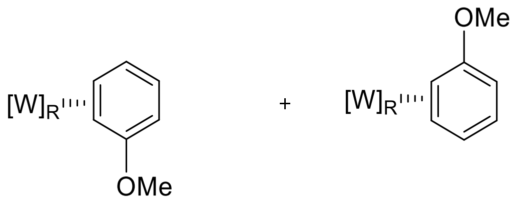


***R*-1 (*R*-1D + *R*-1P)**

To a 50 mL round bottom flask were added a stir bar, *R*-WTp(NO)($PMe_3$)(5,6-$\eta^2$-1,3-dimethoxybenzene) (1.1551 g, 1.8017 mmol), and anisole (~50 mL). After stirring for approximately 18 h, the reaction mixture was loaded onto a silica column (350 mL coarse porosity fritted funnel filled 3/4$^{th}$ with silica set in pentane). Once loaded, the column was rinsed with pentane (~300 mL) and the filtrate was discarded. A yellow band was then eluted with THF (~250 mL) and concentrated *in vacuo* to a yellow oil. Pentane (1 L) was added and a yellow precipitate was collected on a 60 mL medium porosity fritted funnel and desiccated under static vacuum overnight to yield ***R*-1** (0.5452 g, 50%) as a 3:1 ratio of ***R*-1P** to ***R*-1D.** Characterization of the **1** has been reported previously.[10]

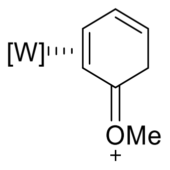


**2D**

To a screw top test tube charged with a stir pea were added **1** (1552 mg, 2.54 mmol) and MeOH (~7 mL). The resulting yellow slurry was chilled to -30 °C in a cold-bath. To a second screw top test tube were added MeOH (~1 mL) and HOTf (574 mg, 3.82 mmol) and the resulting solution was chilled to -30 °C in a cold-bath. The solution of acid was transferred to the slurry of **1**, leading to a dark-red solution. The reaction solution was removed from the cold-bath and allowed to warm to room temperature*, after which the reaction was allowed to stir at room temperature for 75 min. The reaction solution was then transferred to stirring $Et_2O$ (~700 mL), leading to the formation of an orange-yellow precipitate. This precipitate was collected on a 60 mL fine porosity fritted funnel, washed with $Et_2O$ (~30 mL), and desiccated under static vacuum overnight to provide **2D** (1635 mg, 85%). Characterization of **2D** has been reported previously.[19]

***Note:** if the reaction mixture solidifies at this point, addition of the minimal amount of MeOH to provide a homogenous dark-red solution is necessary.

**General Procedure 1:** To a 15-30 mL test tube were added **2D** and MeCN (1-3 mL). This solution was chilled to -30 °C for 5 min before adding a chilled (-30 °C) solution of HOTf in MeCN. A chilled (-30 °C) solution of the nucleophile in MeCN (1-3 mL) was added. The reaction was monitored by $^{31}$P NMR and determined to be complete based on the disappearance of **3D** and the formation of a new signal with $J_{WP}$ ~285 Hz. The reaction mixture was then diluted with DCM (5 mL) and washed with DI $H_2O$ (3 x 5 mL). The organic layer was dried over anhydrous $MgSO_4$ and concentrated *in vacuo*. The film was dissolved in minimal DCM and added to stirring $Et_2O$ (300 mL). The resulting precipitate was collected on a fine porosity fritted funnel and washed with $Et_2O$ (30 mL).

**General Procedure 2:** To a 15-30 mL test tube were added **2D** and MeCN (1-3 mL). This solution was chilled to -30 °C for 5 min before adding a chilled (-30 °C) solution of HOTf in MeCN. To a

chilled (-30 °C) solution of the nucleophile in MeCN (1-3 mL) was added a chilled (-30 °C) solution of HOTf in MeCN. This solution was then added to the solution containing **3D**. The reaction was monitored by $^{31}$P NMR and determined to be complete based on the disappearance of **3D** and the formation of a new signal with $J_{WP}$ ~285 Hz. The reaction mixture was then diluted with DCM (5 mL) and washed with DI $H_2O$ (3 x 5 mL). The organic layer was dried over anhydrous $MgSO_4$ and concentrated *in vacuo*. The film was dissolved in minimal DCM and added to stirring $Et_2O$ (300 mL). The resulting precipitate was collected on a fine porosity fritted funnel and washed with $Et_2O$ (30 mL).

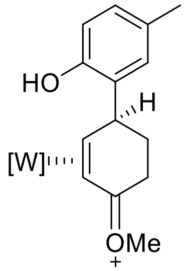


**9D**

Used **General Procedure 1** with **2D** (0.1028 g, 0.1351 mmol), HOTf (0.1099 g, 0.7323 mmol) in MeCN (1 mL), and p-cresol (0.3976 g, 3.6767 mmol) in MeCN (1 mL). Orange-tan solid (0.0695 g, 59%).

**$^{1}$H NMR (600 MHz, $CD_2Cl_2$) δ:** 8.13 (d, $J$ = 2.2 Hz, 1H), 7.94 (d, $J$ = 2.3 Hz, 1H), 7.85 (dt, $J$ = 2.4, 0.8 Hz, 1H), 7.83 (dt, $J$ = 2.5, 0.8 Hz, 1H), 7.76 (d, $J$ = 2.3 Hz, 1H), 7.57 (br s, 1H), 7.46 (d, $J$ = 2.2, 1H), 7.10 (d, $J$ = 1.7, 1H), 6.91 (m, 2H), 6.50 (t, $J$ = 2.3 Hz, 1H), 6.45 (t, $J$ = 2.3 Hz, 1H), 6.35 (t, $J$ = 2.3 Hz, 1H), 4.70 (ddd, $J$ = 10.0, 6.3, 2.4 Hz, 1H), 4.23 (ddd, $J$ = 15.1, 8.1, 2.4 Hz, 1H), 3.29 (d, $J$ = 8.2 Hz, 1H), 3.13 (m, 1H), 3.08 (s, 3H), 2.83 (m, 1H), 2.29 (s, 3H), 2.16 (m, 1H), 1.91 (m, 1H), 1.12 (d, $J$ = 9.4 Hz, 9H). **$^{13}$C NMR (201 MHz, $CD_2Cl_2$) δ:** 195.0, 152.0, 145.0, 144.0, 141.7, 139.1, 138.8, 138.6, 134.6, 130.0, 128.9, 128.7, 117.0, 108.4 108.4, 107.9, 77.0 (d, $J_{CP}$ = 15.1 Hz), 66.0, 57.7, 38.1, 32.5, 30.1, 20.8, 14.1 (d, $J_{CP}$ = 31.6 Hz). Composition confirmed by single crystal X-ray diffraction.

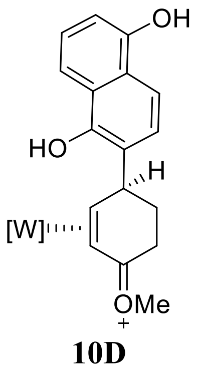


**10D**

Used **General Procedure 1** with **2D** (0.1708 g, 0.2244 mmol), HOTf (0.1213 g, 0.8082 mmol) in MeCN (1 mL), and 1,5-dihydroxynaphthalene (0.0695 g, 0.4339 mmol) in MeCN (1 mL). Orange-tan solid (0.1178 g, 57%).

**$^1$H NMR (800 MHz, $(CD_3)_2CO$) δ:** 8.38 (d, *J* = 1.9 Hz, 1H), 8.19 (d, *J* = 2.3 Hz, 1H), 8.12 (m, 2H), 8.11 (d, *J* = 2.6 Hz, 1H), 7.94 (d, *J* = 8.7 Hz, 1H), 7.78 (d, *J* = 8.4 Hz, 1H), 7.73 (d, *J* = 8.7 Hz, 1H), 7.63 (d, *J* = 1.8 Hz, 1H), 7.32 (t, *J* = 7.9 Hz, 1H), 6.95 (d, *J* = 7.4 Hz, 1H), 6.56 (m, 2H), 6.48 (t, *J* = 2.4 Hz, 1H), 5.25 (m, 1H), 4.41 (dd, *J* = 15.2, 8.1 Hz, 1H), 3.67 (d, *J* = 8.0 Hz, 1H), 3.26 (m, 4H), 2.89 (dt, *J* = 17.7, 4.0 Hz, 1H), 2.29 (m, 1H), 1.89 (m, 1H), 1.19 (d, *J* = 9.6 Hz, 9H). **$^{13}$C NMR (201 MHz, $(CD_3)_2CO$) δ:** 195.3, 154.0, 148.6, 146.0, 144.7, 139.8, 139.4, 139.2, 131.1, 127.6, 126.5, 125.5, 125.4, 125.3, 116.3, 113.5, 108.9, 108.8, 108.6, 108.4, 76.3 (d, $J_{CP}$ = 14.9 Hz), 66.9, 58.1, 36.9, 33.8, 30.2, 13.7 (d, $J_{CP}$ = 31.7 Hz). Composition confirmed by single crystal X-ray diffraction.

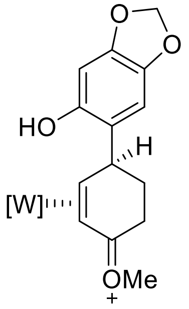


**11D**

Used **General Procedure 1** with **2D** (0.7509 g, 0.9865 mmol), HOTf (2.5500 g, 16.991 mmol) in MeCN (1 mL), and sesamol (1.1343 g, 8.2124 mmol) in MeCN (1 mL). Orange-tan solid (0.6847 g, 77%).

**$^1$H NMR (800 MHz, $CD_2Cl_2$) δ:** 8.12 (d, *J* = 2.2 Hz, 1H), 7.94 (d, *J* = 2.3 Hz, 1H), 7.85 (d, *J* = 2.5 Hz, 1H), 7.83 (d, *J* = 2.5 Hz, 1H), 7.73 (d, *J* = 2.3 Hz, 1H), 7.45 (d, *J* = 2.2 Hz, 1H), 6.82 (s, 1H), 6.65 (s, 1H), 6.50 (t, *J* = 2.3 Hz, 1H), 6.44 (t, *J* = 2.3 Hz, 1H), 6.34 (t, *J* = 2.3 Hz, 1H), 5.88 (dd, *J* = 12.1, 1.4 Hz, 2H), 4.72 (ddd, *J* = 9.2, 6.3, 2.3 Hz, 1H), 4.11 (ddd, *J* = 15.0, 8.1, 2.4 Hz, 1H), 3.25 (d, *J* = 8.1 Hz, 1H), 3.12 (ddd, *J* = 17.7, 11.4, 5.8 Hz, 1H), 3.06 (s, 3H), 2.80 (ddt, *J* = 18.0, 5.1, 2.3 Hz, 1H), 2.16 (dtd, *J* = 12.7, 6.1, 3.1 Hz, 1H), 1.80 (m, 1H), 1.14 (d, *J* = 9.5 Hz, 9H). **$^{13}$C NMR (201 MHz, $CD_2Cl_2$) δ:** 194.7, 148.8, 146.9, 145.1, 144.0, 141.7, 141.5, 139.1, 138.8, 138.6, 126.8, 108.4, 108.3, 107.9, 107.1, 101.5, 99.2, 76.8 (d, $J_{CP}$ = 15.1 Hz), 66.0, 57.7, 37.1, 32.9, 30.0, 14.1 (d, $J_{CP}$ = 15.1 Hz). Composition confirmed by single crystal X-ray diffraction.

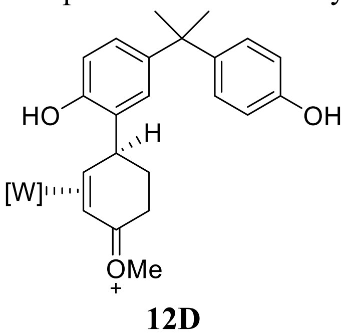


**12D**

Used **General Procedure 1** with **2D** (0.5166 g, 0.6787 mmol), HOTf (0.9522 g, 6.345 mmol) in MeCN (1 mL), and bisphenol A (1.1209 g, 4.9100 mmol) in MeCN (1 mL). Orange-tan solid (0.4385 g, 65%).

**$^1$H NMR (800 MHz, $CD_3CN$) δ:** 8.15 (d, *J* = 2.2 Hz, 1H), 8.02 (d, *J* = 2.4 Hz, 1H), 7.93 (d, *J* = 2.5 Hz, 1H), 7.91 (d, *J* = 2.5 Hz, 1H), 7.47 (d, *J* = 2.3 Hz, 1H), 7.46 (d, *J* = 2.2 Hz, 1H), 7.18 (d, *J*

= 2.4 Hz, 1H), 7.09 (m, 3H), 6.79 (d, $J$ = 8.4 Hz, 1H), 6.73 (d, $J$ = 8.7 Hz, 2H), 6.55 (t, $J$ = 2.3 Hz, 1H), 6.45 (t, $J$ = 2.3 Hz, 1H), 6.35 (t, $J$ = 2.3 Hz, 1H), 4.68 (ddd, $J$ = 9.9, 6.1, 2.4 Hz, 1H), 3.86 (ddd, $J$ = 15.0, 8.1, 2.4 Hz, 1H), 3.17 (m, 1H), 3.02 (s, 3H), 2.99 (m, 1H), 2.66 (m, 1H), 2.12 (m, 1H), 1.64 (m, 7H), 0.99 (d, $J$ = 9.6 Hz, 9H). **$^{13}$C NMR (201 MHz, $CD_3CN$) δ:** 196.8, 155.6, 152.1, 145.9, 144.7, 144.6, 143.7, 142.3, 139.9, 139.6, 139.3, 135.2, 128.8, 128.4, 125.8, 115.5, 115.5, 108.9, 108.8, 108.5, 76.6 (d, $J_{CP}$ = 14.9 Hz), 66.1, 58.5, 42.5, 37.4, 33.1, 31.2, 31.0, 30.2, 13.8 (d, $J_{CP}$ = 31.7 Hz). **ESI-HRMS (m/z):** $[M]^+$ calculated for $C_{34}H_{44}BN_7O_4PW$ 840.2789; found, 840.2782.

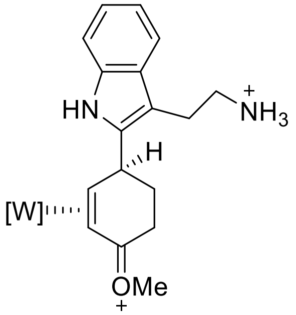


**14D**

Used **General Procedure 2** with **2D** (0.1034 g, 0.1358 mmol), HOTf (0.1048 g, 0.6983 mmol) in MeCN (1 mL), tryptamine (0.4051 g, 2.5285 mmol) in MeCN (1 mL), and a separate solution of HOTf (0.4931 g, 3.286 mmol) in MeCN (1 mL) to protonate the nucleophile. Orange-tan solid (0.0920 g, 63%).

**$^1$H NMR (600 MHz, $CD_3CN$) δ:** 9.80 (s, 1H), 8.20 (d, $J$ = 2.3 Hz, 1H), 8.04 (d, $J$ = 2.4 Hz, 1H), 7.97 (d, $J$ = 2.4 Hz, 1H), 7.95 (d, $J$ = 2.5 Hz, 1H), 7.89 (d, $J$ = 2.3 Hz, 1H), 7.59 (d, $J$ = 7.9 Hz, 1H), 7.53 (d, $J$ = 2.2 Hz, 1H), 7.49 (d, $J$ = 8.0 Hz, 1H), 7.17 (t, $J$ = 7.5 Hz, 1H), 7.11 (t, $J$ = 7.5 Hz, 1H), 6.51 – 6.49 (br m, 5H), 6.40 (t, $J$ = 2.3 Hz, 1H), 4.61 (ddd, $J$ = 9.3, 6.3, 2.3 Hz, 1H), 4.20 (ddd, $J$ = 14.8, 8.0, 2.4 Hz, 1H), 3.44 (d, $J$ = 7.8 Hz, 1H), 3.31 – 3.04 (m, 8H), 2.83 (m, 1H), 2.27 (dtd, $J$ = 12.3, 6.0, 3.6 Hz, 1H), 1.97 (m, 1H), 1.05 (d, $J$ = 9.6 Hz, 9H). **$^{13}$C NMR (201 MHz, $CD_3CN$) δ:** 196.5, 145.8, 144.8, 143.2, 142.8, 140.0, 139.7, 139.4, 137.2, 128.6, 122.6, 120.3, 119.0, 112.3, 109.0, 108.7, 108.6, 105.5, 73.1(d, $J_{CP}$ = 15.9 Hz), 66.4, 58.6, 41.5, 35.8, 33.3, 29.8, 23.2, 13.7 (d, $J_{CP}$ = 32.0 Hz). Composition confirmed by single crystal X-ray diffraction.

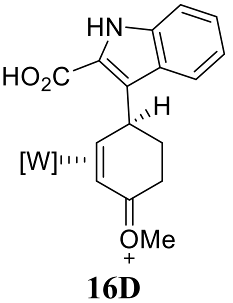


**16D**

Used **General Procedure 1** with **2D** (0.7582 g, 0.9961 mmol), HOTf (2.5500 g, 16.991 mmol) in MeCN (1 mL), and indole-2-carboxylic acid (1.2391 g, 7.6886 mmol) in MeCN (1 mL). Orange-tan solid (0.6003 g, 65%).

**$^{1}$H NMR (800 MHz, $CD_3CN$) δ:** 10.17 (s, 1H), 8.18 (d, $J$ = 2.2 Hz, 1H), 8.15 (d, $J$ = 8.2 Hz, 1H), 8.00 (d, $J$ = 2.3 Hz, 1H), 7.93 (m, 2H), 7.61 (d, $J$ = 2.3 Hz, 1H), 7.55 (d, $J$ = 8.3 Hz, 1H), 7.52 (d, $J$ = 2.2 Hz, 1H), 7.34 (t, $J$ = 7.6 Hz, 1H), 7.15 (t, $J$ = 7.5 Hz, 1H), 6.44 (m, 2H), 6.38 (t, $J$ = 2.4 Hz, 1H), 5.80 (t, $J$ = 9.4 Hz, 1H), 4.46 (ddd, $J$ = 15.2, 8.0, 2.4 Hz, 1H), 3.49 (d, $J$ = 8.0 Hz, 1H), 3.24 (m, 1H), 3.07 (s, 3H), 2.78 (dq, $J$ = 17.3, 2.5 Hz, 1H), 2.07 (ddt, $J$ = 12.2, 8.0, 4.1 Hz, 2H), 1.00 (d, $J$ = 9.7 Hz, 9H). **$^{13}$C NMR (201 MHz, $CD_3CN$) δ:** 194.8, 165.0, 145.9, 144.8, 142.5, 140.0, 139.7, 139.2, 137.5, 129.2, 126.3, 126.1, 125.7, 120.7, 113.6, 108.9, 108.7, 108.6, 107.0, 76.4 (d, $J_{CP}$ = 14.5 Hz), 67.1, 58.4, 35.1, 33.8, 30.6, 13.8 (d, $J_{CP}$ = 31.6 Hz). **APCI-HRMS (m/z):** $[M]^+$ calculated for $C_{28}H_{35}BN_8O_4PW$ 773.2116; found, 773.2086.

***Note:** The signal at 3.42 ppm overlaps with a $Et_2O$ solvent impurity.

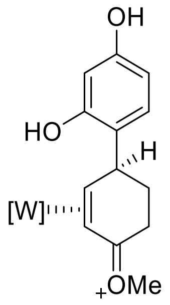


**17D**

Used **General Procedure 1** with **2D** (0.3044 g, 0.3999 mmol), HOTf (0.6977 g, 4.649 mmol) in MeCN (1 mL), and resorcinol (1.2824 g, 11.646 mmol) in MeCN (1 mL). Orange-tan solid (0.1998 g, 57%).

**$^{1}$H NMR (800 MHz, $CD_2Cl_2$) δ:** 8.12 (d, $J$ = 2.1 Hz, 1H), 7.93 (d, $J$ = 2.3 Hz, 1H), 7.84 (d, $J$ = 2.4 Hz, 1H), 7.81 (d, $J$ = 2.5 Hz, 1H), 7.73 (d, $J$ = 2.3 Hz, 1H), 7.49 (s, 1H), 7.45 (d, $J$ = 2.1 Hz, 1H), 7.14 (d, $J$ = 8.3 Hz, 1H), 6.71 (br s, 1H), 6.59 (d, $J$ = 2.5 Hz, 1H), 6.51 (dd, $J$ = 8.4, 2.5 Hz, 1H), 6.49 (t, $J$ = 2.3 Hz, 1H), 6.44 (t, $J$ = 2.3 Hz, 1H), 6.34 (t, $J$ = 2.3 Hz, 1H), 4.64 (ddd, $J$ = 9.2, 6.3, 2.4 Hz, 1H), 4.19 (ddd, $J$ = 15.0, 8.1, 2.4 Hz, 1H), 3.25 (d, $J$ = 8.0 Hz, 1H), 3.10 (m, 1H), 3.07 (s, 3H), 2.82 (m, 1H), 2.15 (dtd, $J$ = 12.8, 6.1, 3.4 Hz, 1H), 1.87 (m, 1H), 1.12 (d, $J$ = 9.4 Hz, 9H). **$^{13}$C NMR (201 MHz, $CD_2Cl_2$) δ:** 195.4, 156.5, 154.8, 145.1, 144.0, 141.6, 139.0, 138.7, 138.6, 129.1, 126.7, 108.4, 108.4, 108.3, 107.9, 104.1, 77.1 (d, $J_{CP}$ = 15.2 Hz), 65.9, 57.8, 37.4, 32.5, 30.1, 14.1 (d, $J_{CP}$ = 31.4 Hz). Composition confirmed by single crystal X-ray diffraction.

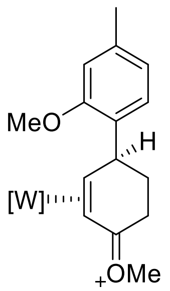


**18D**

Used **General Procedure 1** with **2D** (0.1167 g, 0.1533 mmol), HOTf (0.1398 g, 0.9315 mmol) in MeCN (1 mL), and 3-methylanisole (0.2482 g, 2.032 mmol) in MeCN (1 mL). Orange-tan solid (0.0871 g, 64%).

**$^1$H NMR (600 MHz, $(CD_3)_2CO$) δ:** 8.36 (d, $J$ = 2.3 Hz, 1H), 8.19 (d, $J$ = 2.3 Hz, 1H), 8.15 – 8.08 (m, 3H), 7.60 (d, $J$ = 2.2 Hz, 1H), 7.49 (d, $J$ = 7.7 Hz, 1H), 6.88 (s, 1H), 6.86 (d, $J$ = 7.0 Hz, 1H), 6.57 (t, $J$ = 2.3 Hz, 1H), 6.56 (t, $J$ = 2.3 Hz, 1H), 6.47 (t, $J$ = 2.3 Hz, 1H), 4.91 (ddd, $J$ = 9.2, 6.3, 2.3 Hz, 1H), 4.28 (m, 1H), 3.89 (s, 3H), 3.60 (dt, $J$ = 8.1, 1.7 Hz, 1H), 3.24 (s, 3H), 3.17 (m, 1H), 2.84 (m, 1H), 2.34 (s, 3H), 2.17 (dtd, $J$ = 12.7, 6.1, 3.5 Hz, 1H), 1.74 (dtd, $J$ = 13.4, 10.5, 5.6 Hz, 1H), 1.20 (d, $J$ = 9.6 Hz, 9H). **$^{13}$C NMR (201 MHz, $(CD_3)_2CO$) δ:** 195.5, 157.0, 146.1, 144.8, 143.0, 140.0, 139.6, 139.4, 138.3, 135.1, 128.9, 122.8, 112.5, 109.0, 108.8, 108.6, 76.6 (d, $J_{CP}$ = 15.3 Hz), 66.9, 58.2, 55.9, 36.2, 33.7, 30.1, 21.5, 13.8 (d, $J_{CP}$ = 31.8 Hz). Composition confirmed by single crystal X-ray diffraction.

**General Procedure 3:** To a 15-30 mL test tube was added **1**. The complex was chilled to -60 °C for 15 min before adding a chilled (-60 °C) solution of HOTf in EtCN (2-3 mL). After 5 min, a chilled (-60 °C) solution of the nucleophile in EtCN (1-2 mL) was added. The reaction was then allowed to stir at room temperature and was monitored by $^{31}$P NMR and determined to be complete based on the disappearance of **3P** and the formation of a new signal with $J_{WP}$ ~290 Hz. The reaction mixture was then diluted with DCM (5-10 mL) and washed with 15 mL of DI $H_2O$ (3 x 5 mL). The organic layer was dried over anhydrous $MgSO_4$ and concentrated *in vacuo*. The film was dissolved in minimal DCM and added to stirring $Et_2O$. The resulting precipitate was collected on a fine porosity fritted funnel and washed with $Et_2O$.

**General Procedure 4:** To a 15-30 mL test tube was added **1**. The complex was chilled to -60 °C for 15 min before adding a chilled (-60 °C) solution of HOTf in EtCN (2-3 mL). To a chilled (-60 °C) solution of the nucleophile in EtCN (1-3 mL) was added a chilled (-60 °C) solution of HOTf in EtCN. This solution was then added to the solution containing **3P**. The reaction was then allowed to stir at room temperature and was monitored by $^{31}$P NMR and determined to be complete based on the disappearance of **3P** and the formation of a new signal with $J_{WP}$ ~290 Hz. The reaction mixture was then diluted with DCM (5-10 mL) and washed with 15 mL of DI $H_2O$ (3 x 5 mL). The organic layer was dried over anhydrous $MgSO_4$ and concentrated *in vacuo*. The film was dissolved in minimal DCM and added to stirring $Et_2O$. The resulting precipitate was collected on a fine porosity fritted funnel and washed with $Et_2O$.

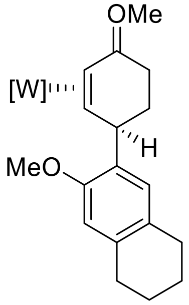


**5P**

Used **General Procedure 3** with **1** (0.7460 g, 1.221 mmol), HOTf (1.0274 g, 6.8457 mmol) in EtCN (3 mL), and 6-methoxy-1,2,3,4-tetrahydronaphthalene (0.6771 g, 3.843 mmol) in EtCN (1 mL). Tan-orange solid (0.9218 g, 82%).

**$^1$H NMR (800 MHz, $(CD_3)_2CO$) δ:** 8.33 (d, $J$ = 2.1 Hz, 1H), 8.18 (d, $J$ = 2.3 Hz, 1H), 8.16 (m, 2H), 7.97 (m, 2H), 7.10 (s, 1H), 6.60 (s, 1H), 6.57 (t, $J$ = 2.3 Hz, 1H), 6.53 (t, $J$ = 2.3 Hz, 1H), 6.39 (t, $J$ = 2.3 Hz, 1H), 4.99 (t, $J$ = 8.8 Hz, 1H), 4.56 (s, 3H), 4.39 (m, 1H), 3.70 (s, 3H), 3.20 (dt, $J$ = 20.2, 7.3 Hz, 1H), 2.98 (ddd, $J$ = 20.8, 7.8, 3.0 Hz, 1H), 2.73 – 2.58 (m, 5H), 2.45 (m, 1H), 2.03 (m, 1H)*, 1.71 (m, 4H), 1.24 (d, $J$ = 9.3 Hz, 9H). **$^{13}$C NMR (201 MHz, $(CD_3)_2CO$) δ:** 210.1, 155.6, 144.4, 143.3, 143.1, 139.3, 138.9, 138.4, 136.5, 135.8, 129.4, 128.9, 112.0, 108.8, 108.4, 107.4, 75.3, 64.0, 60.0, 56.0, 33.9, 29.9, 29.6, 28.1, 27.6, 24.3, 24.1, 12.0 (d, $J_{CP}$ = 31.8 Hz). Composition confirmed by single crystal X-ray diffraction.

***Note:** The signal at 2.03 ppm overlaps with a $(CD_3)_2CO$ residual solvent peak.

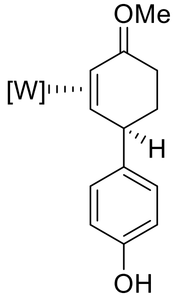


**6P**

Used **General Procedure 3** with **1** (0.7639 g, 1.250 mmol), HOTf (1.152 g, 7.676 mmol) in EtCN (3 mL), and phenol (1.4076 g, 14.957 mmol) in EtCN (1 mL). Tan-orange solid (0.8040 g, 75%).

**$^1$H NMR (800 MHz, $CD_2Cl_2$) δ:** 8.11 (d, $J$ = 2.1 Hz, 1H), 7.90 (m, 2H), 7.75 (d, $J$ = 2.5 Hz, 1H), 7.70 (d, $J$ = 2.1 Hz, 1H), 7.59 (d, $J$ = 2.3 Hz, 1H), 7.16 (d, $J$ = 8.5 Hz, 2H), 6.85 (d, $J$ = 8.6 Hz, 2H), 6.47 (t, $J$ = 2.3 Hz, 1H), 6.38 (t, $J$ = 2.3 Hz, 1H), 6.31 (t, $J$ = 2.3 Hz, 1H), 4.67 (t, $J$ = 9.0 Hz, 1H), 4.27 (s, 3H), 3.71 (m, 1H), 2.83 (dd, $J$ = 20.6, 7.5 Hz, 1H), 2.68 (m, 2H), 2.45 (m, 1H), 2.07 (m, 1H), 1.10 (d, $J$ = 9.1 Hz, 9H). **$^{13}$C NMR (201 MHz, $CD_2Cl_2$) δ:** 206.1, 155.8, 143.0, 142.3, 141.6, 139.7, 138.4, 138.2, 137.5, 128.7, 115.9, 108.2, 108.0, 107.0, 74.4, 63.3, 59.3, 38.5, 28.0, 27.2, 11.9 (d, $J_{CP}$ = 30.3 Hz). **APCI-HRMS (m/z):** $[M]^+$ calculated for $C_{25}H_{34}BFeN_7O_3PW$ 706.2058; found, 706.2056.

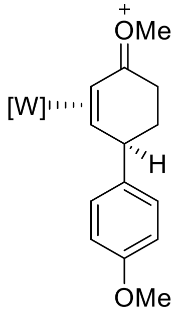


**7P**

Used **General Procedure 3** with **1** (0.2966 g, 0.4854 mmol), HOTf (0.3518 g, 2.344 mmol) in EtCN (3 mL), and anisole (1.5228 g, 14.082 mmol) in EtCN (1 mL). Tan-orange solid (0.3032 g, 72%).

**$^1$H NMR (600 MHz, $(CD_3)_2CO$) δ:** 8.32 (d, $J$ = 2.1 Hz, 1H), 8.19 (m, 2H), 8.17 (d, $J$ = 2.4 Hz, 1H), 7.98 (d, $J$ = 2.5 Hz, 1H), 7.96 (d, $J$ = 2.0 Hz, 1H), 7.33 (d, $J$ = 8.5 Hz, 2H), 6.83 (d, $J$ = 8.8 Hz, 2H), 6.58 (t, $J$ = 2.3 Hz, 1H), 6.54 (t, $J$ = 2.3 Hz, 1H), 6.39 (t, $J$ = 2.3 Hz, 1H), 5.06 (t, $J$ = 9.0 Hz, 1H), 4.54 (s, 3H), 3.90 (m, 1H), 3.75 (s, 3H), 3.09 (m, 1H), 2.99 (dd, $J$ = 21.0, 7.6 Hz, 1H), 2.65 (m, 1H), 2.58 (dq, $J$ = 14.5, 7.8 Hz, 1H), 2.11 (m, 1H)*, 1.24 (d, $J$ = 9.4 Hz, 9H). **$^{13}$C NMR (201 MHz, $(CD_3)_2CO$) δ:** 207.7, 159.2, 144.2, 143.3, 143.2, 142.2, 139.3, 138.9, 138.3, 129.4, 114.7, 108.8, 108.5, 107.7, 74.9 (d, $J_{CP}$ = 9.9 Hz), 63.3 (d, $J_{CP}$ = 5.4 Hz), 60.0, 55.5, 39.2, 28.4, 27.5, 11.9 (d, $J_{CP}$ = 31.1 Hz).

***Note:** The signal at 2.11 ppm overlaps with an acetone solvent impurity.

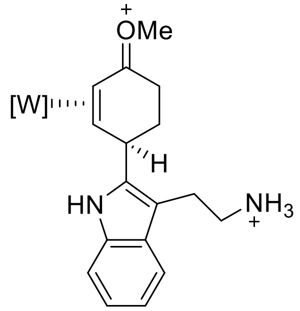


**14P**

Used **General Procedure 4** with **1** (0.4171 g, 0.6825 mmol), HOTf (0.4469 g, 2.978 mmol) in EtCN (2 mL), tryptamine (0.2786 g, 1.739 mmol) in EtCN (2 mL), and a separate solution of HOTf (0.4264 g, 2.842 mmol) in EtCN (1 mL) to protonate the nucleophile. Tan solid (0.3814 g, 52%).

**$^1$H NMR (800 MHz, $(CD_3)_2CO$) δ:** 10.07 (s, 1H), 8.31 (d, $J$ = 2.2 Hz, 1H), 8.18 – 8.10 (m, 4H), 7.94 (d, $J$ = 2.5 Hz, 1H), 7.65 (br m, 3H), 7.52 (d, $J$ = 8.1 Hz, 2H), 7.03 (t, $J$ = 7.6 Hz, 1H), 6.97 (t, $J$ = 7.5 Hz, 1H), 6.57 (t, $J$ = 2.3 Hz, 1H), 6.49 (m, 2H), 5.25 (t, $J$ = 9.1 Hz, 1H), 4.53 (s, 3H), 4.35 (d, $J$ = 6.6 Hz, 1H), 3.44 – 3.19 (m, 4H), 3.11 (dt, $J$ = 21.0, 8.2 Hz, 1H), 3.04 (dd, $J$ = 20.9, 7.5 Hz, 1H), 2.68 (m, 1H), 2.60 (d, $J$ = 8.4 Hz, 1H), 2.16 (dd, $J$ = 14.7, 7.8 Hz, 1H), 1.23 (d, $J$ = 9.5 Hz, 9H). **$^{13}$C NMR (201 MHz, $(CD_3)_2CO$) δ:** 207.7, 144.1, 144.0, 143.9, 143.5, 139.1, 138.8, 138.2, 136.8, 129.2, 121.8, 120.0, 118.3, 112.8, 108.8, 108.4, 108.1, 105.3, 70.7, 62.7, 59.9, 41.8, 31.8, 27.8, 27.5, 23.0, 11.9 (d, $J_{CP}$ = 30.6 Hz). Composition confirmed by single crystal X-ray diffraction.

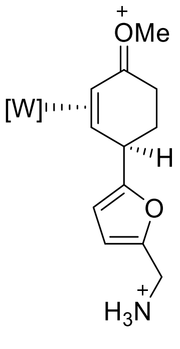

**15P**

Used **General Procedure 4** with **1** (0.2547 g, 0.4168 mmol), HOTf (0.2376 g, 1.583 mmol) in EtCN (2 mL), furfurylamine (0.1997 g, 2.056 mmol) in EtCN (2 mL), and a separate solution of HOTf (0.4264 g, 2.842 mmol) in EtCN (1 mL) to protonate the nucleophile. Tan solid (0.1121 g, 27%).

**$^{1}$H NMR (800 MHz, $(CD_3)_2CO$) δ:** 8.32 (d, $J$ = 2.1 Hz, 1H), 8.20 (d, $J$ = 2.4 Hz, 1H), 8.18 (d, $J$ = 2.4 Hz, 1H), 8.15 (d, $J$ = 2.3 Hz, 1H), 8.00 (d, $J$ = 2.5 Hz, 1H), 7.85 (d, $J$ = 2.1 Hz, 1H), 6.58 (t, $J$ = 2.3 Hz, 1H), 6.54 (t, $J$ = 2.3 Hz, 1H), 6.52 (d, $J$ = 3.3 Hz, 1H), 6.41 (t, $J$ = 2.3 Hz, 1H), 6.28 (d, $J$ = 3.2 Hz, 1H), 4.93 (t, $J$ = 9.2 Hz, 1H), 4.51 (s, 3H), 4.43 (m, 2H), 3.74 (m, 1H), 3.28 (m, 1H), 2.95 (dd, $J$ = 21.1, 7.7 Hz, 1H), 2.73 (dt, $J$ = 8.6, 2.4 Hz, 1H), 2.44 (m, 1H), 2.31 (m, 1H), 1.22 (d, $J$ = 9.4 Hz, 9H). **$^{13}$C NMR (201 MHz, $(CD_3)_2CO$) δ:** 208.2, 165.1, 144.2, 144.2, 143.5, 143.4, 139.3, 138.9, 138.3, 112.9, 108.8, 108.6, 107.8, 107.4, 72.5, 62.6, 60.1, 37.6, 34.5, 27.8, 24.7, 11.8 (d, $J_{CP}$ = 29.8 Hz). Composition confirmed by single crystal X-ray diffraction.

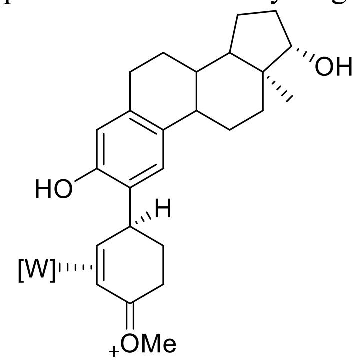


**20D**

Used **General Procedure 1** with **2D** (0.5768 g, 0.7578 mmol), HOTf (0.6621 g, 4.411 mmol) in MeCN (3 mL), and estradiol (0.5972 g, 2.192 mmol) in MeCN (3 mL). Tan solid (0.4497 g, 57%).

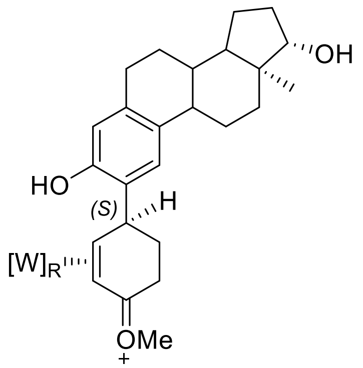


***(WR, 4S, 9'S)*-20D**

Used **General Procedure 1** with **(R)-2D** (0.3101 g, 0.4074 mmol), HOTf (1.0777 g, 7.1808 mmol) in MeCN (3 mL), and estradiol (0.5502 g, 2.020 mmol) in MeCN (3 mL). Tan solid (0.2601 g, 62%).

**$^{1}$H NMR (800 MHz, $CD_3CN$) δ:** 8.18 (d, $J$ = 2.2 Hz, 1H), 8.02 (d, $J$ = 2.4 Hz, 1H), 7.94 (d, $J$ = 2.4 Hz, 1H), 7.92 (d, $J$ = 2.5 Hz, 1H), 7.82 (d, $J$ = 2.3 Hz, 1H), 7.50 (d, $J$ = 2.2 Hz, 1H), 7.39 (s, 1H), 6.54 (s, 1H), 6.50 (t, $J$ = 2.3 Hz, 1H), 6.47 (t, $J$ = 2.3 Hz, 1H), 6.37 (t, $J$ = 2.3 Hz, 1H), 4.76

(ddd, $J$ = 9.1, 6.1, 2.3 Hz, 1H), 4.08 (ddd, $J$ = 15.1, 8.1, 2.4 Hz, 1H), 3.64 (t, $J$ = 8.5 Hz, 1H), 3.34 (d, $J$ = 8.1 Hz, 1H), 3.07 (m, 4H), 2.79 (m. 1H), 2.72 (m, 1H), 2.45 (m, 1H), 2.21 (m, 1H), 2.09 (m, 1H)*, 2.01 (m, 1H), 1.89 (m, 2H), 1.71 (m, 1H), 1.50 – 1.14 (m, 9H), 1.07 (d, $J$ = 9.6 Hz, 9H), 0.76 (s, 3H). **$^{13}$C NMR (201 MHz, $CD_3CN$) δ:** 196.0, 151.9, 145.9, 144.8, 142.8, 139.9, 139.6, 139.3, 136.9, 133.8, 133.2, 126.2, 116.0, 108.9, 108.6, 108.5, 82.0, 76.5 (d, $J_{CP}$ = 15.2 Hz), 66.8, 58.4, 50.9, 45.2, 44.1, 39.9, 37.8, 36.8, 33.8, 30.9, 30.2, 29.8, 28.1, 27.4, 23.8, 13.8, 11.7 (d, $J_{CP}$ = 30.8 Hz). **ESI-HRMS (m/z):** $[M]^+$ calculated for $C_{37}H_{52}BN_7O_4PW$ 884.3420; found, 884.3420.

***Note:** The signal at 2.09 ppm overlaps with an acetone solvent impurity.

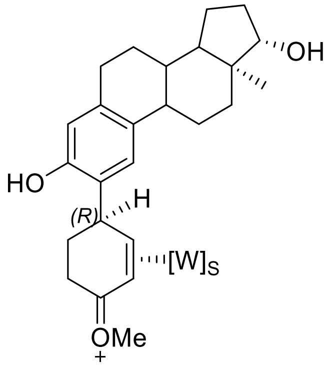


***(WS, 4R, 9'S)*-20D**

Used **General Procedure 1** with **(S)-2D** (0.0581 g, 0.0763 mmol), HOTf (0.0783 g, 0.522 mmol) in MeCN (1 mL), and estradiol (0.1127 g, 0.4138 mmol) in MeCN (2 mL). Tan solid (0.0366 g, 46%).

**$^1$H NMR (800 MHz, $CD_3CN$) δ:** 8.18 (d, $J$ = 2.2 Hz, 1H), 8.03 (d, $J$ = 2.4 Hz, 1H), 7.94 (d, $J$ = 2.5 Hz, 1H), 7.93 (d, $J$ = 2.4 Hz, 1H), 7.74 (d, $J$ = 2.3 Hz, 1H), 7.50 (d, $J$ = 2.1 Hz, 1H), 7.35 (s, 1H), 6.87 (s, 1H), 6.55 (s, 1H), 6.51 (t, $J$ = 2.3 Hz, 1H), 6.47 (t, $J$ = 2.3 Hz, 1H), 6.37 (t, $J$ = 2.4 Hz, 1H), 4.74 (ddd, $J$ = 9.0, 6.3, 2.3 Hz, 1H), 4.06 (m, 1H), 3.63 (m, 1H), 3.35 (d, $J$ = 8.0 Hz, 1H), 3.11 – 3.00 (m, 4H), 2.78 (m, 1H), 2.63 (d, $J$ = 5.3 Hz, 1H), 2.40 (m, 1H), 2.14 (m, 2H), 1.99 (m, 1H), 1.89 (m, 2H), 1.69 (m, 2H), 1.56 (qd, $J$ = 13.1, 4.0 Hz, 1H), 1.50 – 1.14 (m, 8H), 1.10 (d, $J$ = 9.6 Hz, 9H), 0.77 (s, 3H). **$^{13}$C NMR (201 MHz, $CD_3CN$) δ:** 196.2, 151.9, 145.9, 144.7, 142.7, 139.9, 139.6, 139.3, 136.9, 133.8, 133.5, 125.8, 116.1, 108.9, 108.7, 108.5, 81.9, 76.9 (d, $J_{CP}$ = 14.7 Hz), 66.5, 58.5, 50.9, 45.1, 44.1, 40.0, 37.7, 36.5, 33.2, 30.9, 30.1, 29.9, 28.1, 27.4, 23.8, 13.8, 11.7 (d, $J_{CP}$ = 31.8 Hz).

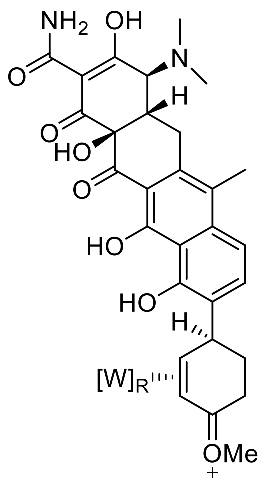


***(WR, 4S, 5a'S)*-21D**

Used **General Procedure 2** with **(R)-10D** (0.3767 g, 0.4948 mmol), HOTf (0.9170 g, 6.110 mmol) in MeCN (1 mL), tetracycline (0.3941 g, 0.8867 mmol) in MeCN (4 mL), and a separate solution of HOTf (0.4195 g, 2.795 mmol) in MeCN (1 mL) to protonate the nucleophile. Chloroform (~15 mL) used during extraction step. Gold-tan solid (0.4127 g, 62%).

**$^1$H NMR (600 MHz, $CD_3CN$) δ:** 8.21 (d, *J* = 2.2 Hz, 1H), 8.03 (d, *J* = 2.3 Hz, 1H), 8.01 (d, *J* = 8.8 Hz, 1H), 7.95 (d, *J* = 2.5 Hz, 1H), 7.94 (d, *J* = 2.5 Hz, 1H), 7.84 (d, *J* = 2.4 Hz, 1H), 7.66 (d, *J* = 8.8 Hz, 1H), 7.52 (d, *J* = 2.2 Hz, 1H), 6.50 (t, *J* = 2.3 Hz, 1H), 6.48 (t, *J* = 2.3 Hz, 1H), 6.38 (t, *J* = 2.4 Hz, 1H), 5.05 (ddd, *J* = 9.0, 6.2, 2.3 Hz, 1H), 4.13 (ddd, *J* = 15.0, 8.2, 2.3 Hz, 1H), 4.06 (m, 1H), 3.57 (m, 1H), 3.40 (m, 2H), 3.10 (m, 5H), 2.98 (m, 6H), 2.81 (m, 1H), 2.45 (s, 3H), 2.19 (m, 1H), 1.80 (m, 1H), 1.07 (d, *J* = 9.6 Hz, 9H). **ESI-HRMS (m/z):** $[M]^+$ calculated for $C_{41}H_{50}BN_9O_9PW$ 1038.3066; found, 1038.3071.

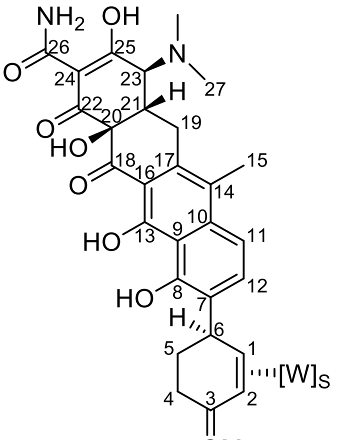


***(WS, 4R, 5a'S)*-21D**

Used **General Procedure 1** with **(S)-2D** (0.2110 g, 0.2772 mmol), HOTf (0.2764 g, 1.842 mmol) in MeCN (1 mL), tetracycline (0.2318 g, 0.5216 mmol) in MeCN (3 mL), and a separate solution of HOTf (0.1616 g, 1.076 mmol) in MeCN (1 mL) to protonate the nucleophile. Gold-tan solid (0.2403 g, 65%).

**$^1$H NMR (800 MHz, $CD_3CN$) δ:** 8.21 (d, *J* = 2.2 Hz, 1H, Pz3/5), 8.03 (d, *J* = 2.3 Hz, 1H, Pz3/5), 7.99 (d, *J* = 8.7 Hz, 1H, H12), 7.95 (d, *J* = 2.4 Hz, 1H, Pz3/5), 7.94 (d, *J* = 2.6 Hz, 1H, Pz3/5), 7.83 (d, *J* = 2.3 Hz, 1H, Pz3/5), 7.65 (d, *J* = 8.8 Hz, 1H, H11), 7.52 (d, *J* = 2.2 Hz, 1H, Pz3/5), 6.49 (t, *J* = 2.3 Hz, 1H, Pz4), 6.48 (t, *J* = 2.4 Hz, 1H, Pz4), 6.39 (t, *J* = 2.3 Hz, 1H, Pz4), 5.05 (ddd, *J* = 9.2, 6.4, 2.2 Hz, 1H, H6), 4.12 (ddd, *J* = 15.0, 8.0, 2.3 Hz, 1H, H1), 4.03 (m, 1H, H23), 3.55 (dd, *J* = 17.6, 5.4 Hz, 1H, H19x), 3.40 (overlapping, 2H, H2 and H21), 3.15 (overlapping, 2H, H4x and H19y), 3.09 (s, 3H, OMe), 3.01 (br s, 6H, H27), 2.81 (dt, *J* = 15.7, 3.6 Hz, 1H, H4y), 2.43 (s, 3H, H15), 2.21 (m, 1H, H5x), 1.78 (m, 1H, H5y), 1.07 (d, *J* = 9.6 Hz, 9H, $PMe_3$). **$^{13}$C NMR (201 MHz, $CD_3CN$) δ:** 198.6, 195.5, 174.4, 165.5, 159.4, 155.0, 145.9, 144.8, 142.8, 140.0, 139.7, 139.3, 139.0, 133.9, 131.7, 130.1, 126.2, 117.2, 112.8, 109.4, 109.0, 108.6, 108.6, 99.1, 77.2, 75.5 (d, $J_{CP}$ = 14.7 Hz), 69.0, 66.7, 58.4, 44.1, 37.6, 35.8, 33.1, 30.8, 30.1, 28.0, 14.6, 13.8 (d, $J_{CP}$ = 31.8 Hz).

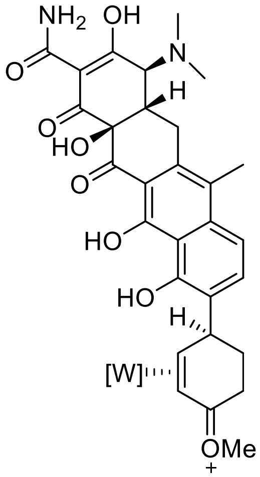


**21D**

To a screw top test tube charged with a stir pea were added **2D** (400 mg, 0.526 mmol) and EtCN (3 mL). In a second screw top test tube was added EtCN (1 mL) and HOTf (974 mg, 6.49 mmol). In a third screw top test tube charged with a stir pea was made a slurry of tetracycline hydrochloride (453 mg, 0.942 mmol) in EtCN (4 mL). All three solutions were chilled to -30 °C in a cold bath. The solution of HOTf was transferred to the test tube containing **2D** and the resulting mixture was allowed to stir at -30 °C for 5 min, after which the solution of tetracycline hydrochloride was also transferred to this test tube. The reaction was allowed to proceed for 17 h, after which the reaction mixture was removed from the cold bath, diluted with $CHCl_3$ (15 mL), washed with DI $H_2O$ (3 x 5 mL), and dried over anhydrous $MgSO_4$. The $MgSO_4$ was filtered with a medium porosity fritted funnel and the filtrate concentrated *in vacuo* to ~3 mL. The concentrated filtrate was transferred to stirring $Et_2O$ (300 mL), providing a golden-yellow precipitate which was collected on a 30 mL fine porosity fritted funnel, washed with $Et_2O$ (30 mL), and desiccated under static vacuum overnight to provide *(WR,4S,5a'S)*-**21D** and *(WS,4R,5a'S)*-**21D** as a 1:1 mixture of diastereomers [513 mg, 82 % (mass recovery)]. NMR data show free anhydrotetracycline present as an impurity in an ~1.00:1.25 ratio (**21D**:anhydrotetracycline) based on integrations. Attempts to remove the free anhydrotetracycline with both a Florisil® column and a water-deactivated basic alumina column caused decomposition of the complex. An attempt to remove the free anhydrotetracycline with a C18 stationary phase column failed to provide adequate separation. An attempt to separate the complex from the free anhydrotetracycline *via* size-exclusion chromatography (GPC column)

was unsuccessful. Attempts to remove the free anhydrotetracycline by an extraction between chloroform and 10% aqueous AcOH improved the ratio to ~1.00:0.55 ratio (**21D**:anhydrotetracycline), but more extractions with 10% aqueous AcOH failed to improve the ratio further, and other concentrations of aqueous AcOH (30% and 50%) did not improve on this result. Lastly, various attempts at recrystallization did not prove successful.

**$^1$H NMR (800 MHz, DMSO-*d*$_6$) δ:** 9.59 (br s, 1H)*, 9.25 (br s, 3H)*, 8.35 (d, *J* = 1.9 Hz, 2H), 8.28 (d, *J* = 2.1 Hz, 2H), 8.19 (d, *J* = 2.2 Hz, 2H), 8.18 (d, *J* = 2.3 Hz, 2H), 8.15 (d, *J* = 1.9 Hz, 1H), 8.15 (d, *J* = 1.9 Hz, 1H), 8.14 (d, *J* = 2.0 Hz, 1H), 8.05 (d, *J* = 8.8 Hz, 2H), 7.64 (m, 2H), 7.54 (d, *J* = 1.8 Hz, 2H), 6.57 (m, 2H), 6.55 (t, *J* = 2.0 Hz, 2H), 6.48 (m, 2H), 5.01 (t, *J* = 7.7 Hz, 2H), 4.31 (br s, 1H)*, 4.18 (dd, *J* = 7.9, 15.1 Hz, 2H), 3.50 (br s, 2H), 3.39 (m, 2H), 3.13 (s, 3H)**, 3.09 (m, 4H), 2.86 (br s, 12H)*, 2.80 (m, 2H)*, ~2.5 (buried)***, 2.45 (s, 3H), 2.44 (s, 3H), 2.15 (m, 2H), 1.71 (m, 2H), 1.08 (m, 18H). **$^{13}$C NMR (201 MHz, DMSO-*d*$_6$) δ:** 194.2, 194.1, 192.9 (very br), 187.3 (very br), 172.2, 163.7, 153.3, 153.3, 145.2, 143.9, 142.4, 139.1, 139.0, 138.7, 138.5, 132.7 (br), 130.6 (br), 130.4, 123.1 (triflate), 122.0, 121.9, 121.5 (triflate), 119.9 (triflate), 118.3 (triflate), 116.0, 116.0, 112.2, 108.7, 108.2, 107.9, 107.7, 97.4 (br.), 76.3, 73.8 ($J_{CP}$ = 14.9 Hz), 73.8 ($J_{CP}$ = 14.7 Hz), 67.0 (br.), 65.8, 57.4, 42.7 (very br), 41.4 (very br), 34.3, 31.9, 28.6, 14.0, 13.9, 12.9 ($J_{CP}$ = 31.3 Hz), 12.8 ($J_{CP}$ = 31.3 Hz).

***Note:** The signals at 9.59, 9.25, 4.31, 2.86, and 2.80 ppm overlap with a free anhydrotetracycline impurity.

****Note:** The signal at 3.13 ppm overlaps with a MeOH solvent impurity.

*****Note:** HSQC data indicate that the proton signal associated with the carbon at 121.9 ppm is buried under the DMSO-*d*$_6$ solvent peak.

**General Procedure 5:** To a 30 mL test tube were added **4P** or **4D** and MeOH (5 mL). This solution was chilled to -30 °C for 5 min before adding $NaBH_4$. The reaction mixture was allowed to stir at -30 °C for 30 min before diluting with DCM (5 mL) and washing with 10 mL of DI $H_2O$ (2 x 5 mL). The organic layer was dried over anhydrous $Na_2SO_4$ and concentrated *in vacuo*. A solution of HOTf in DME was added to the resulting oil to give a dark red solution. This solution was then added to stirring $Et_2O$ (300 mL) to produce a precipitate which was collected on a 30 mL medium porosity fritted funnel, washed with $Et_2O$ (30 mL).

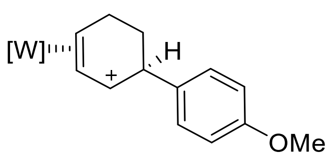


**27P**

Used **General Procedure 5** with **7P** (0.2838 g, 0.3266 mmol), $NaBH_4$ (0.0893 g, 2.36 mmol), and HOTf (0.1138 g, 0.7583 mmol) in DME (1 mL). Tan solid (0.1321 g, 48%).

**$^1$H NMR (600 MHz, $(CD_3)_2CO$) δ:** 8.63 (d, *J* = 2.2 Hz, 1H), 8.34 (d, *J* = 2.2 Hz, 1H), 8.21 (d, *J* = 2.3 Hz, 1H), 8.17 (d, *J* = 2.4 Hz, 1H), 8.01 (d, *J* = 2.2 Hz, 1H), 7.94 (d, *J* = 2.4 Hz, 1H), 7.40 (d, *J* = 8.7 Hz, 2H), 6.99 (d, *J* = 8.7 Hz, 2H), 6.65 (m, 2H), 6.32 (t, *J* = 2.4 Hz, 1H), 6.29 (d, *J* = 8.7

Hz, 1H), 5.54 (t, $J$ = 7.4 Hz, 1H), 4.93 (m, 1H), 4.53 (m, 1H), 3.82 (s, 3H), 3.34 (ddd, $J$ = 13.4, 9.9, 4.1 Hz, 1H), 2.66 (m, 1H), 1.86 (m, 1H), 1.39 (m, 10H).

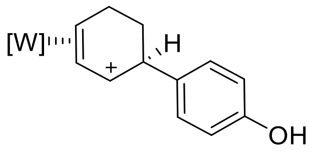


**28P**

Used **General Procedure 5** with **6P** (0.2651 g, 0.3100 mmol), $NaBH_4$ (0.0914 g, 2.42 mmol), and HOTf (0.1075 g, 0.7163 mmol) in DME (1 mL). Tan solid (0.1629 g, 64%).

**$^1$H NMR (600 MHz, $(CD_3)_2CO$) δ:** 8.63 (d, $J$ = 2.3 Hz, 1H), 8.34 (d, $J$ = 2.4 Hz, 1H), 8.21 (d, $J$ = 2.5 Hz, 1H), 8.17 (d, $J$ = 2.5 Hz, 1H), 8.02 (d, $J$ = 2.3 Hz, 1H), 7.94 (d, $J$ = 2.5 Hz, 1H), 7.29 (d, $J$ = 8.5 Hz, 2H), 6.90 (d, $J$ = 8.5 Hz, 2H), 6.66 (t, $J$ = 2.4 Hz, 1H), 6.65 (t, $J$ = 2.3 Hz, 1H), 6.32 (m, 2H), 5.51 (t, $J$ = 7.2 Hz, 1H), 4.89 (dt, $J$ = 16.0, 7.8 Hz, 1H), 4.49 (t, $J$ = 8.2 Hz, 1H), 3.34 (m, 1H), 2.65 (m, 1H), 1.85 (m, 1H), 1.39 (m, 10H). **$^{13}$C NMR (201 MHz, $CD_2Cl_2$) δ:** 156.7, 148.5, 145.3, 142.2, 138.8, 138.7, 138.6, 136.9, 135.8, 129.1, 116.5, 108.9, 108.9, 107.6, 104.0 (d, $J_{CP}$ = 3.3 Hz), 70.2 (d, $J_{CP}$ = 12.6 Hz), 42.4, 30.0, 25.9, 13.5 (d, $J_{CP}$ = 32.7 Hz). Composition confirmed by single crystal X-ray diffraction.

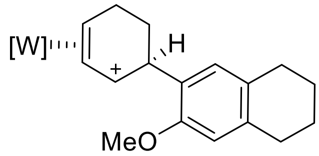


**30P**

Used **General Procedure 5** with **5P** (0.4409 g, 0.4775 mmol), $NaBH_4$ (0.1869 g, 4.941 mmol), and HOTf (0.1364 g, 0.9088 mmol) in DME (2 mL). Tan solid (0.2487 g, 58%).

**$^1$H NMR (800 MHz, $CD_3CN$) δ:** 8.42 (d, $J$ = 2.2 Hz, 1H), 8.13 (d, $J$ = 2.3 Hz, 1H), 8.00 (d, $J$ = 2.4 Hz, 1H), 7.95 (m, 2H), 7.77 (d, $J$ = 2.4 Hz, 1H), 7.01 (s, 1H), 6.77 (s, 1H), 6.54 (t, $J$ = 2.3 Hz, 1H), 6.53 (t, $J$ = 2.3 Hz, 1H), 6.27 (t, $J$ = 2.3 Hz, 1H), 6.22 (d, $J$ = 7.6 Hz, 1H), 5.12 (t, $J$ = 7.3 Hz, 1H), 4.87 (m, 1H), 4.50 (dt, $J$ = 15.6, 7.4 Hz, 1H), 3.86 (s, 3H), 3.28 (m, 1H), 2.77 (br m, 2H), 2.68 (m, 2H), 2.56 (m, 1H), 1.76 – 1.65 (m, 5H), 1.54 (m, 1H), 1.21 (d, $J$ = 9.8 Hz, 9H). **$^{13}$C NMR (201 MHz, $CD_3CN$) δ:** 155.5, 148.8, 146.2, 143.2, 139.7, 139.5, 139.5, 139.4, 138.0, 131.3, 130.3, 129.2, 112.7, 109.4, 109.0, 108.1, 103.2, 70.4 (d, $J_{CP}$ = 11.6 Hz), 56.3, 36.3, 30.0, 29.2, 27.1, 26.4, 24.1, 23.9, 13.3 (d, $J_{CP}$ = 32.7 Hz). Composition confirmed by single crystal X-ray diffraction.

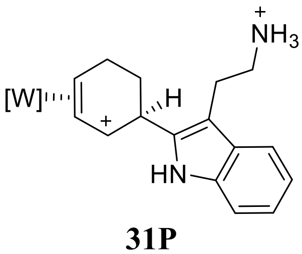


**31P**

Used **General Procedure 5** with **14P** (0.3691 g, 0.3445 mmol), $NaBH_4$ (0.089 g, 2.35 mmol), and HOTf (0.1034 g, 0.6890 mmol) in DME (2 mL). Tan solid (0.2606 g, 73%).

**$^1$H NMR (800 MHz, $CD_3CN$) δ:** 9.62 (s, 1H), 8.47 (d, $J$ = 1.9 Hz, 1H), 8.02 (d, $J$ = 2.2 Hz, 1H), 8.01 (d, $J$ = 2.0 Hz, 1H), 7.97 (d, $J$ = 2.1 Hz, 1H), 7.82 (d, $J$ = 2.1 Hz, 1H), 7.77 (m, 1H), 7.62 (d, $J$ = 7.9 Hz, 1H), 7.40 (d, $J$ = 8.1 Hz, 1H), 7.17 (t, $J$ = 7.6 Hz, 1H), 7.11 (t, $J$ = 7.5 Hz, 1H), 6.56 (t, $J$ = 2.3 Hz, 1H), 6.55 (t, $J$ = 2.3 Hz, 1H), 6.22 (t, $J$ = 2.3 Hz, 1H), 6.13 (d, $J$ = 7.4 Hz, 1H), 5.45 (t, $J$ = 7.3 Hz, 1H), 4.81 (m, 1H), 4.74 (dt, $J$ = 15.4, 7.5 Hz, 1H), 3.27 (m, 5H), 2.58 (ddd, $J$ = 16.7, 11.4, 5.9 Hz, 1H), 1.87 (dt, $J$ = 12.7, 6.1 Hz, 1H), 1.55 (m, 1H), 1.23 (d, $J$ = 9.8 Hz, 9H). **$^{13}$C NMR (201 MHz, $CD_3CN$) δ:** 149.0, 146.4, 143.4, 139.6, 139.6, 139.5, 137.3, 129.2, 129.1, 128.8, 122.9, 120.5, 119.1, 112.1, 109.5, 109.1, 108.3, 106.4, 106.1 (d, $J_{CP}$ = 3.4 Hz), 72.8 (d, $J_{CP}$ = 11.9 Hz), 41.8, 34.9, 28.3, 25.9, 22.9, 13.3 (d, $J_{CP}$ = 33.3 Hz). Composition confirmed by single crystal X-ray diffraction.

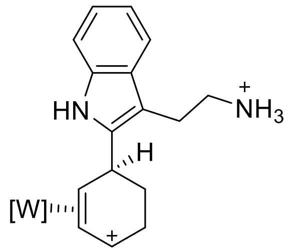


**31D**

Used **General Procedure 5** with **14D** (0.8523 g, 0.7955 mmol), $NaBH_4$ (0.1368 g, 3.616 mmol), and HOTf (0.3646 g, 2.429 mmol) in DME (2 mL). Tan solid (0.6444 g, 78%).

**$^1$H NMR (800 MHz, $CD_3CN$) δ:** 9.72 (s, 1H), 8.43 (d, $J$ = 2.3 Hz, 1H), 8.17 (d, $J$ = 2.3 Hz, 1H), 8.02 (d, $J$ = 2.3 Hz, 1H), 8.00 (d, $J$ = 2.4 Hz, 1H), 7.97 (d, $J$ = 2.4 Hz, 1H), 7.84 (d, $J$ = 2.6 Hz, 1H), 7.58 (d, $J$ = 7.9 Hz, 1H), 7.48 (m, 1H), 7.18 (ddd, $J$ = 8.1, 7.1, 1.1 Hz, 1H), 7.11 (ddd, $J$ = 8.0, 7.1, 1.0 Hz, 1H), 6.69 (t, $J$ = 7.1 Hz, 1H), 6.54 (t, $J$ = 2.3 Hz, 1H), 6.52 (t, $J$ = 2.3 Hz, 1H), 6.37 (t, $J$ = 2.4 Hz, 1H), 5.47 (t, $J$ = 7.4 Hz, 1H), 4.37 (ddt, $J$ = 15.7, 7.2, 1.8 Hz, 1H), 4.06 (m, 1H), 3.54 (m, 1H), 3.37 (m, 1H), 3.24 (m, 2H), 3.17 (m, 1H), 2.97 (m, 1H), 1.85 (dtd, $J$ = 13.0, 6.3, 1.9 Hz, 1H), 1.49 (dtd, $J$ = 13.3, 10.9, 6.1 Hz, 1H), 1.02 (d, $J$ = 9.8 Hz, 9H). **$^{13}$C NMR (201 MHz, $CD_3CN$) δ:** 149.3, 146.2, 143.2, 142.5, 139.6, 139.6, 139.6, 137.2, 137.1, 128.4, 122.8, 120.4, 119.0, 112.3, 109.6, 109.1, 108.1, 105.5, 105.1 (d, $J_{CP}$ = 3.4 Hz), 71.5 (d, $J_{CP}$ = 13.1 Hz), 41.5, 36.2, 29.6, 25.7, 23.1, 13.5 (d, $J_{CP}$ = 33.0 Hz). **APCI-HRMS (m/z):** $[M]^+$ calculated for $C_{28}H_{39}BN_9OPW$ 743.2612; found, 743.2583.

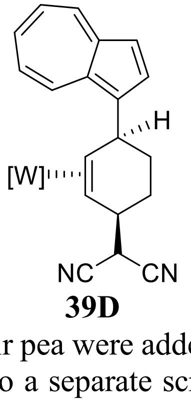


**39D**

To a screw top test tube charged with a stir pea were added THF (~2 mL) and **79D** (349 mg, 0.407 mmol), yielding a green/blue solution. To a separate screw top test tube charged with a stir pea were added malononitrile (67 mg, 1.0 mmol) and THF (~1 mL). Both solutions were chilled to -40 °C in a cold-bath. KOtBu (0.30 mL, 20% w/w in THF, 0.48 mmol) was added to the

malononitrile solution. The resultant solution was allowed to stir at -40 °C for 30 s, after which this solution was transferred to the THF/**79D** solution. The reaction was allowed to stir at -40 °C for 18 h, after which the reaction mixture was removed from the cold-bath, diluted with DCM (~5 mL), and washed with DI $H_2O$ (3 x ~3 mL). The reaction mixture was dried with anhydrous $Na_2SO_4$, which was then filtered with a 30 mL medium porosity fritted funnel. The filtrate was concentrated *in vacuo* to leave a green/blue oil, which was redissolved in minimal DCM and added to stirring chilled pentane (-20 °C, ~50 mL), forming a light-blue precipitate which was collected on a 15 mL fine porosity fritted funnel. The precipitate was washed with chilled (-20 °C) pentane (~15 mL) and then desiccated under static vacuum overnight, yielding **39D** as the major product [246 mg, 78% (mass recovery)].

NMR data show two impurities with approximately 12% and 15% the integration of the major species characterized by two sets of $PMe_3$ and Tp signals that we tentatively ascribe to binuclear complexes in which two tungsten complexes coupled to one azulene moiety.

**$^1$H NMR (600 MHz, $CD_3CN$) δ:** 8.51 (d, $J$ = 3.9 Hz, 1H), 8.49 (d, $J$ = 9.8 Hz, 1H), 8.36 (d, $J$ = 9.5 Hz, 1H), 8.09 (d, $J$ = 2.1 Hz, 1H), 8.08 (d, $J$ = 2.0 Hz, 1H), 7.86 (m, 3H), 7.62 (t, $J$ = 9.8 Hz, 1H), 7.47 (d, $J$ = 3.8 Hz, 1H), 7.38 (d, $J$ = 2.2 Hz, 1H), 7.17 (t, $J$ = 9.7 Hz, 1H), 7.14 (t, $J$ = 9.7 Hz, 1H), 6.39 (t, $J$ = 2.2 Hz, 1H), 6.36 (t, $J$ = 2.3 Hz, 1H), 6.26 (t, $J$ = 2.2 Hz, 1H), 4.94 (t, $J$ = 5.1 Hz, 1H), 4.01 (d, $J$ = 5.6 Hz, 1H), 3.71 (m, 1H), 3.06 (t, $J$ = 12.2 Hz, 1H), 2.34 (m, 1H), 1.89 (m, 1H), 1.60 (m, 2H), 1.13 (m, 1H), 0.94 (d, $J$ = 8.3 Hz, 9H). **$^{13}$C NMR (201 MHz, $CD_3CN$) δ:** 144.3, 142.1, 142.0, 141.8, 139.0, 138.6, 138.1, 138.0, 137.6, 137.5, 134.9, 133.7, 123.3, 122.7, 117.7, 115.0, 115.0, 107.7, 107.5, 107.4, 58.7 (d, $J_{CP}$ = 10.9 Hz), 53.1, 41.9, 38.3 (d, $J_{CP}$ = 3.3 Hz), 34.4, 30.2, 25.0, 13.6 (d, $J_{CP}$ = 27.4 Hz). **CV (MeCN, 50 mV/s):** $E_{p,a}$ = +0.57 V (NHE). Composition confirmed by single crystal X-ray diffraction.

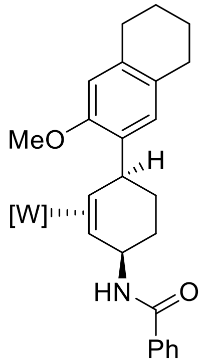


**40D**

To a 15 mL test tube were added **30D** (0.3309 g, 0.3704 mmol) and THF (5 mL). This solution was chilled to -60 °C. To a separate 15 mL test tube were added benzamide (0.1121 g, 0.9254 mmol) and THF (5 mL). A solution of *n*-BuLi (0.6 mL, 1.6 M in hexanes, 1 mmol) was then added dropwise to the test tube containing benzamide. This solution was allowed to stir at room temperature for 5 min before chilling to -60 °C for 5 min and adding to the test tube containing **30D.** After 18 h at -60 °C, the reaction mix was diluted with DCM (~5 mL) and washed with 20 mL of a saturated aqueous solution of $Na_2CO_3$ (2 x 10 mL). The organic layer was dried over anhydrous $Na_2SO_4$ and evaporated to an oil. The film was dissolved in minimal DCM and added to 150 mL of stirring pentane. A tan-white solid collected on 15 mL fine porosity fritted funnel and washed with 30 mL of pentane (2 x 15 mL) to yield **40D** (0.2218 g, 69%).

**¹H NMR (800 MHz, $CD_3CN$) δ:** 8.29 (d, *J* = 2.1 Hz, 1H), 8.09 (d, *J* = 2.0 Hz, 1H), 7.86 (d, *J* = 2.4 Hz, 1H), 7.82 (d, *J* = 2.2 Hz, 1H), 7.71 (m, 3H), 7.68 (br s, 1H), 7.47 (t, *J* = 7.3 Hz, 1H), 7.40 (t, *J* = 7.7 Hz, 2H), 7.37 (d, *J* = 2.2 Hz, 1H), 7.04 (d, *J* = 8.5 Hz, 1H), 6.67 (s, 1H), 6.40 (t, *J* = 2.2 Hz, 1H), 6.22 (t, *J* = 2.3 Hz, 1H), 6.08 (t, *J* = 2.3 Hz, 1H), 5.48 (tt, *J* = 8.5, 3.3 Hz, 1H), 4.61 (dd, *J* = 6.4, 3.6 Hz, 1H), 3.82 (s, 3H), 2.79 (m, 4H), 2.67 (t, *J* = 11.9 Hz, 1H), 2.09 (m, 1H), 1.80 (m, 5H), 1.38 (m, 3H), 1.01 (d, *J* = 8.3 Hz, 9H). **¹³C NMR (201 MHz, $CD_3CN$) δ:** 166.8, 155.2, 144.2, 143.2, 141.7, 139.2, 137.9, 137.2, 137.2, 136.8, 135.6, 131.8, 131.2, 129.3, 129.2, 127.9, 111.7, 107.6, 107.0, 106.4, 58.6 (d, $J_{CP}$ = 10.4 Hz), 56.7, 56.2, 53.2, 37.5, 30.0, 29.6, 28.4, 28.1, 24.5, 24.2, 13.4 (d, $J_{CP}$ = 27.4 Hz). **APCI-HRMS (m/z):** $[M+H]^+$ calculated for $C_{36}H_{46}BN_8O_3PW$ 865.3106; found, 865.3100.

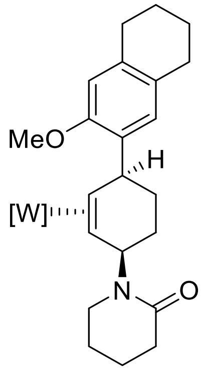


**41D**

To a 15 mL test tube were added **30D** (0.3503 g, 0.3921 mmol) and THF (5 mL). This solution was chilled to -60 °C. To a separate 15 mL test tube were added 2-piperidone (0.5150 g, 5.195 mmol) and THF (5 mL). A solution of *n*-BuLi (0.7 mL, 1.6 M in hexanes, 1 mmol) was then added dropwise to the test tube containing 2-piperidone. This solution was allowed to stir at room temperature for 5 min before chilling to -60 °C for 5 min and adding to the test tube containing **30D.** After 18 h at -60 °C, the reaction mix was diluted with DCM (~5 mL) and washed with 20 mL of a saturated aqueous solution of $Na_2CO_3$ (2 x 10 mL). The organic layer was dried over anhydrous $Na_2SO_4$ and evaporated to an oil. The film was dissolved in minimal DCM and added to 150 mL of stirring pentane. A tan-white solid collected on 15 mL fine porosity fritted funnel and washed with 30 mL of pentane (2 x 15 mL) to yield **41D** (0.2028 g, 61%).

**¹H NMR (800 MHz, $CD_2Cl_2$) δ:** 8.49 (d, *J* = 2.1 Hz, 1H), 8.12 (d, *J* = 2.0 Hz, 1H), 7.76 (d, *J* = 2.3 Hz, 1H), 7.73 (d, *J* = 2.2 Hz, 1H), 7.63 (d, *J* = 2.4 Hz, 1H), 7.56 (s, 1H), 7.33 (d, *J* = 2.2 Hz, 1H), 6.61 (s, 1H), 6.36 (t, *J* = 2.2 Hz, 1H), 6.28 (m, 1H), 6.17 (t, *J* = 2.2 Hz, 1H), 6.16 (t, *J* = 2.3 Hz, 1H), 4.58 (dd, *J* = 6.1, 2.3 Hz, 1H), 3.82 (s, 3H), 3.60 (ddd, *J* = 11.4, 6.8, 4.2 Hz, 1H), 3.38 (ddd, *J* = 12.0, 7.5, 4.2 Hz, 1H), 2.77 – 2.70 (m, 4H), 2.62 (t, *J* = 11.6 Hz, 1H), 2.40 – 2.27 (m, 3H), 1.87 – 1.67 (m, 6H), 1.63 (m, 2H), 1.50 – 1.30 (m, 4H), 1.02 (d, *J* = 8.1 Hz, 9H). **¹³C NMR (201 MHz, $CD_2Cl_2$) δ:** 169.4, 154.9, 144.0, 143.3, 140.9, 138.7, 137.1, 136.1, 136.1, 135.2, 130.7, 128.4, 111.0, 107.0, 106.1, 105.9, 59.5 (d, $J_{CP}$ = 10.3 Hz), 56.0, 55.6, 53.3, 42.2, 37.2, 33.3, 30.0, 29.5, 26.7, 24.3, 24.3, 24.0, 23.2, 21.8, 13.3 (d, $J_{CP}$ = 27.6 Hz). Composition confirmed by single crystal X-ray diffraction.

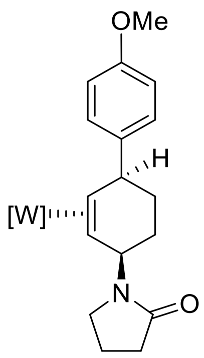


**42D**

To a screw top test tube were added **27D** (0.225 g, 0.268 mmol) and THF (~5 mL). To a separate screw top test tube equipped with a stir pea were added 2-pyrrolidone (0.091 mg, 1.1 mmol) and *n*-BuLi (0.503 mL, 1.6 M in hexanes, 0.8 mmol). Both test tubes were allowed to chill to -60 ºC before adding the test tube containing 2-pyrrolidone and *n*-BuLi to the test tube containing **27D**. This solution was allowed to stir for 20 h at -60 ºC. After stirring, the reaction mixture was diluted with DCM (~5 mL) and washed with 15 mL of a saturated aqueous solution of $Na_2CO_3$ (3 x 5 mL). The organic layer was dried over anhydrous $Na_2SO_4$ and evaporated to a film. The film was dissolved in minimal DCM and added to 150 mL of stirring pentane. A dark-grey solid was collected on a 15 mL fine porosity fritted funnel and washed with 20 mL of pentane (2 x 10 mL) to give **42D** (0.107 g, 52%).

**$^{1}$H NMR (800 MHz, $CD_3CN$) δ:** 8.44 (d, *J* = 2.2 Hz, 1H), 8.11 (d, *J* = 2.0 Hz, 1H), 7.87 (dt, *J* = 2.4, 0.6 Hz, 1H), 7.82 (d, *J* = 2.3 Hz, 1H), 7.74 – 7.73 (m, 1H), 7.60 – 7.57 (m, 2H), 7.37 (d, *J* = 2.3 Hz, 1H), 6.97 – 6.94 (m, 2H), 6.41 (td, *J* = 2.1, 0.8 Hz, 1H), 6.22 (td, *J* = 2.2, 0.7 Hz, 1H), 6.17 (t, *J* = 2.3 Hz, 1H), 5.71 – 5.63 (m, 1H), 4.12 (t, *J* = 4.1 Hz, 1H), 3.80 (s, 3H), 3.56 – 3.51 (m, 1H), 3.51 – 3.46 (m, 1H), 2.69 (t, *J* = 11.5 Hz, 1H), 2.36 – 2.29 (m, 1H), 2.23 – 2.20 (m, 2H), 1.93 – 1.86 (m, 1H), 1.78 – 1.72 (m, 1H), 1.50 – 1.47 (m, 1H), 1.47 – 1.44 (m, 1H), 1.39 (dt, *J* = 11.4, 2.3 Hz, 1H), 1.28 – 1.23 (m, 2H), 0.99 (d, *J* = 8.4 Hz, 7H). **$^{13}$C NMR (201 MHz, $CD_3CN$) δ:** 173.4, 157.7, 144.9, 143.1, 143.0, 141.0, 137.1, 136.2, 136.2, 129.5, 113.3, 106.7, 106.0, 105.5, 58.0 (d, $J_{CP}$ = 10.6 Hz), 54.9, 53.0, 51.6, 44.6 (d, $J_{CP}$ = 4.6 Hz), 42.6, 31.6, 29.1, 22.1, 18.1, 12.2 (d, $J_{CP}$ = 28.2 Hz). **CV (MeCN, 100 mV/s):** $E_{p,a}$ = +0.53 V (NHE). **APCI-HRMS (m/z):** $[M]^+$ calculated for $C_{32}H_{42}BN_8O_4PSW$ 775.2643; found 775.2599.

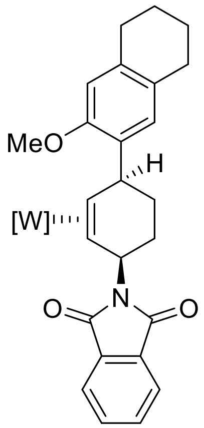


**43D**

To a 15 mL test tube were added **30D** (0.2903 g, 0.3249 mmol) and THF (5 mL). To a separate 15 mL test tube were added phthalimide (0.1580 g, 1.074 mmol), KOtBu (0.4481 g, 20% w/w in THF, 7987 mmol) and THF (5 mL). This solution was allowed to stir at room temperature for 5 min before chilling to -60 °C for 5 min and adding to the test tube containing **30D**. After 18 h at -60 °C, the reaction mix was evaporated to an oil. The film was dissolved in minimal DCM and added to 150 mL of stirring pentane. A tan-white solid collected on 15 mL fine porosity fritted funnel and washed with 30 mL of pentane (2 x 15 mL) to yield **43D** (0.1416 g, 49%).

**$^1$H NMR (800 MHz, $CD_2Cl_2$) δ:** 8.45 (d, *J* = 2.3 Hz, 1H), 8.30 (s, 1H), 8.10 (d, *J* = 1.5 Hz, 1H), 7.76 (dd, *J* = 5.4, 3.0 Hz, 2H), 7.74 (d, *J* = 2.3 Hz, 1H), 7.67 (d, *J* = 2.2 Hz, 1H), 7.64 (dd, *J* = 5.4, 3.0 Hz, 2H), 7.56 (d, *J* = 2.4 Hz, 1H), 7.33 (d, *J* = 2.2 Hz, 1H), 6.61 (s, 1H), 6.35 (t, *J* = 2.2 Hz, 1H), 6.15 (t, *J* = 2.2 Hz, 1H), 6.08 (t, *J* = 2.3 Hz, 1H), 5.92 (m, 1H), 4.70 (d, *J* = 5.8 Hz, 1H), 3.77 (s, 3H), 2.95 (m, 2H), 2.80 (m, 2H), 2.76 (t, *J* = 12.1 Hz, 1H), 2.30 (td, *J* = 13.5, 13.0, 5.6 Hz, 1H), 2.04 (m, 1H), 1.91 – 1.74 (m, 5H), 1.44 (m, 2H), 1.05 (d, *J* = 8.1 Hz, 9H). **$^{13}$C NMR (201 MHz, $CD_2Cl_2$) δ:** 168.1, 154.4, 143.2, 142.9, 140.7, 138.6, 136.9, 136.1, 136.1, 134.9, 133.1, 132.9, 131.7, 130.2, 122.9, 110.4, 106.8, 106.4, 105.8, 59.1 (d, $J_{CP}$ = 10.1 Hz), 55.9, 55.6, 54.3, 36.8, 29.9, 29.3, 27.1, 24.8, 24.2, 24.0, 13.3 (d, $J_{CP}$ = 27.0 Hz). Composition confirmed by single crystal X-ray diffraction.

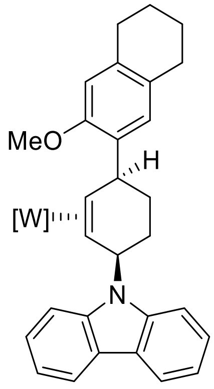


**44D**

To a 15 mL test tube were added **30D** (0.4004 g, 0.4482 mmol) and THF (5 mL). This solution was chilled to -60 °C. To a separate 15 mL test tube were added carbazole (0.4412 g, 2.639 mmol) and THF (5 mL). A solution of *n*-BuLi (0.65 mL, 1.6 M in hexanes, 1.0 mmol) was then added dropwise to the test tube containing carbazole. This solution was allowed to stir at room temperature for 5 min before chilling to -60 °C for 5 min and adding to the test tube containing **30D.** After 18 h at -60 °C, the reaction mix was diluted with DCM (~5 mL) and washed with 20 mL of a saturated aqueous solution of $Na_2CO_3$ (2 x 10 mL). The organic layer was dried over anhydrous $Na_2SO_4$ and evaporated to an oil. The film was dissolved in minimal DCM and added to 150 mL of stirring pentane. A tan-white solid collected on 15 mL fine porosity fritted funnel and washed with 30 mL of pentane (2 x 15 mL) to yield **44D** (0.2205 g, 54%).

**$^1$H NMR (800 MHz, $CD_2Cl_2$) δ:** 8.26 (br s, 1H), 8.18 (d, *J* = 2.1 Hz, 1H), 8.17 (d, *J* = 2.1 Hz, 1H), 8.07 (d, *J* = 7.8 Hz, 2H), 8.03 (s, 1H), 7.74 (d, *J* = 2.3 Hz, 1H), 7.63 (d, *J* = 2.3 Hz, 1H), 7.46 (m, 3H)*, 7.45 (d, *J* = 2.3 Hz, 1H), 7.40 (d, *J* = 2.5 Hz, 1H), 7.18 (t, *J* = 7.4 Hz, 2H), 6.65 (s, 1H), 6.57 (dt, *J* = 11.1, 4.2 Hz, 1H), 6.37 (t, *J* = 2.2 Hz, 1H), 6.15 (t, *J* = 2.3 Hz, 1H), 5.52 (t, *J* = 2.3 Hz, 1H), 4.87 (dd, *J* = 6.2, 2.3 Hz, 1H), 3.86 (s, 3H), 3.03 – 2.75 (m, 5H), 2.53 (dt, *J* = 11.3, 2.6 Hz, 1H), 2.43 (m, 1H), 2.04 (m, 1H), 1.85 (m, 4H), 1.62 (m, 2H), 1.07 (d, *J* = 8.1 Hz, 9H). **$^{13}$C**

**NMR (201 MHz, $CD_2Cl_2$) δ:** 154.7, 143.3, 143.0, 140.8, 140.0, 138.5, 137.0, 136.1, 136.0, 135.3, 130.0, 128.6, 126.2, 123.6, 120.4, 118.6, 111.0, 106.9, 106.1, 105.1, 59.2, 59.0 (d, $J_{CP}$ = 10.3 Hz), 55.9, 54.8, 37.3, 37.3, 29.8, 29.4, 28.1, 26.0, 24.1, 23.9, 13.3 (d, $J_{CP}$ = 27.8 Hz). Composition confirmed by single crystal X-ray diffraction.

***Note:** The signal at 7.46 ppm overlaps with a free carbazole impurity.

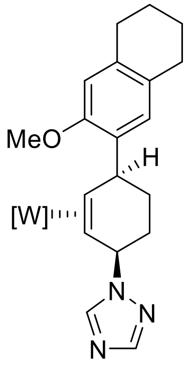


**45D**

To a 15 mL test tube were added **30D** (0.4163 g, 0.4660 mmol) and THF (5 mL). This solution was chilled to -60 °C. To a separate 15 mL test tube were added 1,2,4-triazole (0.7704 g, 11.16 mmol), KOtBu (0.9690 g, 20% w/w in THF, 1.727 mmol) and THF (5 mL). This solution was allowed to stir at room temperature for 5 min before chilling to -60 °C for 5 min and adding to the test tube containing **30D**. After 18 h at -60 °C, the reaction mix was diluted with DCM (~5 mL) and washed with 20 mL of a saturated aqueous solution of $Na_2CO_3$ (2 x 10 mL). The organic layer was dried over anhydrous $Na_2SO_4$ and evaporated to an oil. The film was dissolved in minimal DCM and added to 150 mL of stirring pentane. A tan-white solid collected on 15 mL fine porosity fritted funnel and washed with 30 mL of pentane (2 x 15 mL) to yield **45D** (0.2161 g, 57%).

**$^1$H NMR (800 MHz, $CD_2Cl_2$) δ:** 8.61 (s, 1H), 8.10 (d, $J$ = 2.0 Hz, 1H), 8.09 (d, $J$ = 2.0 Hz, 1H), 7.82 (s, 1H), 7.76 (d, $J$ = 2.4 Hz, 1H), 7.72 (d, $J$ = 2.3 Hz, 1H), 7.67 (d, $J$ = 2.5 Hz, 1H), 7.45 (s, 1H), 7.28 (d, $J$ = 2.2 Hz, 1H), 6.61 (s, 1H), 6.35 (t, $J$ = 2.3 Hz, 1H), 6.23 (t, $J$ = 2.3 Hz, 1H), 6.20 (t, $J$ = 2.2 Hz, 1H), 5.74 (m, 1H), 4.61 (m, 1H), 3.77 (s, 3H), 2.98 (t, $J$ = 12.4 Hz, 1H), 2.78 (m, 4H), 2.24 (m, 1H), 1.92 (m, 1H), 1.80 (m, 5H), 1.50 (d, $J$ = 11.2 Hz, 1H), 1.26 (m, 1H), 0.99 (d, $J$ = 8.3 Hz, 9H). **$^{13}$C NMR (201 MHz, $CD_2Cl_2$) δ:** 154.7, 151.1, 143.4, 142.8, 142.7, 140.5, 137.6, 136.9, 136.7, 136.5, 135.5, 130.0, 129.4, 111.1, 106.9, 106.4, 106.4, 62.8, 55.6, 55.6 (d, $J_{CP}$ = 16.7 Hz), 53.9, 29.9, 29.3, 28.3, 27.9, 24.2, 24.1, 23.8, 13.9 (d, $J_{CP}$ = 28.4 Hz). Composition confirmed by single crystal X-ray diffraction.

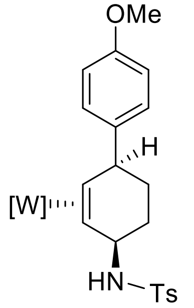


**47D**

To a 30 mL screw top test tube charged with a stir pea were added MeCN (~2 mL) and **27D** (250 mg, 0.298 mmol). To a separate test tube was added p-toluenesulfonamide (56.0 mg, 0.327 mmol) followed by MeCN (~2 mL). To this solution was added DBU (0.044 mL, 0.298 mmol). Both solutions were allowed to chill to -30 °C for 5 min before adding the test tube containing p-toluenesulfonamide and DBU to the test tube containing **27D**. After 1 h at -30 °C, the reaction mix was diluted with DCM (~5 mL) and washed with saturated aqueous $Na_2CO_3$ (3 x ~3 mL). Then the reaction was washed with a 0.1 M aqueous solution of NaOH (3 x ~3 mL). The reaction mixture was dried was over anhydrous $Na_2SO_4$ and evaporated to an oil. The oil was dissolved in minimal DCM and added to 200 mL of stirring pentane. A tan solid was collected on a 15 mL fine porosity fritted funnel and washed with pentane (2 x 20 mL) to yield **47D** (0.111 g, 43%).

**$^1$H NMR (800 MHz, $CD_3CN$) δ:** 7.97 (d, $J$ = 2.1 Hz, 1H), 7.82 (d, $J$ = 0.7 Hz, 1H), 7.81 – 7.78 (m, 3H), 7.49 (d, 2H, $J$ = 8.5 Hz), 7.22 (d, 2H, $J$ = 8.2 Hz), 6.95 (d, $J$ = 2.2 Hz, 1H), 6.93 (d, 2H, $J$ = 8.5 Hz), 6.86 (d, 2H, $J$ = 7.9 Hz), 6.33 – 6.31 (m, 2H), 6.16 (t, $J$ = 2.2 Hz, 1H), 5.68 (d, $J$ = 7.7 Hz, 1H), 4.43 (s, 2H), 3.93 – 3.87 (m, 1H), 3.80 (s, 3H), 2.76 (ddd, $J$ = 14.3, 11.2, 2.8 Hz, 1H), 2.23 (s, 3H), 2.02 (tdd, $J$ = 12.9, 3.8, 2.6 Hz, 1H), 1.61 – 1.56 (m, 2H), 1.55 – 1.50 (m, 1H), 1.42 (tdd, $J$ = 13.2, 11.0, 2.7 Hz, 1H), 0.83 (d, $J$ = 8.4 Hz, 14H), 0.60 (d, $J$ = 11.1 Hz, 2H). **$^{13}$C NMR (201 MHz, $CD_3CN$) δ**: 158.6, 145.6, 144.2, 143.8, 141.0, 137.5, 137.4 137.2, 130.1, 129.9, 127.1, 114.6, 107.4, 107.2, 106.9, 57.1, 55.8, 55.4, 54.8 (d, $J_{CP}$ = 10.6 Hz), 44.6 (d, $J_{CP}$ = 2.7 Hz), 32.2 (d, $J_{CP}$ = 10.2 Hz), 28.9, 21.5, 14.2 (d, $J_{CP}$ = 28.0 Hz). **CV (MeCN, 100 mV/s):** $E_{p,a}$ = +0.516 V (NHE). **APCI-HRMS (m/z):** $[M+H]^+$ calculated for $C_{32}H_{42}BN_8O_4PSW$ 860.2390; found 690.2099 corresponding to $[M-(NHTs)]^+$, $C_{25}H_{34}BN_7O_2PW^+$ (**27D**).

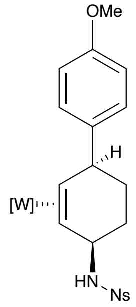


**82D**

To a 20 mL screw top test tube charged with a stir pea were added DCM (~3 mL) and **27D** (25 mg, 0.030 mmol). To a separate test tube was added 4-nitrobenzenesulfonamide (18.0 mg, 0.090 mmol) followed by DCM (~2 mL). To this solution was added DBU (0.008 mL, 0.060 mmol). Both solutions were allowed to chill to -30 °C for 5 min before adding the test tube containing 4-nitrobenzenesulfonamide and DBU to the test tube containing **27D**. After 24 h at -30 °C, the reaction mix was washed with saturated aqueous $Na_2CO_3$ (3 x ~3 mL). Then the reaction was washed with a 0.1 M aqueous solution of NaOH (3 x ~3 mL). The reaction mixture was dried was over anhydrous $Na_2SO_4$ and evaporated to an oil. The oil was dissolved in minimal DCM and added to 50 mL of stirring pentane. A light grey solid was collected on a 15 mL fine porosity fritted funnel and washed with pentane (2 x 5 mL) to yield **82D** (26 mg, 65%).

**$^1$H NMR** (800 MHz, $CD_2Cl_2$) δ: 7.94 (d, $J$ = 2.0 Hz, 1H), 7.87 – 7.85 (m, 2H), 7.72 (d, $J$ = 2.4 Hz, 1H), 7.71 – 7.69 (m, 2H), 7.58 (d, $J$ = 2.3 Hz, 1H), 7.46 – 7.42 (m, 2H), 7.39 – 7.36 (m, 2H), 6.92 – 6.89 (m, 2H), 6.75 (d, $J$ = 2.1 Hz, 1H), 6.44 (t, $J$ = 2.2 Hz, 1H), 6.29 (t, $J$ = 2.2 Hz, 1H), 6.01 (t,

*J* = 2.2 Hz, 1H), 4.48 (s, 1H), 3.93 – 3.89 (m, 1H), 3.81 (s, 3H), 2.66 (ddd, *J* = 14.4, 11.1, 3.0 Hz, 1H), 2.16 (tt, *J* = 13.4, 3.2 Hz, 1H), 1.80 – 1.76 (m, 1H), 1.67 – 1.61 (m, 1H), 1.36 – 1.29 (m, 1H), 0.82 (d, *J* = 8.3 Hz, 9H), 0.25 (d, *J* = 11.1 Hz, 1H). **$^{13}$C NMR** (201 MHz, $CD_2Cl_2$) δ: 158.2, 157.9, 144.5, 143.6, 143.6, 139.7, 136.8, 136.8, 136.6, 129.1, 128.1, 123.9, 114.3, 114.0, 107.0, 106.8, 106.1, 56.7, 55.5, 55.4, 53.4 (d, $J_{CP}$ = 10.6 Hz), 43.7, 32.2, 28.6, 14.4 (d, $J_{CP}$ = 28.3 Hz). **APCI-HRMS (m/z):** $[M+H]^+$ calculated for $C_{31}H_{39}BN_9O_6PSW$ 892.2092; found 690.2118 corresponding to $[M-(NHNs)]^+$,$C_{25}H_{34}BN_7O_2PW^+$ (**27D**).

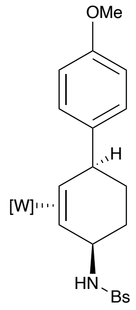


**83D**

To a 20 mL screw top test tube charged with a stir pea were added MeCN (~3 mL) and **27D** (75 mg, 0.089 mmol). To a separate test tube was added 4-bromobenzenesulfonamide (63.0 mg, 0.270 mmol) followed by MeCN (~2 mL). To this solution was added DBU (0.027 mL, 0.180 mmol). Both solutions were allowed to chill to -30 ºC for 5 min before adding the test tube containing 4-bromobenzenesulfonamide and DBU to the test tube containing **27D**. After 24 h at -30 ºC, the reaction mix was diluted with DCM (~5 mL) and washed with saturated aqueous $Na_2CO_3$ (3 x ~3 mL). Then the reaction was washed with a 0.1 M aqueous solution of NaOH (3 x ~3 mL). The reaction mixture was dried was over anhydrous $Na_2SO_4$ and evaporated to an oil. The oil was dissolved in minimal DCM and added to 100 mL of stirring pentane. A dark grey solid was collected on a 15 mL fine porosity fritted funnel and washed with pentane (2 x 10 mL) to yield **83D** (56 mg, 68%).

**$^1$H NMR** (800 MHz, $CD_2Cl_2$) δ: 7.96 (d, *J* = 2.0 Hz, 1H), 7.80 (d, *J* = 2.0 Hz, 1H), 7.78 (dd, *J* = 4.6, 2.4 Hz, 2H), 7.73 (d, *J* = 2.4 Hz, 1H), 7.41 – 7.37 (m, 2H), 7.21 – 7.23 (m, 2H), 7.12 – 7.11 (m, 2H), 6.93 – 6.90 (m, 2H), 6.89 (d, *J* = 2.2 Hz, 1H), 6.34 (t, *J* = 2.2 Hz, 1H), 6.30 (t, *J* = 2.2 Hz, 1H), 6.17 (t, *J* = 2.2 Hz, 1H), 4.92 (s, 1H), 4.43 (s, 1H), 3.96-3.90 (m, 1H), 3.81 (s, 3H), 2.77 – 2.70 (m, 1H), 2.09 (tt, *J* = 13.3, 3.2 Hz, 1H), 1.71-1.67 (m, 1H), 1.65 – 1.61 (m, 1H), 1.41 – 1.33 (m, 1H), 0.86 (d, *J* = 8.3 Hz, 9H), 0.56 (d, *J* = 11.1 Hz, 1H). **$^{13}$C NMR** (201 MHz, $CD_2Cl_2$) δ: 157.5, 143.1, 143.0 (d, *J* = 7.0 Hz), 140.5, 139.5, 136.6, 136.2, 136.1, 132.3, 131.6, 128.7, 128.1, 128.0, 113.9, 106.4, 106.4, 106.0, 56.6, 55.2 (d, $J_{CP}$ = 12.9 Hz), 54.7 (d, $J_{CP}$ = 18.3 Hz), 43.4, 31.6, 27.9, 13.9 (d, $J_{CP}$ = 27.5 Hz). **APCI-HRMS (m/z):** $[M+H]^+$ calculated for $C_{31}H_{39}BBrN_8O_4PSW$ 925.1200; found 690.2118 corresponding to $[M-(NHBs)]^+$, $C_{25}H_{34}BN_7O_2PW^+$ (**27D**).

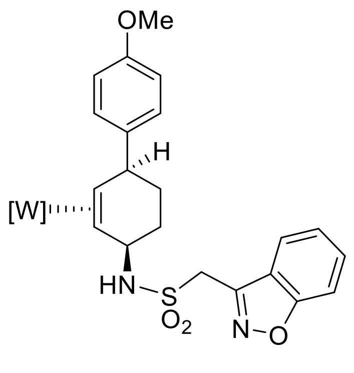

**48D**

To a screw top test tube charged with a stir pea were added MeCN (1 mL) and **27D** (99 mg, 0.12 mmol), yielding a brown solution. To a separate screw top test tube charged with a stir pea were added zonisamide (benzo[*d*]isoxazol-3-ylmethanesulfonamide; 42 mg, 0.20 mmol), DMSO (~5 drops), and MeCN (1 mL). Both solutions were allowed to cool to -30 °C in a cold-bath. KOtBu (0.10 mL, 20% w/w in THF, 0.16 mmol) was added to the zonisamide solution, yielding a heterogeneous mixture of an off-white salt; additional MeCN (1 mL) was added to the mixture in an attempt to increase its homogeneity. Both test tubes were allowed to chill at -30 °C for 30 s, after which the solution of **27D** was transferred to the salt mixture. The reaction was allowed to stir at -30 °C for 48 h, after which the reaction mixture was removed from the cold-bath and passed through a 15 mL fine porosity fritted funnel to remove solid from the reaction mixture. The reaction mixture was then diluted with DCM (5 mL) and washed with DI $H_2O$ (3 x 3 mL). The reaction mixture was dried with anhydrous $Na_2SO_4$. The $Na_2SO_4$ was removed by passing the mixture through a 15 mL medium porosity fritted funnel. The resulting filtrate was concentrated *in vacuo* to leave a brown oil, which was redissolved in minimal DCM and added to stirring chilled (-20 °C) pentane (50 mL), forming a grey-brown precipitate which was collected on a 15 mL fine porosity fritted funnel. The precipitate was washed with chilled (-20 °C) pentane (15 mL) and then desiccated under static vacuum overnight to give **48D** as the major product [41 mg, 39% (mass recovery)]. NMR data show the corresponding elimination product (**87D**) as a byproduct with approximately 15% the integration of the major species; distinguishable (non-overlapping) signals in the $^1$H NMR are labeled as "Elimination product," and signals in the $^{13}$C NMR are likewise labeled as "Elimination product." Attempts to purify and isolate the desired product *via* recrystallization (both slow evaporation and vapor-vapor diffusion) were unsuccessful.

**$^1$H NMR (600 MHz, $CD_3CN$) δ:** 8.11 (d, *J* = 2.0 Hz, 1H), 8.05 (d, *J* = 1.7 Hz, 1H), 7.85 (d, *J* = 2.4 Hz, 1H), 7.84 (d, *J* = 2.3 Hz, 1H), 7.78 (d, *J* = 2.4 Hz, 1H), 7.69 (dt, *J* = 8.0, 1.0 Hz, 1H), 7.58 (m, 2H), 7.51 (d, *J* = 8.6 Hz, 2H), 7.24 (d, *J* = 2.3 Hz, 1H), 7.22 (ddd, *J* = 8.0, 6.4, 1.5 Hz, 1H), 6.94 (d, *J* = 8.7 Hz, 2H), 6.37 (t, *J* = 2.2 Hz, 1H), 6.30 (t, *J* = 2.2 Hz, 1H), 6.25 (t, *J* = 2.2 Hz, 1H), 4.78 (m, 1H), 4.48 (m, 2H), 3.98 (m, 1H), 3.80 (s, 3H), 2.84 (ddd, *J* = 13.7, 11.1, 2.4 Hz, 1H), 2.13 (m, 1H), 1.73 (m, 1H), 1.69 (m, 1H), 1.48 (m, 1H), 1.14 (d, *J* = 11.7 Hz, 1H), 0.90 (d, *J* = 8.4 Hz, 9H). **$^{13}$C NMR (201 MHz, $CD_3CN$) δ:** 164.3, 158.8*, 151.5, 145.8, 144.4*, 143.9, 141.6, 137.9, 137.8, 137.4, 131.3, 130.2, 124.8, 123.9, 122.2, 114.7, 110.6, 107.7, 107.3, 107.1, 57.1, 56.7, 56.1 (d, $J_{CP}$ = 10.5 Hz), 55.9, 50.5, 45.0 (d, $J_{CP}$ = 3.9 Hz), 31.7, 28.8, 14.1 (d, $J_{CP}$ = 28.3 Hz). **CV (MeCN, 100 mV/s):** $E_{p,a}$ = +0.49 V (NHE). **APCI-HRMS (m/z):** $[M+H]^+$ calculated for $C_{33}H_{41}BN_9O_5PSW$ 901.2291; found 690.2108 corresponding to $[M-(C_8H_7N_2O_3S)]^+$, $C_{25}H_{34}BN_7O_2PW^+$ (**27D**).

***Note:** 2D NMR experiments (HSQC and HMBC) support the assignments that the carbons at 158.8 and 144.4 ppm overlap with the corresponding elimination product.

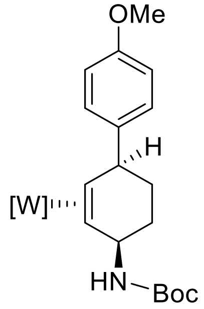


**49D**

To a screw top test tube were added **27D** (198 mg, 0.236 mmol) and dry THF (~2 mL), yielding a brown solution. To a separate screw top test tube charged with a stir pea was added *t*-butyl carbamate (115 mg, 0.982 mmol) followed by dry THF (~2 mL). To the *t*-butyl carbamate solution was added LiHMDS (0.48 mL, 0.48 mmol), and this solution was allowed to stir at room temperature for 15 min. Both solutions were then chilled to -60 °C in a cold-bath, after which the solution of **27D** was transferred to the solution of *t*-butyl carbamate and LiHMDS. The reaction was allowed to stir at -60 °C for 30 min, after which the reaction mixture was removed from the cold-bath, diluted with DCM (~5 mL), and washed with a saturated aqueous solution of $Na_2CO_3$ (~3 mL). The organic layer was concentrated *in vacuo* to leave a gray residue, which was redissolved in minimal MeCN and added to stirring pentane (~100 mL). The pentane was decanted, leaving the MeCN fraction, which was again concentrated *in vacuo* leaving a brown residue, which was redissolved in minimal DCM and added to stirring pentane (~100 mL). This formed an off-white precipitate which was collected on a 15 mL fine porosity fritted funnel. The precipitate was washed with pentane (10 x ~15 mL) and then desiccated under static vacuum overnight, yielding **49D** as the major product [40 mg, 21% (mass recovery)]. NMR data show the elimination product (**87D**) as a byproduct with approximately 9% the integration of the major species; we note that we were able to purify this compound on a silica gel micro-scale column with a gradient of eluents (hexanes → $Et_2O$ → MTBE → EtOAc → MeOH) with the desired product eluting in the EtOAc fraction.

**$^1$H NMR (800 MHz, $CD_3CN$) δ:** 8.26 (br s, 1H), 8.07 (br s, 1H), 7.87 (d, *J* = 2.4 Hz, 1H), 7.83 (d, *J* = 2.3 Hz, 1H), 7.77 (d, *J* = 2.5 Hz, 1H), 7.56 (d, *J* = 8.4 Hz, 2H), 7.29 (br d, *J* = 1.5 Hz, 1H), 6.94 (d, *J* = 8.7 Hz, 2H), 6.39 (br t, *J* = 2.1 Hz, 1H), 6.22 (t, *J* = 2.2 Hz, 1H), 6.21 (m, 1H), 5.34 (d, *J* = 8.4 Hz, 1H), 4.97 (m, 1H), 4.06 (m, 1H), 3.80 (s, 3H), 2.66 (t, *J* = 12.0 Hz, 1H), 2.07 (m, 1H), 1.67 (m, 1H), 1.38 (s, 9H), 1.37 (m, 1H), 1.27 (m, 1H), 1.11 (d, *J* = 12.0 Hz, 1H), 0.98 (d, *J* = 8.4 Hz, 9H). **$^{13}$C NMR (201 MHz, $CD_3CN$) δ:** 159.3, 156.6, 146.7, 144.8, 143.9, 142.2, 138.6, 137.9, 137.9, 131.0, 114.9, 108.3, 107.7, 107.2, 79.2, 58.6 (d, $J_{CP}$ = 10.8 Hz), 57.4, 56.5, 53.6, 46.3 (d, $J_{CP}$ = 2.7 Hz), 31.4, 29.4, 29.0, 14.2 (d, $J_{CP}$ = 27.9 Hz). **CV (MeCN, 100 mV/s):** $E_{p,a}$ = +0.52 V (NHE). **APCI-HRMS (m/z):** $[M+H]^+$ calculated for $C_{30}H_{44}BN_8O_4PW$ 806.2826; found 690.2106 corresponding to $[M-(NHBoc)]^+$, $C_{25}H_{34}BN_7O_2PW^+$ (**27D**).

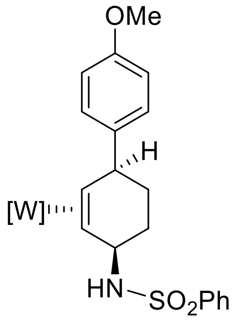

**50D**

To a screw top test tube were added MeCN (~2 mL) and **27D** (399 mg, 0.476 mmol), yielding a brown solution. To a separate screw top test tube charged with a stir pea was added benzenesulfonamide (82 mg, 0.52 mmol) followed by DMSO (~5 drops) and MeCN (~1 mL). To the benzenesulfonamide solution was added DBU (0.08 mL, 0.5 mmol). Both solutions were chilled to -30 °C in a cold-bath, after which the solution of **27D** was transferred to the solution of benzenesulfonamide and DBU. The reaction was allowed to stir at -30 °C for 21 h, after which the reaction mixture was removed from the cold-bath, diluted with DCM (~5 mL), and washed with ~9 mL of a 0.1 M aqueous solution of NaOH (3 x ~3 mL). The reaction mixture was dried over anhydrous $Na_2SO_4$, which was then filtered with a 30 mL medium porosity fritted funnel. The filtrate was concentrated *in vacuo* to leave a brown oil, which was redissolved in minimal DCM and added to stirring pentane (~200 mL), forming an off-white precipitate which was collected on a 30 mL fine porosity fritted funnel. The precipitate was washed with pentane (~30 mL) and then desiccated under static vacuum overnight, yielding **50D** (149 mg, 37%).

**$^1$H NMR (600 MHz, $CD_3CN$) δ:** 7.97 (d, $J$ = 2.0 Hz, 1H), 7.82 (d, $J$ = 2.2 Hz, 2H,), 7.81 (d, $J$ = 2.4 Hz, 1H), 7.79 (d, $J$ = 2.3 Hz, 1H), 7.49 (d, $J$ = 8.5 Hz, 2H), 7.37 (dd, $J$ = 1.2, 8.5 Hz, 2H), 7.22 (t, $J$ = 7.4 Hz, 1H), 7.08 (t, $J$ = 7.4 Hz, 2H), 6.93 (d, $J$ = 8.6 Hz, 2H,), 6.32 (m, 2H,), 6.15 (t, $J$ = 2.2 Hz, 1H), 5.75 (br s, 1H), 4.47 (m, 1H), 3.90 (m, 1H), 3.79 (s, 3H), 2.75 (ddd, $J$ = 2.7, 11.2, 14.2 Hz, 1H), 2.02 (m, 1H), 1.58 (m, 1H), 1.53 (m, 1H), 1.41 (m, 1H), 0.83 (d, $J$ = 8.4 Hz, 9H), 0.53 (d, $J$ = 11.1 Hz, 1H). **$^{13}$C NMR (201 MHz, $CD_3CN$) δ**: 158.7, 145.7, 144.3, 143.9, 142.9, 141.0, 137.7, 137.6, 137.3, 132.6, 130.2, 129.5, 127.2, 114.7, 107.5, 107.3, 107.2, 57.1, 55.9, 55.7, 55.0 (d, $J_{CP}$ = 10.8 Hz), 44.7 (d, $J_{CP}$ = 3.0 Hz), 32.2, 28.9, 14.3 (d, $J_{CP}$ = 28.3 Hz). **CV (MeCN, 75 mV/s):** $E_{p,a}$ = +0.50 V (NHE). **APCI-HRMS (m/z):** $[M+H]^+$ calculated for $C_{31}H_{40}BN_8O_4PSW$ 862.2546; found 690.2112 corresponding to $[M-(NHSO_2Ph)]^+$, $C_{25}H_{34}BN_7O_2PW^+$ (**27D**).

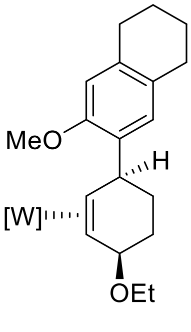

**52D**

To a 15 mL test tube were added **30D** (0.1946 g, 0.2179 mmol) and EtOH (3 mL). This solution was chilled to -60 °C for 5 min before adding a chilled (-60 °C) solution of KOH in EtOH (0.5 mL, 2.0 M, 1 mmol). After 72 h at -60 °C, reaction mix diluted with DCM (~5 mL) and washed with 20 mL of a saturated aqueous solution of $Na_2CO_3$ (2 x 10 mL). The organic layer was dried over anhydrous $Na_2SO_4$ and evaporated to an oil, which, was dissolved in minimal DCM and added to 250 mL of stirring pentane. A tan-white solid collected on 30 mL fine porosity fritted funnel and washed with pentane (2 x 15 mL) to give **52D** (0.0756 g, 44%).

**$^1$H NMR (800 MHz, $CD_2Cl_2$) δ:** 8.17 (d, $J$ = 2.0 Hz, 1H), 8.09 (d, $J$ = 1.9 Hz, 1H), 7.77 (d, $J$ = 2.3 Hz, 1H), 7.70 (d, $J$ = 2.2 Hz, 2H), 7.55 (s, 1H), 7.25 (d, $J$ = 2.2 Hz, 1H), 6.59 (s, 1H), 6.35 (t, $J$ = 2.2 Hz, 1H), 6.26 (t, $J$ = 2.2 Hz, 1H), 6.14 (t, $J$ = 2.3 Hz, 1H), 4.73 (m, 1H), 4.55 (t, $J$ = 5.7 Hz, 1H), 3.81 (s, 3H), 3.66 (dq, $J$ = 9.3, 6.9 Hz, 1H), 3.42 (tq, $J$ = 9.4, 7.0 Hz, 1H), 2.79 (dt, $J$ = 15.9, 5.4 Hz, 4H), 2.65 (t, $J$ = 12.1 Hz, 1H), 1.92 (m, 1H), 1.82 (m, 5H), 1.46 (m, 3H), 1.14 (t, $J$ = 7.0 Hz, 3H), 0.98 (d, $J$ = 8.2 Hz, 9H). **$^{13}$C NMR (201 MHz, $CD_2Cl_2$) δ:** 154.5, 143.2, 142.8, 140.8, 138.9, 136.7, 136.3, 136.1, 134.9, 130.1, 129.0, 110.7, 106.8, 105.9, 105.5, 81.9, 63.0, 58.3, 58.1 (d, $J_{CP}$ = 9.9 Hz), 55.8, 36.8, 29.9, 29.3, 27.7, 25.8, 24.1, 23.9, 16.3, 13.7 (d, $J_{CP}$ = 27.2 Hz).

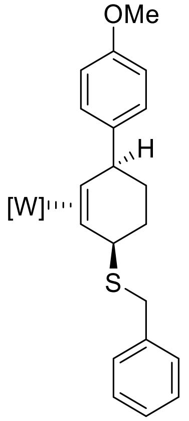


**53D**

To a 15 mL test tube were added **27D** (0.1500 g, 0.179 mmol) and MeCN (3 mL). In a separate test tube NaOtBu (0.0258 g, 0.268 mmol) was dissolved in THF (2 mL) followed by adding benzyl mercaptan (0.0667 g, 0.537 mmol). Both solutions were chilled to -30 °C for 15 min before adding the chilled (-30 °C) solution of **27D** to the solution of benzyl mercaptan. After 30 min at -30 °C, reaction mix was removed from the cold bath and quenched with saturated $NaHCO_3$ (~5 mL). This was then extracted with DCM (2 x 3 mL). The organic layer was dried over anhydrous $Na_2SO_4$ and evaporated to an oil, which, was dissolved in minimal DCM and added to 50 mL of stirring chilled pentane. A tan-white solid was collected on a 15 mL fine porosity fritted funnel to give **53D** (0.0670 g, 46%).

**$^1$H NMR (800 MHz, $CD_3CN$) δ:** 8.01 (d, $J$ = 2.0 Hz, 1H), 7.82 (d, $J$ = 2.3 Hz, 1H), 7.80 (d, $J$ = 2.2 Hz, 1H), 7.72 (d, $J$ = 2.5 Hz, 1H), 7.57 (d, $J$ = 2.0 Hz, 1H), 7.49 (d, $J$ = 8.6 Hz, 2H), 7.27 (t, $J$ = 7.3 Hz, 2H), 7.24 (d, $J$ = 6.6 Hz, 2H), 7.22 (d, $J$ = 2.2 Hz, 1H), 7.20 (t, $J$ = 7.2 Hz, 1H), 6.95 (d, $J$ = 8.6 Hz, 2H), 6.34 (t, $J$ = 2.2 Hz, 1H), 6.20 (t, $J$ = 2.2 Hz, 1H), 6.12 (t, $J$ = 2.2 Hz, 1H), 4.16 (t, $J$ = 4.9 Hz, 1H), 4.05 (t, $J$ = 6.9 Hz, 1H), 3.80 (s, 3H), 3.74 (d, $J$ = 13.6 Hz, 1H), 3.66 (d, $J$ = 13.6 Hz, 1H), 2.80 (ddd, $J$ = 13.5, 11.2, 2.0 Hz, 1H), 2.14 (m, 1H), 1.80 (m, 1H), 1.74 (m, 1H), 1.70 (m, 1H), 1.04 (d, $J$ = 11.3 Hz, 1H), 0.90 (d, $J$ = 8.4 Hz, 9H). **$^{13}$C NMR (201 MHz, $CD_3CN$) δ:**

158.6, 146.2, 144.1, 143.6, 141.5, 141.0, 137.8, 137.3, 137.3, 130.1, 129.8, 129.3, 127.5, 114.5, 107.5, 107.2, 106.9, 57.1, 56.5 (d, $J_{CP}$ = 10.2 Hz), 55.9, 46.0, 45.5 (d, $J_{CP}$ = 2.7 Hz), 36.5, 32.1, 26.6, 13.9 (d, $J_{CP}$ = 28.1 Hz). **APCI-HRMS (m/z):** $[M+H]^+$ calculated for $C_{32}H_{41}BN_7O_2PSW$ 813.2382; found, 690.2108 corresponding to $[M\text{-}SBn]^+$, $C_{25}H_{34}BN_7O_2PW^+$ (**27D**).

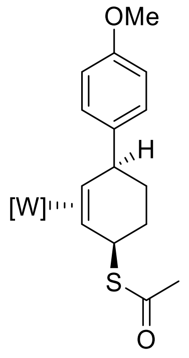


**54D**

To a 15 mL test tube were added **27D** (0.1000 g, 0.119 mmol) and MeCN (2 mL). In a separate test tube KSAc (0.0204 g, 0.179 mmol) was dissolved in MeCN (1.5 mL) and DMSO (0.5 mL). Both solutions were chilled to -30 °C for 15 min before adding the chilled (-30 °C) solution of **27D** to the solution of KSAc. After 1 h at -30 °C, reaction mix was removed from the cold bath and immediately quenched with saturated $NaHCO_3$ (~3 mL). This was then extracted with 6 mL of DCM (2 x 3 mL). The organic layer was dried over anhydrous $Na_2SO_4$ and evaporated to an oil, which, was dissolved in minimal DCM and added to 40 mL of stirring chilled pentane. A tan-white solid was collected on a 15 mL fine porosity fritted funnel to give **54D** (0.0290 g, 32%). The pentane filtrate was then evaporated to yield additional **54D** (0.0231 g, 25%; 57% overall).

**$^1$H NMR (800 MHz, $CD_3CN$) δ**: 8.05 (d, $J$ = 2.0 Hz, 1H), 8.01 (d, $J$ = 2.0 Hz, 1H), 7.85 (d, $J$ = 2.4 Hz, 1H), 7.82 (d, $J$ = 2.3 Hz, 1H), 7.79 (d, $J$ = 2.5 Hz, 1H), 7.47 (d, $J$ = 8.6 Hz, 1H), 7.25 (d, $J$ = 2.1 Hz, 1H), 6.95 (d, $J$ = 8.6 Hz, 1H), 6.37 (t, $J$ = 2.2 Hz, 1H), 6.30 (t, $J$ = 2.3 Hz, 1H), 6.23 (t, $J$ = 2.2 Hz, 1H), 5.07 (s, 1H), 4.08 (t, $J$ = 7.2 Hz, 1H), 3.80 (s, 3H), 2.84 (ddd, $J$ = 13.7, 11.1, 2.1 Hz, 1H), 2.23 (m, 1H), 2.20 (s, 3H), 1.84 (dtd, $J$ = 13.3, 6.7, 2.7 Hz, 1H), 1.63 (m, 1H), 1.47 (dddd, $J$ = 18.6, 9.1, 5.1, 2.8 Hz, 1H), 1.16 (d, $J$ = 11.4 Hz, 1H), 0.92 (d, $J$ = 8.4 Hz, 9H). **$^{13}$C NMR (201 MHz, $CD_3CN$) δ**: 197.5, 158.7, 146.0, 144.3, 143.6, 141.6, 137.9, 137.6, 137.4, 130.0, 114.7, 107.6, 107.2, 106.9, 56.7, 55.9, 55.7 (d, $J_{CP}$ = 10.6 Hz), 48.6, 44.9 (d, $J_{CP}$ = 3.1 Hz), 33.9, 31.1, 28.4, 14.0 (d, $J_{CP}$ = 28.2 Hz). Composition confirmed by single crystal X-ray diffraction.

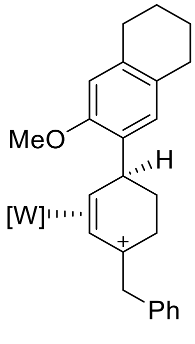


**55D**

To a 30 mL test tube were added **5D** (0.6872 g, 0.7442 mmol) and THF (15 mL). This solution was chilled to -30 °C for 10 min before slowly adding a chilled (-30 °C) solution of BnMgCl (2.0 mL, 1.0 M in $Et_2O$, 2.0 mmol) in THF (5 mL). After 18 h at -30 °C, the reaction mix was quenched with MeOH (1 mL) and eluted through a silica plug (60 mL medium porosity fritted funnel filled 3/4th with silica set in $Et_2O$) with $Et_2O$ (~150 mL). The filtrate was evaporated to an oil before adding a chilled (-30 °C) solution of HOTf (0.6006 g, 4.002 mmol) in DME (1 mL). This red solution was then added to 300 mL of stirring $Et_2O$. A bright orange precipitate was collected on 30 mL fine porosity fritted funnel and washed with $Et_2O$ (2 x 15 mL) to yield **55D** (0.3148 g, 43%).

**$^1$H NMR (600 MHz, 0 °C, $CD_3CN$) δ:** 8.21 (d, $J$ = 2.2 Hz, 1H), 8.06 (d, $J$ = 2.3 Hz, 1H), 7.99 (d, $J$ = 2.3 Hz, 1H), 7.94 (d, $J$ = 2.5 Hz, 1H), 7.93 (d, $J$ = 2.4 Hz, 1H), 7.90 (d, $J$ = 2.2 Hz, 1H), 7.29 (t, $J$ = 7.3 Hz, 2H), 7.24 (t, $J$ = 7.3 Hz, 1H), 7.18 (d, $J$ = 7.3 Hz, 2H), 6.89 (s, 1H), 6.64 (s, 1H), 6.53 (t, $J$ = 2.3 Hz, 1H), 6.47 (t, $J$ = 2.3 Hz, 1H), 6.40 (t, $J$ = 2.3 Hz, 1H), 4.91 (dd, $J$ = 7.4, 1.8 Hz, 1H), 4.63 (ddd, $J$ = 8.7, 6.6, 1.8 Hz, 1H), 4.28 (ddd, $J$ = 16.2, 7.3, 1.9 Hz, 1H), 3.76 (s, 3H), 3.56 (d, $J$ = 12.9 Hz, 1H), 3.09 – 2.96 (m, 2H), 2.79 (m, 1H), 2.71 (m, 2H), 2.58 (m, 2H), 1.90 (m, 1H), 1.73 (m, 4H), 1.10 – 0.96 (m, 10H). **$^{13}$C NMR (201 MHz, $CD_2Cl_2$) δ:** 182.1, 154.1, 146.7, 145.2, 141.6, 139.9, 139.2, 139.1, 137.0, 135.7, 134.4, 130.1, 129.4, 129.2, 128.3, 128.0, 111.5, 108.8, 108.7, 108.1, 99.3, 78.6 (d, $J_{CP}$ = 14.5 Hz), 55.7, 49.9, 35.8, 30.8, 30.4, 29.8, 29.0, 23.8, 23.6, 13.9 (d, $J_{CP}$ = 32.4 Hz). Composition confirmed by single crystal X-ray diffraction.

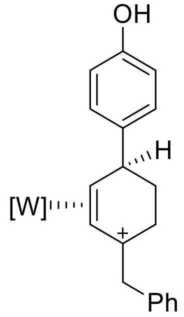


**85D**

To a 30 mL test tube were added **6D** (0.526 g, 0.615 mmol) and THF (3 mL). This solution was chilled to -30 °C for 10 min before slowly adding a chilled (-30 °C) solution of BnMgCl (2.0 mL, 1.0 M in $Et_2O$, 2.0 mmol) in THF (5 mL). After 25 h at -30 °C, the reaction mix was quenched with a saturated aqueous solution of $NH_4Cl$ (1 mL) and eluted through a silica plug (30 mL medium porosity fritted funnel filled 3/4th with silica set in $Et_2O$) with $Et_2O$ (~100 mL). The filtrate was evaporated to an oil before adding a chilled (-30 °C) solution of HOTf (0.489 g, 3.26 mmol) in DME (1 mL). This red solution was then added to 300 mL of stirring $Et_2O$. A bright orange precipitate was collected on 30 mL fine porosity fritted funnel and washed with $Et_2O$ (2 x 15 mL) to yield **85D** (0.220 g, 39%).

**$^1$H NMR (600 MHz, 0 °C, $CD_3CN$) δ:** 8.21 (d, $J$ = 2.2 Hz, 1H), 8.05 (d, $J$ = 2.4 Hz, 1H), 7.96 (d, $J$ = 2.4 Hz, 1H), 7.95 (d, $J$ = 2.6 Hz, 1H), 7.93 (d, $J$ = 2.5 Hz, 1H), 7.90 (d, $J$ = 2.2 Hz, 1H), 7.32 – 7.40 (m, 3H), 7.13 (d, $J$ = 7.1 Hz, 2H), 7.10 (d, $J$ = 8.5 Hz, 2H), 6.74 (d, $J$ = 8.5 Hz, 2H), 6.52 (t, $J$ = 2.3 Hz, 1H), 6.47 (t, $J$ = 2.3 Hz, 1H), 6.42 (t, $J$ = 2.3 Hz, 1H), 4.88 (dd, $J$ = 7.4, 1.8 Hz, 1H), 4.34 (ddd, $J$ = 16.3, 7.3, 1.9 Hz, 1H), 4.16 (m, 1H), 3.57 (d, $J$ = 13.3 Hz, 1H), 3.10 (d, $J$ = 13.2 Hz, 1H), 3.03 (m, 1H), 2.81 (ddd, $J$ = 20.1, 6.0, 3.8 Hz, 1H), 2.04 (m, 1H), 1.12 (m, 1H)*, 1.06 (d, $J$ = 9.8 Hz, 9H). **$^{13}$C NMR (201 MHz, $CD_3CN$) δ:** 184.8, 156.5, 147.6, 146.1, 143.2, 141.6, 140.4,

139.9, 139.5, 136.8, 130.1, 129.7, 129.3, 128.3, 116.3, 109.2, 108.8, 108.6, 99.6, 78.9 (d, $J_{CP}$ = 14.6 Hz), 49.4, 43.9, 33.7, 30.5, 13.8 (d, $J_{CP}$ = 32.7 Hz). Composition confirmed by single crystal X-ray diffraction.

***Note:** The signal at 1.12 ppm overlaps with a $Et_2O$ solvent impurity.

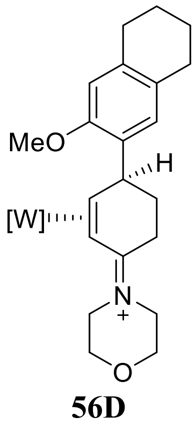


**56D**

To a 30 mL test tube were added **5D** (0.7783 g, 0.8429 mmol) and THF (~7 mL). This slurry was chilled to -30 °C for 5 min before adding room temperature morpholine (1.7360 g, 19.926 mmol). After 10 min at -30 °C, the reaction mixture was allowed to stir at room temperature. After 3 h at room temperature, the reaction mixture added to 450 mL of stirring $Et_2O$. A pale-yellow solid was collected on a 60 mL fine porosity fritted funnel and washed with $Et_2O$ (2 x 15 mL) to yield **56D** (0.5352 g, 65%).

**$^1H$ NMR (800 MHz, $CD_3CN$) δ:** 8.09 (d, $J$ = 2.1 Hz, 1H), 7.97 (d, $J$ = 2.4 Hz, 1H), 7.90 (m, 2H), 7.65 (d, $J$ = 2.3 Hz, 1H), 7.34 (d, $J$ = 2.1 Hz, 1H), 7.24 (s, 1H), 6.71 (s, 1H), 6.43 (t, $J$ = 2.3 Hz, 1H), 6.41 (m, 2H), 4.68 (m, 1H), 4.17 (m, 1H), 3.87 (dt, $J$ = 11.9, 3.6 Hz, 1H), 3.82 (s, 3H), 3.76 (m, 1H), 3.63 (m, 2H), 3.48 (dt, $J$ = 12.3, 3.8 Hz, 1H), 3.43 (m, 1H), 3.10 (m, 1H), 2.74 (m, 6H), 2.58 (d, $J$ = 9.0 Hz, 1H), 2.30 (m, 2H), 1.79 (m, 4H), 1.70 (m, 1H), 1.11 (d, $J$ = 9.2 Hz, 9H). **$^{13}C$ NMR (201 MHz, $CD_3CN$) δ:** 192.8, 155.2, 144.6, 143.8, 141.9, 139.1, 138.0, 137.1, 136.8, 135.4, 131.6, 128.8, 111.6, 107.5, 106.8, 105.9, 68.6, 67.5, 61.5, 60.2 (d, $J_{CP}$ = 10.0 Hz), 58.8, 56.4, 56.2, 47.2, 38.0, 30.3, 29.9, 29.5, 24.4, 24.2, 12.9 (d, $J_{CP}$ = 28.8 Hz). Composition confirmed by single crystal X-ray diffraction.

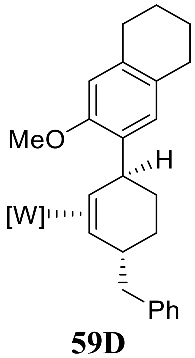


**59D**

To a 15 mL test tube were added **55D** (0.3130 g, 0.3182 mmol) and THF (5 mL). This solution was chilled to -60 °C for 20 min before slowly adding $NBu_4BH_4$ (0.2910 g, 1.131 mmol). After 18 h at -60 °C, the reaction mix was diluted with DCM (~5 mL) and washed with 20 mL of DI $H_2O$

(2 x 10 mL). The organic layer was dried over anhydrous $MgSO_4$ and evaporated to an oil. The film was dissolved in minimal DCM and added to 300 mL of stirring pentane. A tan-white solid was collected on a 30 mL fine porosity fritted funnel and washed with pentane (2 x 15 mL) to yield **59D** (0.1806 g, 68%).

**$^1$H NMR (800 MHz, $CD_2Cl_2$) δ:** 8.42 (d, $J$ = 1.9 Hz, 1H), 8.09 (d, $J$ = 1.9 Hz, 1H), 7.77 (d, $J$ = 2.3 Hz, 1H), 7.71 (d, $J$ = 2.3 Hz, 1H), 7.70 (d, $J$ = 2.4 Hz, 1H), 7.36 (s, 1H), 7.28 (d, $J$ = 2.2 Hz, 1H), 7.13 (t, $J$ = 7.6 Hz, 2H), 7.06 (t, $J$ = 7.3 Hz, 1H), 6.96 (d, $J$ = 7.3 Hz, 2H), 6.55 (s, 1H), 6.30 (t, $J$ = 2.2 Hz, 1H), 6.23 (t, $J$ = 2.2 Hz, 1H), 6.21 (t, $J$ = 2.2 Hz, 1H), 4.55 (d, $J$ = 7.8 Hz, 1H), 3.85 – 3.75 (m, 4H), 3.02 (ddd, $J$ = 16.3, 11.8, 2.3 Hz, 1H), 2.90 – 2.64 (m, 6H), 2.04 (m, 1H), 1.78 (m, 6H), 1.58 (m, 1H), 1.27 (m, 1H)*, 0.94 (d, $J$ = 8.3 Hz, 9H). **$^{13}$C NMR (201 MHz, $CD_2Cl_2$) δ:** 154.7, 147.7, 144.4, 143.0, 140.8, 140.0, 137.1, 136.8, 136.0, 134.7, 130.1, 129.4, 129.3, 128.1, 125.3, 110.8, 106.5, 106.1, 105.7, 61.5, 58.7 (d, $J_{CP}$ = 10.6 Hz), 55.6, 45.9, 44.5, 36.6, 34.2, 29.8, 29.2, 28.4, 24.1, 23.9, 13.7 (d, $J_{CP}$ = 27.3 Hz). Composition confirmed by single crystal X-ray diffraction.

***Note:** The signal at 1.27 ppm overlaps with a hexane solvent impurity.

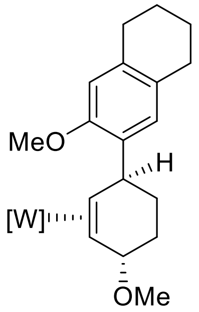


**26D**

To a 15 mL test tube were added **5D** (0.4521 g, 0.4896 mmol) and MeOH (5 mL). This solution was allowed to chill to -30 °C for 5 min before slowly adding $NaBH_4$ (0.2023 g, 5.348 mmol). After 30 min at -30 °C, the reaction mix was diluted with DCM (~5 mL) and washed with 20 mL of a saturated aqueous solution of $Na_2CO_3$ (2 x 10 mL). The organic layer was dried over anhydrous $Na_2SO_4$ and evaporated to an oil. The film was dissolved in minimal DCM and added to 250 mL of stirring pentane. Tan-white solid collected on 30 mL fine porosity fritted funnel and washed with pentane (2 x 15 mL) to yield **26D** (0.2390 g, 63%).

**$^1$H NMR (800 MHz, $CD_2Cl_2$) δ:** 8.91 (d, $J$ = 2.0 Hz, 1H), 8.14 (d, $J$ = 1.9 Hz, 1H), 7.73 (d, $J$ = 2.4 Hz, 1H), 7.72 (d, $J$ = 2.3 Hz, 1H), 7.58 (d, $J$ = 2.4 Hz, 1H), 7.40 (s, 1H), 7.29 (d, $J$ = 2.1 Hz, 1H), 6.56 (s, 1H), 6.32 (t, $J$ = 2.2 Hz, 1H), 6.19 (t, $J$ = 2.2 Hz, 1H), 6.13 (t, $J$ = 2.2 Hz, 1H), 5.26 (ddd, $J$ = 9.4, 5.8, 4.2 Hz, 1H), 4.56 (td, $J$ = 7.4, 6.5, 1.9 Hz, 1H), 3.80 (s, 3H), 3.19 (s, 3H), 2.86 (ddd, $J$ = 14.0, 11.8, 2.1 Hz, 1H), 2.77 (m, 4H), 2.04 (m, 1H), 2.99 (m, 1H), 1.90 (m, 1H), 1.81 (m, 4H), 1.73 (m, 1H), 1.26 (m, 1H), 0.86 (d, $J$ = 8.3 Hz, 9H). **$^{13}$C NMR (201 MHz, $CD_2Cl_2$) δ:** 154.5, 149.3, 142.9, 140.6, 139.4, 136.9, 136.0, 135.8, 134.9, 130.2, 129.7, 110.7, 106.7, 106.0, 105.0, 85.8, 60.6 (d, $J_{CP}$ = 10.7 Hz), 56.9, 55.7, 54.2, 36.5, 33.1, 29.8, 29.2, 26.8, 24.1, 23.9, 13.4 (d, $J_{CP}$ = 27.3 Hz). The proposed stereochemistry of **26D** is supported by an NOE interaction between a methine proton at 5.29 ppm (on a carbon at 85 ppm) and a singlet at 7.45 ppm, corresponding to an aromatic proton of 6-methoxytetralin.

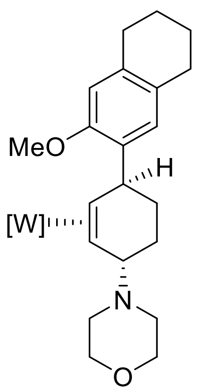


***trans*-46D**

To a 15 mL test tube were added **56D** (0.3056 g, 0.3123 mmol) and MeOH (5 mL). This slurry was chilled to -30 °C for 5 min before slowly adding $NaBH_4$ (0.2514 g, 6.646 mmol). After 30 min at -30 °C, the reaction mix was diluted with DCM (~5 mL) and washed with 20 mL of a saturated aqueous solution of $Na_2CO_3$ (2 x 10 mL). The organic layer was dried over anhydrous $Na_2SO_4$ and evaporated to an oil. The film was dissolved in minimal DCM and added to 300 mL of stirring pentane. A tan-white solid was collected on 30 mL fine porosity fritted funnel and washed with 30 mL of pentane (2 x 15 mL) to give ***trans*-46D** (0.1663 g, 64%).

Full characterization of ***trans*-46D** via $^1$H and $^{13}$C NMR were not pursued. However, it's proposed stereochemistry is supported by an NOE interaction between a methine proton at 4.20 ppm (on a carbon at 74 ppm) and a singlet at 7.41 ppm, corresponding to an aromatic proton of 6-methoxytetralin. **ESI-HRMS (m/z):** $[M]^+$ calculated for $C_{33}H_{48}BN_8O_3PW$ 830.3189; found, 830.3188.

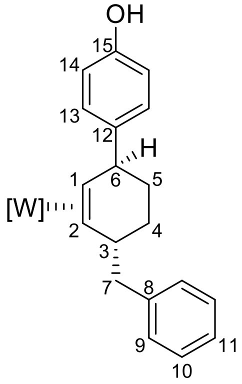


***trans*-58D**

A 30 mL test tube was charged with **85D** (0.252 g, 0.276 mmol), and THF (6 mL). This dark red solution was chilled to -30 °C and $NBu_4BH_4$ (0.133 g, 0.517 mmol) was added. The dark brown solution was allowed to stir at -30 °C for 1 h. The reaction mix was brought to room temperature and added to stirring DI $H_2O$ to produce a light gray precipitate. The precipitate was collected on a 15 mL fine porosity fritted funnel, washed with DI $H_2O$ (5 mL), and desiccated under static vacuum overnight before weighing (0.112 g, 53%).

**$^1$H NMR (800 MHz, $CD_3CN$) δ:** 8.36 (d, $J$ = 1.7 Hz, 1H, Pz3/5), 8.04 (d, $J$ = 1.8 Hz, 1H, Pz3/5), 7.86 (d, $J$ = 2.2 Hz, 1H, Pz3/5), 7.80 (d, $J$ = 2.3 Hz, 1H, Pz3/5), 7.79 (d, $J$ = 2.4 Hz, 1H, Pz3/5),

7.31 (d, $J$ = 8.4 Hz, 2H, H13), 7.28 (d, $J$ = 1.9 Hz, 1H, Pz3/5), 7.09 (t, $J$ = 7.4 Hz, 2H, H10), 7.04 (t, $J$ = 7.3 Hz, 1H, H11), 6.86 (d, $J$ = 7.0 Hz, 2H, H9), 6.76 (d, $J$ = 8.5 Hz, 2H, H14), 6.32 (t, $J$ = 2.2 Hz, 1H, Pz4), 6.26 (t, $J$ = 2.2 Hz, 1H, Pz4), 6.24 (t, $J$ = 2.2 Hz, 1H, Pz4), 4.06 (m, 1H, H6), 3.69 (m, 1H, H3), 3.07 (m, 1H, H1)*, 2.73 (dd, $J$ = 13.1, 5.9 Hz, 1H, H7x), 2.62 (dd, $J$ = 13.2, 8.6 Hz, 1H, H7y), 2.01 (m, 1H, $H5_{endo}$), 1.76 (m, 1H, $H4_{endo}$), 1.53 (overlapping, 2H, H2 and $H4_{exo}$)*, 1.35 (m, 1H, $H5_{exo}$), 0.86 (d, $J$ = 8.4 Hz, 9H, $PMe_3$). **$^{13}$C NMR (201 MHz, $CD_3CN$) δ:** 155.4 (C15), 148.1 (Pz3/5), 145.9 (C12), 144.5 (C8), 143.8 (Pz3/5), 141.6 (Pz3/5), 138.1 (Pz3/5), 137.6 (Pz3/5), 137.0 (Pz3/5), 130.2 (C13), 129.9 (C9), 128.7 (C10), 126.0 (C11), 115.8 (C14), 107.2 (Pz4), 107.1 (Pz4), 106.5 (Pz4), 61.4 (C2), 58.0 (d, $J_{CP}$ = 10.9 Hz, C1), 46.6 (C3), 44.8 (d, $J_{CP}$ = 2.2 Hz, C6), 44.5 (C6), 37.3 (C5), 29.3 (C4), 13.8 (d, $J_{CP}$ = 27.2 Hz, $PMe_3$).

***Note:** H1, H2, and $H4_{exo}$ overlap with an unknown tetrabutylammonium salt impurity (e.g., $NBu_4BH_4$ or $NBu_4OTf$). However, 2D NMR experiments (COSY, NOESY, HSQC, and HMBC) support the assignments of 3.07 ppm to H1 and 1.53 ppm to H2 and $H4_{exo}$.

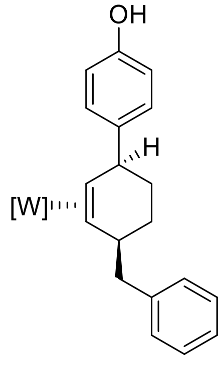


***cis*-58D**

To a 30 mL test tube were added **28D** (0.100 g, 0.121 mmol) and THF (~5 mL). The solution was allowed to chill to -60 ºC for 10 min before adding a chilled solution of BnMgCl (0.480 mL, 1.0M in hexanes, 0.480 mmol) in THF (~3 mL) was added dropwise. The reaction was allowed to stir at -60 ºC for 22 h. The reaction mixture was then diluted with DCM (~8 mL) and washed with 20 mL of DI $H_2O$ (2 x 10 mL). The organic later was dried over anhydrous $Na_2SO_4$ and evaporated to a thin film. The film was dissolved in minimal DCM and added to 150 mL of stirring pentane. A gray solid was collected on a 15 mL fine porosity fritted funnel and washed with pentane (2 x 10 mL) to yield ***cis*-58D** (0.0407 g, 44%).

**$^1$H NMR (800 MHz, $(CD_3)_2SO$) δ:** 9.07 (s, 1H), 8.33 (d, $J$ = 2.1 Hz, 1H), 8.03 (d, $J$ = 2.0 Hz, 1H), 8.02 (d, $J$ = 2.4 Hz, 1H), 8.01 (d, $J$ = 2.3 Hz, 1H), 7.98 (d, $J$ = 2.4 Hz, 1H), 7.33 (d, $J$ = 8.4 Hz, 2H), 7.25 (t, $J$ = 7.6 Hz, 2H), 7.19 – 7.16 (m, 2H), 7.16 – 7.13 (m, 1H), 6.78 – 6.75 (m, 2H), 6.48 (t, $J$ = 2.2 Hz, 1H), 6.42 (t, $J$ = 2.2 Hz, 1H), 6.29 (t, $J$ = 2.2 Hz, 1H), 4.02 – 3.97 (m, 1H), 3.21 (s, 1H), 2.74 – 2.64 (m, 2H), 2.36 (dd, $J$ = 13.4, 11.2 Hz, 1H), 2.07 – 2.01 (m, 1H), 1.24 – 1.20 (m, 1H), 1.16 (d, $J$ = 11.4 Hz, 1H), 1.15 – 1.10 (m, 1H), 1.03 – 0.99 (m, 1H), 0.98 (d, $J$ = 8.3 Hz, 9H). **$^{13}$C NMR (201 MHz, $(CD_3)_2SO$) δ:** 154.8, 142.9, 141.4, 140.4, 137.0, 136.7, 136.2, 129.0, 128.9, 128.2, 128.0, 125.5, 114.7, 113.8, 106.6, 106.4, 105.5, 57.7 (d, $J_{CP}$ = 9.9 Hz), 57.4, 47.9, 45.0 (d, $J_{CP}$ = 3.7 Hz), 40.4, 30.3, 24.3, 12.8 (d, $J_{CP}$ = 27.2 Hz).

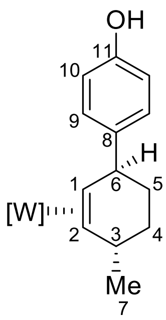


***trans*-57D**

A 30 mL test tube was charged with **89D** (0.040 g, 0.048 mmol), and THF (2 mL). This dark red solution was chilled to -30 °C and $NBu_4BH_4$ (0.061 g, 0.23 mmol) was added. The dark brown solution was allowed to stir at -30 °C for 15 min. The reaction mix was brought to room temperature and added to stirring DI $H_2O$ to produce a light gray precipitate. The precipitate was collected on a 15 mL fine porosity fritted funnel, washed with DI $H_2O$ (5 mL), and desiccated under static vacuum overnight before weighing (0.024 g, 73%).

**$^1$H NMR (800 MHz, $CD_3CN$) δ:** 8.29 (br s, 1H, Pz3/5), 8.08 (br s, 1H, Pz3/5), 7.81 (br s, 1H, Pz3/5), 7.80 (br s, 1H, Pz3/5), 7.71 (br s, 1H, Pz3/5), 7.33 (br s, 1H, Pz3/5), 7.30 (d, *J* = 7.9 Hz, 2H, H9), 6.79 (d, *J* = 8.1 Hz, 2H, H10), 6.34 (br s, 1H, Pz4), 6.21 (overlapping, 2H, 2 Pz4), 3.86 (m, 1H, H6), 3.54 (m, 1H, H3), 2.95 (m, 1H. H1), 1.98 (m, 1H, $H5_{endo}$), 1.72 (m, 1H, $H4_{endo}$), 1.60 (m, 1H, $H4_{exo}$)*, 1.55 (m, 1H, H2), 1.47 (m, 1H, $H5_{exo}$), 1.18 (d, *J* = 7.3 Hz, 3H, H7), 0.78 (d, *J* = 8.4 Hz, 9H, $PMe_3$). **$^{13}$C NMR (201 MHz, $CD_3CN$) δ:** 157.8 (C11), 147.8 (Pz3/5), 143.6 (Pz3/5), 143.5 (C8), 141.8 (Pz3/5), 137.8 (Pz3/5), 137.6 (Pz3/5), 136.9 (Pz3/5), 130.2 (C9), 116.5 (C10), 107.4 (Pz4), 107.0 (Pz4), 106.2 (Pz4), 63.0 (C2), 59.4 (d, $J_{CP}$ = 11.4 Hz, C1), 44.8 (C6), 36.4 (C3), 36.0 (C5), 32.2 (C4), 24.9 (C7), 13.6 (d, $J_{CP}$ = 27.9 Hz, $PMe_3$).

***Note:** $H4_{exo}$ overlaps with an unknown tetrabutylammonium salt impurity (e.g., $NBu_4BH_4$ or $NBu_4OTf$). However, 2D NMR experiments (COSY, NOESY, HSQC, and HMBC) support the assignments of 1.60 ppm to $H4_{exo}$.

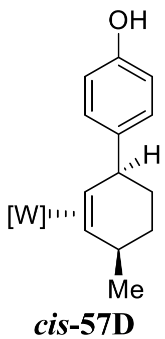


***cis*-57D**

To a 20 mL test tube were added **28D** (0.0533 g, 0.061 mmol) and THF (~3 mL). The solution was allowed to chill to -60 ºC for 10 min before adding a chilled solution of MeMgBr (0.022 mL, 3.0 M in $Et_2O$, 0.066 mmol) in THF (~2 mL) was added dropwise. The reaction was allowed to stir at -60 ºC for 43 h. The reaction mixture was then diluted with DCM (~6 mL) and washed with 20 mL of DI $H_2O$ (2 x 10 mL). The organic later was dried over anhydrous $Na_2SO_4$ and evaporated to a thin film. The film was dissolved in minimal DCM and added to 100 mL of stirring pentane.

A light-gray solid was collected on a 15 mL fine porosity fritted funnel and washed with (2 x 10 mL) to yield ***cis*-57D** (0.0081 g, 20%).

**$^1$H NMR (800 MHz, $CD_3CN$) δ**: 8.25 (d, $J$ = 2.0 Hz, 1H), 8.05 (d, $J$ = 1.4 Hz, 1H), 7.85 (d, $J$ = 2.3 Hz, 1H), 7.82 (d, $J$ = 2.2 Hz, 1H), 7.80 (d, $J$ = 2.4 Hz, 1H), 7.45 (d, $J$ = 6.3 Hz, 1H), 7.32 (d, $J$ = 2.0 Hz, 2H), 6.81 (d, $J$ = 8.5 Hz, 2H), 6.37 (t, $J$ = 2.2 Hz, 1H), 6.29 (t, $J$ = 2.2 Hz, 1H), 6.21 (t, $J$ = 2.2 Hz, 1H), 4.09 (d, $J$ = 2.9 Hz, 1H), 3.19 (d, $J$ = 9.0 Hz, 1H), 2.69 (t, $J$ = 11.9 Hz, 1H), 2.25 (tdd, $J$ = 13.2, 5.8, 2.4 Hz, 1H), 2.09 (s, 3H), 1.41 – 1.37 (m, 1H), 1.37 – 1.33 (m, 1H), 1.14 – 1.11 (m, 1H), 1.10 – 1.07 (m, 1H), 1.01 (d, $J$ = 8.2 Hz, 9H). **$^{13}$C NMR (201 MHz, $CD_3CN$) δ**: 155.5, 145.8, 143.9, 142.3, 141.6, 137.8, 137.3, 137.0, 130.5, 115.5, 107.4, 106.9, 106.4, 60.05, 59.7 (d, $J_{CP}$ = 9.9 Hz), 46.2 (d, $J_{CP}$ = 3.7 Hz), 34.9, 31.7, 29.2, 28.11, 13.6 (d, $J_{CP}$ = 9.3 Hz).

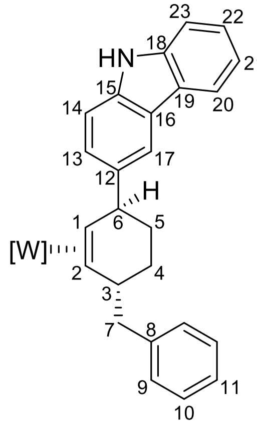


***trans*-60D**

A 30 mL test tube was charged with **90D** (0.407 g, 0.411 mmol), and THF (4 mL). This dark red solution was chilled to -30 °C and $NBu_4BH_4$ (0.175 g, 0.680 mmol) was added. The dark brown solution was allowed to stir at -30 °C for 1 h. The reaction mix was brought to room temperature and added to stirring DI $H_2O$ to produce a light gray precipitate. The precipitate was collected on a 15 mL fine porosity fritted funnel, washed with DI $H_2O$ (5 mL), and desiccated under static vacuum overnight before weighing (0.220 g, 64%).

**$^1$H NMR (800 MHz, $CD_3CN$) δ:** 9.22 (s, 1H, N-H), 8.40 (d, $J$ = 1.9 Hz, 1H, Pz3/5), 8.21 (d, $J$ = 1.7 Hz, 1H, H17), 8.11 (d, $J$ = 7.7 Hz, 1H, H13), 8.06 (d, $J$ = 2.0 Hz, 1H, Pz3/5), 7.86 (d, $J$ = 2.3 Hz, 1H, Pz3/5), 7.80 (overlapping, 2H, 2 Pz3/5), 7.58 (dd, $J$ = 8.3, 1.8 Hz, 1H, H20), 7.48 (overlapping, 2H, H14 and H23), 7.38 (ddd, $J$ = 8.2, 7.0, 1.2 Hz, 1H, H21), 7.33 (d, $J$ = 2.2 Hz, 1H, Pz3/5), 7.18 (ddd, $J$ = 7.9, 7.2, 0.9 Hz, 1H, H22), 7.11 (t, $J$ = 7.6 Hz, 2H, H10), 7.06 (m, 1H, H11), 6.90 (d, $J$ = 7.1 Hz, 2H, H9), 6.33 (t, $J$ = 2.2 Hz, 1H, Pz4), 6.28 (t, $J$ = 2.3 Hz, 1H, Pz4), 6.24 (t, $J$ = 2.2 Hz, 1H, Pz4), 4.35 (td, $J$ = 8.3, 2.9 Hz, 1H, H6), 3.82 (m, 1H, H3), 3.25 (ddd, $J$ = 17.0, 11.7, 3.0 Hz, 1H, H1), 2.79 (dd, $J$ = 13.2, 5.9 Hz, 1H, H7), 2.67 (dd, $J$ = 13.2, 8.6 Hz, 1H, H7), 2.13 (m, 1H, $H5_{endo}$)*, 1.86 (m, 1H, $H4_{endo}$), 1.62 (overlapping, 2H, $H4_{endo}$ and H2), 1.52 (m, 1H, $H5_{exo}$), 0.83 (d, $J$ = 8.4 Hz, 9H, $PMe_3$). **$^{13}$C NMR (201 MHz, $CD_3CN$) δ:** 148.1, 145.5, 144.6, 143.8, 141.7, 141.2, 139.0, 138.1, 137.6, 137.0, 129.9, 128.8, 127.6, 126.4, 126.0, 124.0, 123.9, 121.0, 120.2, 119.6, 111.8, 111.6, 107.2, 107.1, 106.5, 61.8, 58.4 (d, $J_{CP}$ = 10.9 Hz, C1), 46.9, 45.9 (d, $J_{CP}$ = 2.3 Hz, C6), 44.5, 38.3, 29.6, 13.9 (d, $J_{CP}$ = 28.1 Hz, $PMe_3$).

***Note:** H5$_{endo}$ overlaps with an $H_2O$ solvent impurity. However, 2D NMR experiments (COSY, NOESY, HSQC, and HMBC) support the assignments of 2.13 ppm to H5$_{endo}$.

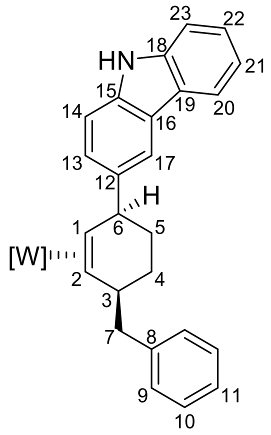


***cis*-60D**

To a 30 mL test tube were added **91D** (0.311 g, 0.346 mmol) and THF (3 mL). This orange mixture was chilled to -60 °C and a chilled (-60 °C) solution of BnMgCl (1.5 mL, 1.0 M in THF, 1.5 mmol) in THF (3 mL) was added dropwise. The resulting dark brown solution was allowed to stir at -60 °C for 18 h. The reaction was quenched with a saturated aqueous solution of $NH_4Cl$ and eluted through a silica column (30 mL medium porosity fritted funnel filled 3/4th with silica set in $Et_2O$) with $Et_2O$. Once the eluate was clear, the solvent was removed *in vacuo*, the product was dissolved in minimal DCM, and added to stirring pentane (150 mL). The resulting white precipitate was collected on a 15 mL fine porosity fritted funnel, washed with pentane (5 mL), and desiccated under static vacuum overnight before weighing (0.151 g, 52%).

**$^1$H NMR (800 MHz, $CD_3CN$) δ:** 9.25 (s, 1H, N-H), 8.43 (d, $J$ = 2.0 Hz, 1H, Pz3/5), 8.33 (d, $J$ = 1.7 Hz, 1H, H17), 8.14 (d, $J$ = 7.7 Hz, 1H, H20), 8.09 (d, $J$ = 2.0 Hz, 1H, Pz3/5), 7.87 (overlapping, 2H, 2 Pz3/5), 7.84 (d, $J$ = 2.3 Hz, 1H, Pz3/5), 7.66 (dd, $J$ = 8.2, 1.8 Hz, 1H, H13), 7.50 (overlapping, 2H, H14 and H23), 7.40 (overlapping, 2H, H22 and Pz3/5), 7.21 (overlapping, 6H, H9-H11 and H21), 6.42 (t, $J$ = 2.3 Hz, 1H, Pz4), 6.39 (t, $J$ = 2.3 Hz, 1H, Pz4), 6.23 (t, $J$ = 2.2 Hz, 1H, Pz4), 4.36 (t, $J$ = 5.0 Hz, 1H, H6), 3.34 (m, 1H, H3), 3.01 (m, 1H, H1), 2.82 (dd, $J$ = 13.5, 4.4 Hz, 1H, H7x), 2.59 (dd, $J$ = 13.5, 10.7 Hz, 1H, H7y), 2.18 (m, 1H, H5$_{endo}$), 1.49 (m, 1H, H5$_{exo}$), 1.39 (m, 1H, H2), 1.29 (m, 1H, H4$_{endo}$)*, 1.21 (m, 1H, H4$_{exo}$), 1.02 (d, $J$ = 8.3 Hz, 9H, $PMe_3$). **$^{13}$C NMR (201 MHz, $CD_3CN$) δ:** 145.3, 144.1, 142.9, 142.7, 141.8, 141.2, 139.2, 137.8, 137.6, 137.1, 130.1, 129.0, 128.0, 126.4, 126.2, 124.1, 123.7, 121.1, 120.5, 119.6, 111.8, 111.2, 107.5, 107.1, 106.5, 59.5 (br), 59.2 (br), 48.9, 47.2, 41.6, 32.0, 25.5, 13.6 (d, $J_{CP}$ = 27.3 Hz, $PMe_3$).

***Note:** H4$_{endo}$ overlaps with a pentane solvent impurity. However, 2D NMR experiments (COSY, NOESY, HSQC, and HMBC) support the assignments of 1.29 ppm to H4$_{endo}$.

**General Procedure 6:** To a 4-dram vial were added **24D**. acetone (~1-2 mL), and $NOPF_6$ or ferrocenium hexafluorophosphate. The reaction mix was removed from the glovebox and allowed to stir for approximately 30 min. The reaction mix was then evaporated to a film, dissolved in minimal $CH_2Cl_2$, and added to a 1:1 solution of hexanes and $Et_2O$ (~200 mL). A dark-brown precipitate was collected on a 30 mL fine porosity fritted funnel and washed with $Et_2O$ (100 mL).

The filtrate was evaporated to dryness, dissolved in minimal $CH_2Cl_2$, and purified via silica gel flash chromatography (0-100% elution gradient of EtOAc in hexanes, product in eluted at ~40% EtOAc).

**General Procedure 7:** To a 15 mL test tube were added **24D** and MeCN (~3 mL). This solution was chilled to -30 °C for 5 min before slowly adding a chilled (-30 °C) solution of $NOPF_6$ in MeCN (~1 mL). After 5 min, the reaction was removed from the glovebox, diluted with DCM (~5 mL) and washed a saturated aqueous solution of $Na_2CO_3$ (2 x 10 mL). The aqueous layers were combined and back-extracted with DCM (~5 mL), The organic layers were combined and dried over anhydrous $Na_2SO_4$ and evaporated onto basic alumina. The product was purified using flash chromatography on basic alumina.

**General Procedure 8:** To a 15 mL test tube were added **24D** and acetone (~3 mL). This solution was chilled to -60 °C for 5 min before slowly adding a chilled (-60 °C) solution of $NOPF_6$ in acetone (~1 mL). After 5 min, the reaction was removed from the glovebox, diluted with DCM (~5 mL) and washed with a saturated aqueous solution of $Na_2CO_3$ (2 x 10 mL). The aqueous layers were combined and back-extracted with DCM (~5 mL), The organic layers were combined and dried over anhydrous $Na_2SO_4$ and evaporated onto basic alumina. The product was purified via silica gel flash chromatography (0-100% elution gradient of EtOAc in hexanes, product in eluted at ~80% EtOAc).

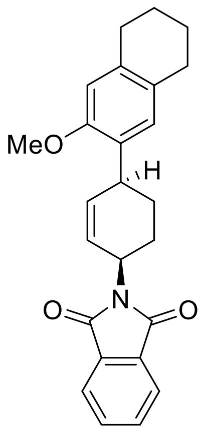


**65**

Used **General Procedure 6** with **43D** (0.0885 g, 0.0994 mmol) and $NOPF_6$ (0.0332 g, 0.1897 mmol). Tan-white solid (0.0310 g, 81%). Approximately 50% phthalimide impurity.

**$^1$H NMR (800 MHz, $CD_2Cl_2$) δ:** 7.83 (dd, *J* = 5.4, 3.0 Hz, 2H), 7.73 (dd, *J* = 5.4, 3.0 Hz, 2H), 7.36 (s, 1H), 6.57 (s, 1H), 5.88 (m, 1H), 5.82 (dd, *J* = 10.0, 1.8 Hz, 1H), 4.89 (ddd, *J* = 10.3, 5.7, 2.6 Hz, 1H), 3.85 (m 1H), 3.80 (s, 3H), 2.80 (m, 2H), 2.75 (m, 2H), 2.08 (m, 2H), 1.80 (m, 5H), 1.64 (dtd, *J* = 11.9, 6.2, 5.6, 3.5 Hz, 1H). **$^{13}$C NMR (201 MHz, $CD_2Cl_2$) δ:** 168.4, 154.9, 136.1, 134.2, 133.1, 132.5, 131.3, 130.2, 128.7, 128.6, 123.3, 110.8, 55.7, 48.0, 32.5, 29.9, 29.0, 28.3, 24.0, 23.8, 23.1. Composition confirmed by single crystal X-ray diffraction. **IR (ATR, cm$^{-1}$):** ν(CO) 1708 cm$^{-1}$.

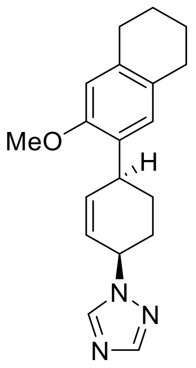


**66**

Used **General Procedure 6** with **45D** (0.2151 g, 0.2648 mmol) and ferrocenium hexafluorophosphate (0.0921 g, 0.2782 mmol). Clear oil (0.0425 g, 52%).

**$^{1}$H NMR (800 MHz, $CD_2Cl_2$) δ:** 8.22 (s, 1H), 7.91 (s, 1H), 6.82 (s, 1H), 6.59 (s, 1H), 6.16 (m, 1H), 5.99 (dt, *J* = 10.1, 3.3 Hz, 1H), 4.97 (m, 1H), 3.79 (s, 3H), 3.76 (m, 1H), 2.73 (m, 2H), 2.68 (m, 2H), 2.09 (m, 2H), 1.90 (m, 1H), 1.77 (m, 4H), 1.53 (dtd, *J* = 13.5, 9.2, 4.4 Hz, 1H). **$^{13}$C NMR (201 MHz, $CD_2Cl_2$) δ:** 155.2, 152.0, 142.7, 138.7, 136.6, 129.8, 129.3, 129.2, 123.6, 111.4, 55.7, 55.0, 35.9, 29.8, 29.0, 28.7, 25.5, 23.9, 23.7. **APCI-HRMS (m/z):** $[M+H]^+$ calculated for $C_{19}H_{23}N_3O$ 310.1914; found 310.1913.

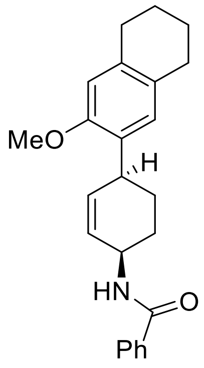


**67**

Used **General Procedure 6** with **40D** (0.1530 g, 0.1770 mmol) and $NOPF_6$ (0.0569 g, 0.3252 mmol). Yellow oil (0.0305 g, 48%).

**$^{1}$H NMR (800 MHz, $CD_2Cl_2$) δ:** 7.77 (d, *J* = 7.6 Hz, 2H), 7.51 (d, *J* = 7.4 Hz, 1H), 7.45 (t, *J* = 7.6 Hz, 2H), 6.82 (s, 1H), 6.58 (s, 1H), 6.24 (d, *J* = 8.2 Hz, 1H), 5.90 (m, 2H), 4.67 (m, 1H), 3.78 (s, 3H), 3.73 (m, 1H), 2.73 (m, 2H), 2.67 (m, 2H), 1.99 (m, 1H), 1.92 (m, 1H), 1.76 (m, 5H), 1.60 (m, 1H). **$^{13}$C NMR (201 MHz, $CD_2Cl_2$) δ:** 166.8, 155.2, 136.3, 135.5, 135.2, 131.7, 130.7, 129.1, 129.0, 128.9, 128.2, 127.2, 111.3, 55.8, 45.0, 34.9, 29.8, 29.0, 27.9, 26.9, 24.0, 23.7. **APCI-HRMS (m/z):** $[M+H]^+$ calculated for $C_{24}H_{27}NO_2$ 362.2115; found 362.2117. **IR (ATR, $cm^{-1}$):** ν(CO) 1700 $cm^{-1}$.

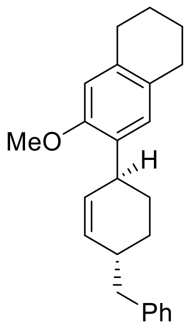


**69**

Used **General Procedure 6** with **59D** (0.1515 g, 0.1813 mmol) and $NOPF_6$ (0.0546 g, 0.312 mmol). Yellow oil (0.0350 g, 58%).

**$^{1}$H NMR (800 MHz, $CD_2Cl_2$) δ:** 7.29 (t, *J* = 7.6 Hz, 2H), 7.21 (m, 3H), 6.78 (s, 1H), 6.54 (s, 1H), 5.72 (m, 1H), 5.61 (dt, *J* = 10.1, 2.8 Hz, 1H), 3.76 (s, 3H), 3.72 (m, 1H), 2.75 to 2.56 (m, 6H), 2.46 (m, 1H), 2.02 (m, 1H), 1.75 (m, 5H), 1.37 (m, 2H). **$^{13}$C NMR (201 MHz, $CD_2Cl_2$) δ:** 155.2, 141.3, 135.8, 132.1, 132.1, 131.6, 129.6, 129.1, 128.7, 128.5, 126.2, 111.2, 55.8, 43.1, 38.0, 35.3, 30.7, 29.8, 29.5, 29.0, 24.0, 23.8. **APCI-HRMS (m/z):** $[M+H]^+$ calculated for $C_{24}H_{28}O$ 333.2213; found 333.2215.

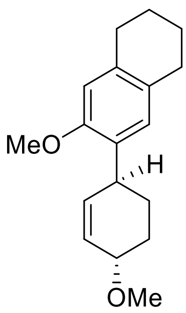


**71**

Used **General Procedure 8** with **26D** (0.1719 g, 0.2217 mmol) and $NOPF_6$ (0.0649 g, 0.371 mmol). Yellow oil (0.0250 g, 41%).

**$^{1}$H NMR (800 MHz, $(CD_3)_2SO$) δ:** 6.65 (s, 1H), 6.62 (s, 1H), 5.88 (dt, *J* = 10.2, 2.7 Hz, 1H), 5.65 (dt, *J* = 10.2, 2.1 Hz, 1H), 3.84 (m, 1H), 3.73 (s, 3H), 3.66 (m, 1H), 3.27 (s, 3H), 2.67 (m, 2H), 2.58 (m, 2H), 1.95 (m, 2H), 1.68 (m, 4H), 1.46 (m, 1H), 1.36 (m, 1H). **$^{13}$C NMR (201 MHz, $(CD_3)_2SO$) δ:** 154.3, 135.2, 132.7, 129.9, 129.0, 127.9, 127.8, 111.0, 74.3, 55.3, 55.0, 34.1, 28.9, 28.0, 27.8, 27.4, 23.0, 22.8. **APCI-HRMS (m/z):** $[M+H]^+$ calculated for $C_{18}H_{24}O_2$ 272.1776; found 241.1587 corresponding to $[M\text{-}OMe]^+$, $C_{17}H_{21}O^+$.

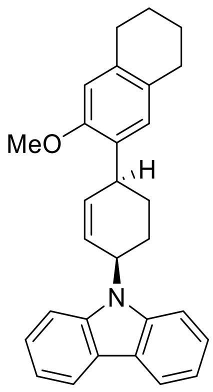


**75**

Used **General Procedure 6** with **44D** (0.2115 g, 0.2323 mmol) and ferrocenium hexafluorophosphate (0.1146 g, 0.3462 mmol). Clear oil (0.0295 g, 32%).

**$^{1}$H NMR (800 MHz, $CD_2Cl_2$) δ:** 8.11 (d, *J* = 7.6 Hz, 2H), 7.74 (m, 2H), 7.45 (t, *J* = 7.7 Hz, 2H), 7.22 (m, 3H), 6.63 (s, 1H), 6.25 (m, 1H), 6.16 (dt, *J* = 10.2, 3.3 Hz, 1H), 5.37 (ddd, *J* = 10.3, 5.5, 2.7 Hz, 1H), 3.94 (m, 1H), 3.84 (s, 3H), 2.91 to 2.71 (m, 4H), 2.24 (tdd, *J* = 13.1, 6.5, 2.8 Hz, 1H), 2.15 (m, 1H), 2.02 (m, 1H), 1.84 (m, 5H). **$^{13}$C NMR (201 MHz, $CD_2Cl_2$) δ:** 155.1, 140.6, 136.5, 133.7, 130.7, 130.5, 130.1, 128.6, 125.7, 123.6, 120.5, 120.5, 119.1, 111.3, 55.7, 52.8, 33.4, 29.9, 29.2, 28.3, 24.3, 24.0, 23.8. **APCI-HRMS (m/z):** $[M+H]^+$ calculated for $C_{29}H_{29}NO$ 408.2322; found 408.2314.

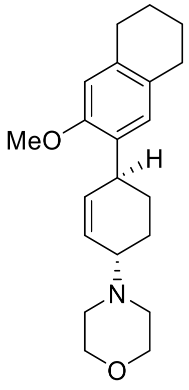


**76**

Used **General Procedure 7** with ***trans*-46D** (0.1440 g, 0.1734 mmol) and $NOPF_6$ (0.0368 g, 0.2103 mmol). Yellow oil (0.0150 g, 37%).

**$^{1}$H NMR (800 MHz, $(CD_3)_2CO$) δ:** 6.75 (s, 1H), 6.61 (s, 1H), 5.83 (m, 1H), 5.76 (m, 1H), 3.77 (s, 3H), 3.73 (m, 1H), 3.66 (m, 4H), 3.38 (m, 1H), 2.70 (m, 2H), 2.64 (m, 6H), 2.08 (ddd, *J* = 9.6, 4.6, 2.3 Hz, 1H), 1.88 (m, 1H), 1.73 (m, 5H), 1.45 (tdd, *J* = 12.9, 10.2, 2.6 Hz, 1H). **$^{13}$C NMR (201 MHz, $(CD_3)_2CO$) δ:** 155.9, 136.3, 135.4, 132.1, 129.9, 129.4, 129.0, 111.9, 67.7, 61.8, 55.9, 49.9, 35.9, 30.2, 29.4, 24.4, 24.2, 24.2, 23.9. **APCI-HRMS (m/z):** $[M+H]^+$ calculated for $C_{21}H_{29}NO_2$ 328.2271; found 328.2266.

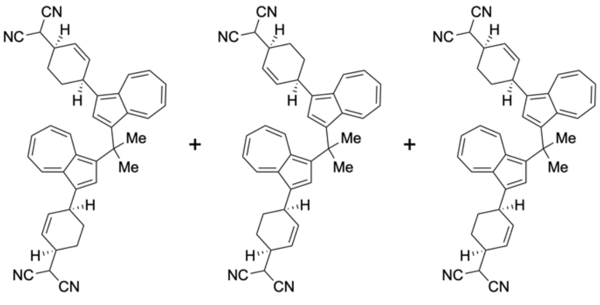

1 : 1 : 2

**80**

To a screw top test tube charged with a stir pea were added **39D** (238 mg, 0.307 mmol) and acetone (1 mL), giving a blue solution. Stirring was initiated, and $NOPF_6$ (67 mg, 0.38 mmol) was added to the solution of complex, upon which the reaction mixture immediately bubbled vigorously and turned brown. The reaction mixture was capped and allowed to stir at room temperature for 30 min. The test tube was then removed from the glovebox and the contents transferred to a 10 mL round bottom flask. The test tube was rinsed once with acetone (1 mL) and this rinse was added to the 10 mL round bottom flask as well. This solution was concentrated *in vacuo* to an oil *via* rotary evaporation. The oil was redissolved in minimal DCM and transferred into an Erlenmeyer flask containing 250 mL of stirring $Et_2O$:hexanes (1:1) to precipitate out metal complex decomposition, which was subsequently removed by filtering the mixture through a 30 mL medium porosity fritted funnel. $Et_2O$ (100 mL) was used to rinse the Erlenmeyer flask, and this $Et_2O$ was run through the 30 mL medium porosity fritted funnel as a further wash. The filtrate containing the organics were then transferred to a 500 mL round-bottom flask and concentrated *in vacuo* to a green-blue oil *via* rotary evaporation. This oil was redissolved in minimal DCM and dry-loaded onto silica *via* rotary evaporation. Purification with a Combi*Flash*® (100% hexanes → 100% EtOAc) showed two major UV-Vis absorption peaks corresponding to two major products. The later-eluting fraction was concentrated *in vacuo via* rotary evaporation to yield a green oil (12 mg, 13% overall). An NMR sample of the later-eluting fraction was prepared in $CD_2Cl_2$ and both $^{1}H$ and $^{13}C$ NMR spectra were obtained, which showed two chemically distinct species. Subsequent slow evaporation of this sample yielded green crystals, which upon analysis by single-crystal x-ray diffraction were found to be the byproduct 2,2'-((1*S*,1'*S*,4*R*,4'*R*)-(propane-2,2-diylbis(azulene-3,1-diyl))bis(cyclohex-2-ene-4,1-diyl))dimalononitrile and its enantiomer. From this, it was determined the second chemically-distinct species was the diastereomer, 2-((1*R*,4*S*)-4-(3-(2-(3-((1*R*,4*S*)-4-(dicyanomethyl)cyclohex-2-en-1-yl)azulen-1-yl)propan-2-yl)azulen-1-yl)cyclohex-2-en-1-yl)malononitrile. $^{1}H$ NMR and HMBC data show isopropylidenemalononitrile present in the sample as an impurity; peaks associated with isopropylidenemalononitrile are labeled in the $^{1}H$ and $^{13}C$ NMR spectra. The $^{1}H$ NMR otherwise shows purity of >90%, but further purification was not pursued.

**$^{1}$H NMR (800 MHz, $CD_2Cl_2$) δ:** 8.21 (m, 2H), 8.17 (s, 2H), 7.63 (d, *J* = 9.9 Hz, 1H), 7.59 (d, *J* = 9.8 Hz, 1H), 7.30 (m, 2H), 6.92 (m, 2H), 6.51 (m, 2H), 6.41 (m, 2H), 6.01 (m, 2H), 4.28 (m, 2H), 3.97 (d, *J* = 5.6 Hz, 1H), 3.96 (d, *J* = 5.6 Hz, 1H), 3.03 (m, 2H), 2.20 (m, 2H), 2.02 (m, 2H)*, 1.99 (s, 3H), 1.99 (s, 3H), 1.82 (m, 2H). **$^{13}$C NMR (201 MHz, $CD_2Cl_2$) δ:** 138.6, 138.5, 137.9, 137.9, 137.2, 137.2, 136.4, 136.4, 136.3, 136.3, 136.0, 135.9, 135.4, 133.2, 124.8, 124.8, 121.5, 121.2, 113.0, 113.0, 112.9, 112.9, 38.4, 38.2, 33.4, 33.4, 33.1, 32.9, 29.6, 29.6, 29.3, 29.3, 23.8, 23.8.

Non-solvent impurity peaks present in the $^{13}C$ NMR spectrum at 179.5, 130.0, 130.0, 112.5, 109.8, 86.6, 24.9, and 9.4 ppm. Composition confirmed by single crystal X-ray diffraction.

***Note:** The signal at 2.02 ppm overlaps with a EtOAc solvent impurity.

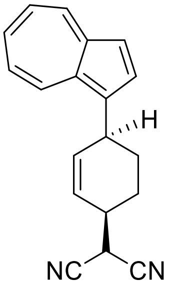


**81**

To a screw top test tube charged with a stir pea were added **39D** (238 mg, 0.307 mmol) and acetone (1 mL), giving a blue solution. Stirring was initiated, and $NOPF_6$ (67 mg, 0.38 mmol) was added to the solution of complex, upon which the reaction mixture immediately bubbled vigorously and turned brown. The reaction mixture was capped and allowed to stir at room temperature for 30 min. The test tube was then removed from the glovebox and the contents transferred to a 10 mL round bottom flask. The test tube was rinsed once with acetone (1 mL) and this rinse was added to the 10 mL round bottom flask as well. This solution was concentrated *in vacuo* to an oil *via* rotary evaporation. The oil was redissolved in minimal DCM and transferred into an Erlenmeyer flask containing 250 mL of stirring $Et_2O$:hexanes (1:1) to precipitate out metal complex decomposition, which was subsequently removed by filtering the mixture through a 30 mL medium porosity fritted funnel. $Et_2O$ (100 mL) was used to rinse the Erlenmeyer flask, and this $Et_2O$ was run through the 30 mL medium porosity fritted funnel as a further wash. The filtrate containing the organics were then transferred to a 500 mL round bottom flask and concentrated *in vacuo* to a green-blue oil *via* rotary evaporation. This oil was redissolved in minimal DCM and dry-loaded onto silica *via* rotary evaporation. Purification with a Combi*Flash*® (100% hexanes → 100% EtOAc) showed two major UV-Vis absorption peaks corresponding to two major products. The earlier-eluting fraction was concentrated *in vacuo via* rotary evaporation to yield a blue oil, which was desiccated under static vacuum overnight to yield **81** [16 mg, 19% (mass recovery)]. The $^{1}H$ NMR shows purity of >90%, but we note that we were able to further purify this compound on a semi-prep scale using an Agilent 1260 Infinity II HPLC equipped with 1260 DAD HS detector (wavelengths measured: 254 and 280 nm). The column used was a Phenomenex® Luna® Omega 5 µm Polar C18 semi-prep column with dimensions 10 mm inner-diameter/250 mm length. The sample concentration was 25 mg/mL. 12.5 mg of the sample was loaded onto the column by placing a 1,500 µL loop between the needle seat and the injection valve and repeating five 100 µL injections during the loading phase of the method. The sample was separated using a 60:40 MeOH:$H_2O$ gradient in which the concentration of MeOH was increased to 100% over 10 min (linear gradient). The flow rate was set to 4 mL/min and the retention time of the purified compound was found to be 9.649 min. Slow evaporation of the eluent led to a blue crystalline solid.

**$^{1}$H NMR (800 MHz, $CD_2Cl_2$) δ**: 8.35 (d, *J* = 9.6 Hz, 1H), 8.31 (d, *J* = 9.4 Hz, 1H), 7.85 (d, *J* = 3.8 Hz, 1H), 7.61 (t, *J* = 9.8 Hz, 1H), 7.34 (d, *J* = 3.8 Hz, 1H), 7.17 (t, *J* = 9.6 Hz, 1H), 7.14 (t, *J* = 9.6 Hz, 1H), 6.26 (m, 1H), 5.94 (d, *J* = 10.0 Hz, 1H), 4.23 (m, 1H), 3.87 (d, *J* = 6.1 Hz, 1H),

2.97 (m, 1H), 2.16 (m, 1H), 1.95 (m, 2H), 1.74 (m, 1H). **$^{13}$C NMR (201 MHz, $CD_2Cl_2$) δ:** 141.4, 138.2, 137.5, 137.4, 137.1, 135.6, 133.4, 132.3, 124.6, 123.1, 122.4, 117.1, 113.0, 112.9, 37.9, 33.5, 29.4, 29.1, 24.0. Non-solvent impurity peaks present in the $^{13}$C NMR spectrum at 160.4, 136.2, 130.1, 129.2, 129.0, 128.6, 125.8, 121.0, 114.4, 114.2, 112.6, and 55.8 ppm. **APCI-HRMS (m/z):** $[M]^+$ calculated for $C_{19}H_{16}N_2$ 272.1313; found, 272.1311. Composition confirmed by single-crystal X-ray diffraction.

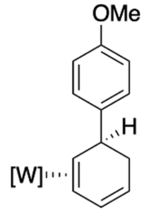


**87D**

This η$^2$-diene complex was isolated cleanly as a byproduct from multiple reactions (see NMR data); we note that it can be intentionally formed using DBU *via* the following procedure, albeit with a slight amount of DBU sill present: To a screw top test tube charged with a stir pea were added **27D** (48 mg, 0.057 mmol) and MeCN (1 mL). Stirring was initiated and DBU (0.01 mL, 0.07 mmol) was added. The reaction mixture was immediately diluted with DCM (5 mL), washed with 9 mL of a 0.1 M aqueous solution of NaOH (3 x 3 mL), and dried over anhydrous $Na_2SO_4$. The $Na_2SO_4$ was filtered with a 30 mL medium porosity fritted funnel and the filtrate concentrated *in vacuo* to an oil. The oil was subsequently redissolved in minimal DCM and transferred to stirring pentane (75 mL). This pentane solution was allowed to evaporate outside the glovebox, yielding crystals of the η$^2$-diene complex with excess DBU still present (by NMR). To remove the majority of excess DBU, these crystals were redissolved in DCM (5 mL), washed with 9 mL of 1.0 M aqueous NaOH (3 x 3 mL), and dried over anhydrous $Na_2SO_4$. The $Na_2SO_4$ was filtered with a 30 mL medium porosity fritted funnel and the filtrate concentrated *in vacuo* to an oil. The oil was subsequently redissolved in DCM (minimal) and transferred to stirring pentane (75 mL). This pentane solution was allowed to evaporate, yielding crystals of the η$^2$-diene complex (22 mg, 54%).

**$^1$H NMR (800 MHz, $CD_3CN$) δ:** 8.20 (d, $J$ = 2.0 Hz, 1H), 8.07 (d, $J$ = 2.1 Hz, 1H), 7.88 (d, $J$ = 2.3 Hz, 1H), 7.84 (d, $J$ = 2.3 Hz, 1H), 7.75 (d, $J$ = 2.4 Hz, 1H), 7.51 (d, $J$ = 8.6 Hz, 2H), 7.38 (d, $J$ = 2.2 Hz, 1H), 6.86 (d, $J$ = 8.7 Hz, 2H), 6.74 (ddd, $J$ = 9.3, 5.9, 3.2 Hz, 1H), 6.39 (t, $J$ = 2.2 Hz, 1H), 6.28 (t, $J$ = 2.2 Hz, 1H), 6.21 (t, $J$ = 2.2 Hz, 1H), 4.89 (ddd, $J$ = 9.2, 6.6, 2.3 Hz, 1H), 3.97 (d, $J$ = 8.0 Hz, 1H), 3.77 (s, 3H), 3.03 (m, 1H), 2.66 (t, $J$ = 10.3 Hz, 1H), 1.85 (dd, $J$ = 16.7, 6.5 Hz, 1H), 1.70 (ddd, $J$ = 10.2, 5.9, 2.0 Hz, 1H), 1.20 (d, $J$ = 8.4 Hz, 9H). **$^{13}$C NMR (201 MHz, $CD_3CN$) δ:** 158.8, 146.6, 145.5, 144.4, 141.9, 138.0, 137.3, 136.7, 136.0, 129.7, 115.6, 114.3, 107.6, 107.1, 106.8, 63.3 (d, $J_{CP}$ = 12.9 Hz), 55.8, 51.0, 45.6 (d, $J_{CP}$ = 2.2 Hz), 32.3, 13.2 (d, $J_{CP}$ = 28.4 Hz). Composition confirmed by single crystal X-ray diffraction.

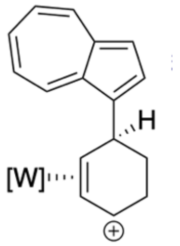


**79D**

To a screw top test tube were added DME (3 mL) and HOTf (50 mg, 0.33 mmol). To a separate screw top test tube charged with a stir pea were added **78D** (170 mg, 0.191 mmol) and MeOH (3 mL). Both solutions were chilled to -30 °C in a cold bath. $NaBH_4$ (150 mg, 3.97 mmol) was added to the solution of **78D** (slow addition necessary to prevent excess bubbling). The test tube was then capped, and the reaction was allowed to proceed at -30 °C for 30 min. The reaction mixture was removed from the cold bath, diluted with DCM (~5 mL), washed with DI $H_2O$ (3 x 3 mL), and dried over anhydrous $Na_2SO_4$. The $Na_2SO_4$ was filtered with a 30 mL medium porosity fritted funnel and the filtrate concentrated *in vacuo* to a dark green oil. The solution of HOTf was added to the oil, and the resulting solution was immediately transferred to stirring $Et_2O$ (100 mL). The resulting light blue precipitate was collected on a 15 mL fine porosity fritted funnel and desiccated under static vacuum overnight to provide **79D** (110 mg, 67%).

**$^1$H NMR (800 MHz, $CD_3CN$) δ:** 8.44 (d, $J$ = 2.3 Hz, 1H), 8.40 (d, $J$ = 9.4 Hz, 1H), 8.39 (d, $J$ = 9.7 Hz, 1H), 8.21 (d, $J$ = 2.2 Hz, 1H), 8.18 (d, $J$ = 3.9 Hz, 1H), 8.01 (d, $J$ = 2.3 Hz, 1H), 7.97 (d, $J$ = 2.1 Hz, 1H), 7.96 (d, $J$ = 2.2 Hz, 1H), 7.84 (d, $J$ = 2.6 Hz, 1H), 7.68 (t, $J$ = 9.8 Hz, 1H), 7.50 (d, $J$ = 3.9 Hz, 1H), 7.24 (t, $J$ = 9.7 Hz, 1H), 7.21 (t, $J$ = 9.8 Hz, 1H), 6.60 (t, $J$ = 6.6 Hz, 1H), 6.53 (t, $J$ = 2.3 Hz, 1H), 6.51 (t, $J$ = 2.3 Hz, 1H), 6.37 (t, $J$ = 2.4 Hz, 1H), 5.50 (t, $J$ = 7.3 Hz, 1H), 4.65 (dd, $J$ = 7.1, 15.9 Hz, 1H), 4.54 (dd, $J$ = 6.1, 11.0 Hz, 1H), 3.62 (m, 1H), 3.35 (dt, $J$ = 6.1, 19.4 Hz, 1H), 1.90 (m, 1H), 1.37 (m, 1H), 0.91 (d, $J$ = 9.8 Hz, 9H). **$^{13}$C NMR (201 MHz, $CD_3CN$) δ:** 149.4, 146.4, 143.5, 142.1, 139.7, 139.7, 139.6, 139.2, 138.3, 137.1, 136.7, 135.1, 134.7, 133.8, 124.2, 123.4, 118.7, 109.7, 109.0, 108.2, 105.8 (d, $J_{CP}$ = 3.3 Hz), 76.4 (d, $J_{CP}$ = 12.1 Hz), 37.4 (d, $J_{CP}$ = 2.2 Hz), 31.8, 26.4, 13.7 (d, $J_{CP}$ = 32.7 Hz). Non-solvent impurity peaks present at 146.4, 144.3, 142.8, 141.9, 138.9, 138.3, 137.8, 137.7, 137.5, 137.3, 137.3, 137.0, 136.5, 123.1, 122.9, 122.4, 121.6, 121.5, 107.7, 107.5, 107.2, 106.9, 72.5, 58.9, 55.9, 55.8, 52.7, 52.6, 50.0, 30.2, 28.3, 14.1, 7.1, and 6.8 ppm in the $^{13}$C NMR spectrum. **CV (MeCN, 100 mV/s):** $E_{p,c}$ = -0.97 V (NHE)

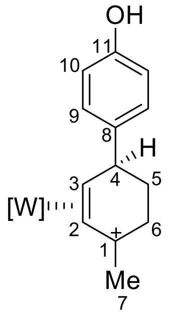


**89D**

To a 30 mL test tube were added **6D** (0.507 g, 0.593 mmol) and THF (10 mL). This solution was chilled to -30 °C for 10 min before slowly adding a chilled (-30 °C) solution of MeMgBr (1.0 mL,

3.0 M) in THF (5 mL). After 1 h at -30 °C, the reaction mix was quenched with a saturated aqueous solution of $NH_4Cl$ (1 mL) and eluted through a silica plug (60 mL medium porosity fritted funnel filled 3/4th with silica set in hexanes) with EtOAc (~150 mL). The filtrate was evaporated to an oil before adding a chilled (-30 °C) solution of HOTf (0.261 g, 1.74 mmol) in DME (1 mL). This red solution was then added to 300 mL of stirring $Et_2O$. A bright orange precipitate was collected on 30 mL fine porosity fritted funnel and washed with $Et_2O$ (2 x 15 mL) to yield **89D** (0.131 g, 26%).

**$^1$H NMR (800 MHz, $CD_3CN$) δ:** 8.19 (d, *J* = 2.0 Hz, 1H, Pz3/5), 8.04 (d, *J* = 2.0 Hz, 1H, Pz3/5), 7.98 (d, *J* = 2.0 Hz, 1H, Pz3/5), 7.93 (d, *J* = 1.9 Hz, 1H, Pz3/5), 7.89 (d, *J* = 1.9 Hz, 1H, Pz3/5), 7.70 (d, *J* = 2.1 Hz, 1H, Pz3/5), 7.27 (d, *J* = 8.4 Hz, 2H, H9), 6.84 (d, *J* = 8.4 Hz, 2H, H10), 6.53 (t, *J* = 2.1 Hz, 1H, Pz4), 6.46 (t, *J* = 2.0 Hz, 1H, Pz4), 6.35 (t, *J* = 2.1 Hz, 1H, Pz4), 4.61 (d, *J* = 7.0 Hz, 1H, H2), 4.25 (dd, *J* = 16.2, 7.2 Hz, 1H, H3), 4.16 (t, *J* = 7.9 Hz, 1H, H4), 3.06 (dt, *J* = 19.9, 7.8 Hz, 1H, $H6_{endo}$), 2.79 (dt, *J* = 19.9, 4.9 Hz, 1H, $H6_{exo}$), 2.07 (m, 1H, $H5_{endo}$), 1.95 (s, 3H, H7), 1.47 (m, 1H, $H5_{exo}$), 1.04 (d, *J* = 9.8 Hz, 9H, $PMe_3$).

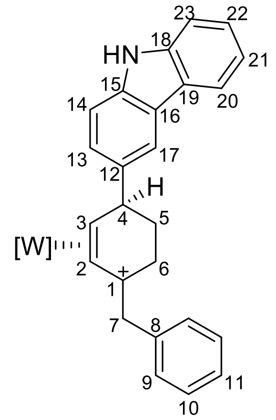


**90D**

To a 30 mL test tube were added **77D** (0.819 g, 0.882 mmol) and THF (6 mL). This solution was chilled to -30 °C for 5 min before slowly adding a chilled (-30 °C) solution of BnMgCl (5.0 mL, 1.0 M) in THF (6 mL). After 15 min at -30 °C, the reaction mix was added to a saturated aqueous solution of $NH_4Cl$ (30 mL) and extracted with DCM (2 x 15 mL). The organic layers were collected and evaporated to ~15 mL before eluting through a silica plug (30 mL medium porosity fritted funnel filled 3/4th with silica set in $Et_2O$) with $Et_2O$ (~100 mL). The filtrate was evaporated to an oil before adding a chilled (-30 °C) solution of HOTf (0.298 g, 1.99 mmol) in DME (1 mL). This red solution was then added to 300 mL of stirring $Et_2O$. A bright orange precipitate was collected on 30 mL fine porosity fritted funnel and washed with $Et_2O$ (2 x 15 mL) to yield **90D** (0.335 g, 38%).

**$^1$H NMR (600 MHz, $CD_3CN$) δ:** 9.38 (s, 1H, N-H), 8.23 (d, *J* = 2.2 Hz, 1H, Pz3/5), 8.10 (d, *J* = 7.9 Hz, 1H, H20), 8.06 (d, *J* = 1.7 Hz, 1H, H17), 8.05 (d, *J* = 2.3 Hz, 1H, Pz3/5), 8.01 (d, *J* = 2.3 Hz, 1H, Pz3/5), 7.95 (overlapping, 2H, Pz3/5), 7.94 (d, *J* = 2.2 Hz, 1H, Pz3/5), 7.51 (d, *J* = 8.2 Hz, 1H, H23), 7.46 (d, *J* = 8.3 Hz, 1H, H14), 7.42 (m, 1H, H22), 7.35 (dd, *J* = 8.3, 1.8 Hz, 1H, H13), 7.28 (t, *J* = 7.4 Hz, 2H, H10), 7.23 (overlapping, 2H, H21 and H11), 7.17 (d, *J* = 7.4 Hz, 2H, H9), 6.51 (t, *J* = 2.3 Hz, 1H, Pz4), 6.48 (t, *J* = 2.3 Hz, 1H, Pz4), 6.44 (t, *J* = 2.4 Hz, 1H, Pz4), 4.98 (dd, *J* = 7.3, 1.9 Hz, 1H, H2), 4.56 (dd, *J* = 16.4, 7.2 Hz, 1H, H3), 4.44 (t, *J* = 7.7 Hz, 1H, H4), 3.61 (d,

$J$ = 13.4 Hz, 1H, H7x), 3.13 (overlapping, 2H, H6$_{endo}$ and H7y), 2.85 (dt, $J$ = 19.7, 4.8 Hz, 1H, H6$_{exo}$), 2.15 (m, 1H, H5$_{endo}$), 1.29 (m, 1H, H5$_{exo}$), 1.05 (d, $J$ = 9.8 Hz, 9H, $PMe_3$).

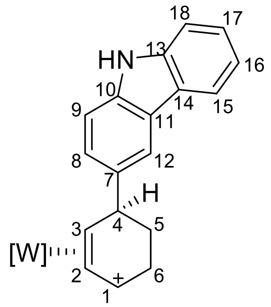


**91D**

To a 15 mL test tube were added **77D** (0.641 g, 0.690 mmol), and MeOH (5 mL). This solution was chilled to -30 °C for 5 min before adding $NaBH_4$ (0.211 g, 5.58 mmol). The reaction mixture was allowed to stir at -30 °C for 30 min before diluting with DCM (5 mL) and washing with 10 mL of DI $H_2O$ (2 x 5 mL). The organic layer was dried over anhydrous $MgSO_4$ and concentrated *in vacuo*. A chilled (-30 °C) solution of HOTf (0.356 g, 2.37 mmol) in DME (1 mL) was added to the resulting oil to give a dark red solution. This solution was then added to stirring $Et_2O$ (300 mL) to produce a precipitate which was collected on a 30 mL medium porosity fritted funnel, washed with $Et_2O$ (30 mL), and desiccated under static vacuum overnight to give **91D** (0.310 g, 50%).

**$^1$H NMR (600 MHz, $CD_3CN$) δ:** 9.48 (s, 1H, N-H), 8.44 (d, $J$ = 2.4 Hz, 1H, Pz3/5), 8.19 (d, $J$ = 2.3 Hz, 1H, Pz3/5), 8.16 (d, $J$ = 1.7 Hz, 1H, H12), 8.15 (d, $J$ = 7.9 Hz, 1H, H15), 8.00 (d, $J$ = 2.4 Hz, 1H, Pz3/5), 7.97 (d, $J$ = 2.3 Hz, 1H, Pz3/5), 7.96 (d, $J$ = 2.3 Hz, 1H, Pz3/5), 7.83 (d, $J$ = 2.4 Hz, 1H, Pz3/5), 7.54 (d, $J$ = 8.3, 1H, H18), 7.52 (d, $J$ = 8.2 Hz, 1H, H9), 7.45 (dd, $J$ = 8.3, 1.8 Hz, 1H, H8), 7.42 (ddd, $J$ = 8.3, 7.1, 1.2 Hz, 1H, H17), 7.22 (ddd, $J$ = 8.0, 7.1, 1.0 Hz, 1H, H15), 6.57 (t, $J$ = 7.0 Hz, 1H, H1), 6.53 (t, $J$ = 2.4 Hz, 1H, Pz4), 6.50 (t, $J$ = 2.3 Hz, 1H, Pz4), 6.36 (t, $J$ = 2.3 Hz, 1H, Pz4), 5.47 (t, $J$ = 7.3 Hz, 1H, H2), 4.56 (dd, $J$ = 15.8, 7.3 Hz, 1H, H3), 3.94 (dd, $J$ = 11.0, 6.1 Hz, 1H, H4), 3.57 (m, 1H, H6$_{endo}$), 3.36 (dt, $J$ = 19.3, 6.2 Hz, 1H, H6$_{exo}$), 1.88 (m, 1H, H5$_{endo}$), 1.36 (m, 1H, H5$_{exo}$), 1.00 (d, $J$ = 9.8 Hz, 9H, $PMe_3$).

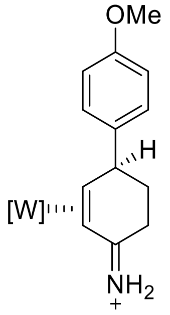


**84D**

A solution of **7D** (0.105 g, 0.121 mmol) in MeOH (2 mL) was chilled to -30 °C over the course of 20 min in a 4-dram vial. To this solution was added 2 mL of a 7 N solution of $NH_3$/MeOH (14.0 mmol) and the reaction mixture was allowed to sit at -30 °C over the course of 30 min. The reaction mixture was then allowed to sit at room temperature for another 30 min before the solution was added to 200 mL of stirring $Et_2O$. The resulting reaction mixture was allowed to sit at -30 °C to

induce precipitation over a period of 16 h. The next day a fine yellow-white crystalline solid had developed in solution and this was filtered through a 15 mL fine porosity fritted funnel. The resulting light-yellow solid was then washed with $Et_2O$ (2 x 10 mL) and allowed to dry over a period of 24 h under static vacuum in a desiccator before a mass was taken (0.072g, 70%).

**$^1$H NMR (600 MHz, $CD_3CN$) δ:** 8.13 (d, $J$ = 2.2 Hz, 1H), 8.07 (s, 1H), 7.97 (d, $J$ = 2.3 Hz, 1H), 7.94 (d, $J$ = 2.4 Hz, 1H), 7.87 (d, $J$ = 2.5 Hz, 1H), 7.61 (m, 2H), 7.44 (m, 3H), 6.96 (d, $J$ = 8.6 Hz, 1H), 6.45 (t, $J$ = 2.3 Hz, 1H), 6.40 (t, $J$ = 2.2 Hz, 1H), 6.32 (t, $J$ = 2.3 Hz, 1H), 4.19 (ddd, $J$ = 9.2, 5.7, 2.9 Hz, 1H), 3.80 (s, 3H), 3.67 (ddd, $J$ = 13.8, 8.6, 2.8 Hz, 1H), 2.82 (ddd, $J$ = 16.5, 10.9, 5.2 Hz, 1H), 2.57 (dtd, $J$ = 16.8, 4.9, 1.7 Hz, 1H), 2.50 (d, $J$ = 8.7 Hz, 1H), 2.19 (dq, $J$ = 13.5, 5.2 Hz, 1H), 1.69 (dtd, $J$ = 14.1, 10.4, 4.8 Hz, 1H), 0.99 (d, $J$ = 9.2 Hz, 9H). **$^{13}$C NMR (201 MHz, $CD_3CN$) δ:** 192.8, 159.3, 145.4, 143.7, 142.7, 142.2, 139.0, 138.7, 138.5, 129.7, 115.1, 108.5, 108.0, 107.8, 72.3 (d, $J_{CP}$ = 14.0), 56.6, 55.9, 36.0, 29.1, 13.9 (d, $J_{CP}$ = 30.6). Composition confirmed by single-crystal X-ray diffraction.

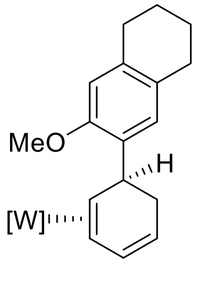


**86D**

Attempts to grow crystals from a DCM/pentane solution of **51D** resulted in the formation of **86D**. Composition confirmed by single-crystal X-ray diffraction.

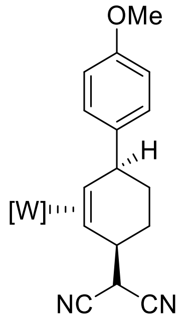


**88D**

To a screw top test tube charged with a stir pea were added THF (~1 mL) and **27D** (100 mg, 0.119 mmol). To a separate screw top test tube charged with a stir pea were added malononitrile (125 mg, 1.89 mmol) and THF (~1 mL). Both solutions were allowed to cool to -30 °C in a cold-bath for 30 s. KOtBu (0.45 mL, 20% w/w in THF, 0.72 mmol) was added to the malononitrile solution. The resultant mixture was allowed to stir at -30 °C for a further 30 s, after which this solution was transferred to the solution of **27D**. The reaction was allowed to stir at -30 °C for 22 h, after which the reaction mixture was removed from the cold-bath, diluted with DCM (~5 mL), and washed with DI $H_2O$ (3 x ~3 mL). The reaction mixture was dried with anhydrous $Na_2SO_4$ and subsequently run through a 30 mL medium porosity fritted funnel to remove the $Na_2SO_4$. The

resulting filtrate was concentrated *in vacuo* to leave a brown oil, which was redissolved in minimal DCM and added to stirring chilled (-20 °C) pentane (~50 mL), forming a brown precipitate which oiled-out. Therefore, solvent was removed *in vacuo* to leave a brown oil which was desiccated under static vacuum overnight. This oil was then redissolved in DCM (~5 mL). The resultant solution was washed with DI $H_2O$ (3 x ~3 mL) and dried with anhydrous $Na_2SO_4$. The reaction mixture was run through a 30 mL medium porosity fritted funnel to remove the $Na_2SO_4$. The filtrate was concentrated *in vacuo* to leave a brown oil, which was redissolved in minimal DCM and added to stirring chilled pentane (-20 °C, ~50 mL), forming a brown precipitate which was collected on a 15 mL fine-porosity fritted funnel. The precipitate was washed with chilled pentane (-20 °C, ~15 mL) and then desiccated under static vacuum overnight, yielding **88D** (30 mg, 33%).

**$^1$H NMR (600 MHz, $CD_3CN$) δ:** 8.04 (d, $J$ = 2.1 Hz, 2H), 7.86 (m, 2H), 7.84 (dt, $J$ = 0.7, 2.4 Hz, 1H), 7.59 (d, $J$ = 8.4 Hz, 2H), 7.36 (d, $J$ = 2.2 Hz, 1H), 6.95 (d, $J$ = 8.7 Hz, 2H), 6.37 (t, $J$ = 2.2 Hz, 1H), 6.33 (t, $J$ = 2.2 Hz, 1H), 6.26 (t, $J$ = 2.2 Hz, 1H), 4.16 (t, $J$ = 5.3 Hz, 1H), 3.97 (d, $J$ = 5.7 Hz, 1H), 3.80 (s, 3H), 3.63 (m, 1H), 2.78 (t, $J$ = 12.1 Hz, 1H), 2.20 (m, 1H), 1.84 (m, 1H), 1.57 (m, 1H), 1.47 (m, 1H), 1.02 (m, 1H) 0.99 (d, $J$ = 8.4 Hz, 9H). **$^{13}$C NMR (201 MHz, $CD_3CN$) δ**: 158.9, 145.4, 144.2, 142.1, 141.7, 138.1, 138.0, 137.5, 130.5, 115.0, 114.9, 114.5, 107.7, 107.4, 107.4, 57.2 (d, $J_{CP}$ = 10.9 Hz), 55.9, 53.0, 45.5 (d, $J_{CP}$ = 3.3 Hz), 41.5, 34.3, 30.8, 23.8, 13.6 (d, $J_{CP}$ = 28.3 Hz). Composition confirmed by single crystal X-ray diffraction.

**Note:** A free malononitrile signal is present in the $^1$H NMR at 3.79 ppm; HMBC data indicate $^{13}$C NMR signals associated with the free malononitrile at 9.9 and 112.0 ppm.

Formation of Binuclear Complexes

When **3P** is treated 0.5 eq of thiophene, the binuclear complex **78** is observed. In the $^1$H NMR spectrum of **78**, two binuclear complexes are present in a 1:1 ratio. Since a racemic sample of **1** was used, the two complexes are attributed to a “homobinuclear” species, where two molecules of **(R)-3P** (or two of **(S)-31P**) add as electrophiles to thiophene and a "heterobinuclear” species where one molecule of **(R)-3P** and one of **(S)-3P** adds.

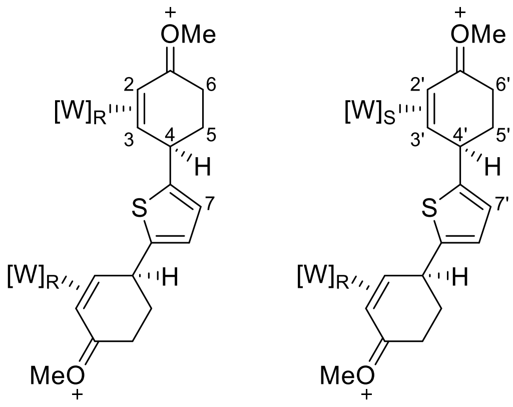


**78**

For $^{1}$H NMR assignments, homobinuclear species (left) = HX (where X is carbon number) and heterobinuclear (right) = HX'.

Used **General Procedure 3** with **1** (0.1771 g, 0.2898 mmol), HOTf (0.1809 g, 1.205 mmol) in EtCN (1 mL), and thiophene (0.149 mM, 1.0 mL) in EtCN (1 mL). Tan-orange solid (0.0747 g, 16%).

**$^{1}$H NMR (600 MHz, $CO(CD_3)_2$) δ:** 8.32 (overlapping, 2H, Pz3/5 and Pz3/5'), 8.19 (overlapping, 6H, Pz3/5 and Pz3/5'), 8.01 (overlapping, 2H, Pz3/5 and Pz3/5'), 7.89 (overlapping, 2H, Pz3/5 and Pz3/5'), 6.84 (s, 1H, H7 or H7'), 6.82 (s, 1H, H7 or H7'), 6.58 (overlapping, 4H, Pz4 and Pz4'), 6.42 (overlapping, 2H, Pz4 and Pz4'), 4.93 (overlapping, 2H, H2 and H2'), 4.55 (s, 3H, OMe or OMe'), 4.51 (s, 3H, OMe or OMe'), 3.96 (overlapping, 2H, H4 and H4'), 3.12 (overlapping, 2H, H6x and H6x'), 2.97 (overlapping, 2H, H6y and H6y'), 2.73 (overlapping, 2H, H3 and H3'), 2.59 (overlapping, 2H, H5x and H5x'), 2.18 (overlapping, 2H, H5y and H5y'), 1.23 (overlapping, 18H, $PMe_3$ and $PMe_3$').

<u>NMR Spectra</u>

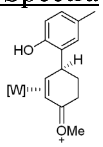


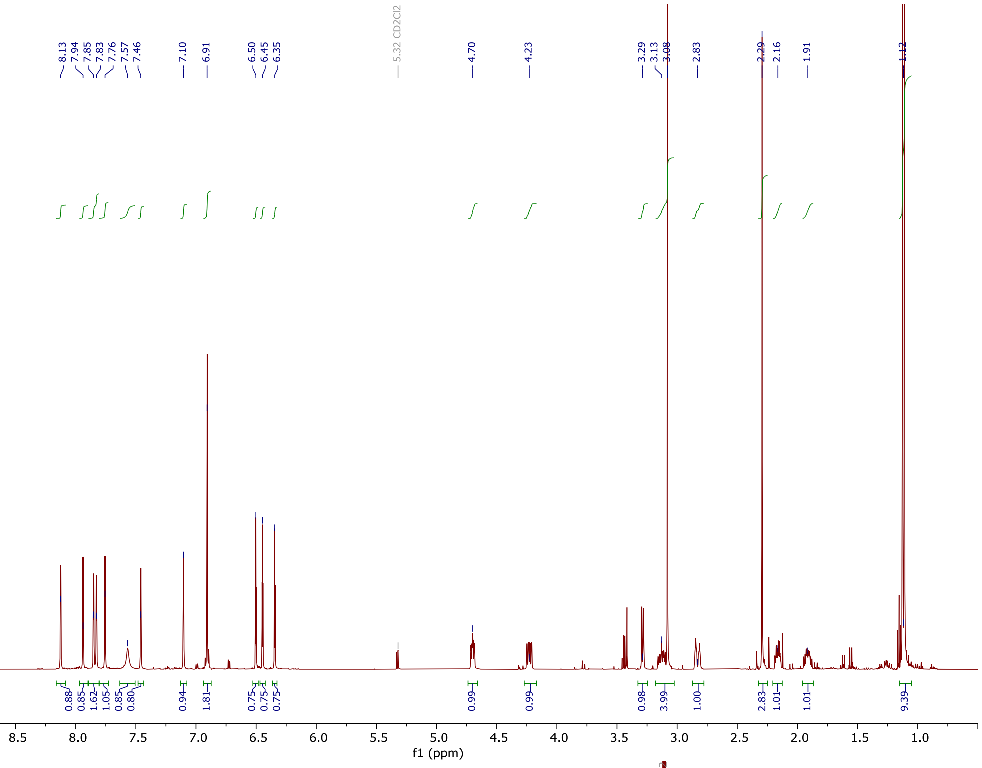

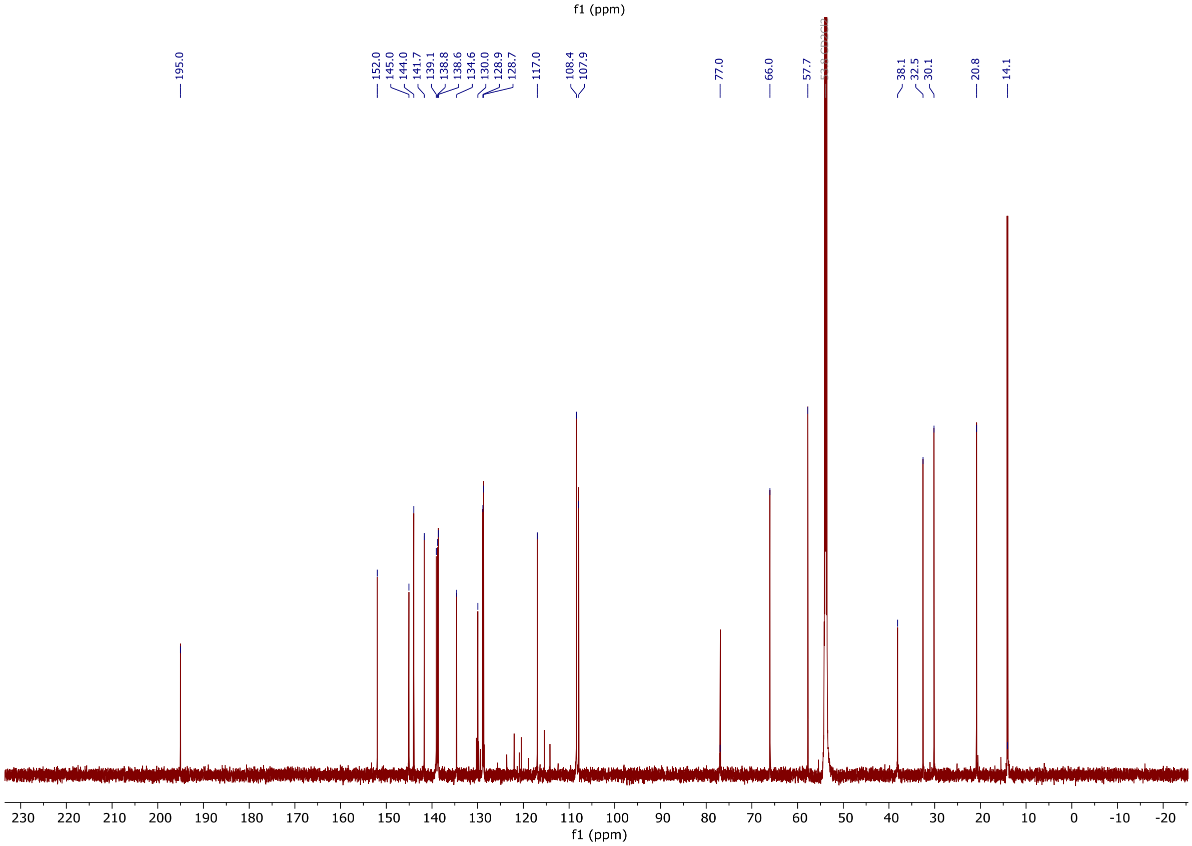

**Fig. S1.** Compound 9D $^{1}$H NMR (600 MHz, $CD_2Cl_2$, 25 °C, top) and $^{13}$C NMR (201 MHz, $CD_2Cl_2$, 25 °C, bottom).

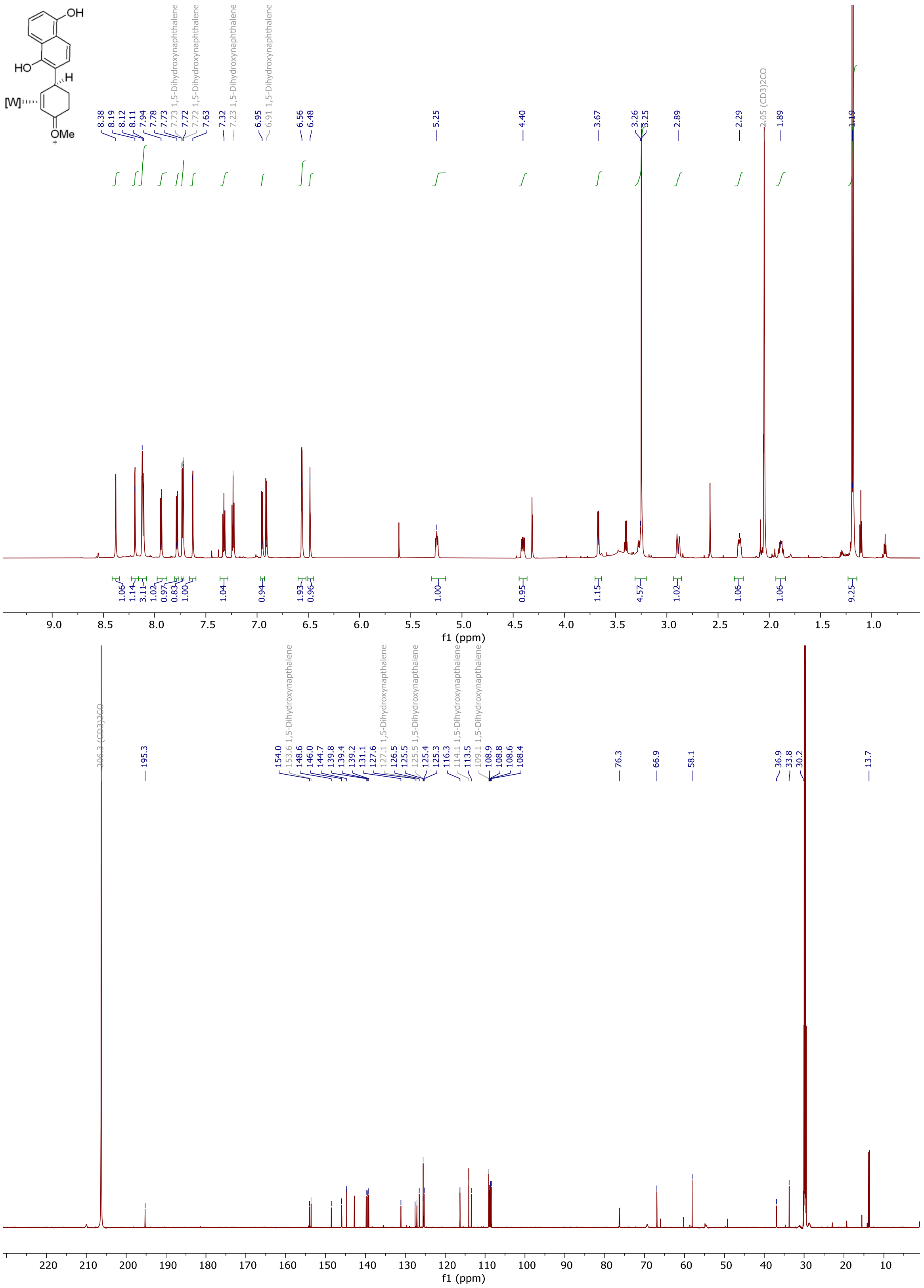


**Fig**. **S2.** Compound 10D $^{1}H$ NMR (800 MHz, $(CD_3)_2CO$, 25 °C, top) and $^{13}C$ NMR (201 MHz, $(CD_3)_2CO$, 25 °C, bottom).

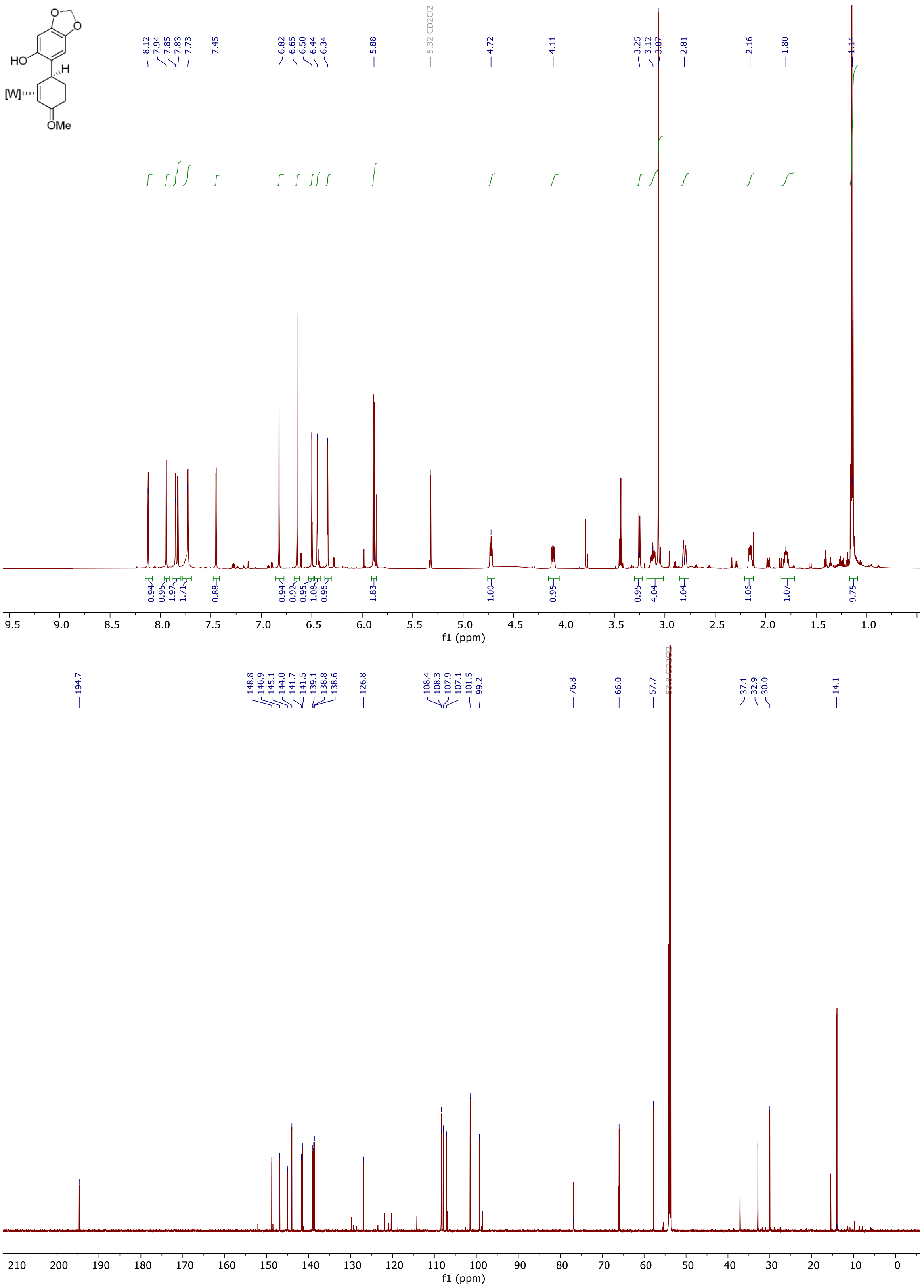


**Fig**. **S3.** Compound 11D $^{1}$H NMR (800 MHz, $CD_2Cl_2$, 25 °C, top) and $^{13}$C NMR (201 MHz, $CD_2Cl_2$, 25 °C, bottom).

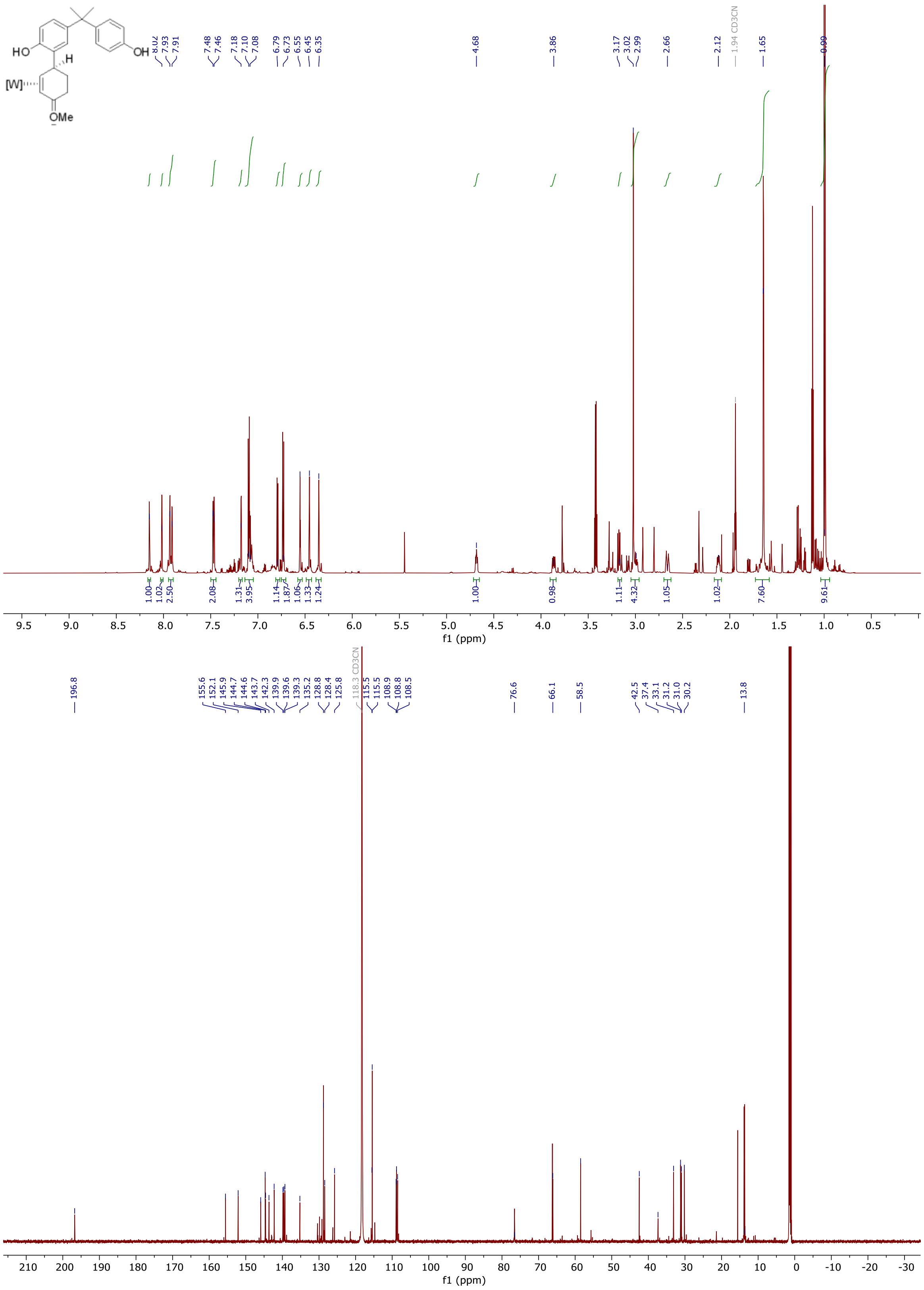

**Fig. S4.** Compound 12D $^{1}H$ NMR (800 MHz, $CD_3CN$, 25 °C, top) and $^{13}C$ NMR (201 MHz, $CD_3CN$, 25 °C, bottom).

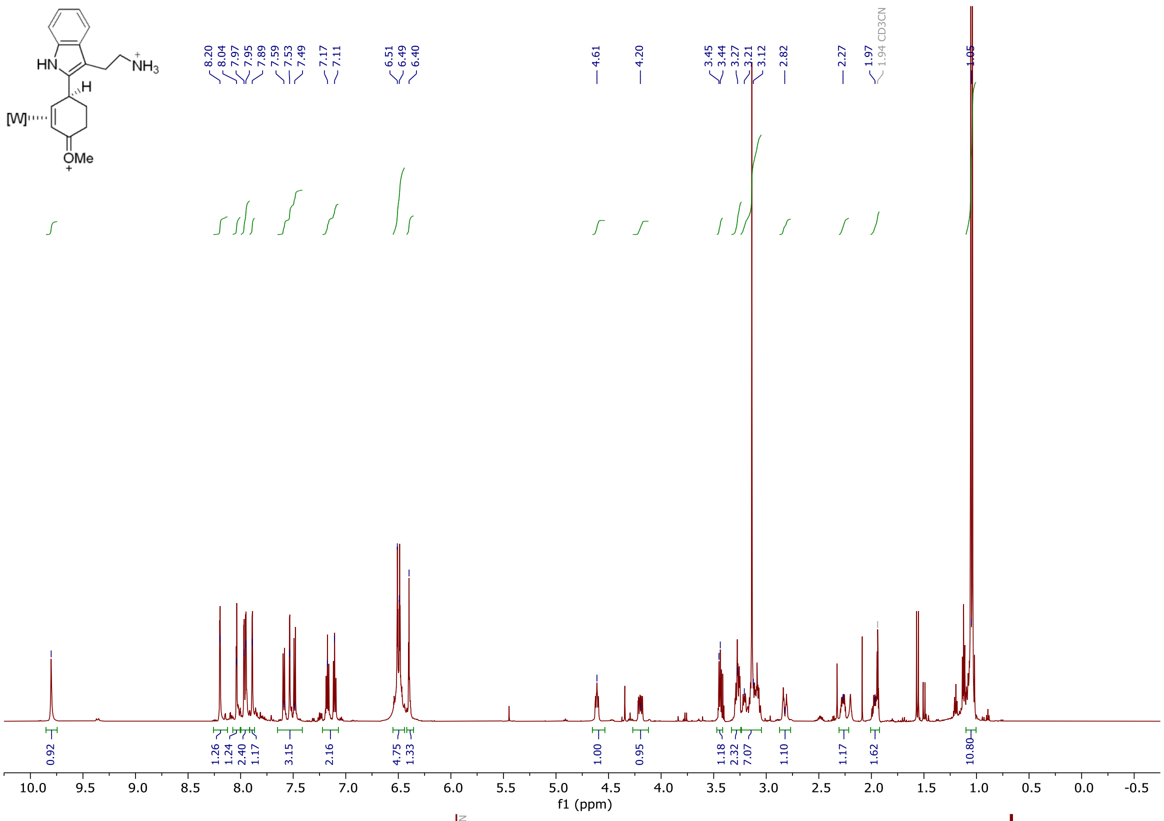

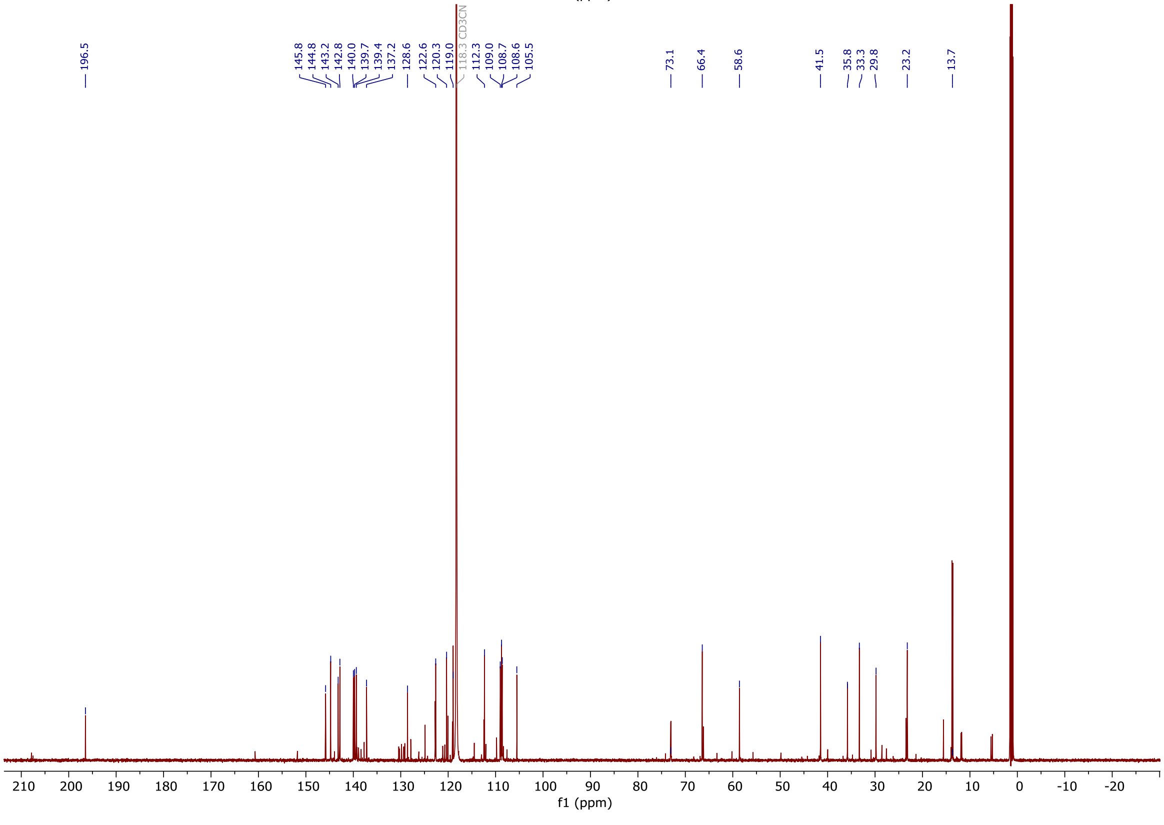

**Fig**. **S5.** Compound 14D $^{1}H$ NMR (600 MHz, $CD_3CN$, 25 °C, top) and $^{13}C$ NMR (201 MHz, $CD_3CN$, 25 °C, bottom).

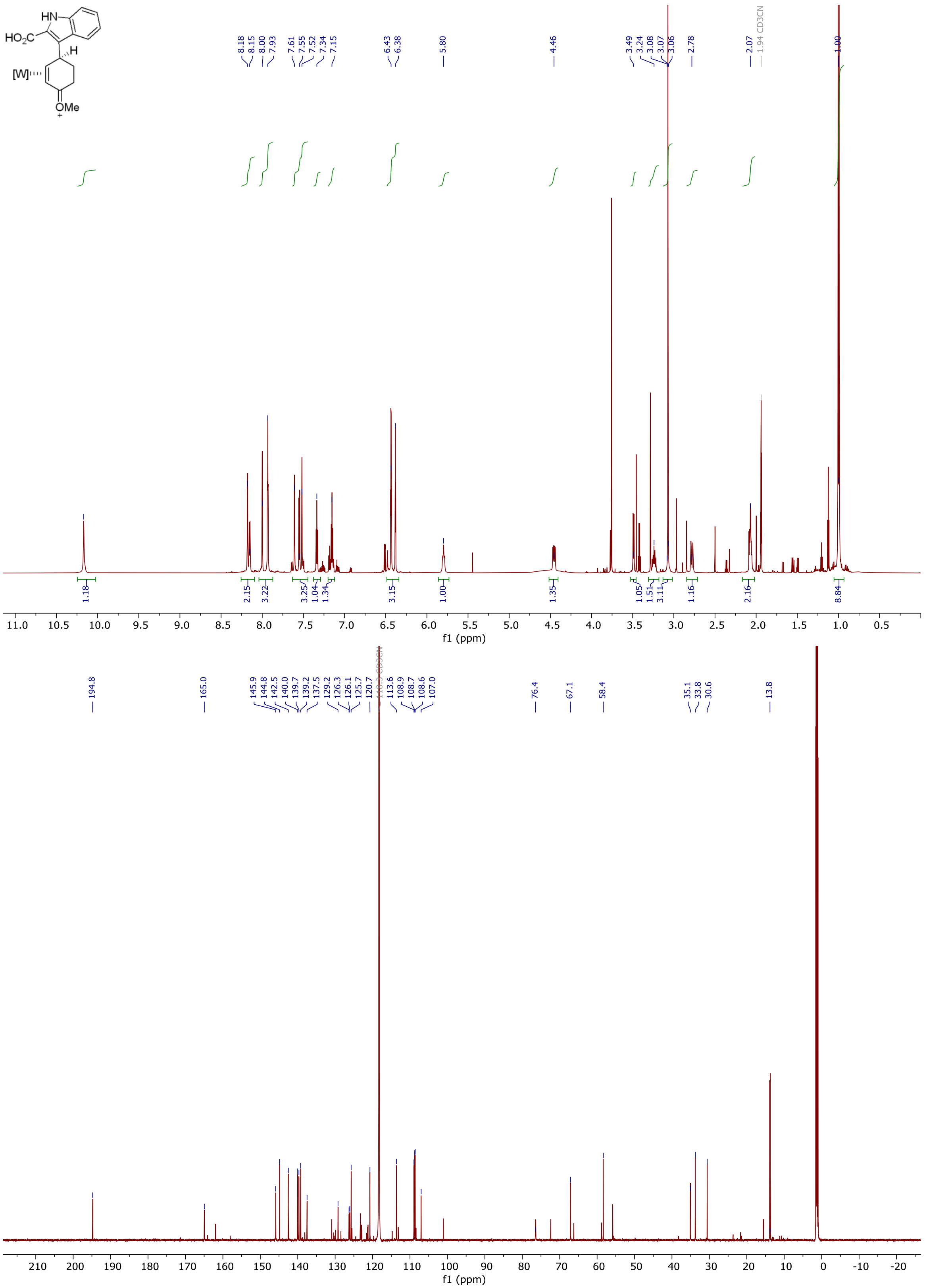


**Fig**. **S6.** Compound 16D $^{1}H$ NMR (800 MHz, $CD_3CN$, 25 °C, top) and $^{13}C$ NMR (201 MHz, $CD_3CN$, 25 °C, bottom).

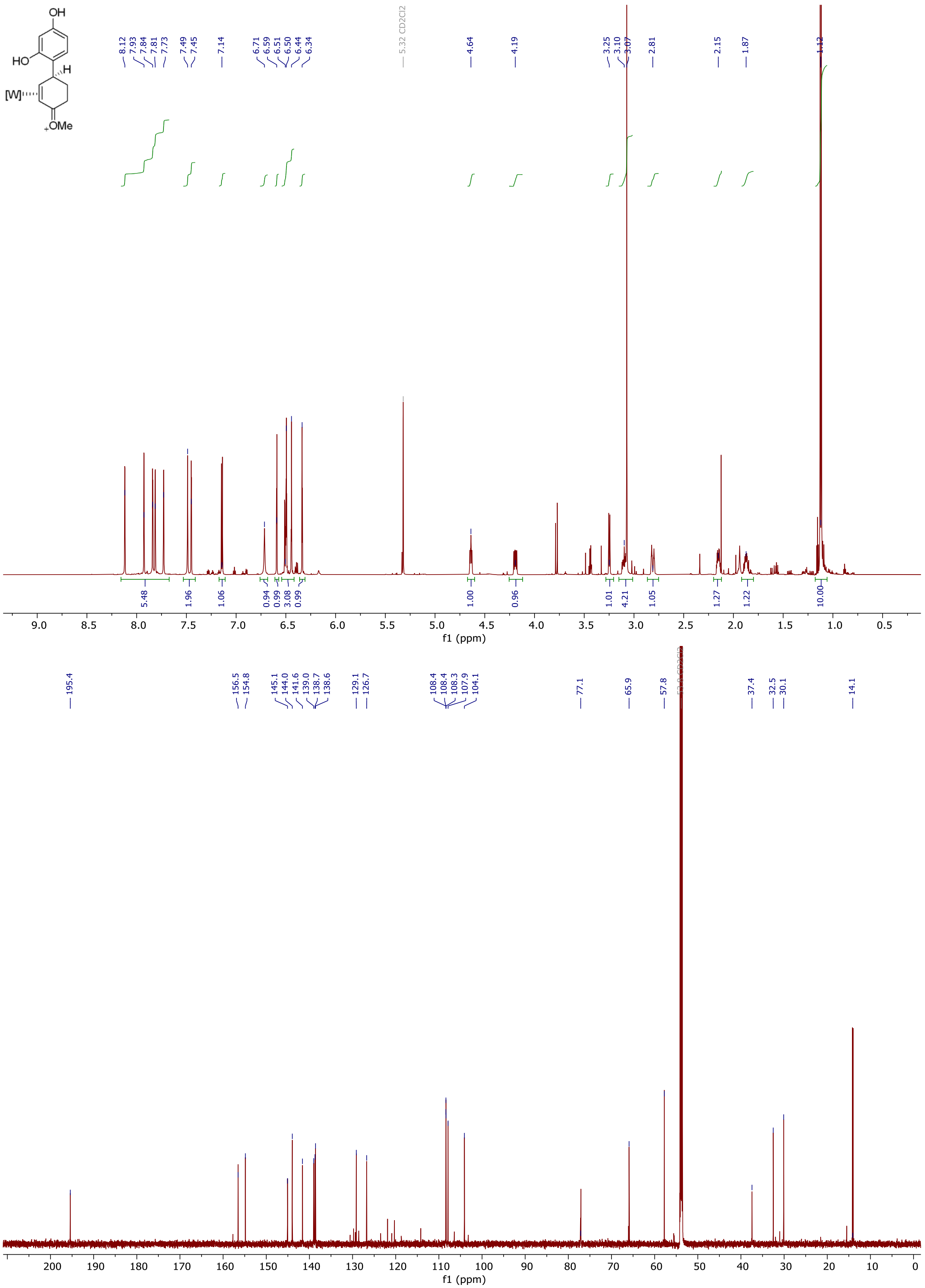


**Fig. S7.** Compound 17D $^{1}H$ NMR (800 MHz, $CD_2Cl_2$, 25 °C, top) and $^{13}C$ NMR (201 MHz, $CD_2Cl_2$, 25 °C, bottom).

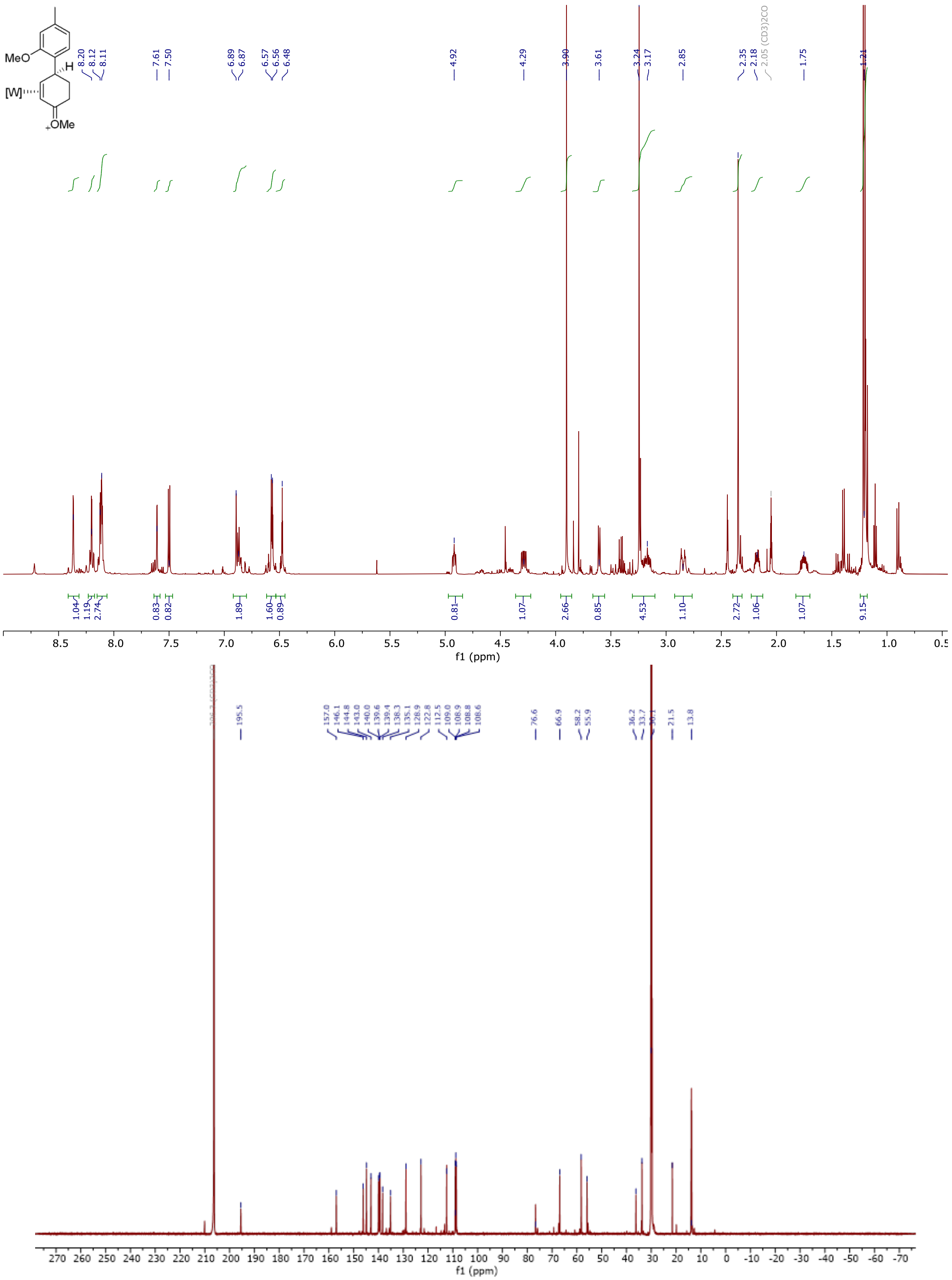


**Fig**. **S8.** Compound 18D $^{1}H$ NMR (600 MHz, $(CD_3)_2CO$, 25 °C, top) and $^{13}C$ NMR (201 MHz, $(CD_3)_2CO$, 25 °C, bottom).

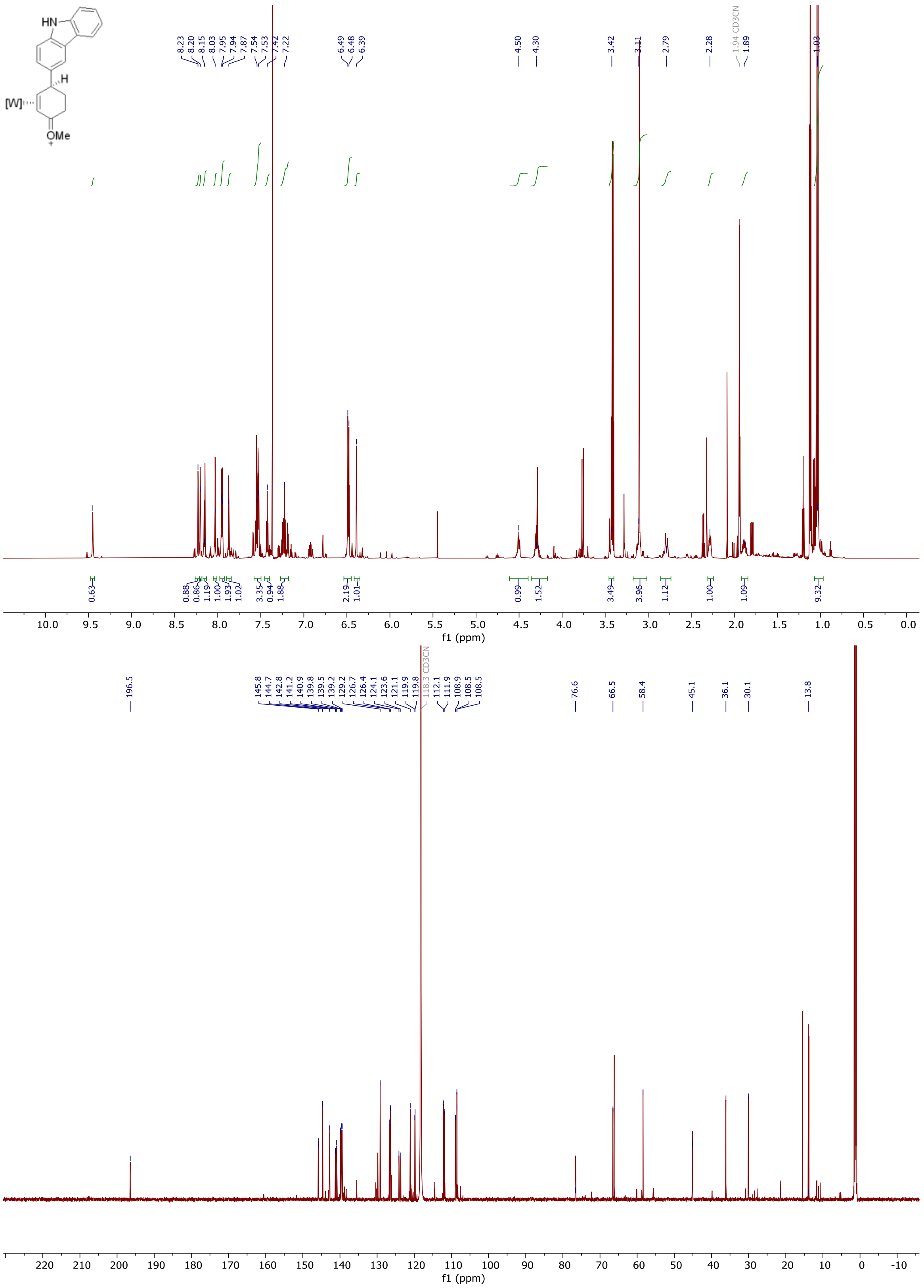


**Fig**. **S9.** Compound 77D $^{1}H$ NMR (800 MHz, $CD_3CN$, 15 °C, top) and $^{13}C$ NMR (201 MHz, $CD_3CN$, 15 °C, bottom).

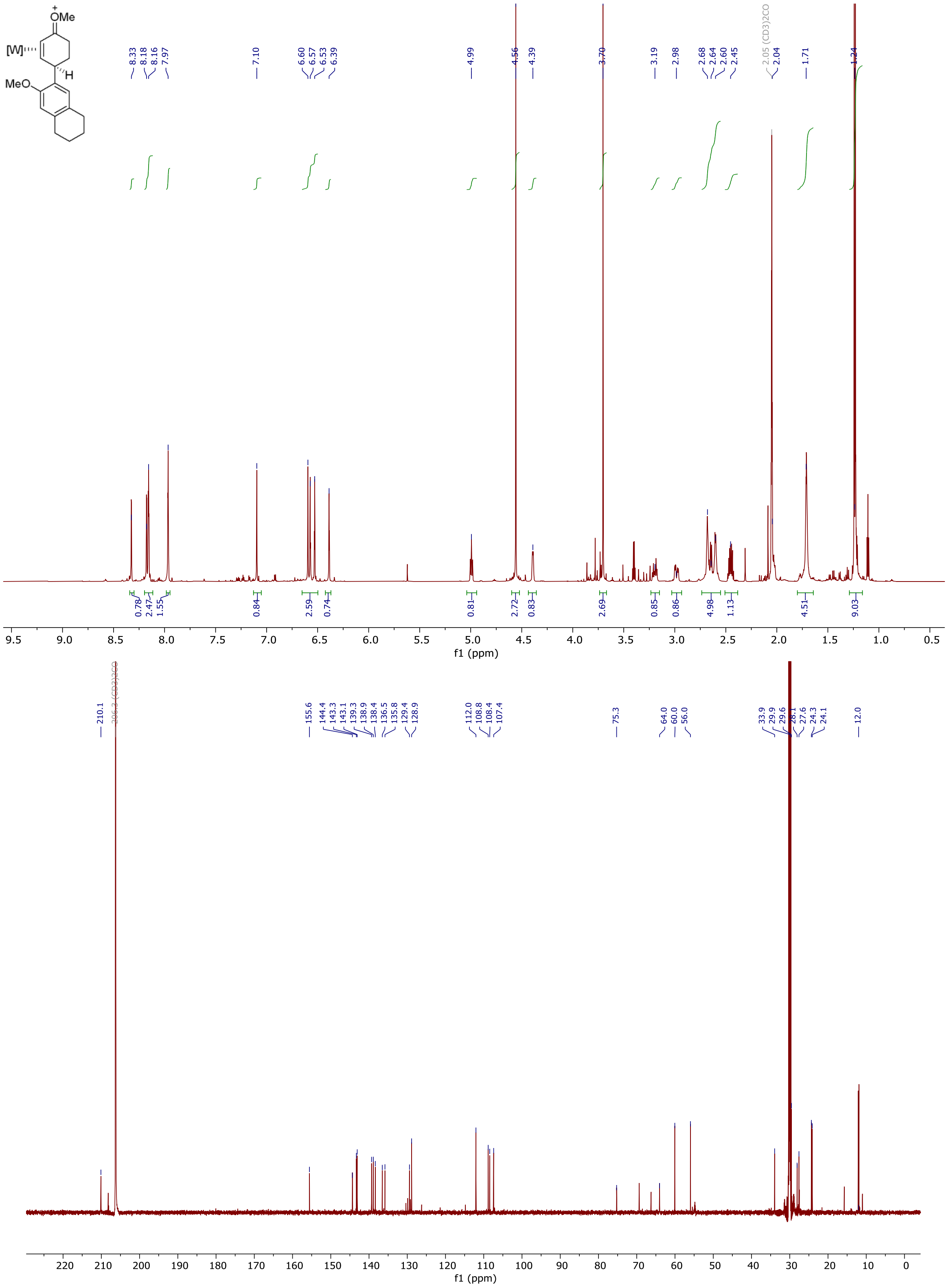


**Fig**. **S10.** Compound 5P $^{1}H$ NMR (800 MHz, $(CD_3)_2CO$, 25 °C, top) and $^{13}C$ NMR (201 MHz, $(CD_3)_2CO$, 25 °C, bottom).

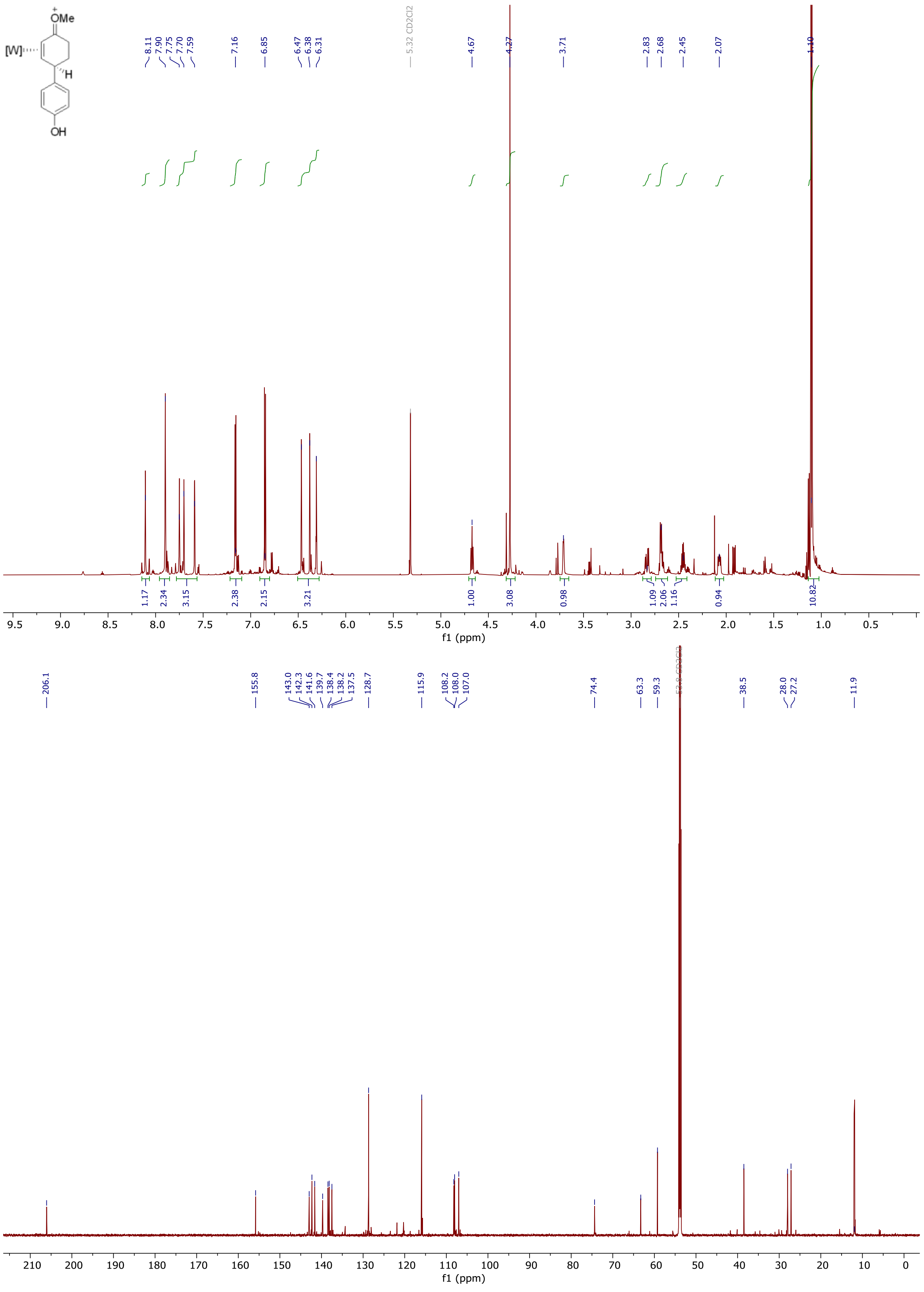


**Fig**. **S11.** Compound 6P $^1$H NMR (800 MHz, $CD_2Cl_2$, 25 °C, top) and $^{13}$C NMR (201 MHz, $CD_2Cl_2$, 25 °C, bottom).

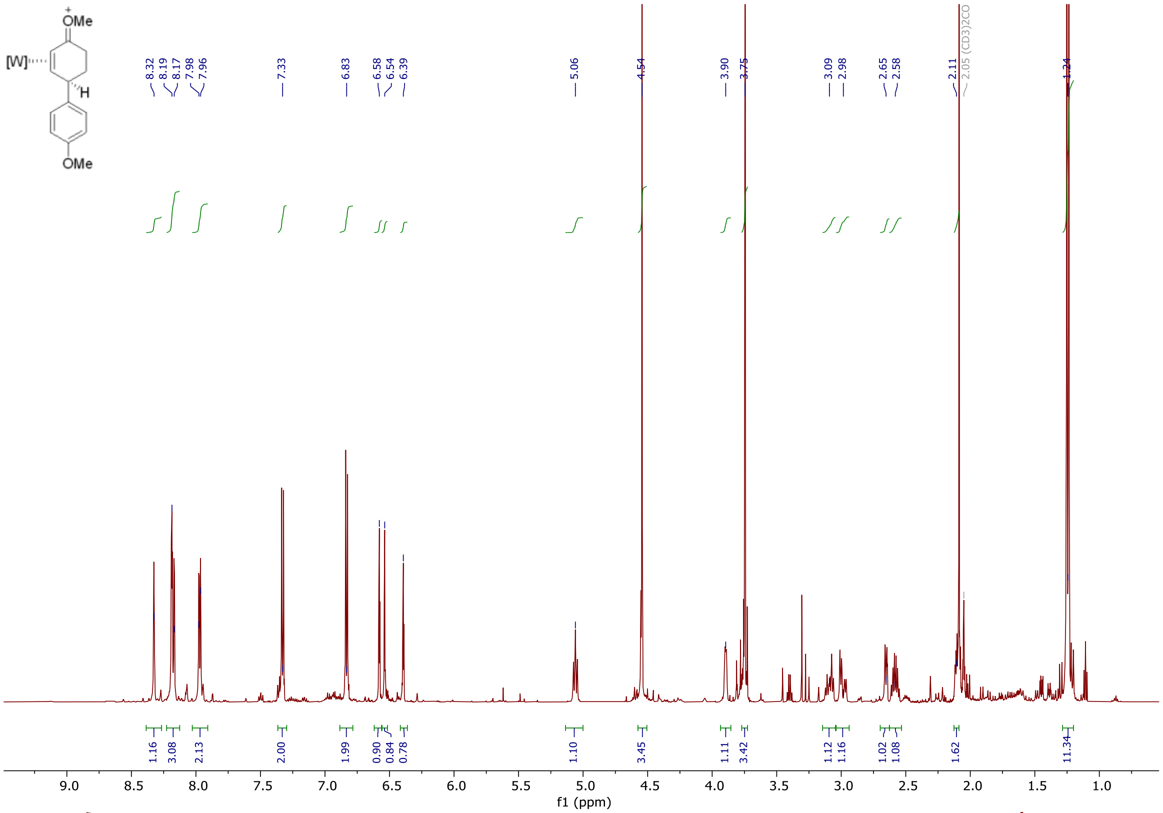

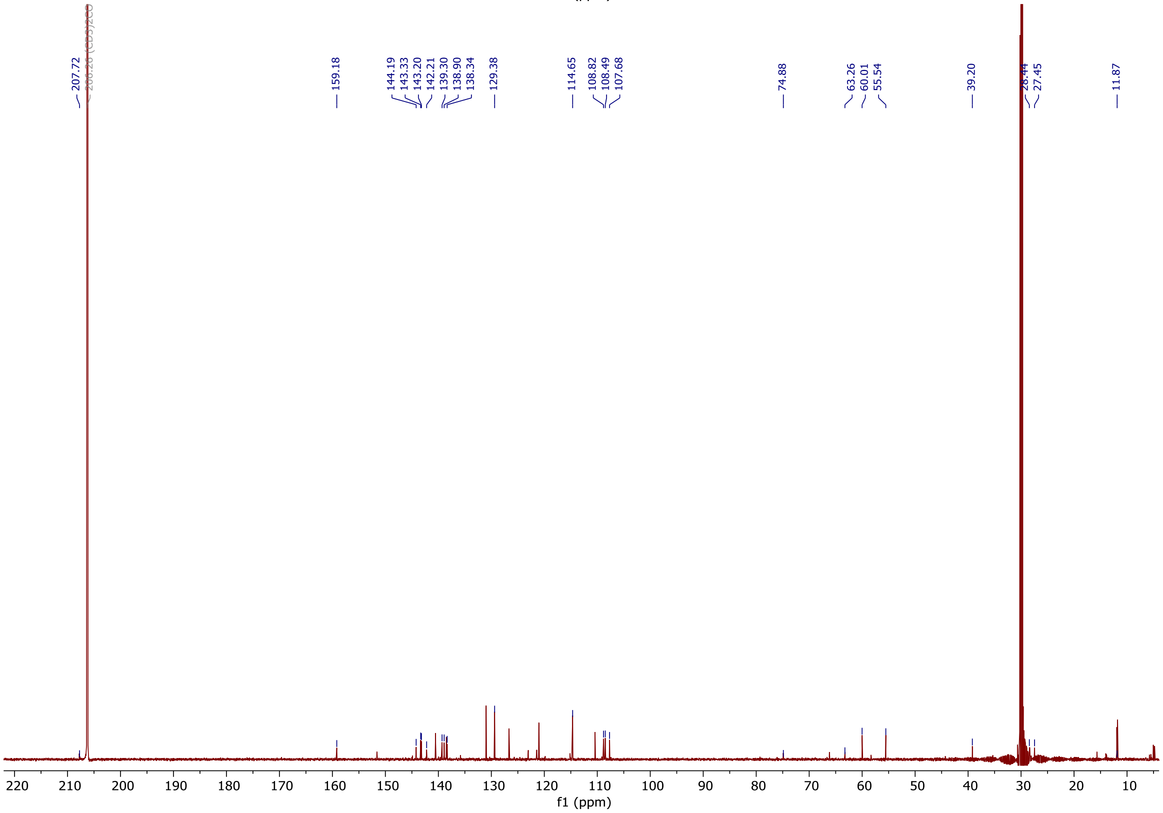

**Fig. S12.** Compound 7P $^{1}H$ NMR (600 MHz, $(CD_3)_2CO$, 25 °C, top) and $^{13}C$ NMR (201 MHz, $(CD_3)_2CO$, 25 °C, bottom).

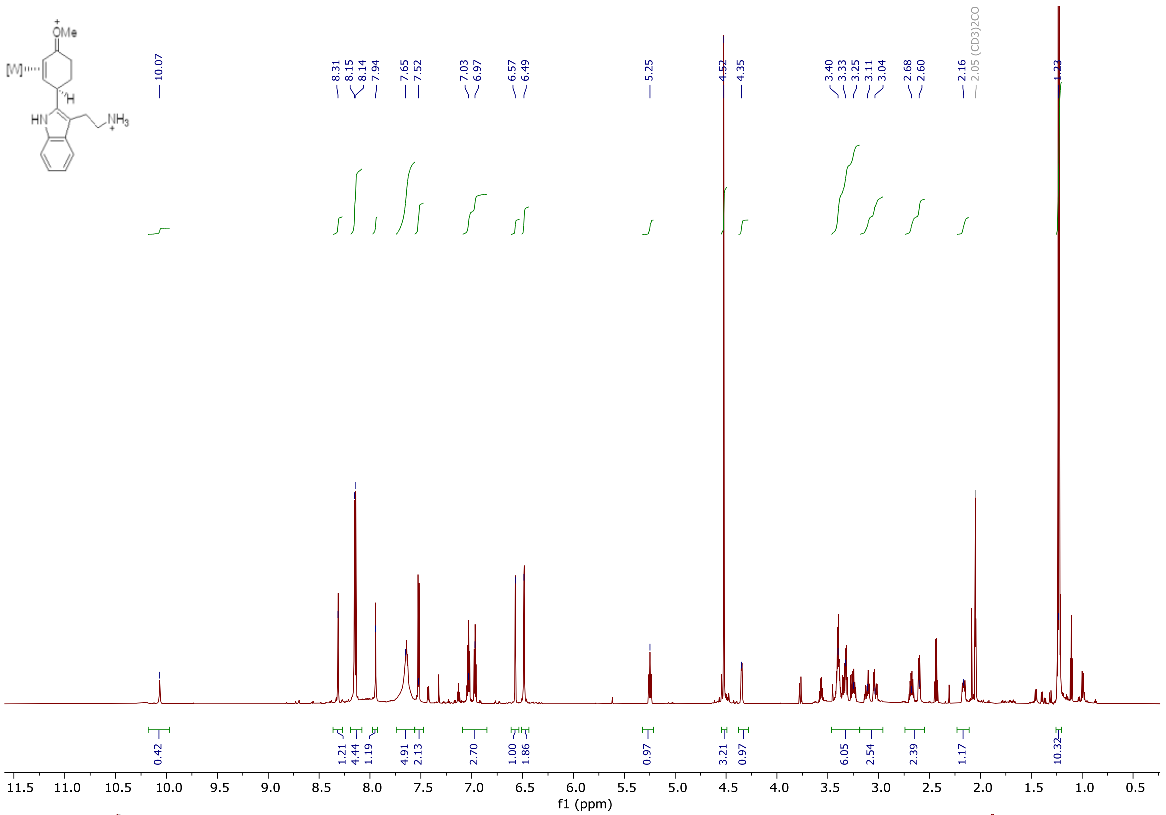


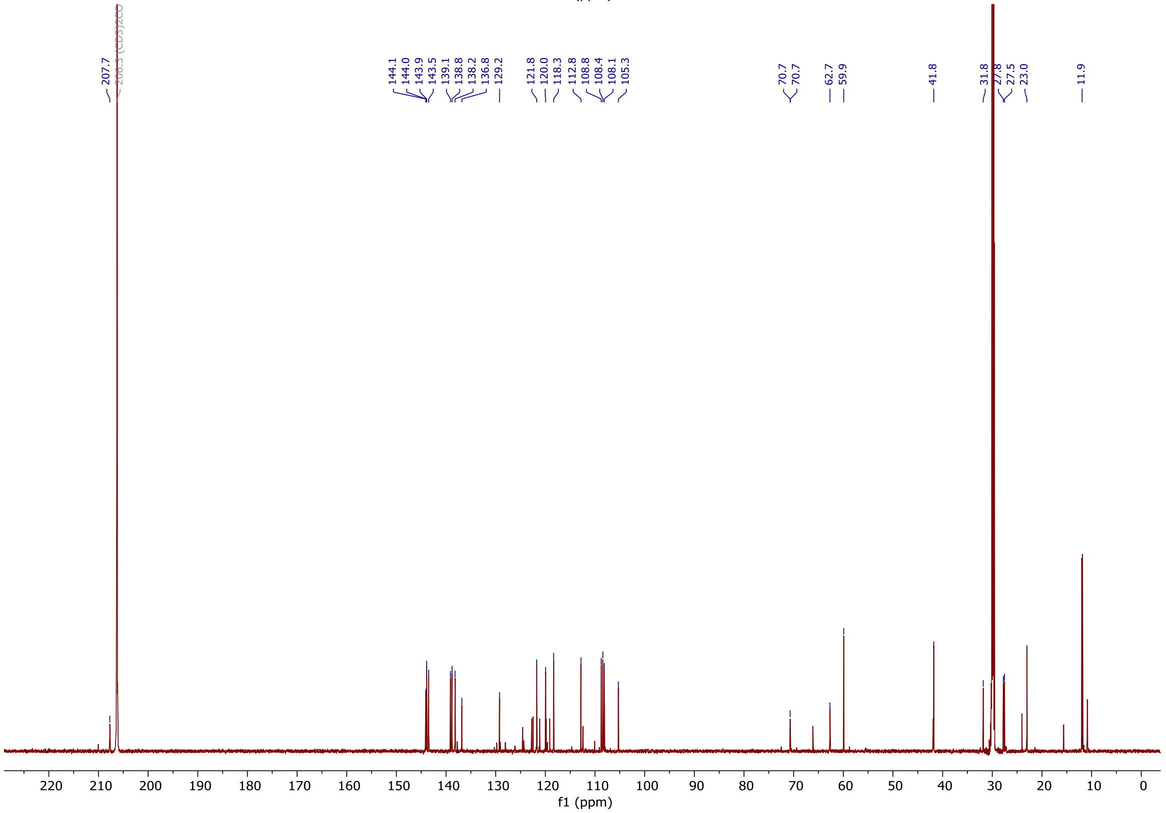


**Fig**. **S13.** Compound 14P $^{1}$H NMR (800 MHz, $(CD_3)_2CO$, 25 °C, top) and $^{13}$C NMR (201 MHz, $(CD_3)_2CO$, 25 °C, bottom).

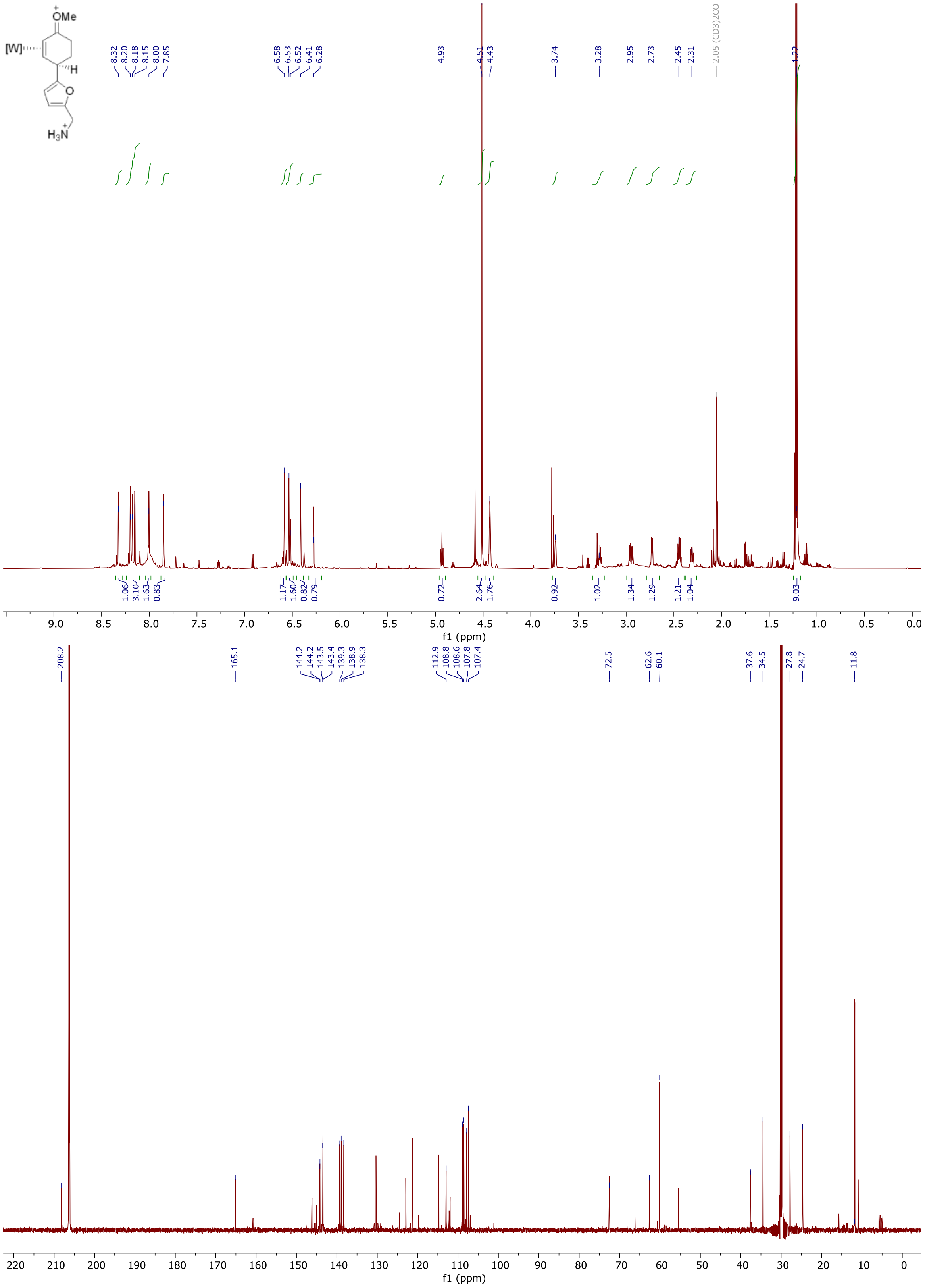


**Fig. S14.** Compound 15P $^{1}H$ NMR (800 MHz, $(CD_3)_2CO$, 25 °C, top) and $^{13}C$ NMR (201 MHz, $(CD_3)_2CO$, 25 °C, bottom).

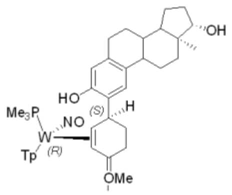

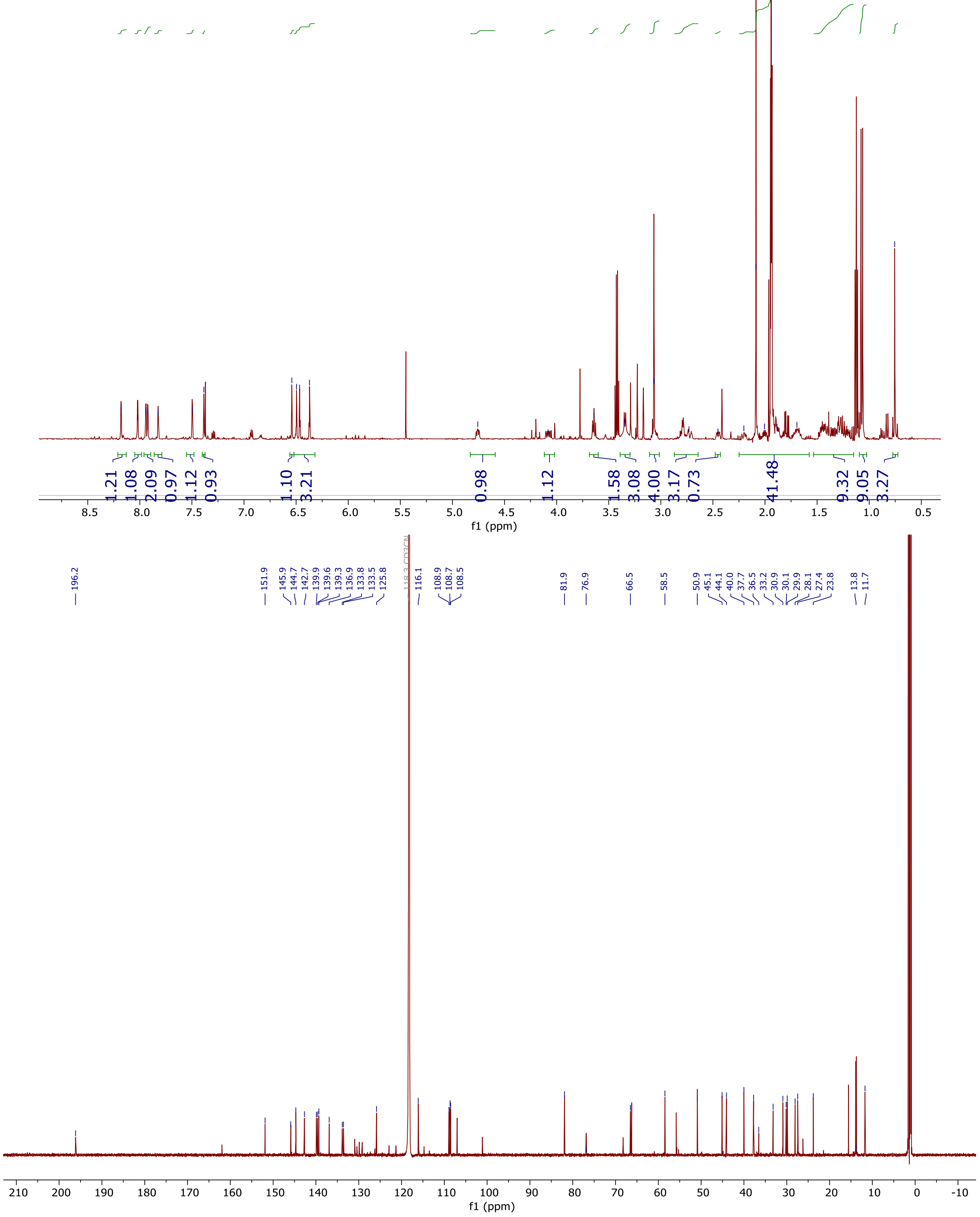

**Fig**. **S15.** Compound *(WR, 4S, 9'S)*-20D $^{1}H$ NMR (600 MHz, $CD_3CN$, 25 °C, top) and $^{13}C$ NMR (201 MHz, $CD_3CN$, 25 °C, bottom).

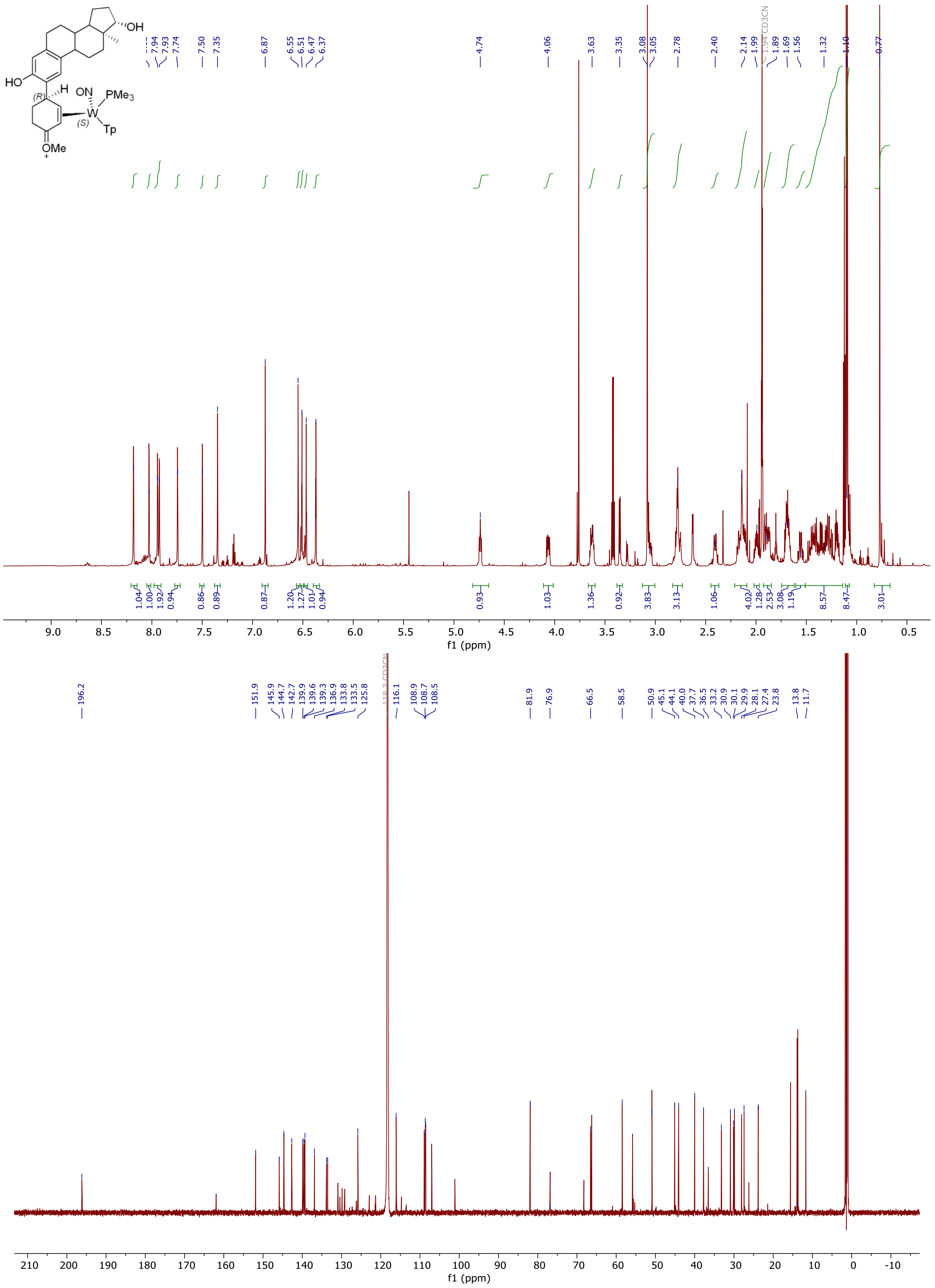


**Fig**. **S16.** Compound *(WS, 4R, 9'S)*-20D $^{1}H$ NMR (800 MHz, $CD_3CN$, 25 °C, top) and $^{13}C$ NMR (201 MHz, $CD_3CN$, 25 °C, bottom).

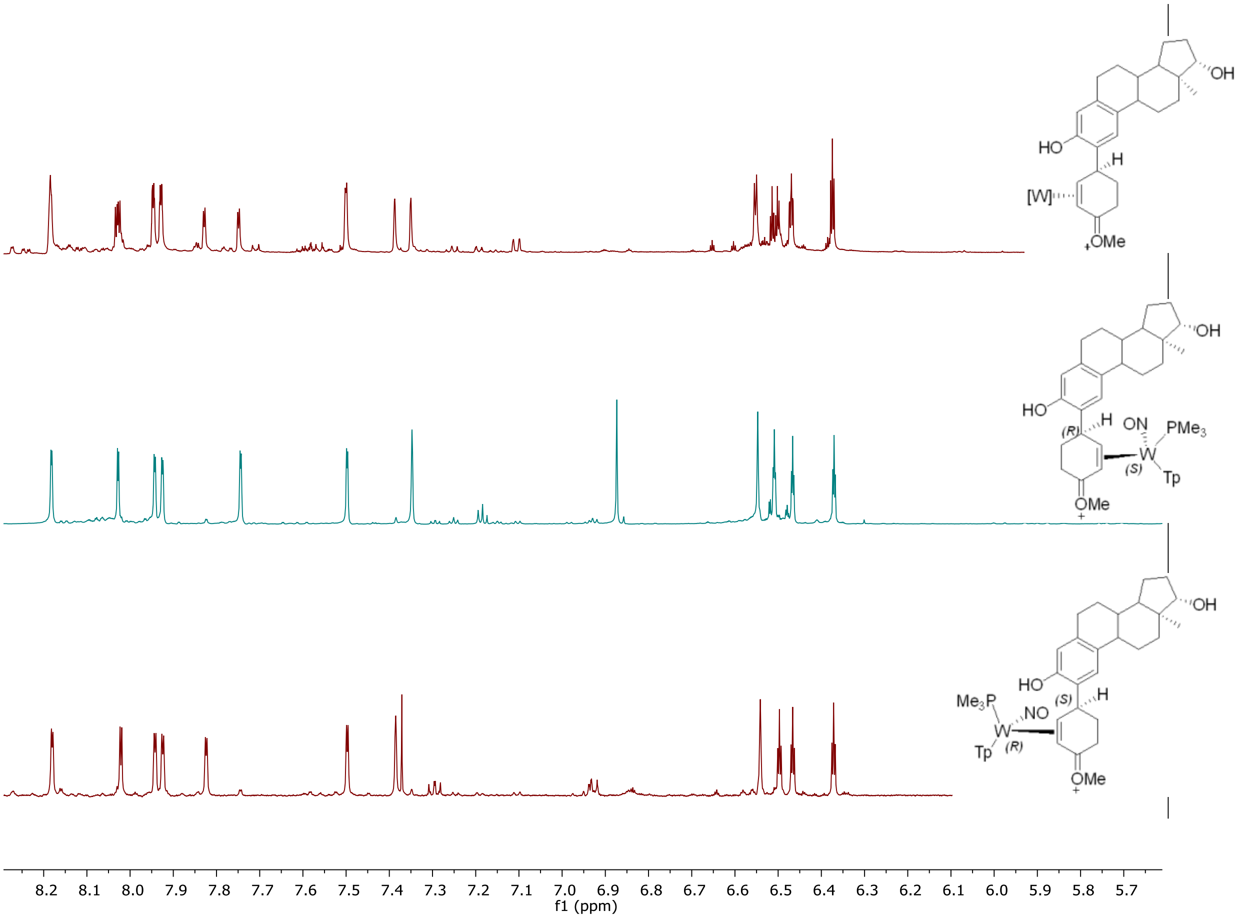


**Fig**. **S17.** Compound 20D (600 MHz, $CD_3CN$, 25 °C, top)), *(WS, 4R, 9'S)*-20D (800 MHz, $CD_3CN$, 25 °C, middle), and *(WR, 4S, 9'S)*-20D (600 MHz, $CD_3CN$, 25 °C, bottom) $^1$H NMR.

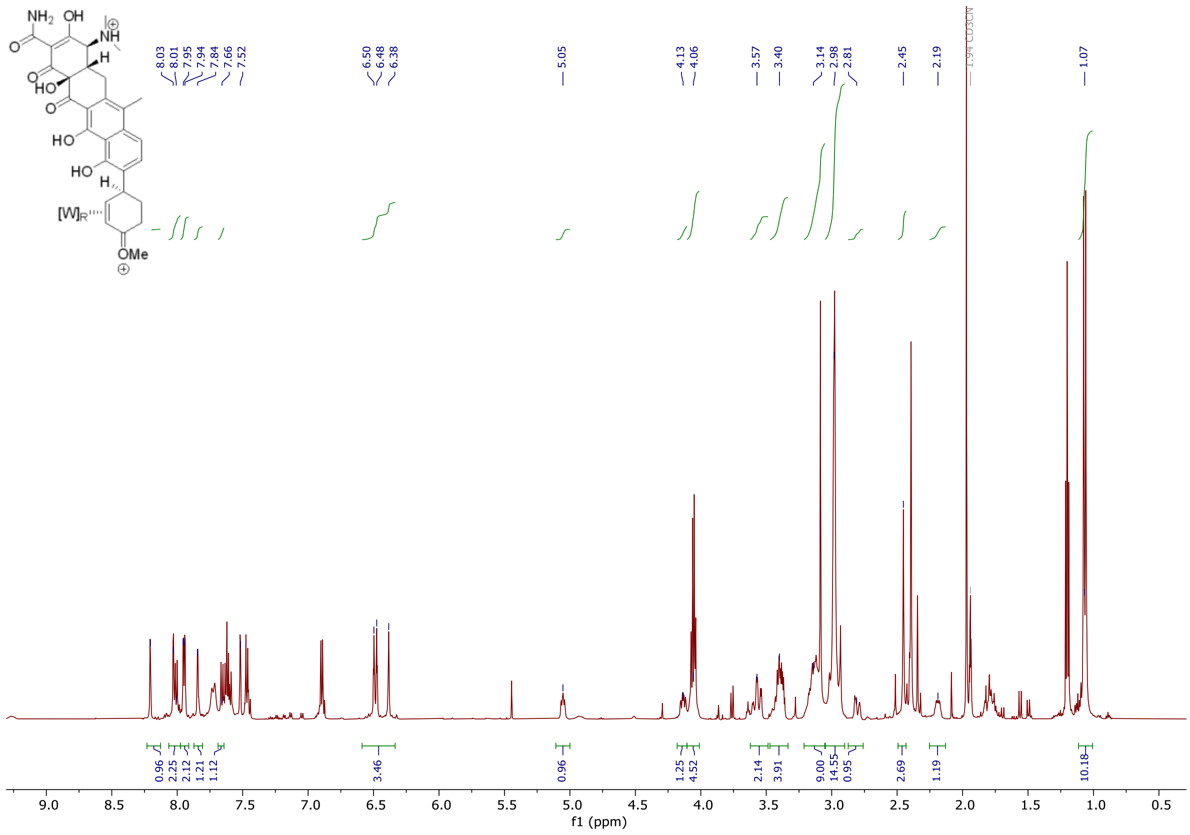

**Fig**. **S18.** Compound *(WR, 4S, 5a'S)*-21D $^{1}H$ NMR (600 MHz, $CD_3CN$, 25 °C).

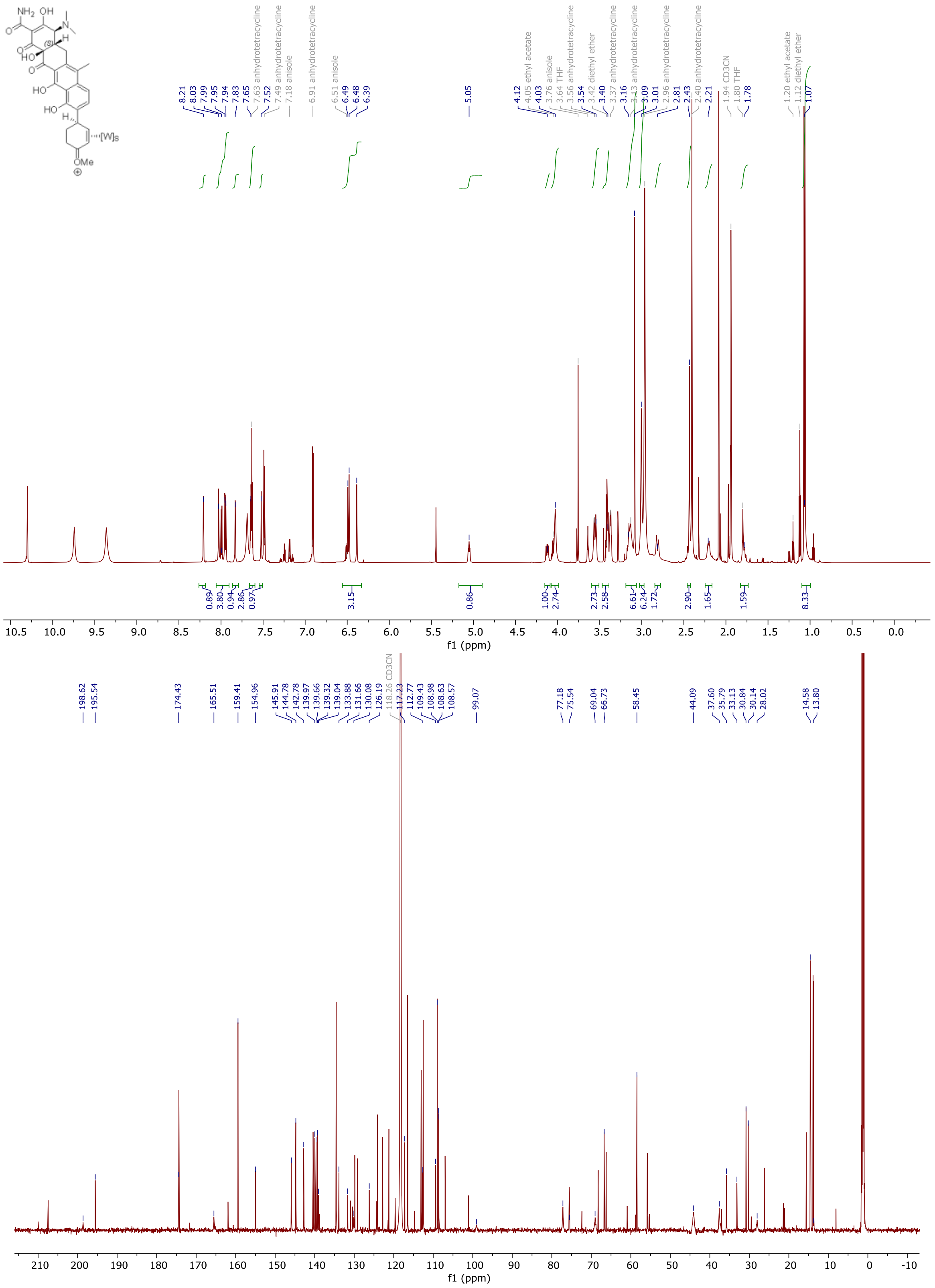


**Fig. S19.** Compound *(WS, 4R, 5a'S)*-21D $^1$H NMR (800 MHz, $CD_3CN$, 25 °C, top) and $^{13}$C NMR (201 MHz, $CD_3CN$, 25 °C, bottom).

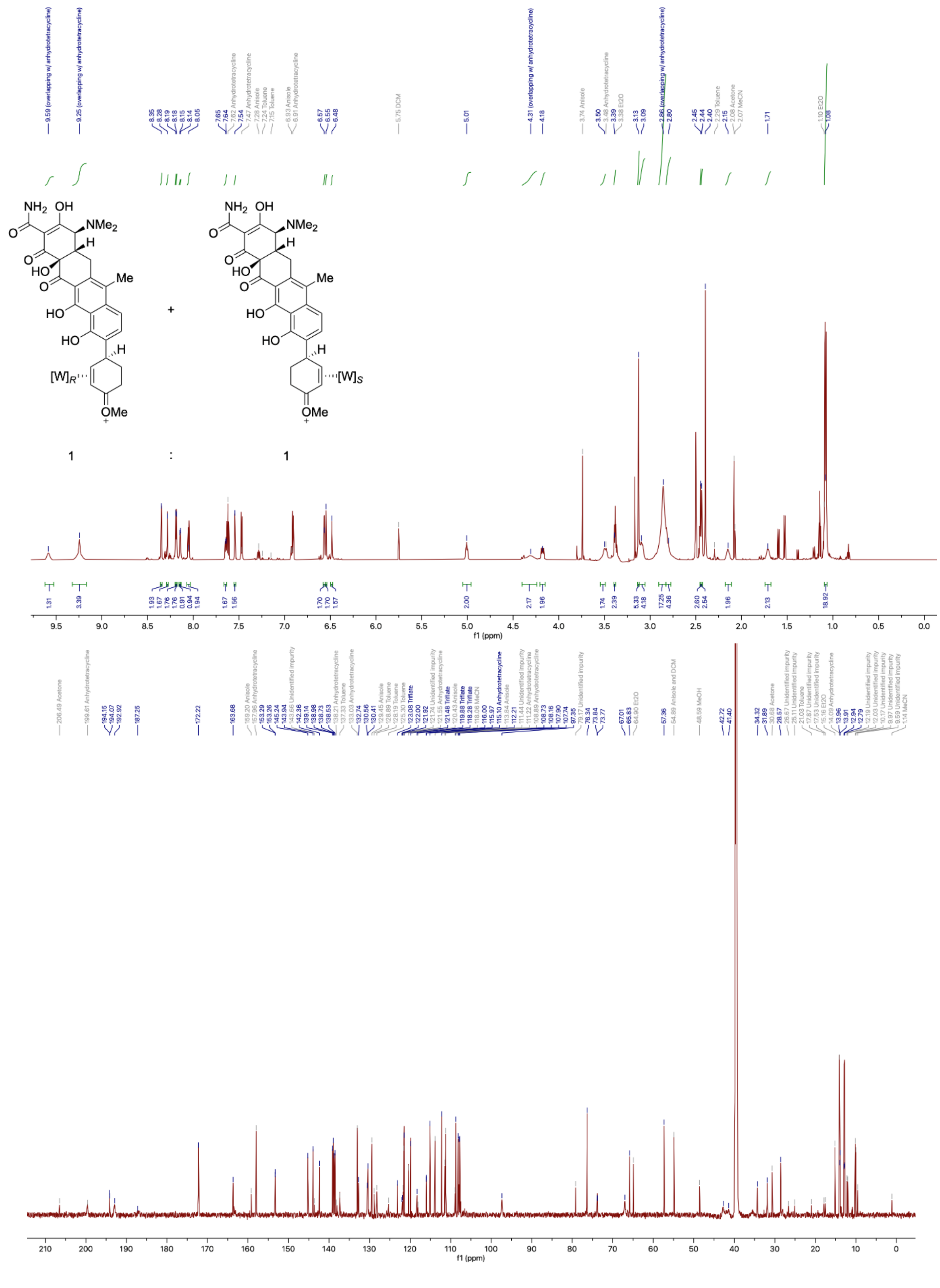

**Fig**. **S20.** Compound 21D $^{1}H$ NMR (800 MHz, $(CD_3)_2SO$, 25 °C, top) and $^{13}C$ NMR (201 MHz, $(CD_3)_2SO$, 25 °C, bottom).

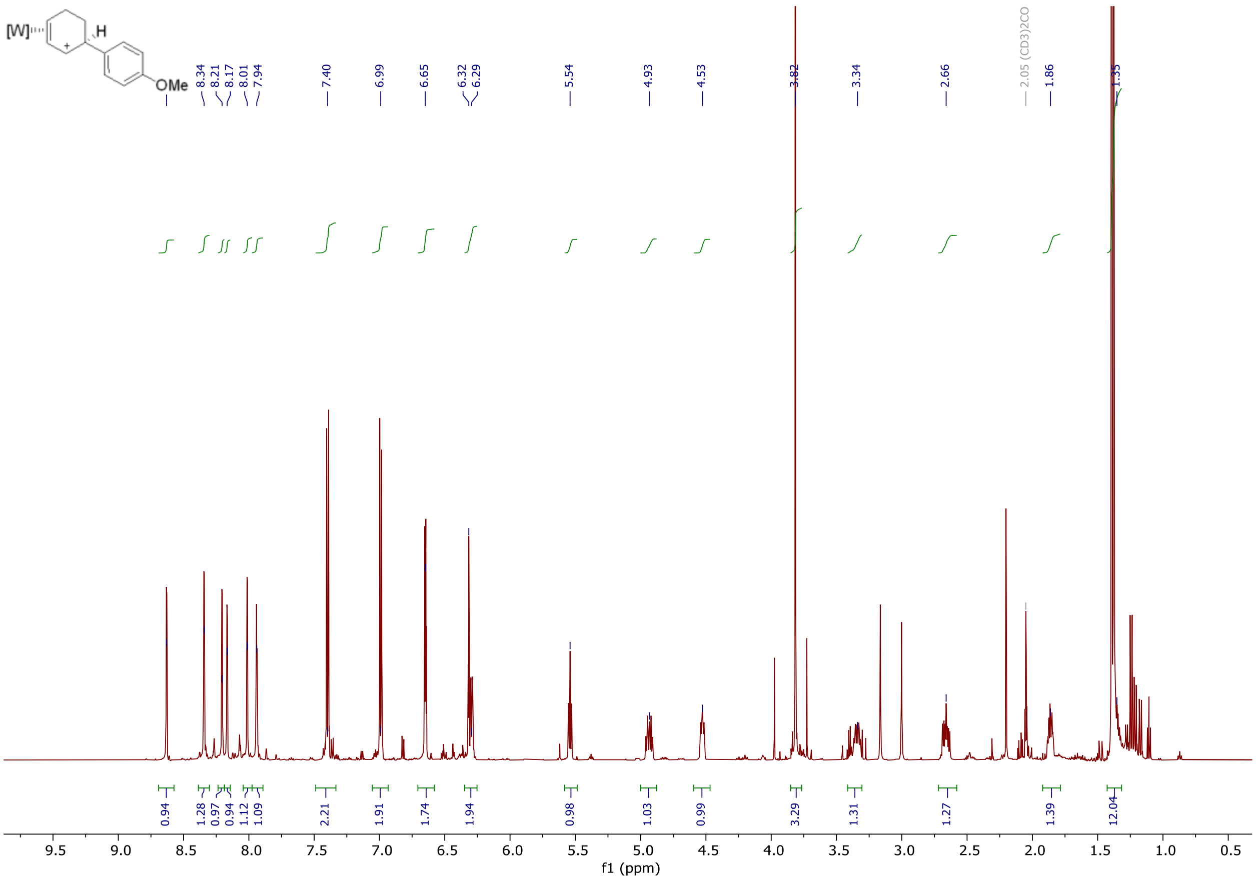


**Fig**. **S21.** Compound 27P $^{1}$H NMR (600 MHz, $(CD_3)_2CO$, 25 °C).

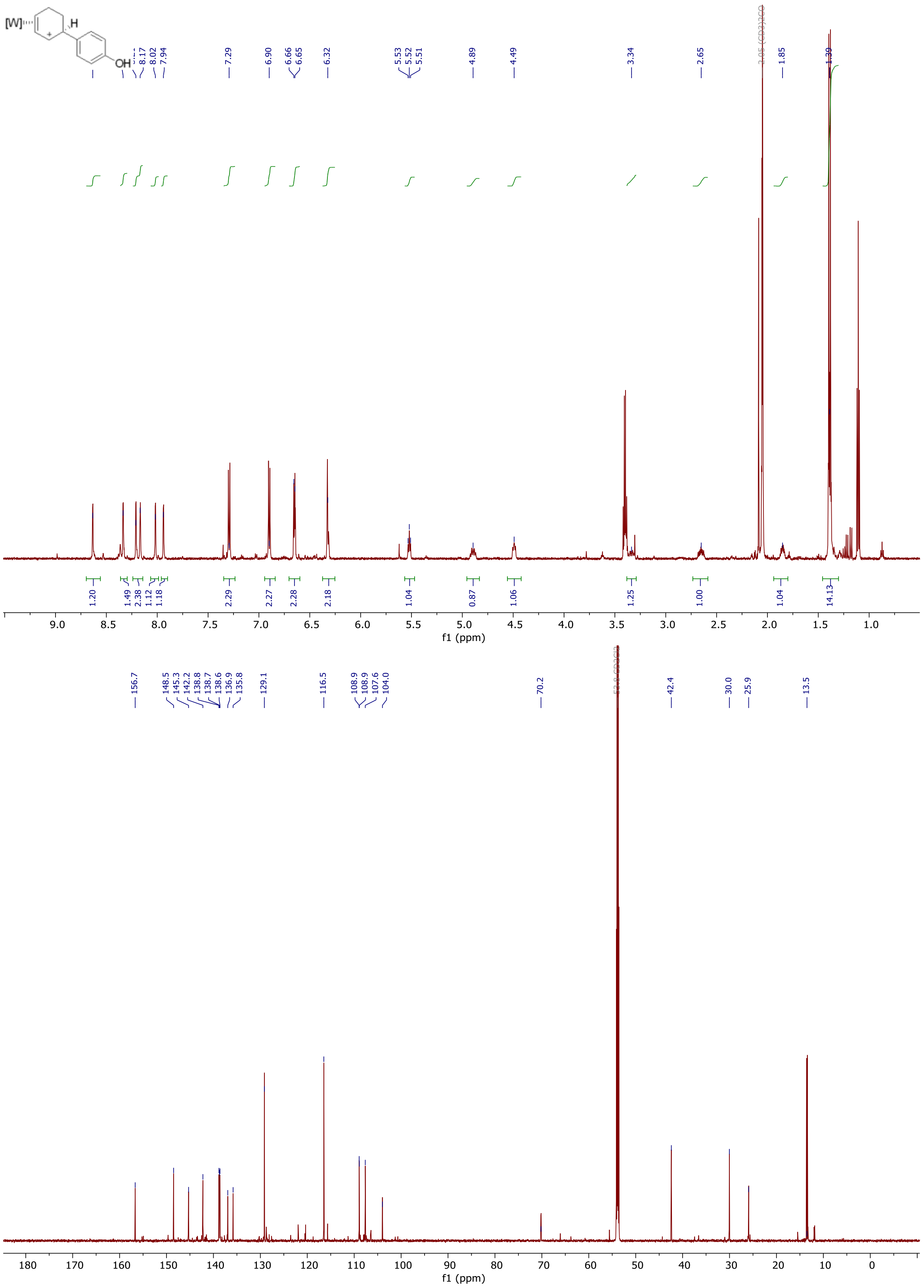


**Fig**. **S22.** Compound 28P $^1$H NMR (800 MHz, $(CD_3)_2CO$, 25 °C, top) and $^{13}$C NMR (201 MHz, $CD_2Cl_2$, 25 °C, bottom).

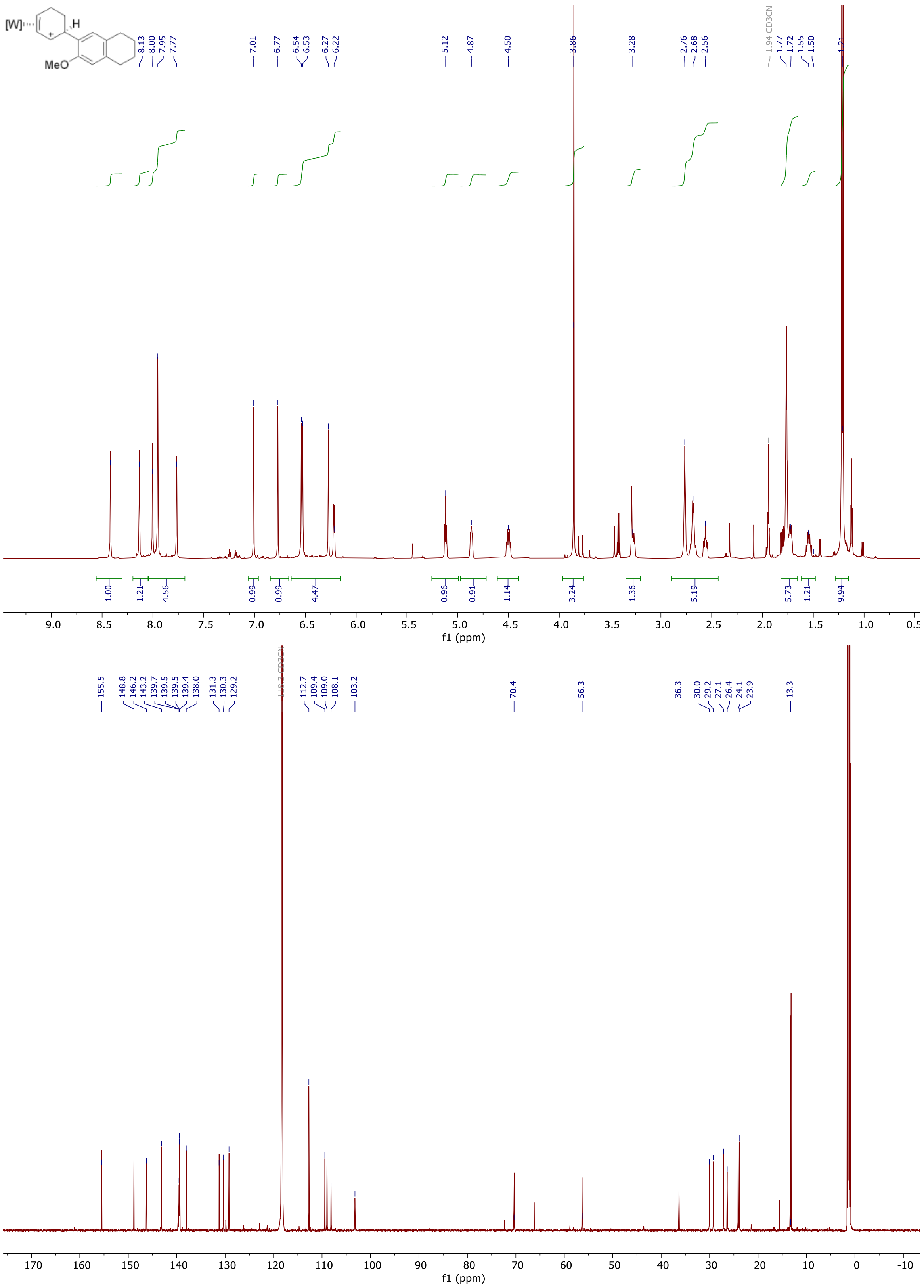

**Fig**. **S23.** Compound 30P $^{1}H$ NMR (800 MHz, $CD_3CN$, 25 °C, top) and $^{13}C$ NMR (201 MHz, $CD_3CN$, 25 °C, bottom).

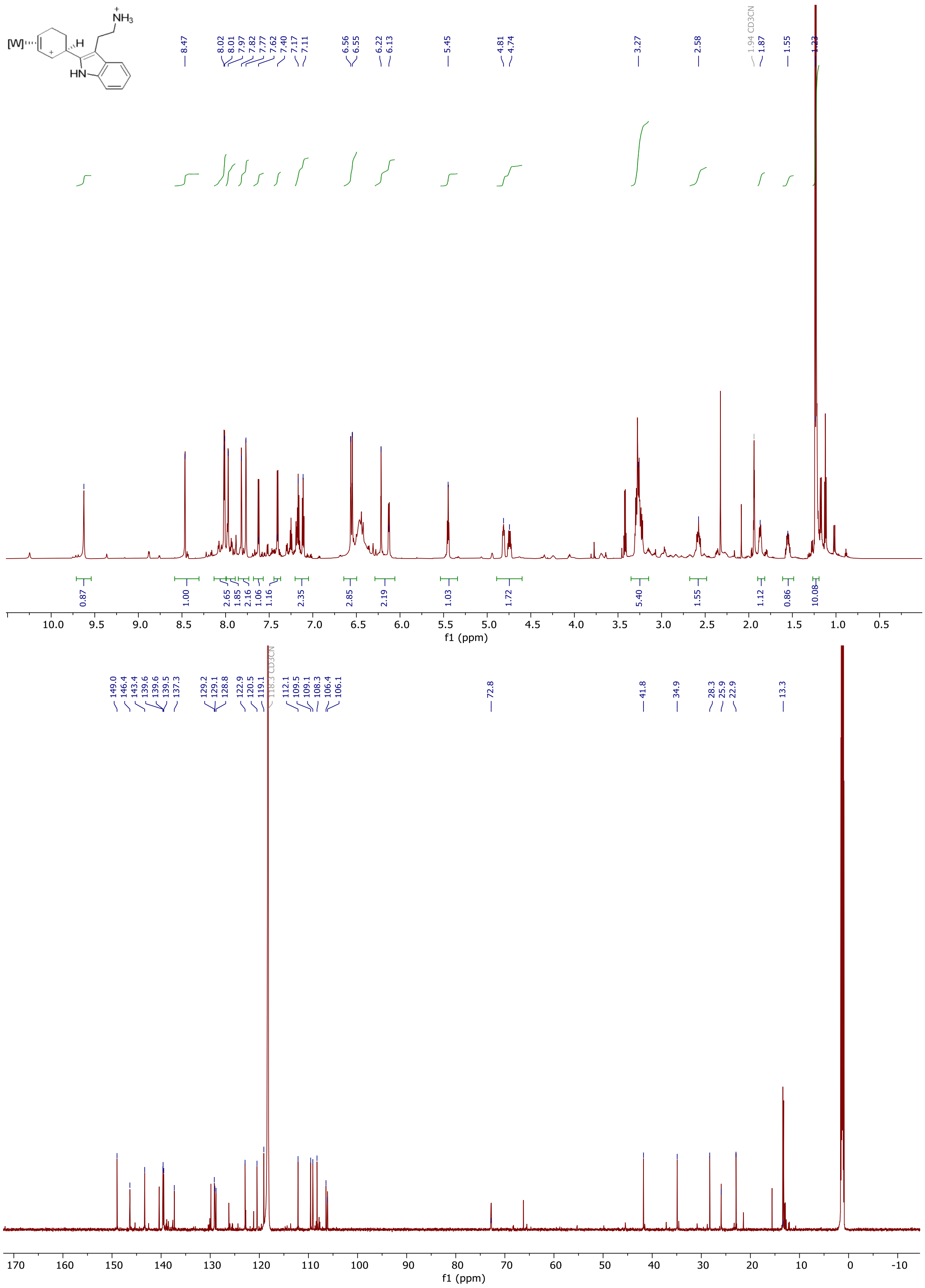


**Fig**. **S24.** Compound 31P $^1$H NMR (800 MHz, $CD_3CN$, 25 °C, top) and $^{13}$C NMR (201 MHz, $CD_3CN$, 25 °C, bottom).

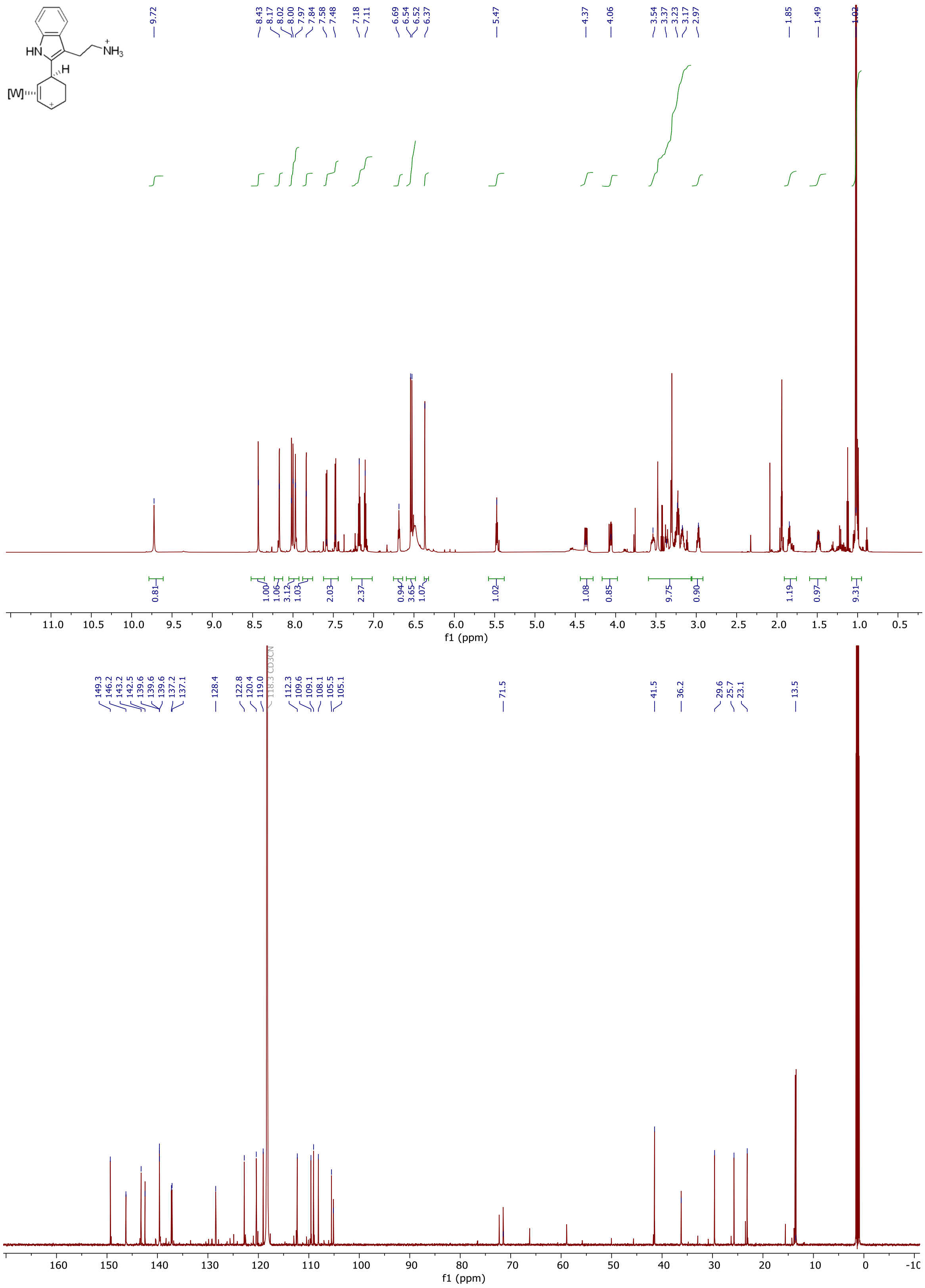


**Fig**. **S25.** Compound 31D $^{1}$H NMR (800 MHz, $CD_3CN$, 25 °C, top) and $^{13}$C NMR (201 MHz, $CD_3CN$, 25 °C, bottom).

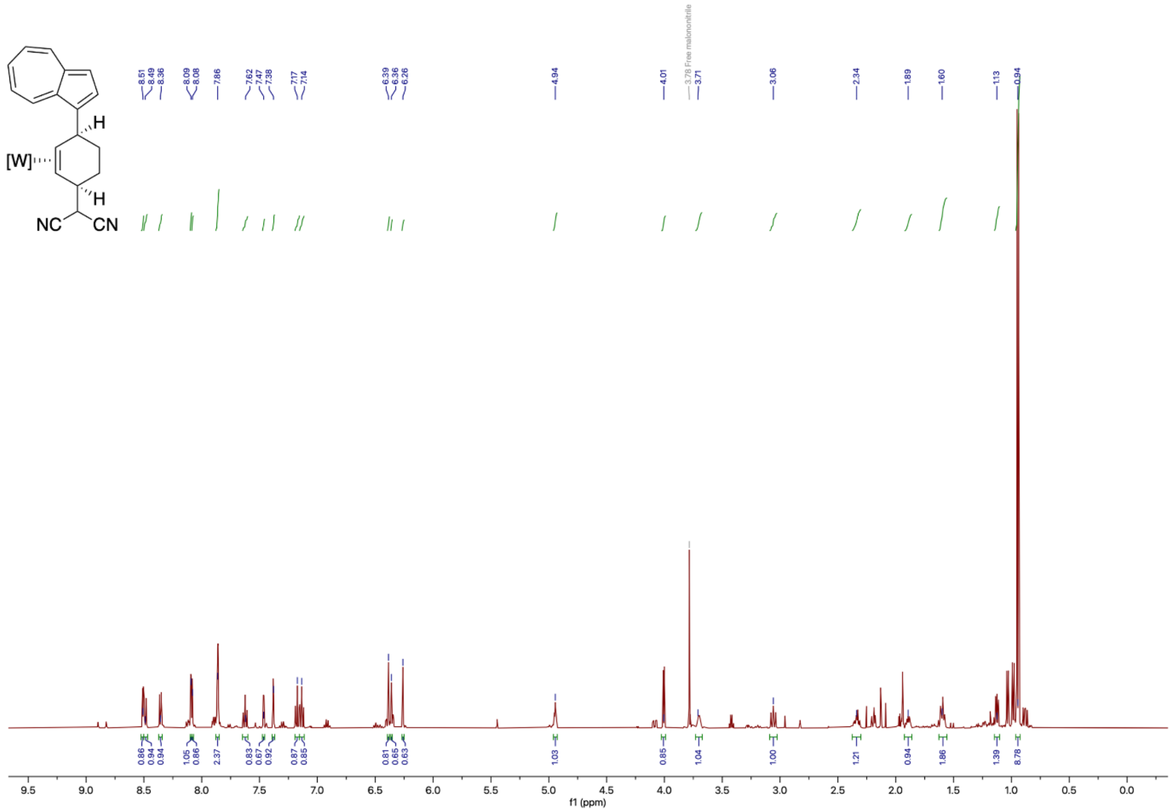

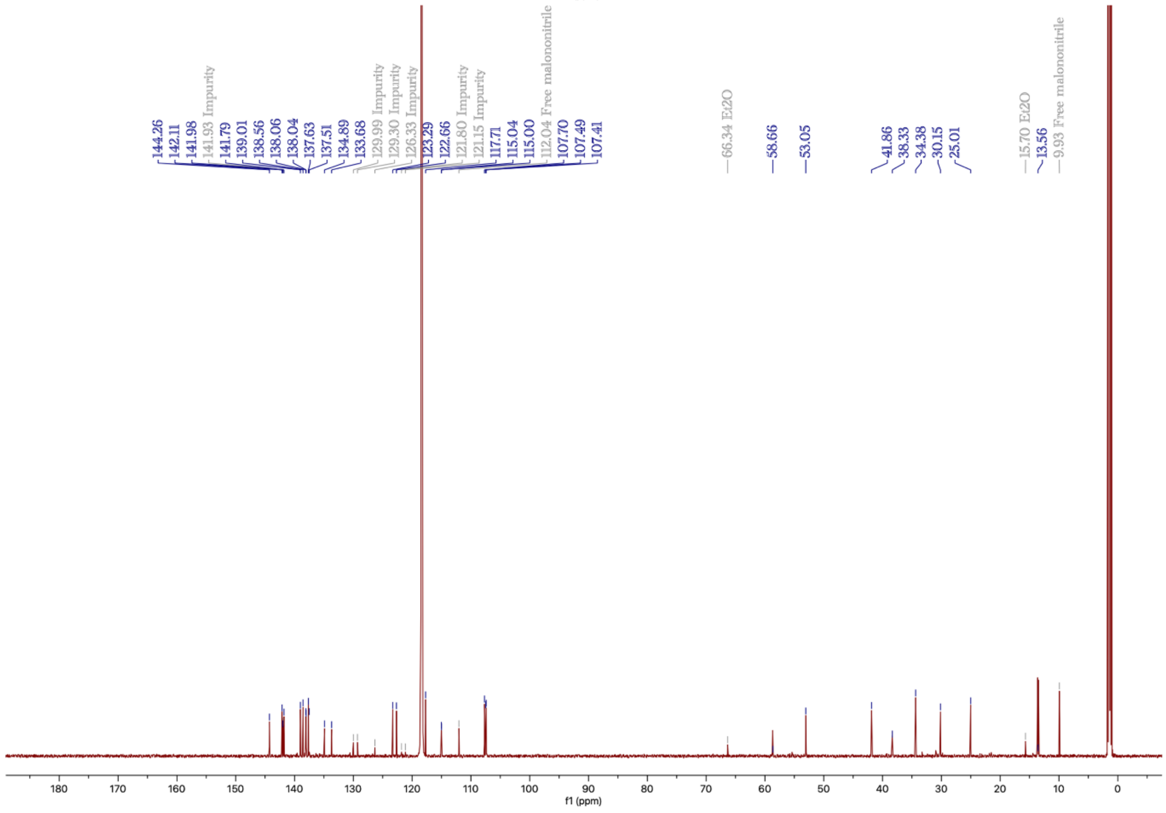

**Fig**. **S26.** Compound 39D $^{1}$H NMR (600 MHz, $CD_3CN$, 25 °C, top) and $^{13}$C NMR (201 MHz, $CD_3CN$, 25 °C, bottom).

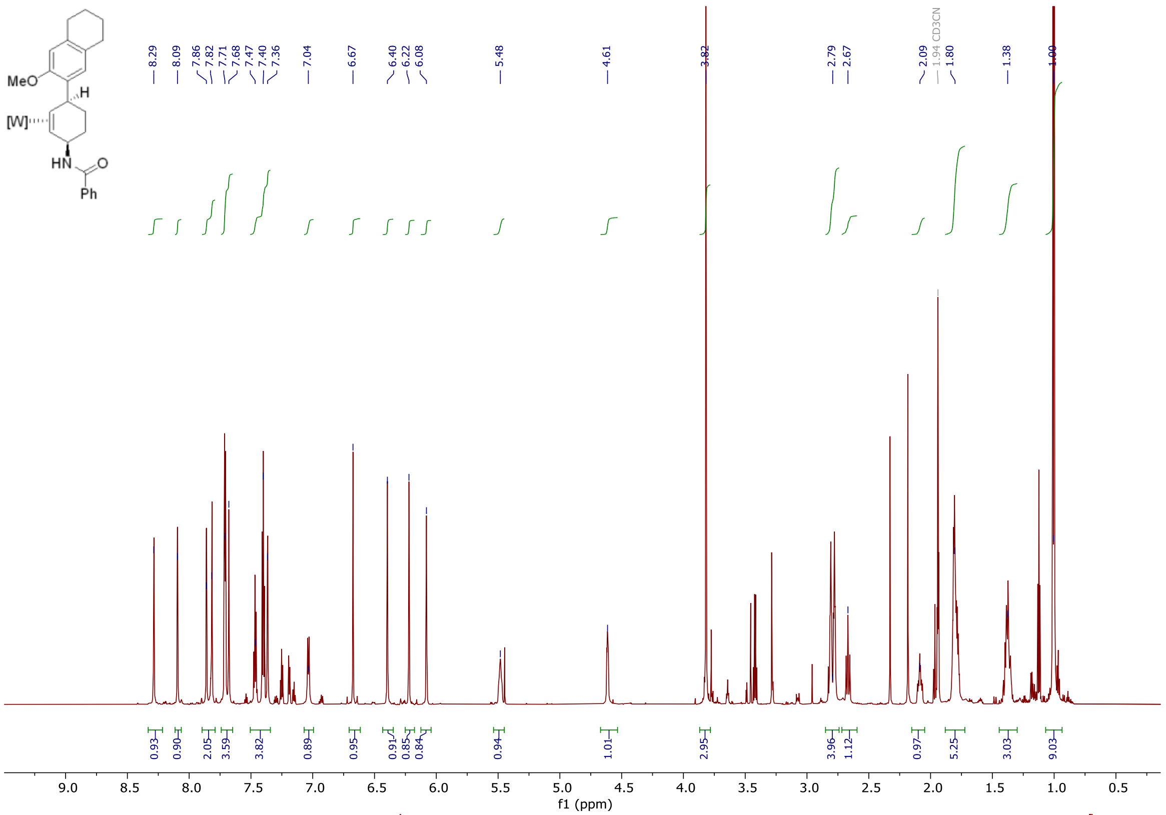

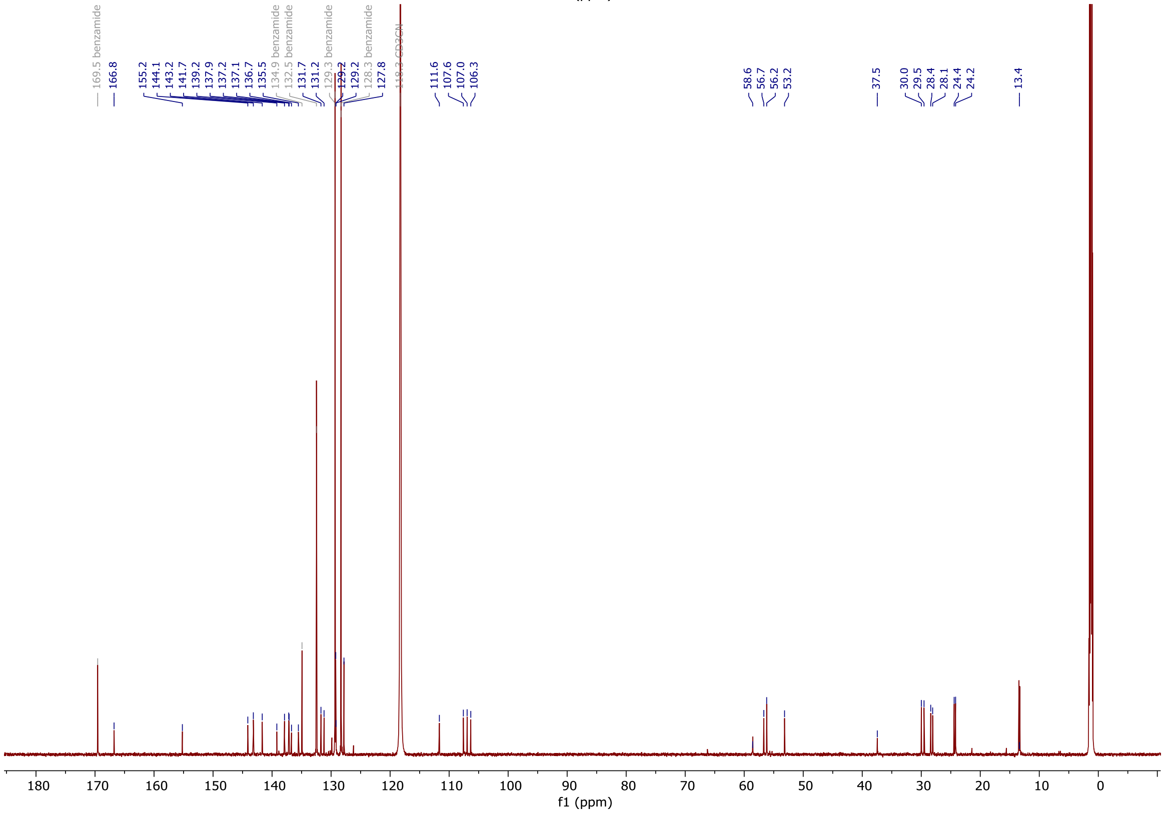

**Fig**. **S27.** Compound 40D $^{1}$H NMR (800 MHz, $CD_3CN$, 25 °C, top) and $^{13}$C NMR (201 MHz, $CD_3CN$, 25 °C, bottom).

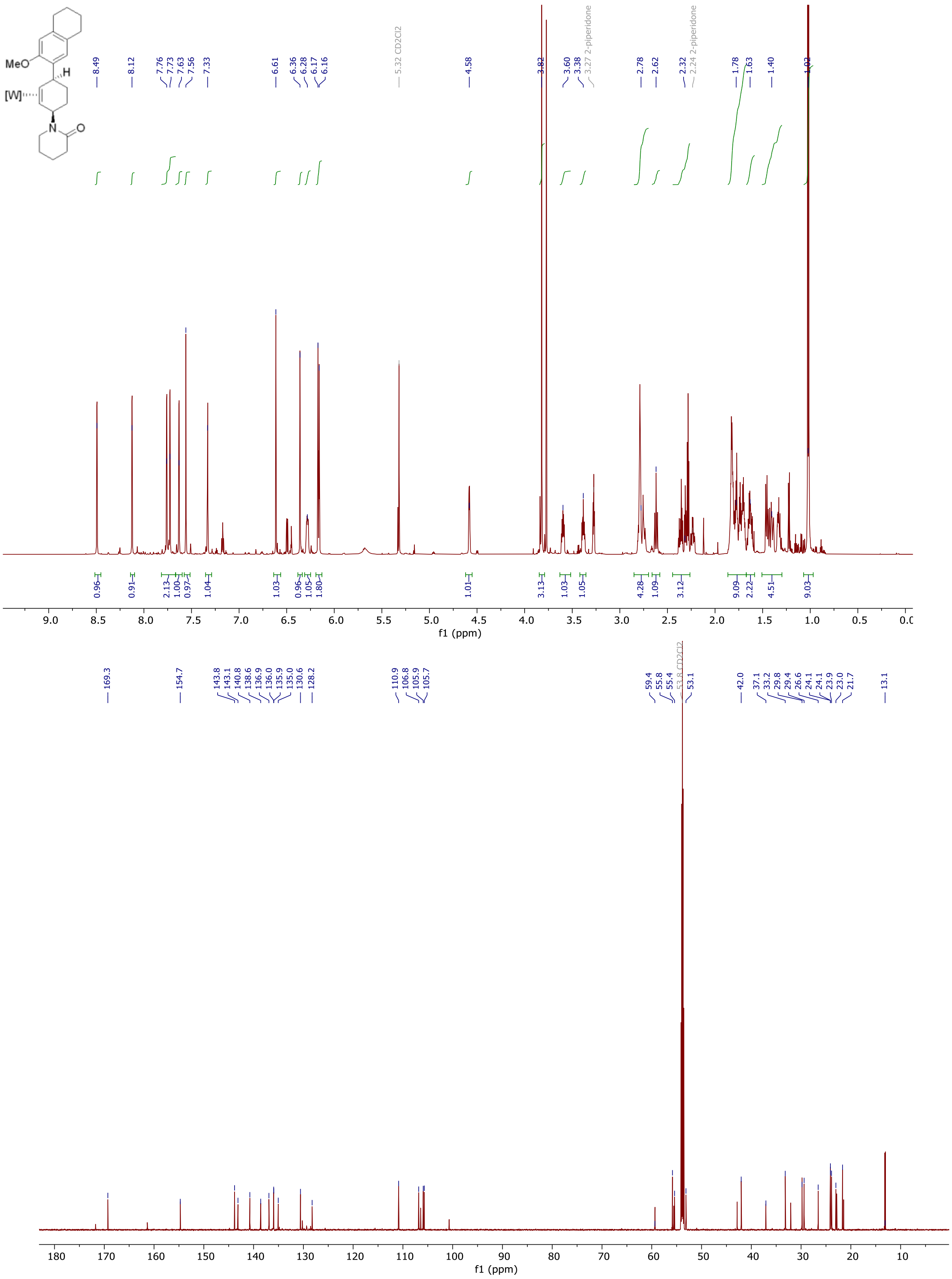


**Fig**. **S28.** Compound 41D $^1$H NMR (800 MHz, $CD_2Cl_2$, 25 °C, top) and $^{13}$C NMR (201 MHz, $CD_2Cl_2$, 25 °C, bottom).

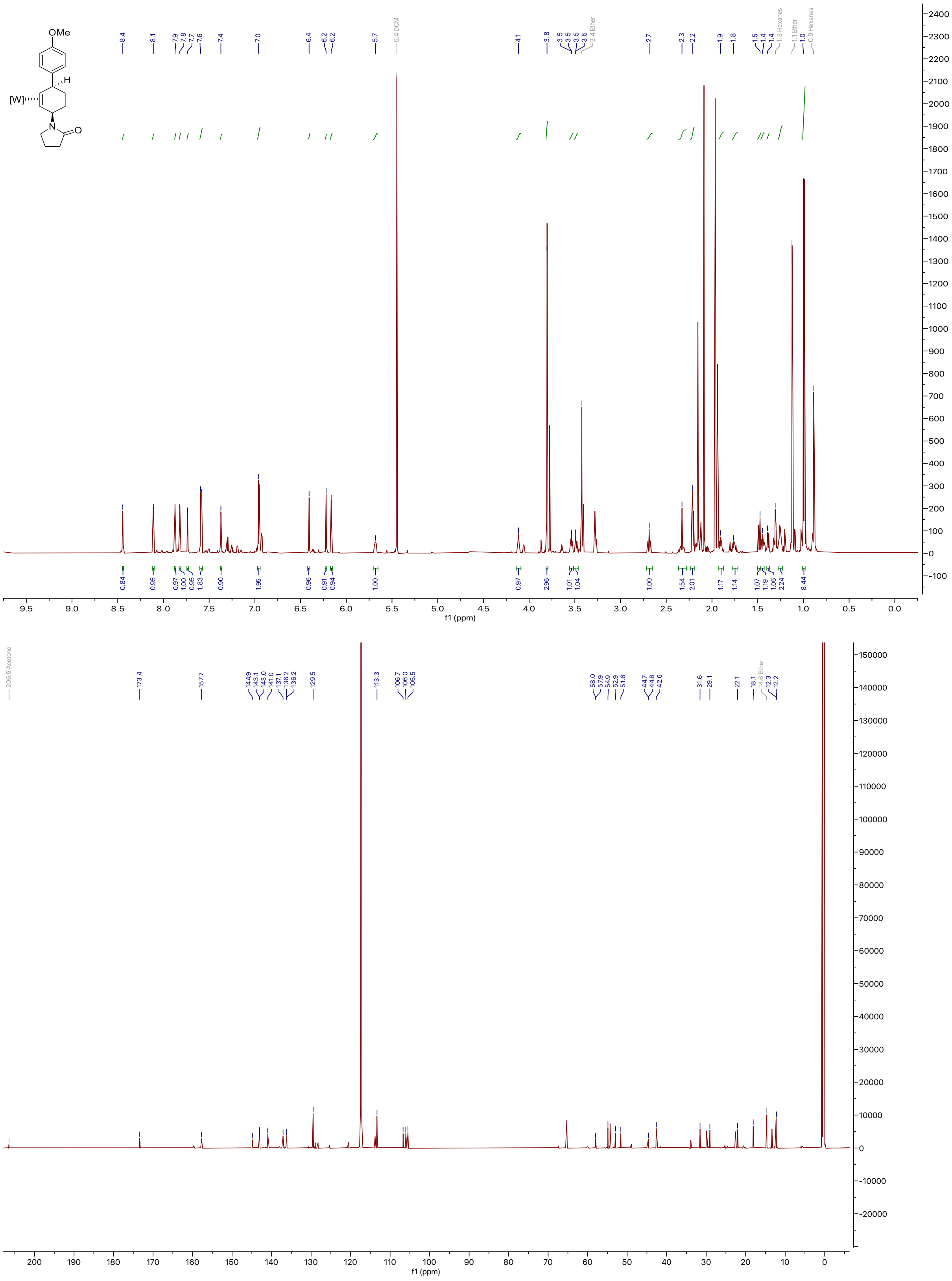


**Fig**. **S29.** Compound 42D $^1$H NMR (800 MHz, $CD_3CN$, 25 °C, top) and $^{13}$C NMR (201 MHz, $CD_3CN$, 25 °C, bottom).

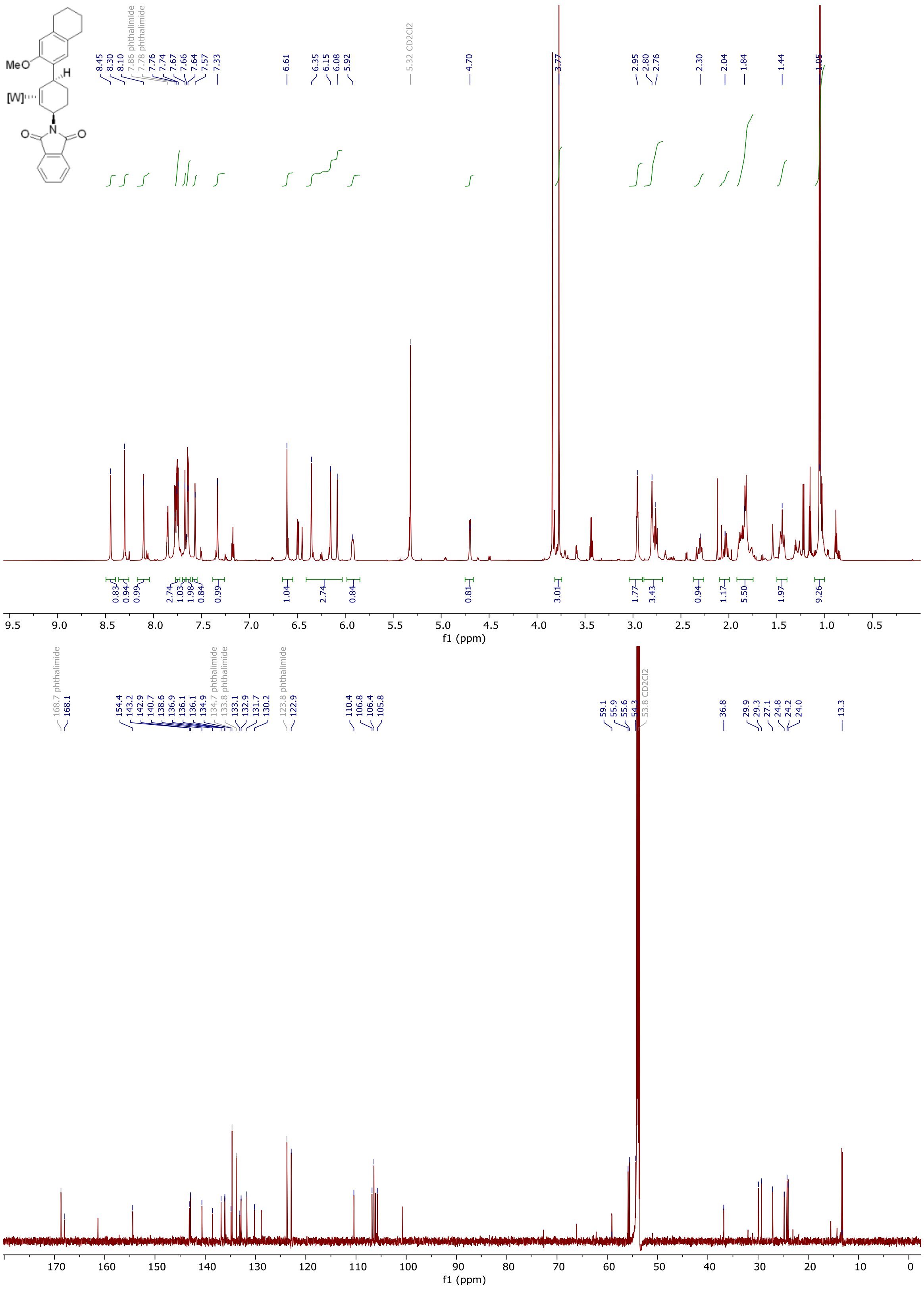

**Fig**. **S30.** Compound 43D $^{1}H$ NMR (800 MHz, $CD_2Cl_2$, 25 °C, top) and $^{13}C$ NMR (201 MHz, $CD_2Cl_2$, 25 °C, bottom).

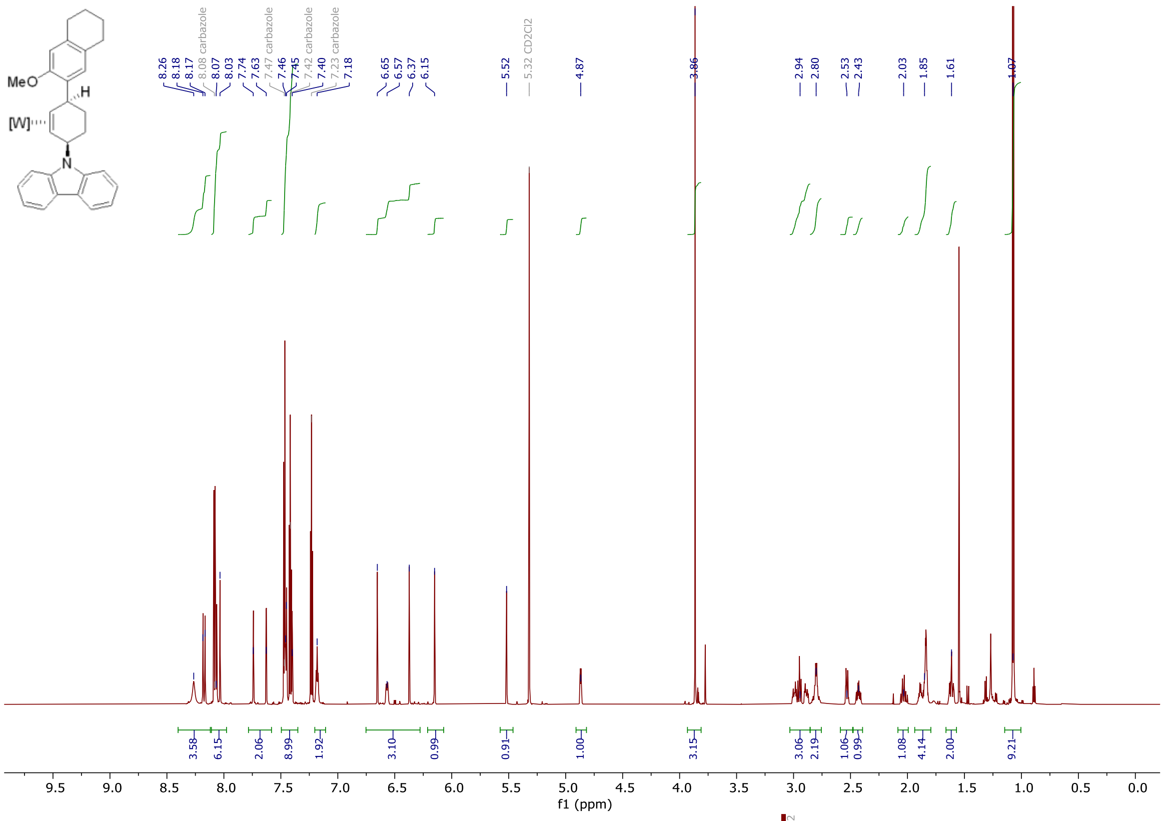

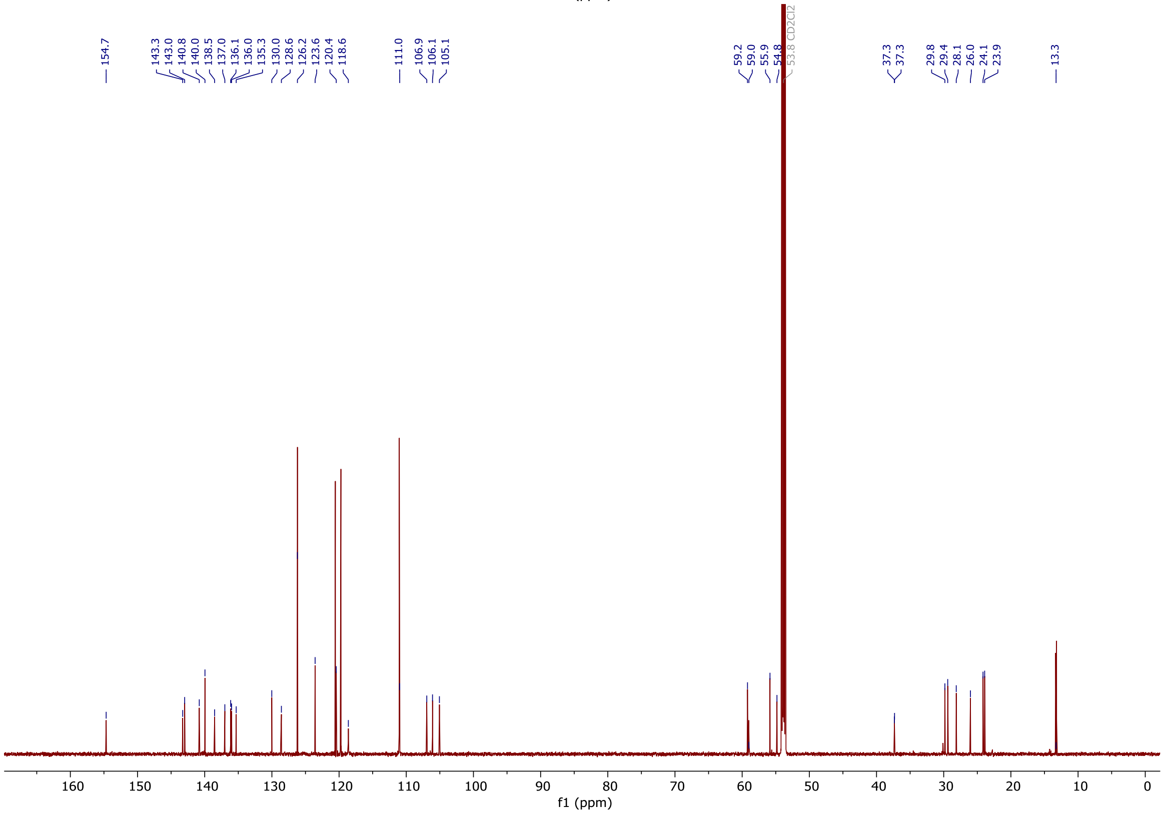

**Fig**. **S31.** Compound 44D $^{1}$H NMR (800 MHz, $CD_2Cl_2$, 25 °C, top) and $^{13}$C NMR (201 MHz, $CD_2Cl_2$, 25 °C, bottom).

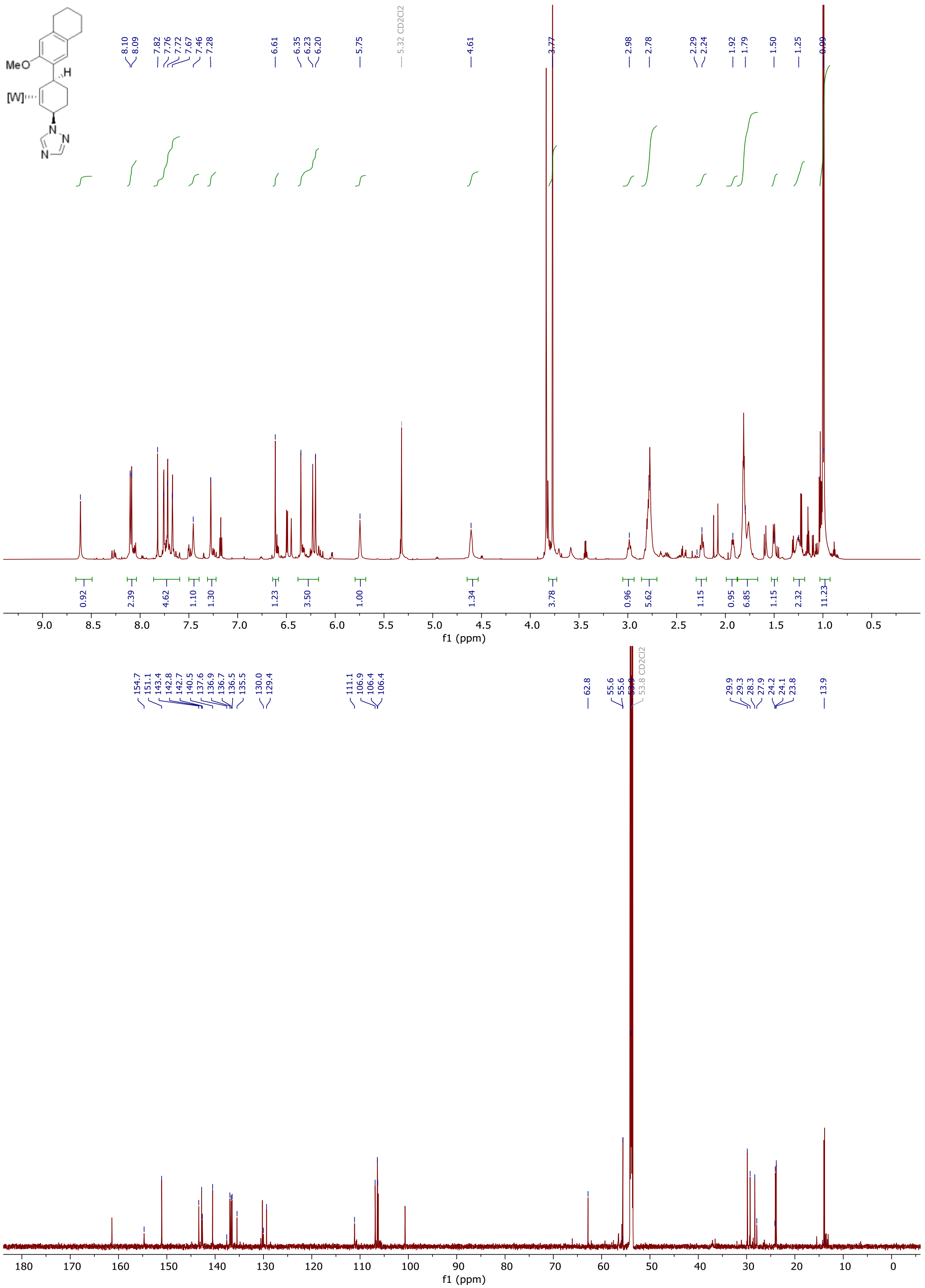


**Fig**. **S32.** Compound 45D $^{1}$H NMR (800 MHz, $CD_2Cl_2$, 25 °C, top) and $^{13}$C NMR (201 MHz, $CD_2Cl_2$, 25 °C, bottom).

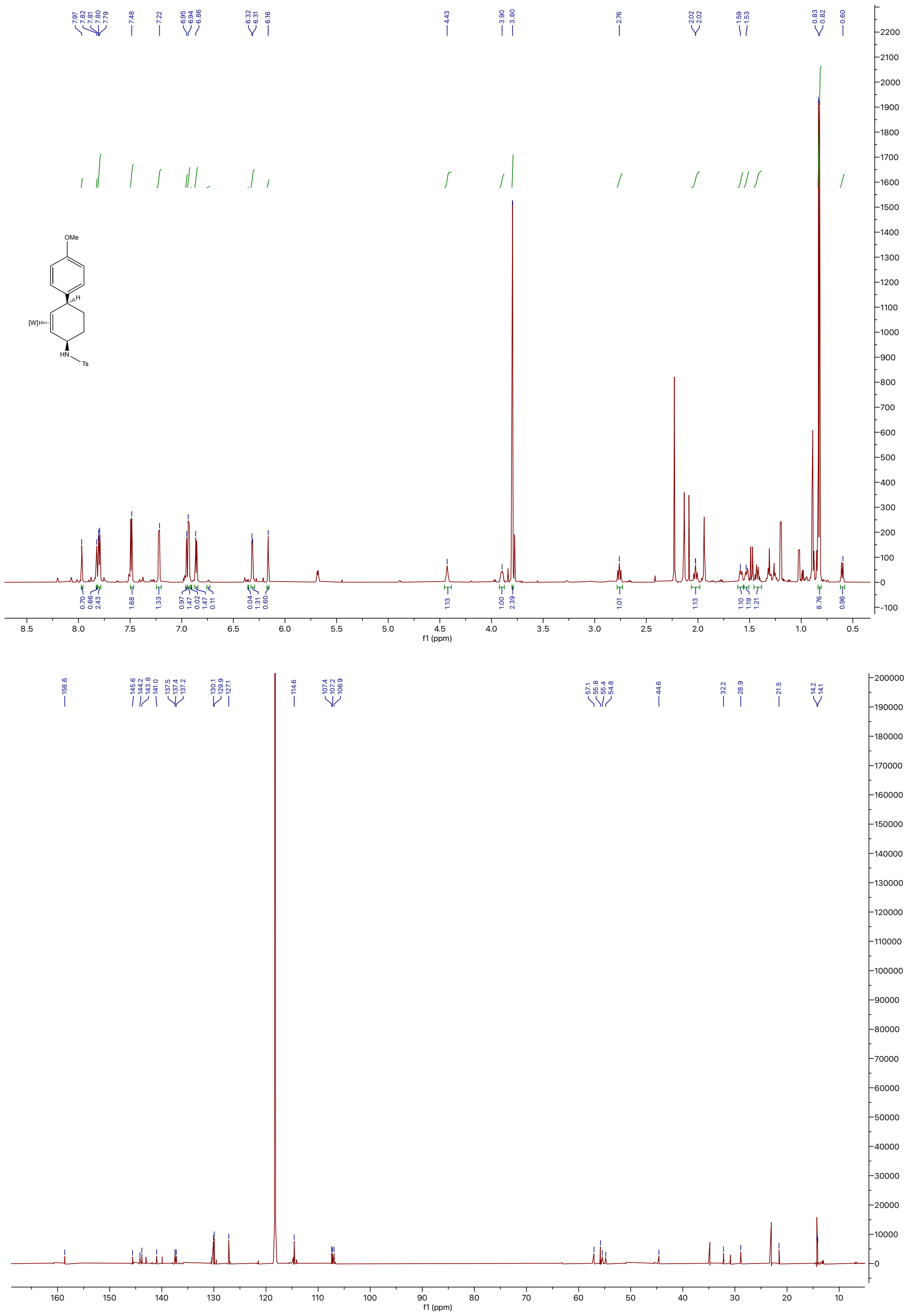


**Fig. S33.** Compound 47D $^{1}H$ NMR (800 MHz, $CD_3CN$, 25 °C, top) and $^{13}C$ NMR (201 MHz, $CD_3CN$, 25 °C, bottom).

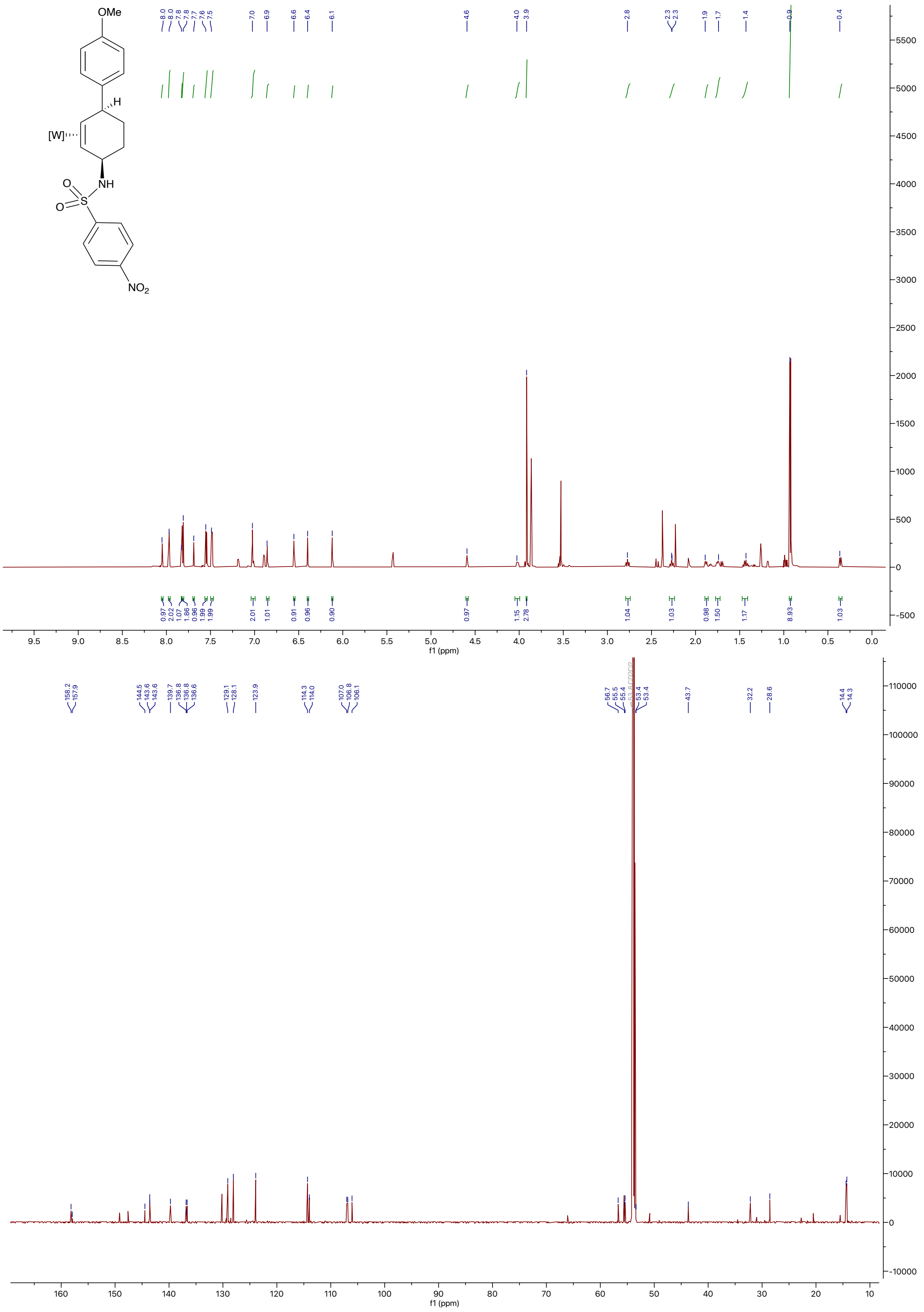


**Fig. S34.** Compound 82D $^1$H NMR (800 MHz, $CD_2Cl_2$, 25 °C, top) and $^{13}$C NMR (201 MHz, $CD_2Cl_2$, 25 °C, bottom).

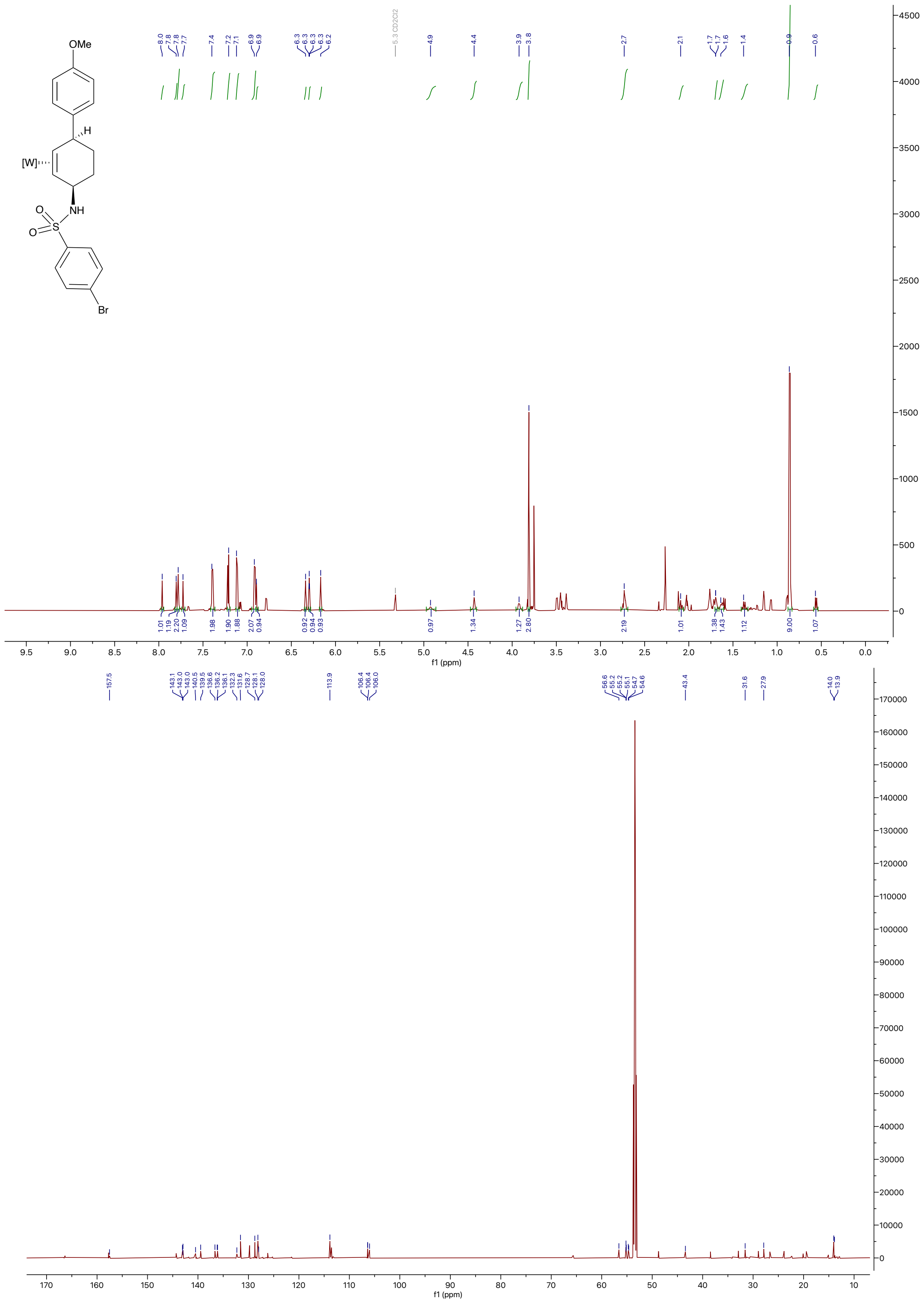


**Fig. S35.** Compound 83D $^{1}H$ NMR (800 MHz, $CD_2Cl_2$, 25 °C, top) and $^{13}C$ NMR (201 MHz, $CD_2Cl_2$, 25 °C, bottom).

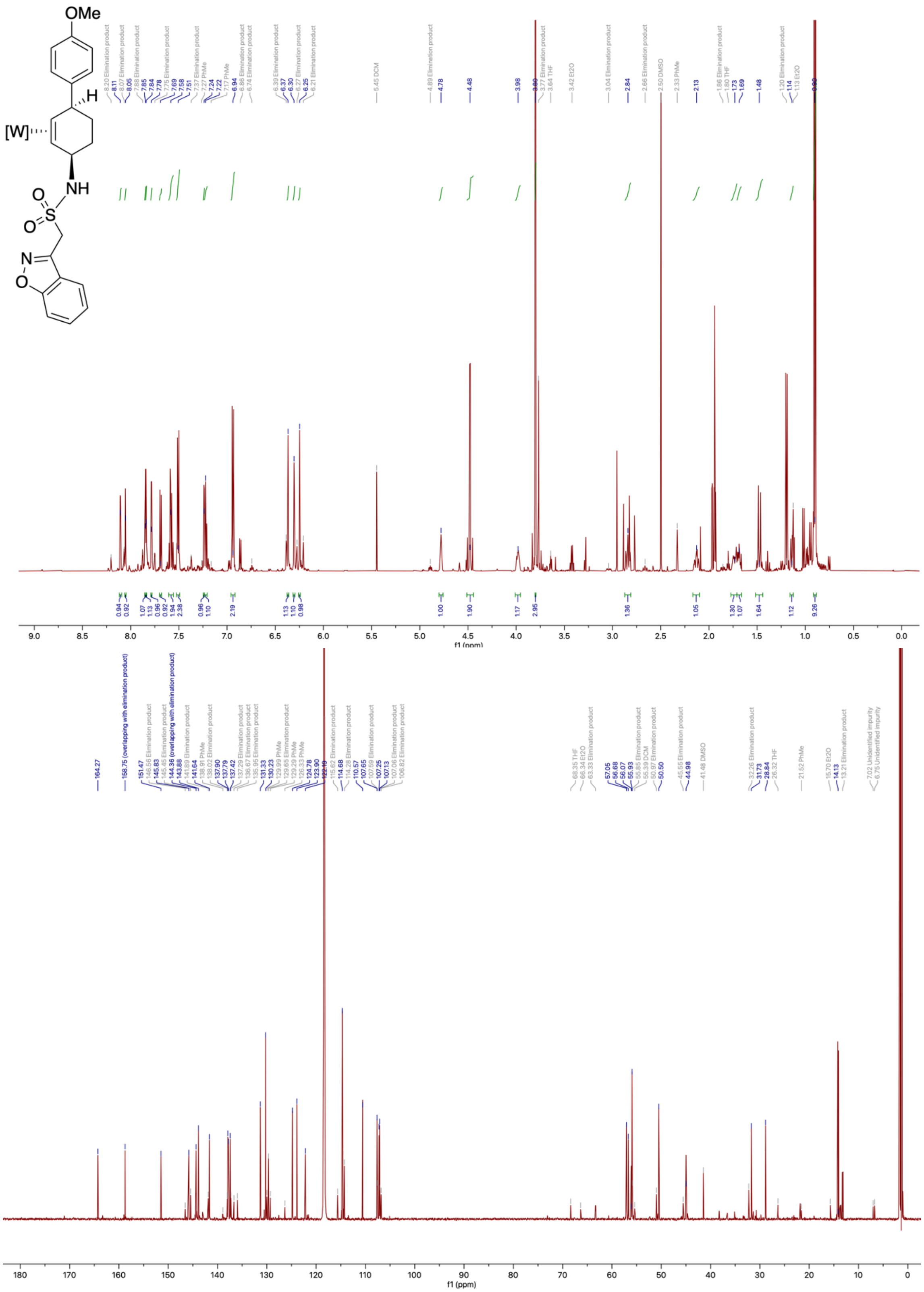


**Fig. S36.** Compound 48D $^{1}H$ NMR (600 MHz, $CD_3CN$, 25 °C, top) and $^{13}C$ NMR (201 MHz, $CD_3CN$, 25 °C, bottom).

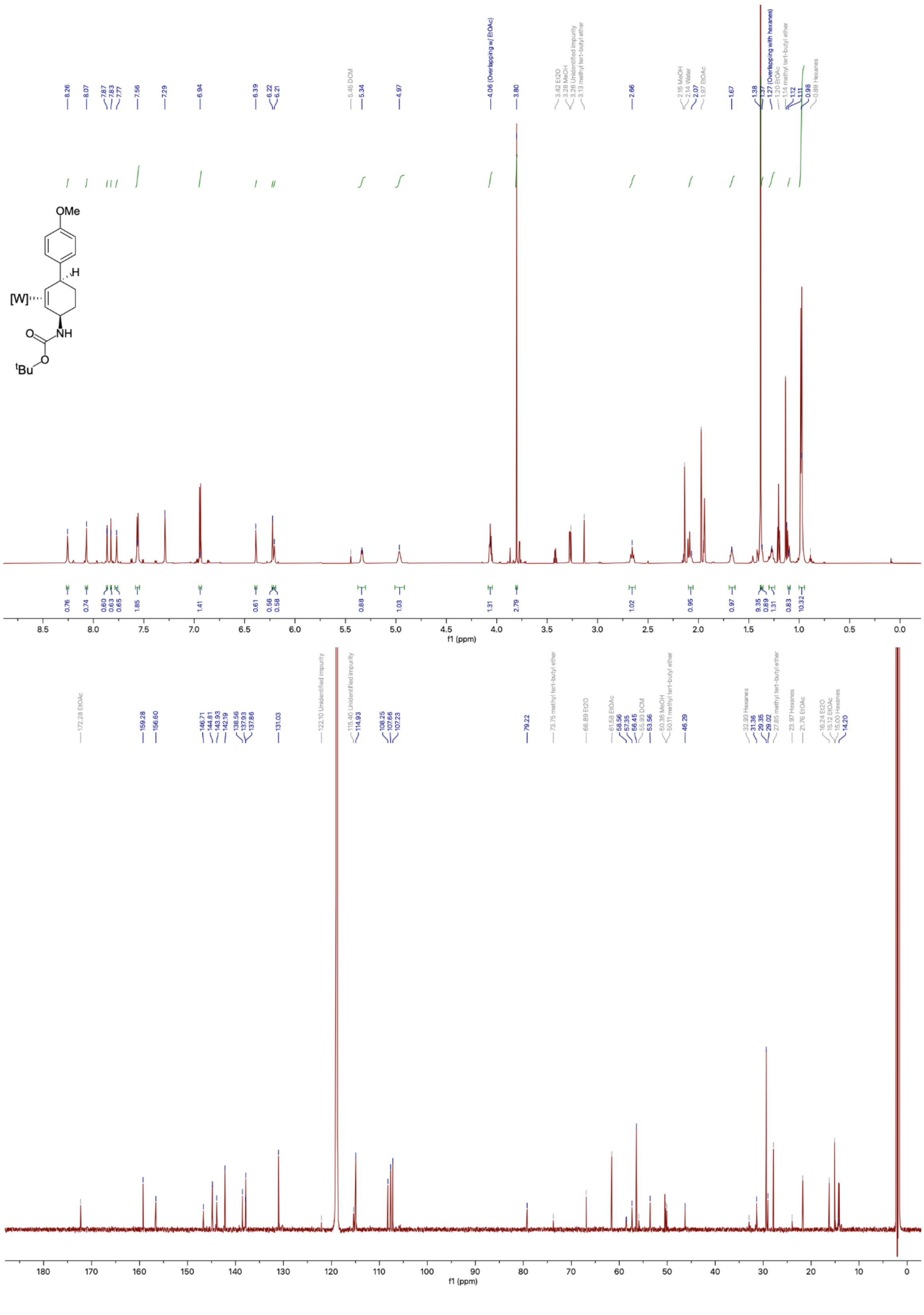

**Fig. S37.** Compound 49D $^1$H NMR (800 MHz, $CD_3CN$, 25 °C, top) and $^{13}$C NMR (201 MHz, $CD_3CN$, 25 °C, bottom).

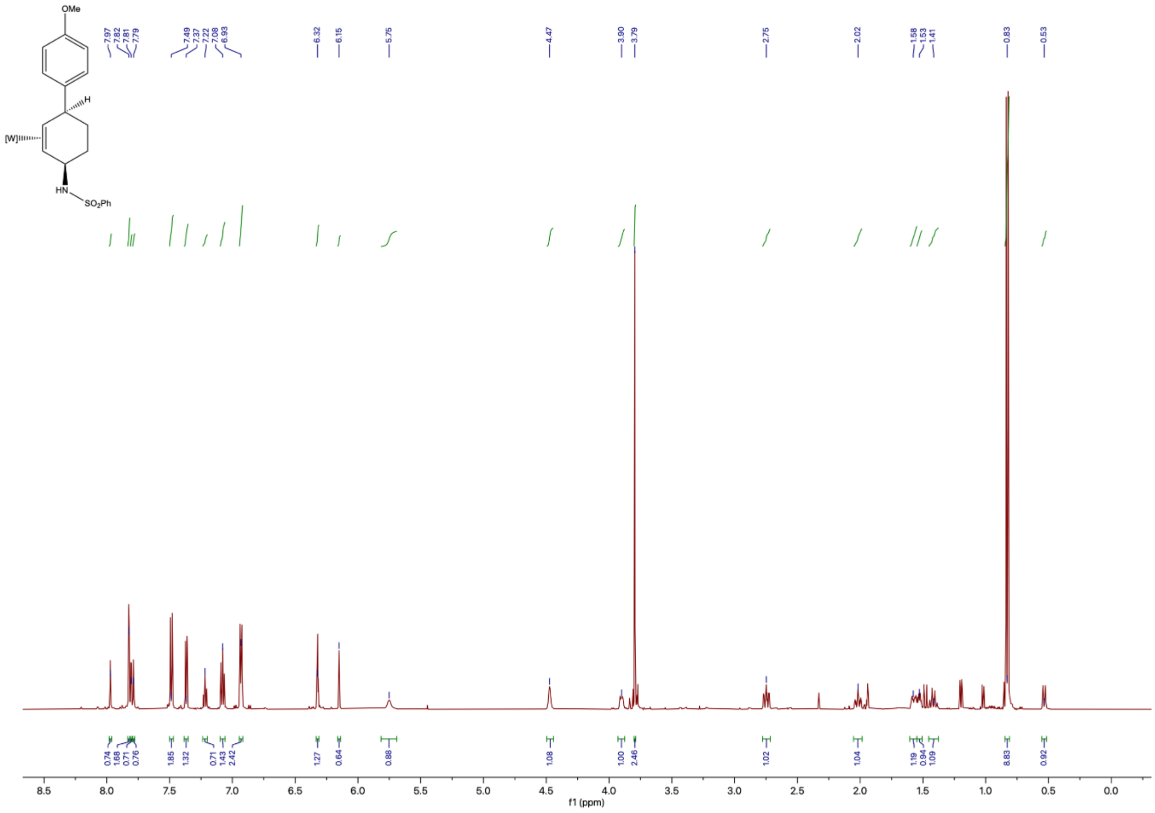

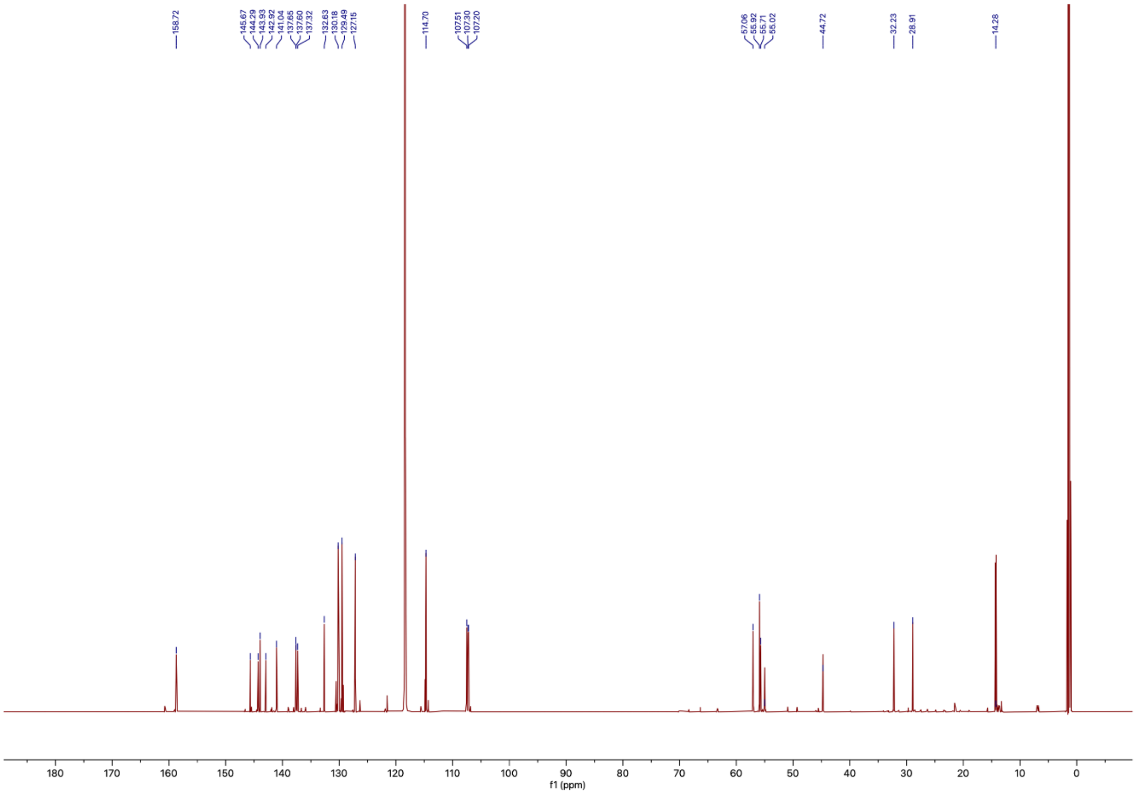

**Fig. S38.** Compound 50D $^{1}H$ NMR (600 MHz, $CD_3CN$, 25 °C, top) and $^{13}C$ NMR (201 MHz, $CD_3CN$, 25 °C, bottom).

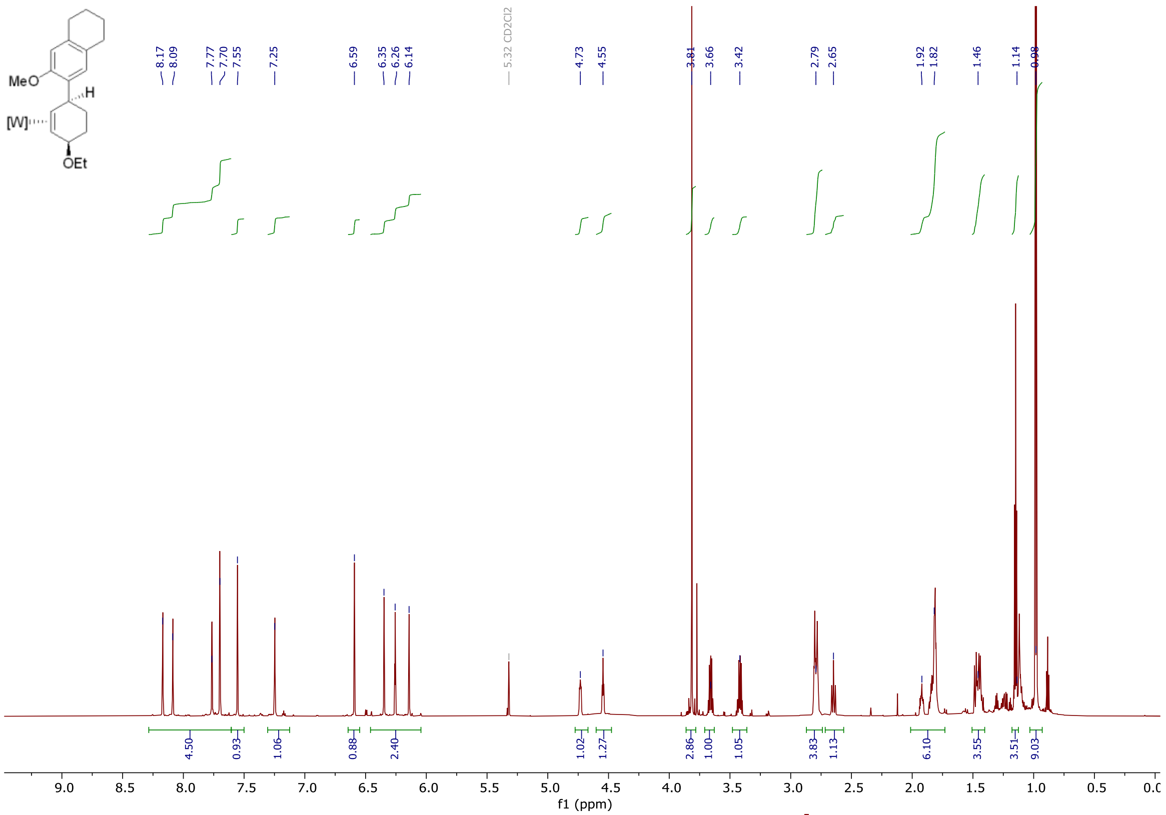

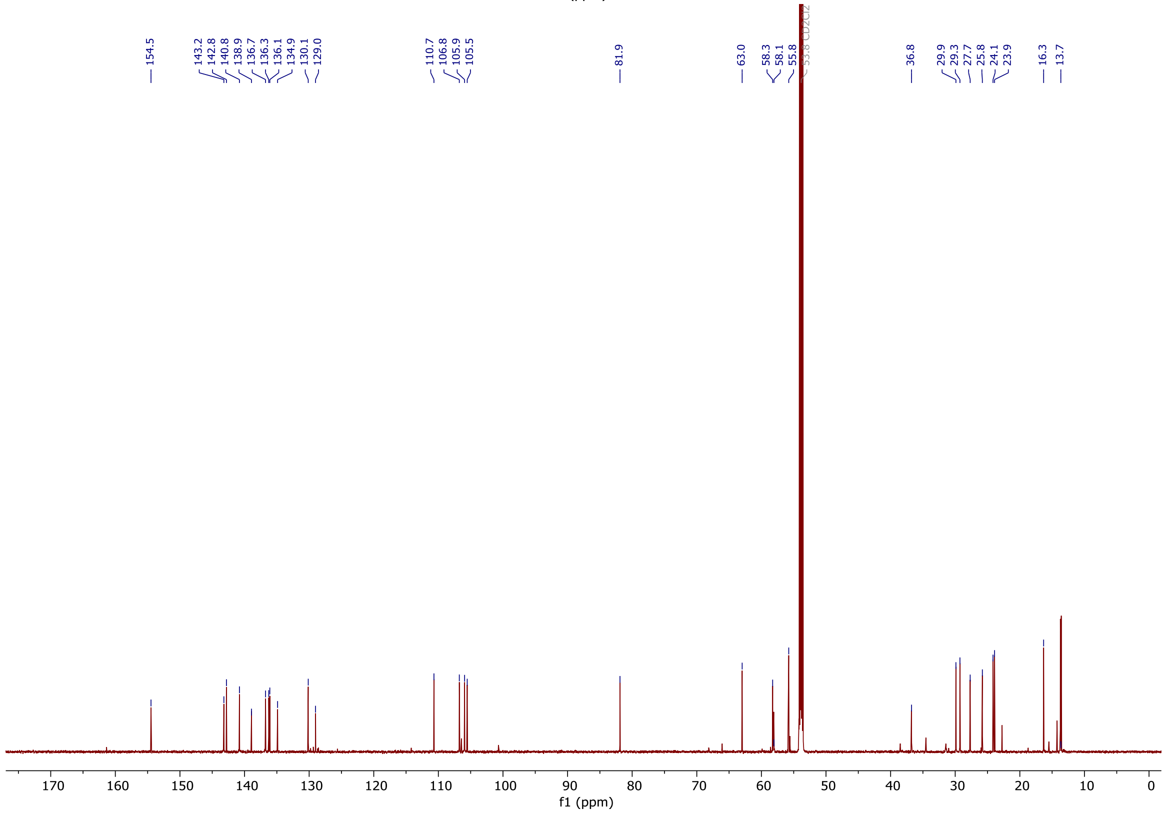

**Fig. S39.** Compound 52D $^{1}H$ NMR (800 MHz, $CD_2Cl_2$, 25 °C, top) and $^{13}C$ NMR (201 MHz, $CD_2Cl_2$, 25 °C, bottom).

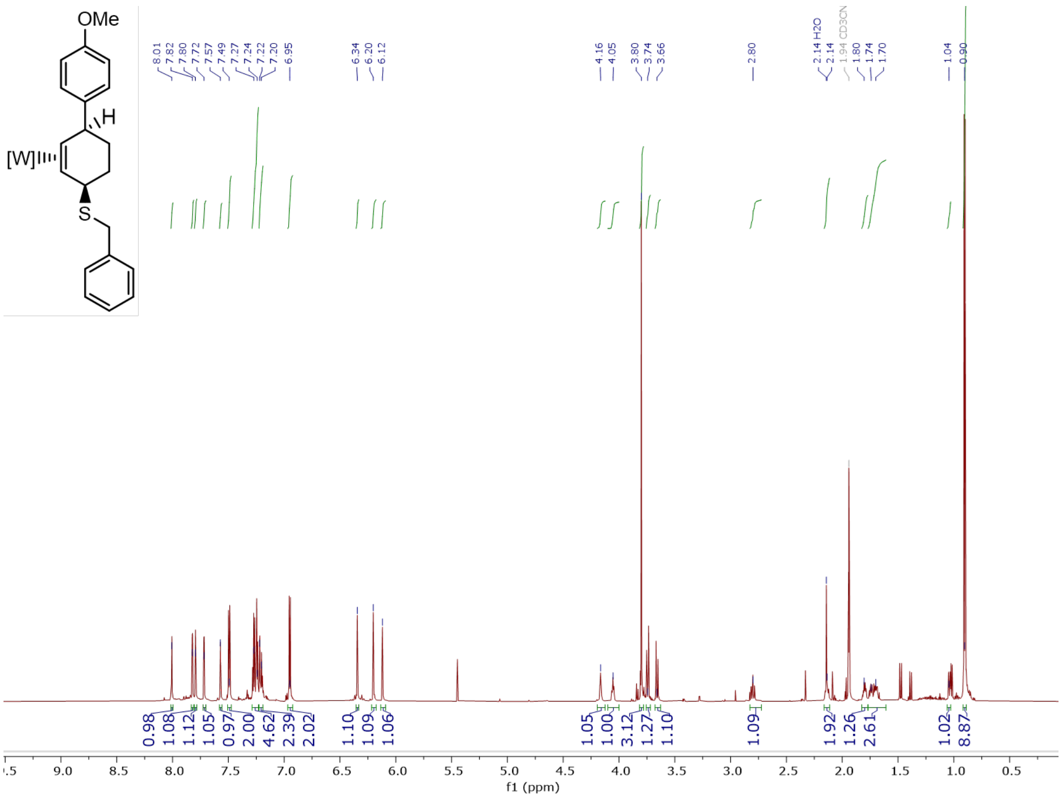

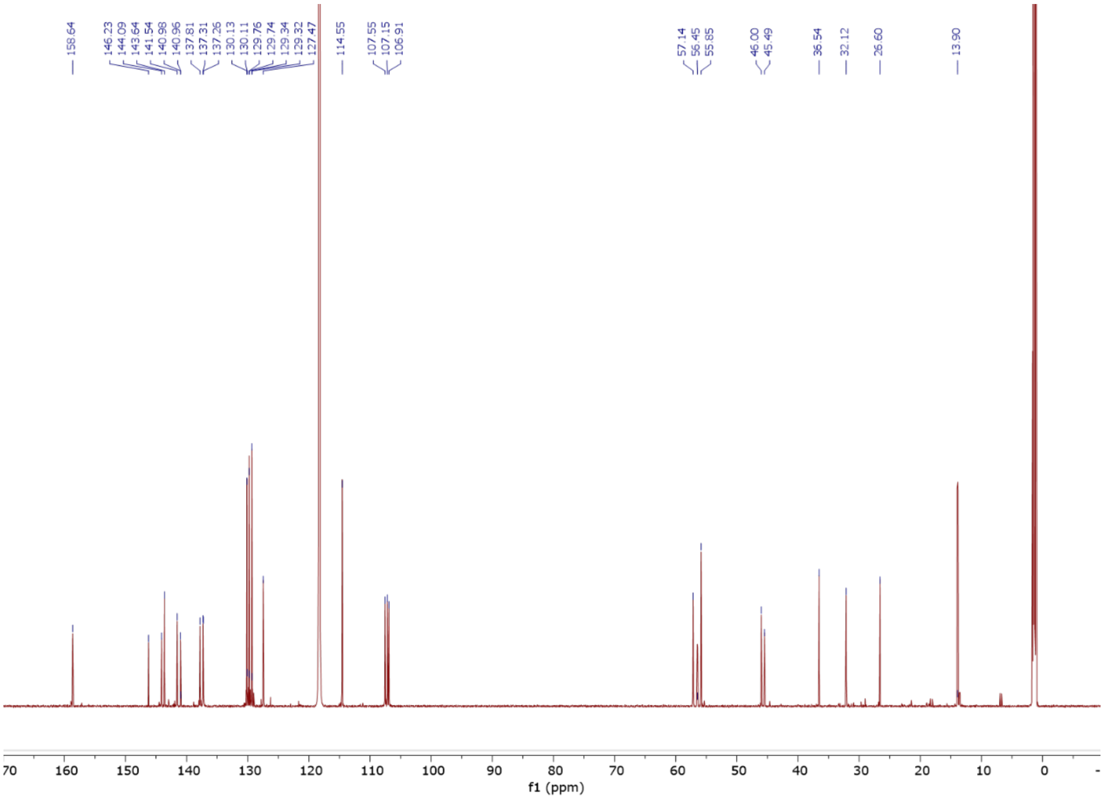

**Fig. S40.** Compound 53D $^{1}H$ NMR (800 MHz, $CD_3CN$, 25 °C, top) and $^{13}C$ NMR (201 MHz, $CD_3CN$, 25 °C, bottom).

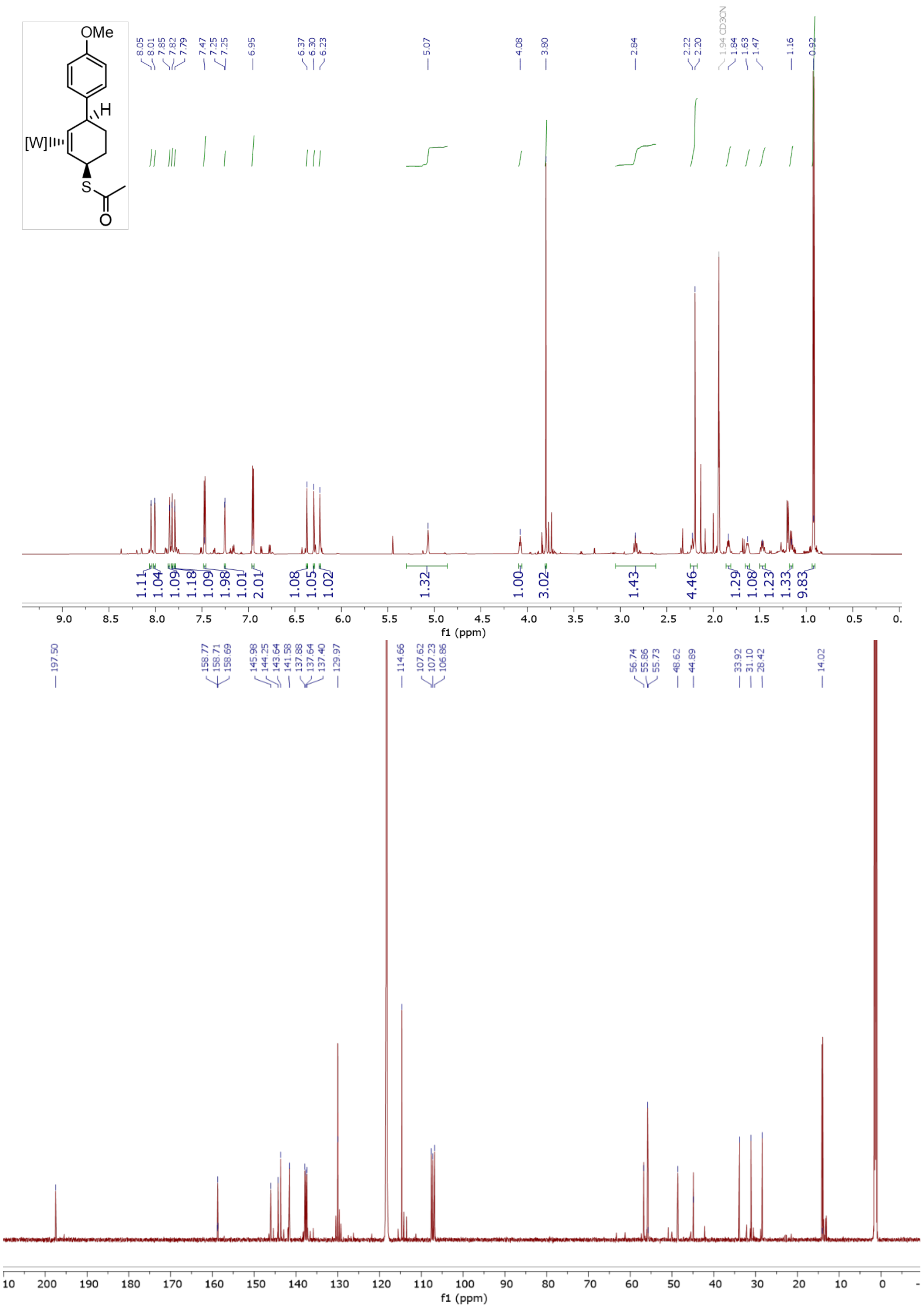


**Fig. S41.** Compound 54D $^{1}$H NMR (800 MHz, $CD_3CN$, 25 °C, top) and $^{13}$C NMR (201 MHz, $CD_3CN$, 25 °C, bottom).

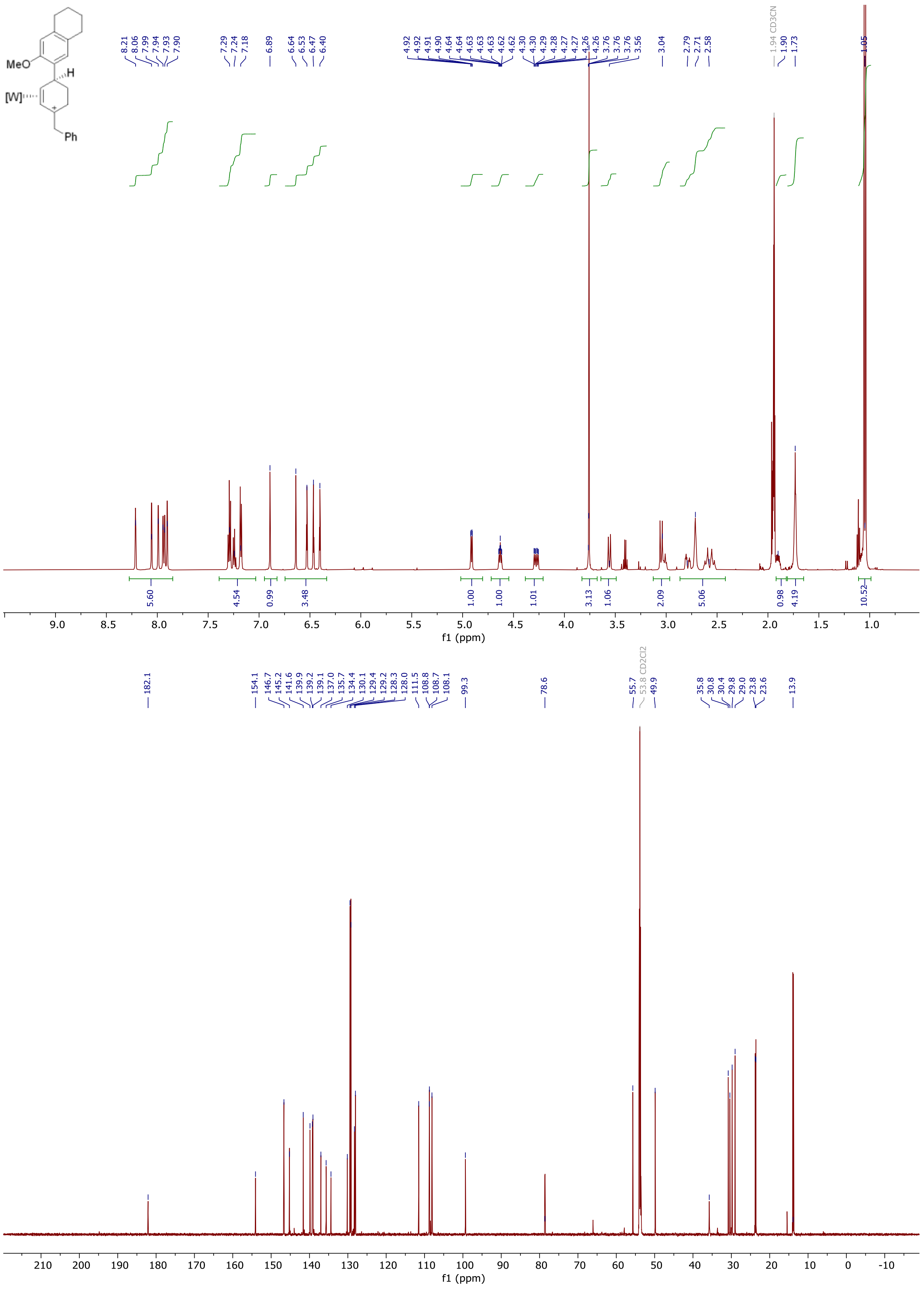

**Fig. S42.** Compound 55D $^{1}H$ NMR (600 MHz, $CD_3CN$, 25 °C, top) and $^{13}C$ NMR (201 MHz, $CD_3CN$, 25 °C, bottom).

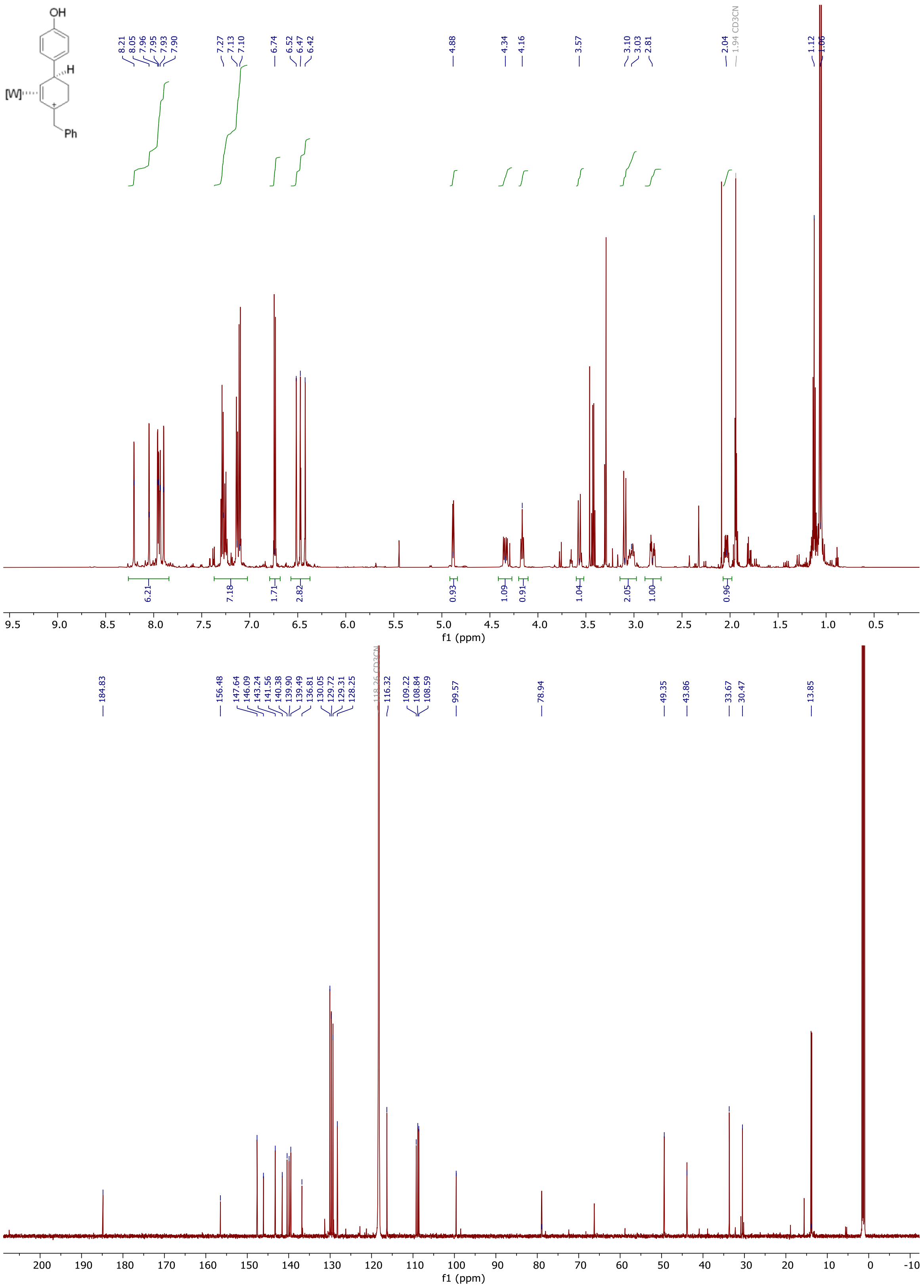


**Fig. S43.** Compound 85D $^{1}H$ NMR (600 MHz, $CD_3CN$, 25 °C, top) and $^{13}C$ NMR (201 MHz, $CD_3CN$, 25 °C, bottom).

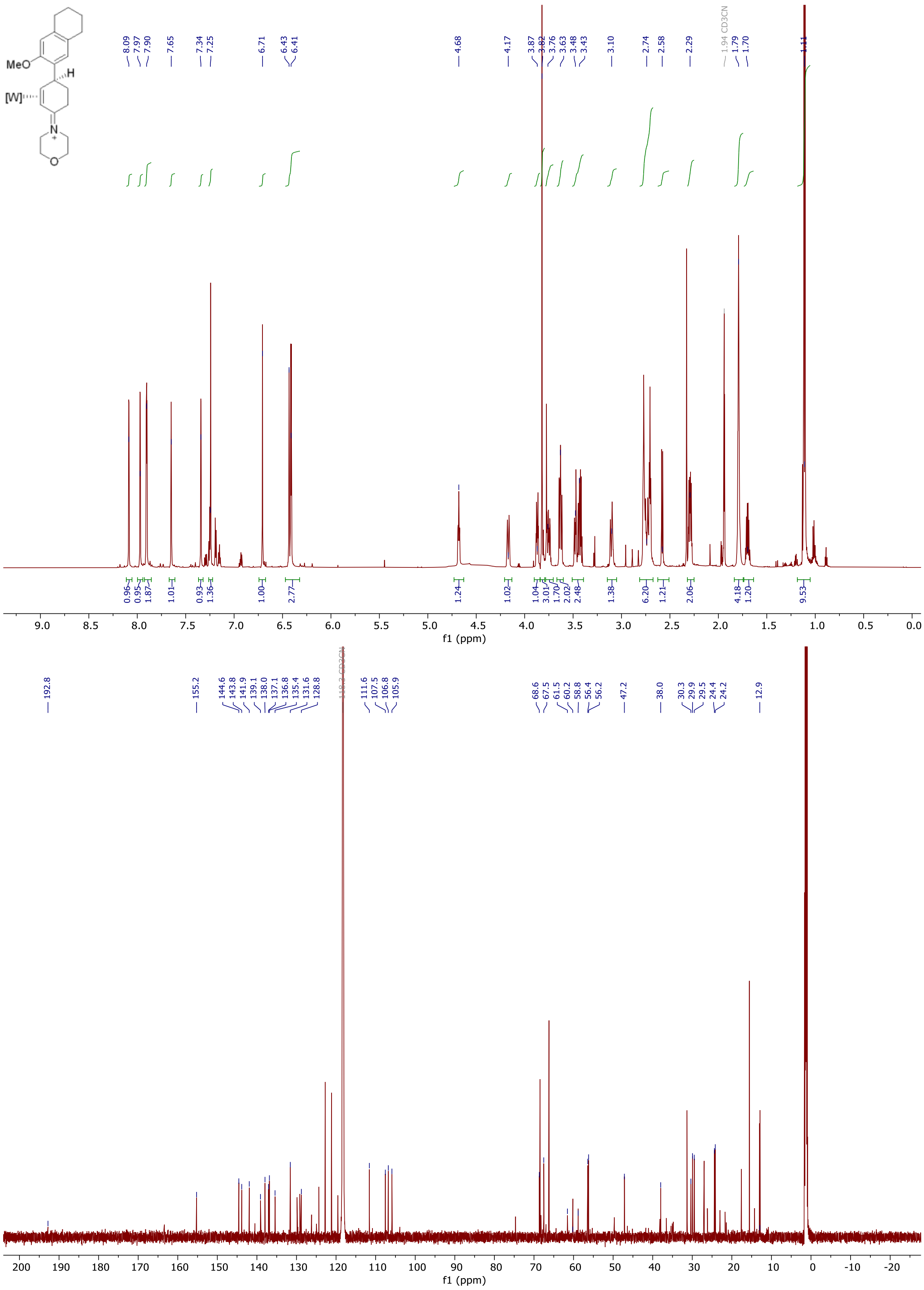

**Fig. S44.** Compound 56D $^{1}H$ NMR (800 MHz, $CD_3CN$, 25 °C, top) and $^{13}C$ NMR (201 MHz, $CD_3CN$, 25 °C, bottom).

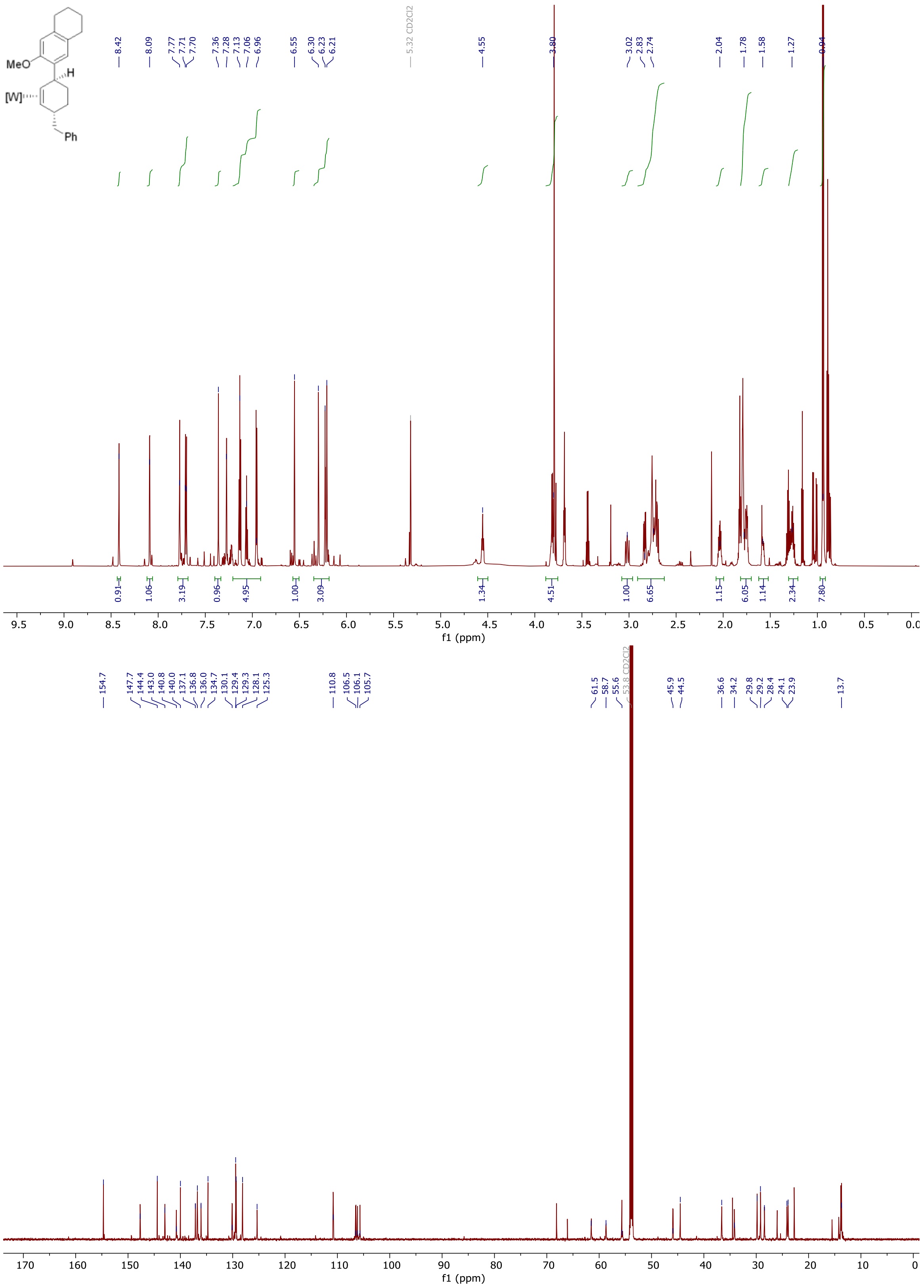


**Fig. S45.** Compound 59D $^1H$ NMR (800 MHz, $CD_2Cl_2$, 25 °C, top) and $^{13}C$ NMR (201 MHz, $CD_2Cl_2$, 25 °C, bottom).

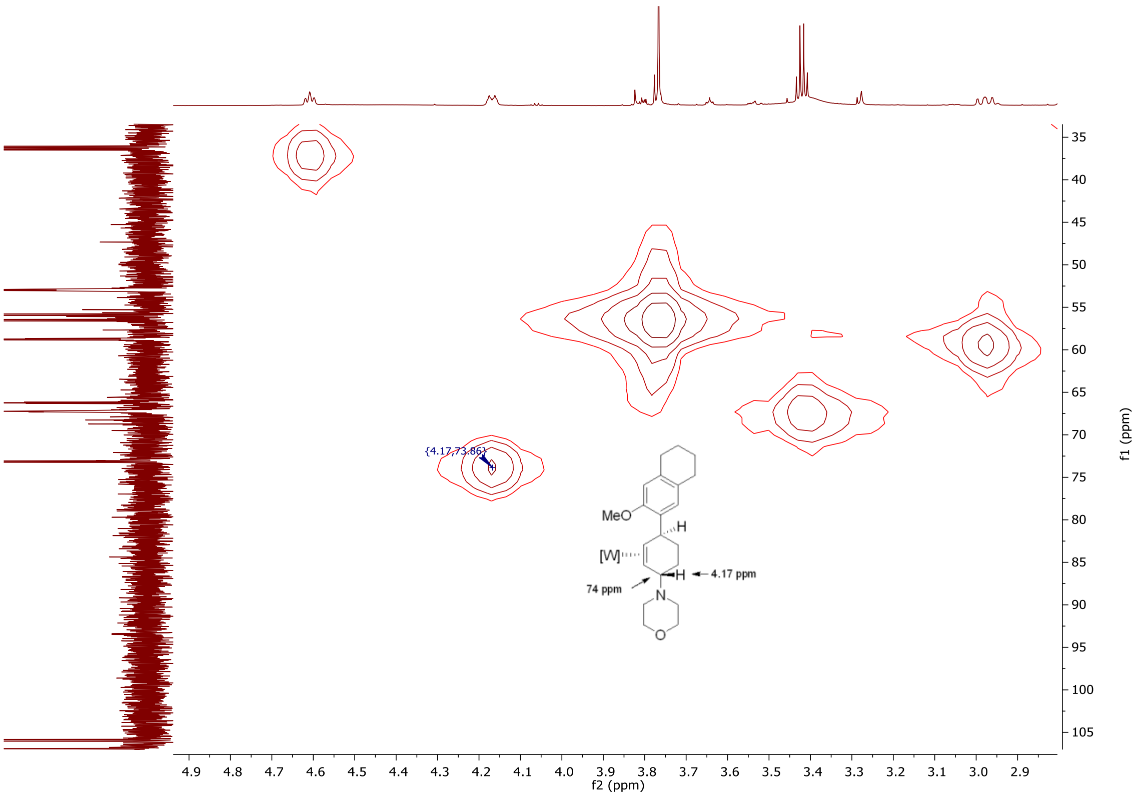


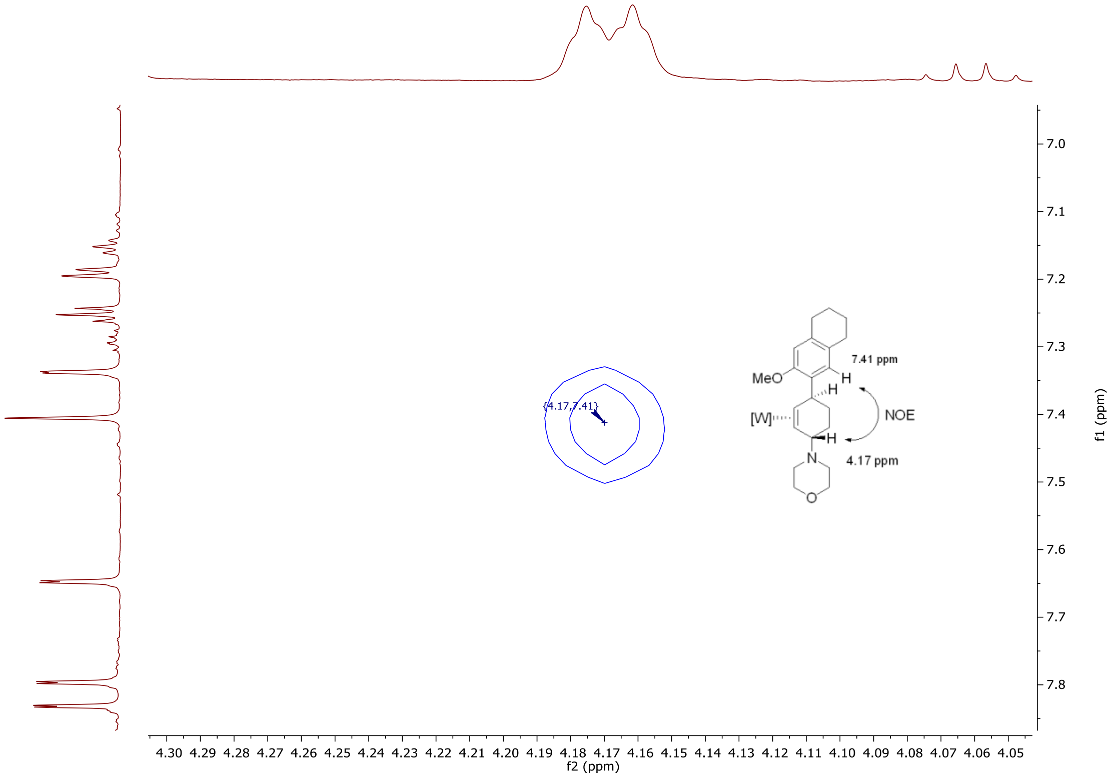


**Fig. S46.** Compound *trans*-46D HSQC (800 MHz, $CD_3CN$, 25 °C, top) and NOE data (800 MHz, $CD_3CN$, 25 °C, bottom).

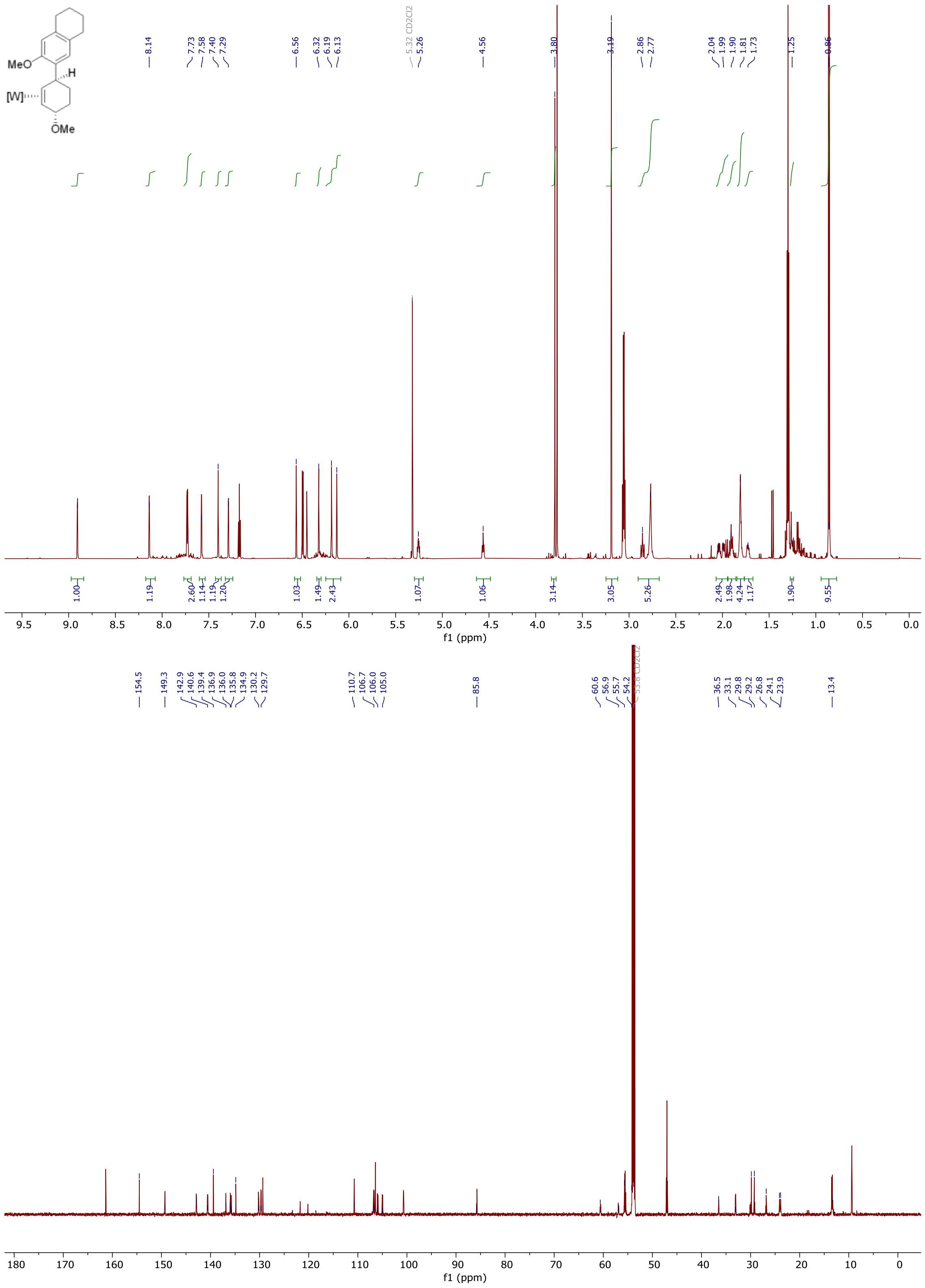

**Fig. S47.** Compound 26D $^1H$ NMR (800 MHz, $CD_2Cl_2$, 25 °C, top) and $^{13}C$ NMR (201 MHz, $CD_2Cl_2$, 25 °C, bottom).

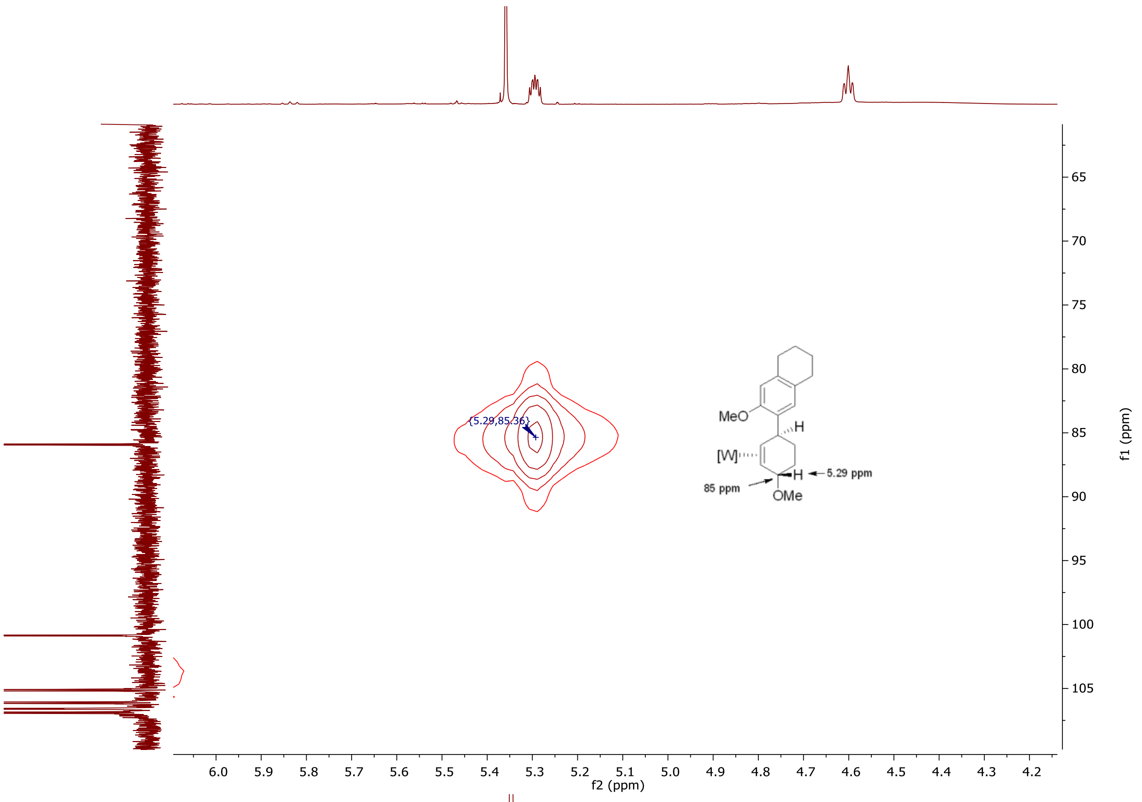


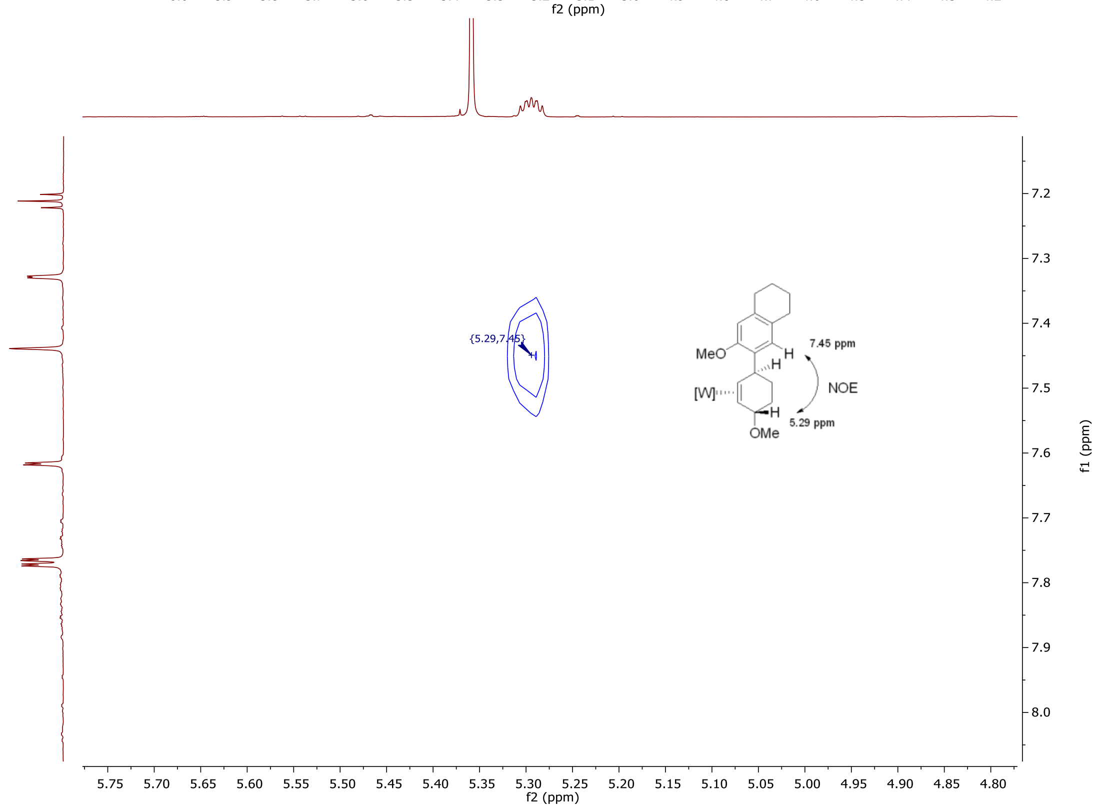


**Fig. S48.** Compound 26D HSQC (800 MHz, $CD_2Cl_2$, 25 °C, top) and NOE data (800 MHz, $CD_2Cl_2$, 25 °C, bottom).

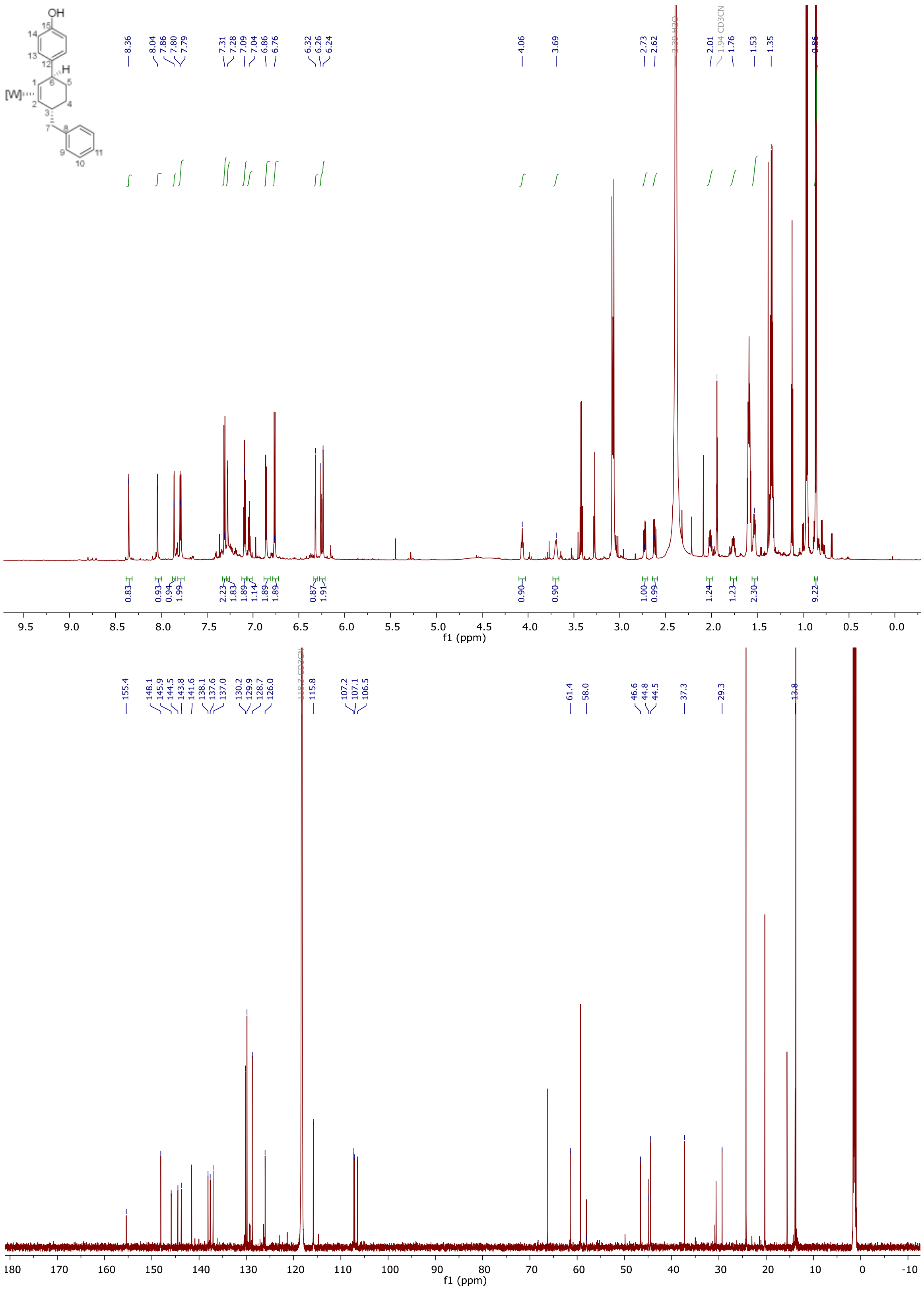

**Fig. S49.** Compound *trans*-58D $^{1}H$ NMR (800 MHz, $CD_3CN$, 25 °C, top) and $^{13}C$ NMR (201 MHz, $CD_3CN$, 25 °C, bottom).

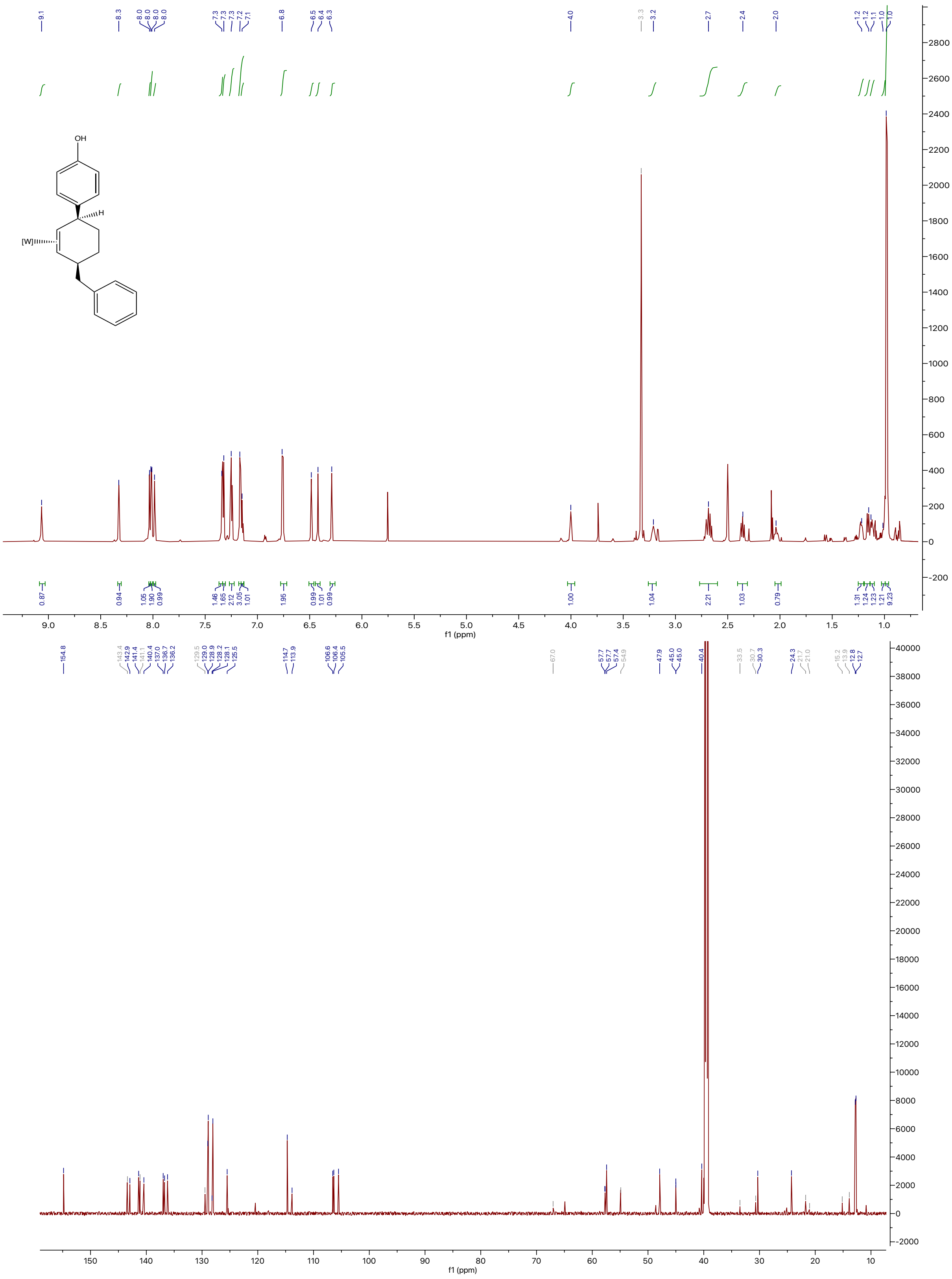


**Fig. S50.** Compound *cis*-58D (800 MHz, $(CD_3)_2SO$, 25 °C, top) and $^{13}C$ NMR (201 MHz, $(CD_3)_2SO$, 25 °C, bottom).

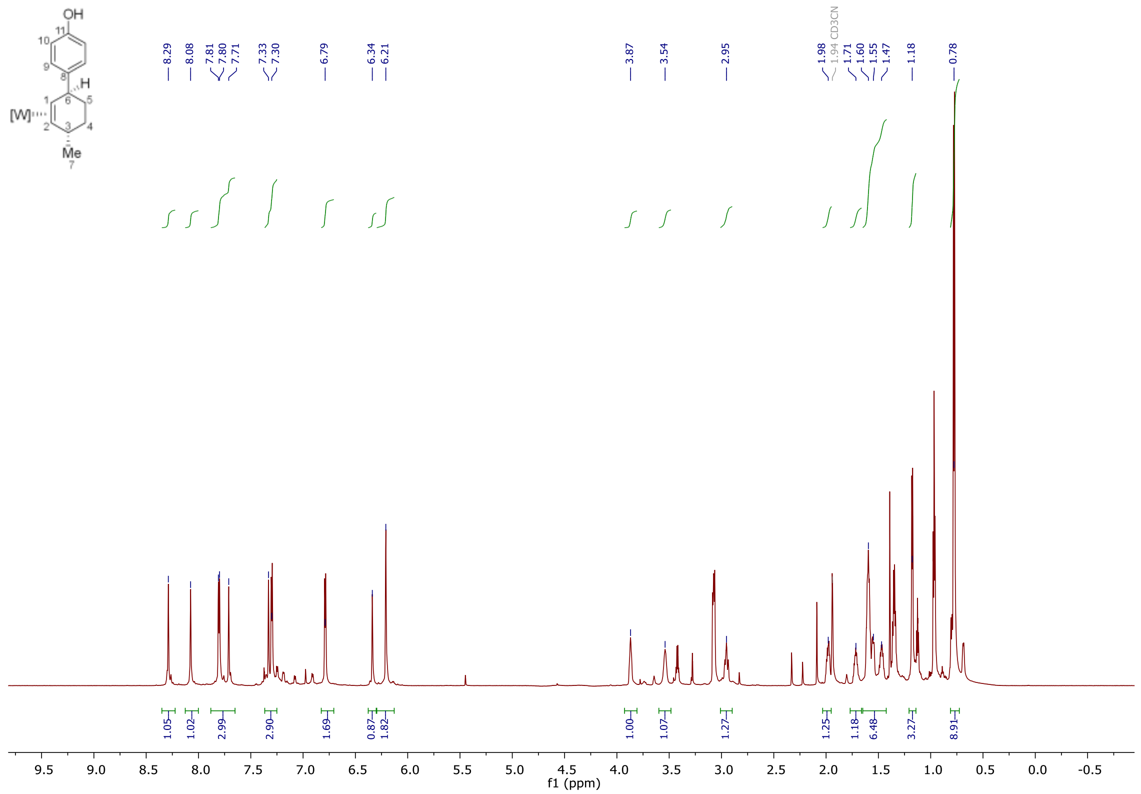

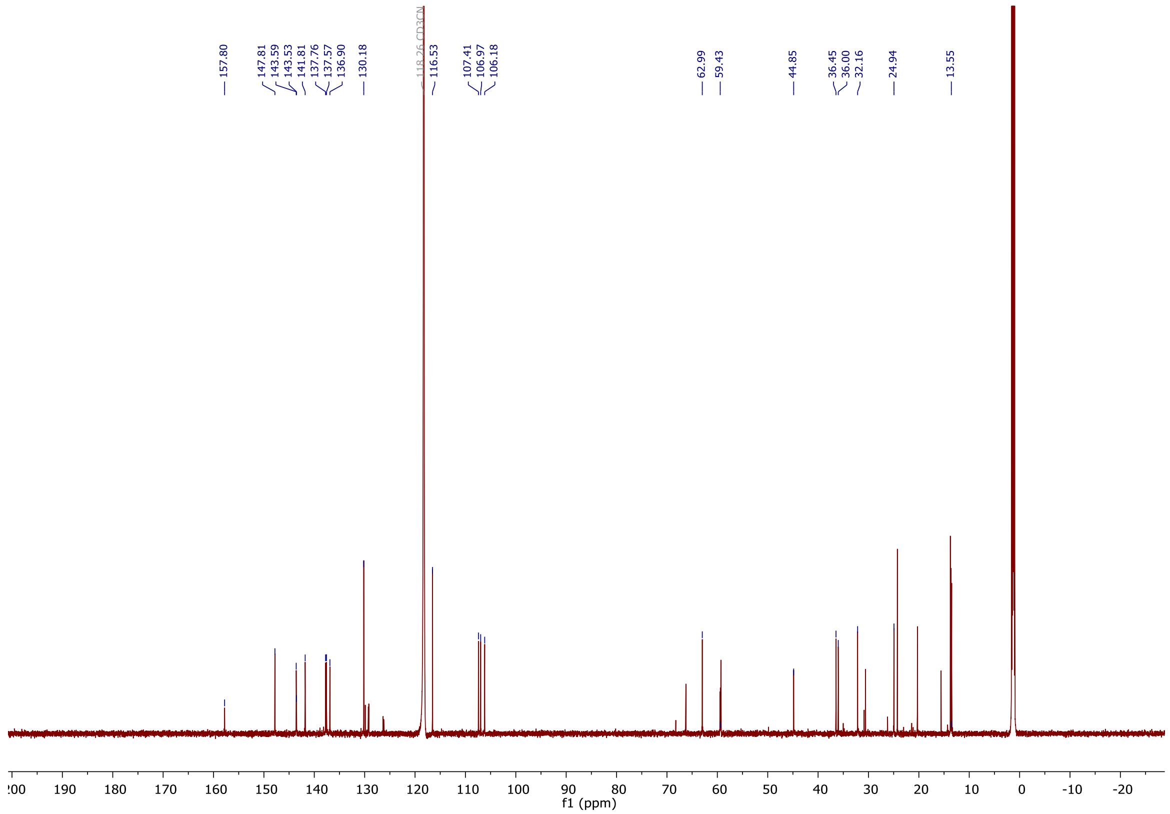

**Fig. S51.** Compound *trans*-57D $^{1}H$ NMR (600 MHz, $CD_3CN$, 25 °C, top) and $^{13}C$ NMR (201 MHz, $CD_3CN$, 25 °C, bottom).

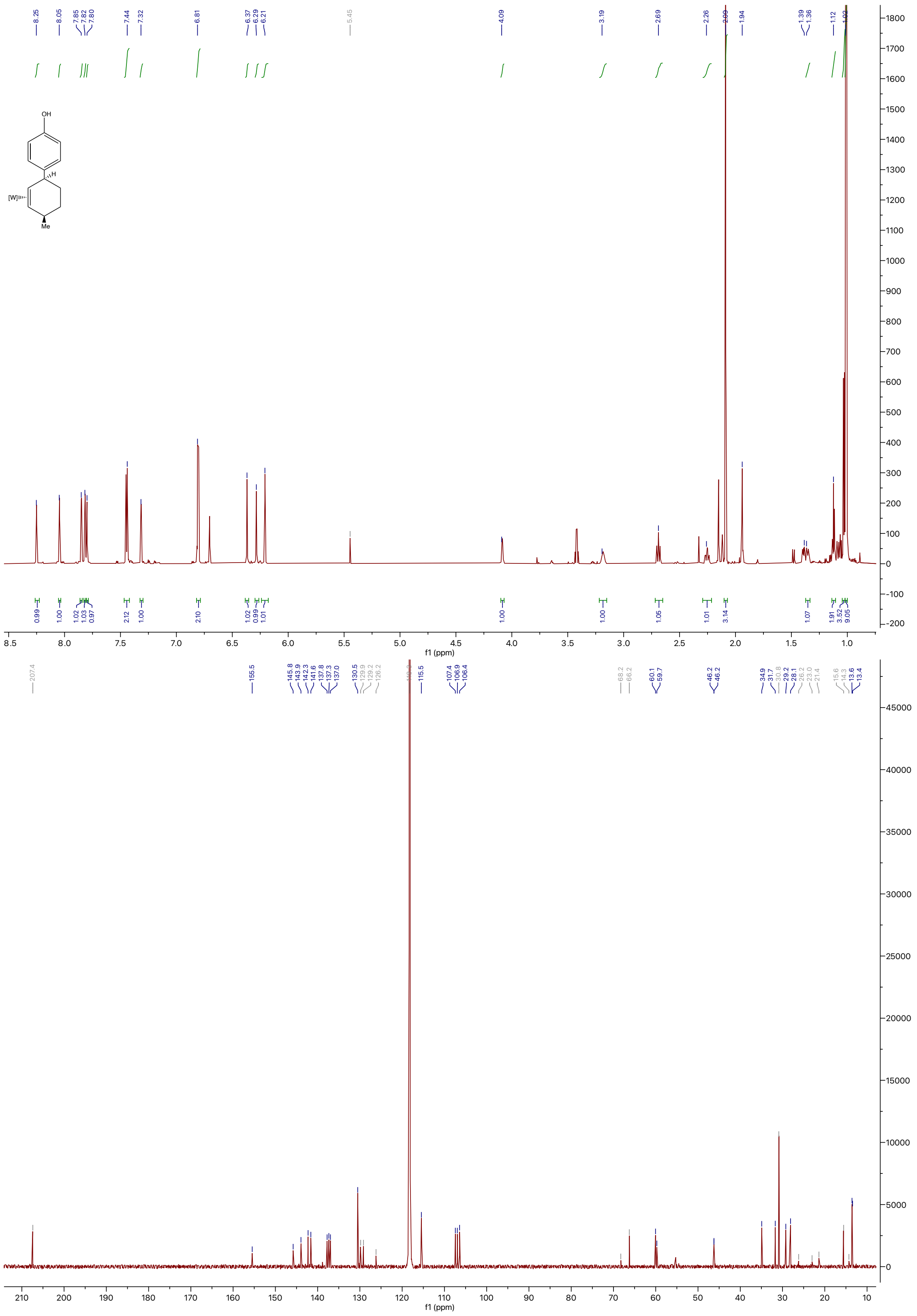

**Fig. S52.** Compound *cis*-57D $^{1}H$ NMR (600 MHz, $CD_3CN$, 25 °C, top) and $^{13}C$ NMR (201 MHz, $CD_3CN$, 25 °C, bottom).

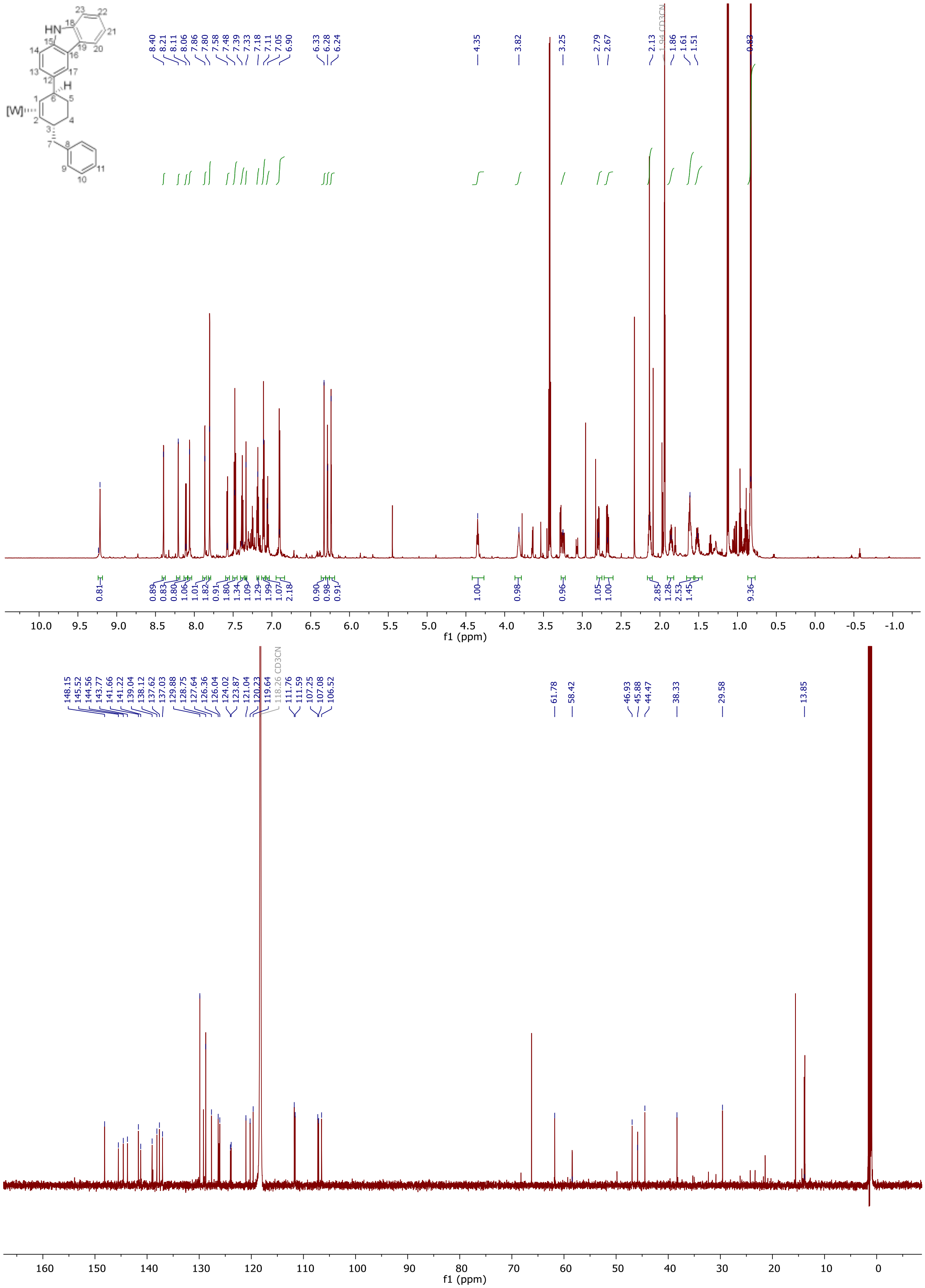

**Fig. S53.** Compound *trans*-60D $^1$H NMR (800 MHz, $CD_3CN$, 25 °C, top) and $^{13}$C NMR (201 MHz, $CD_3CN$, 25 °C, bottom).

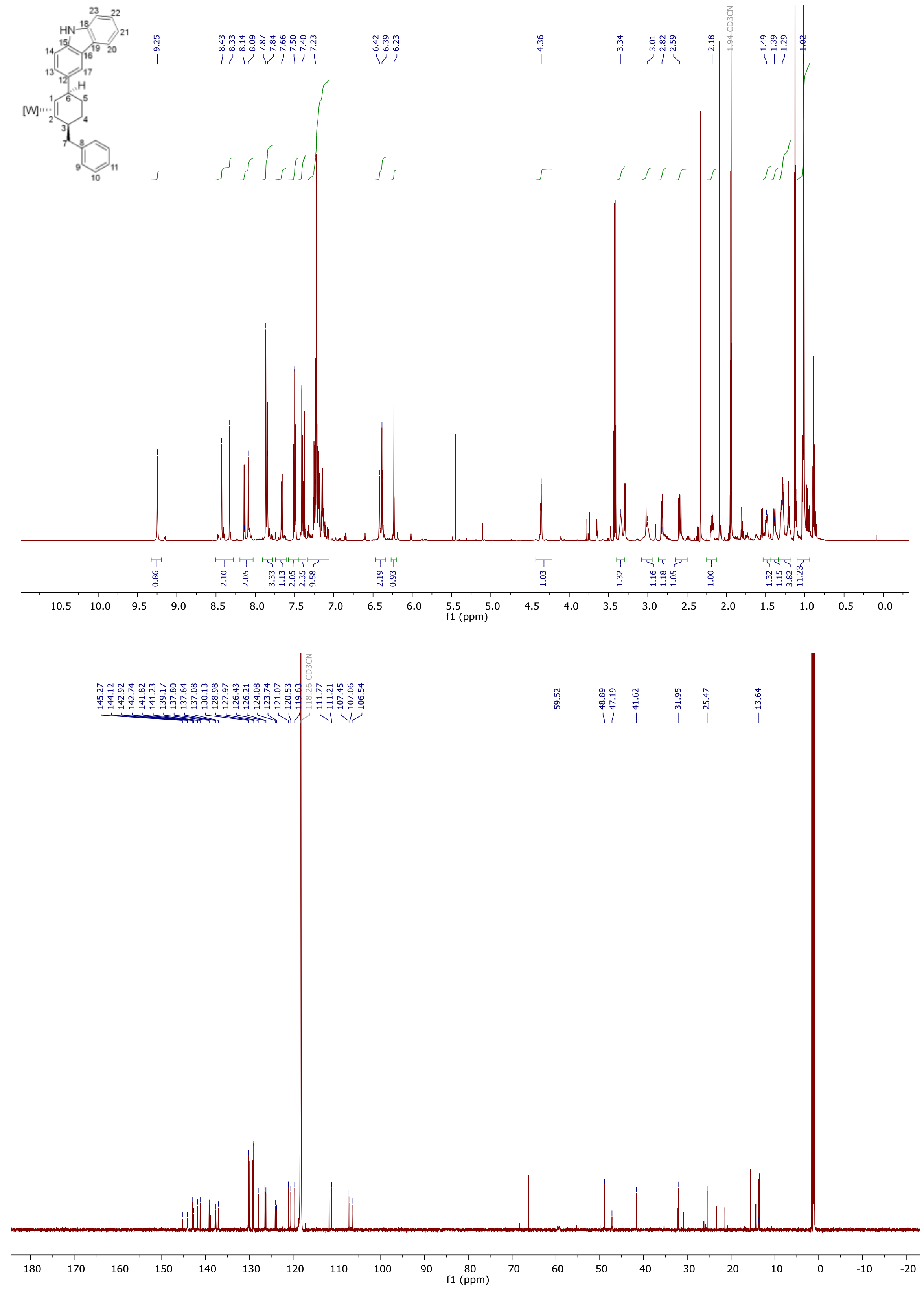

**Fig. S54.** Compound *cis*-60D $^{1}H$ NMR (800 MHz, $CD_3CN$, 25 °C, top) and $^{13}C$ NMR (201 MHz, $CD_3CN$, 25 °C, bottom).

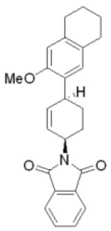


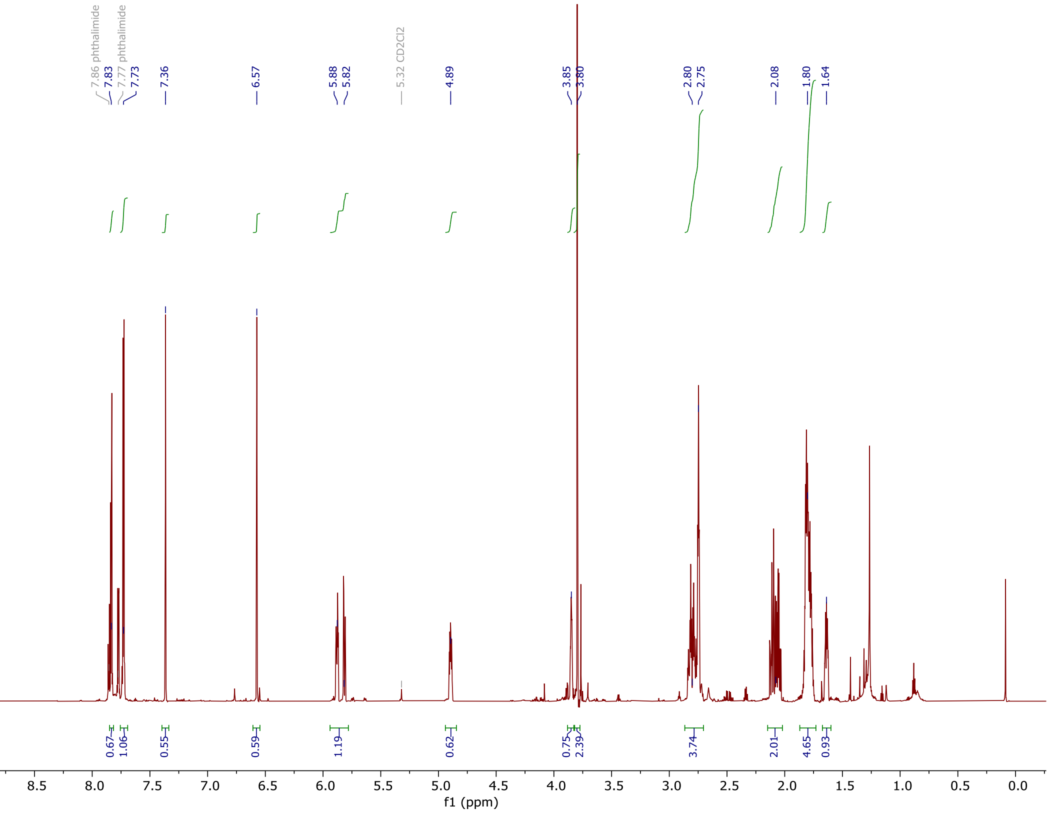


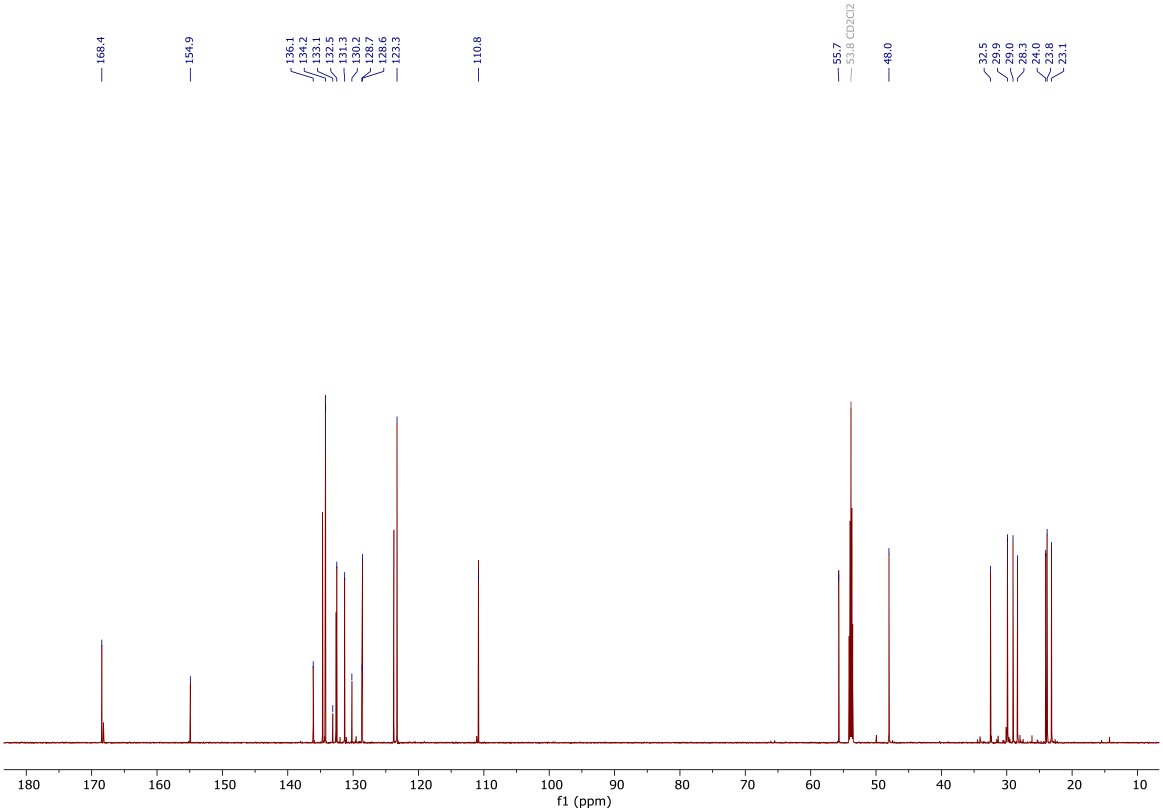


**Fig. S55.** Compound 65 $^{1}$H NMR (800 MHz, $CD_2Cl_2$, 25 °C, top) and $^{13}$C NMR (201 MHz, $CD_2Cl_2$, 25 °C, bottom).

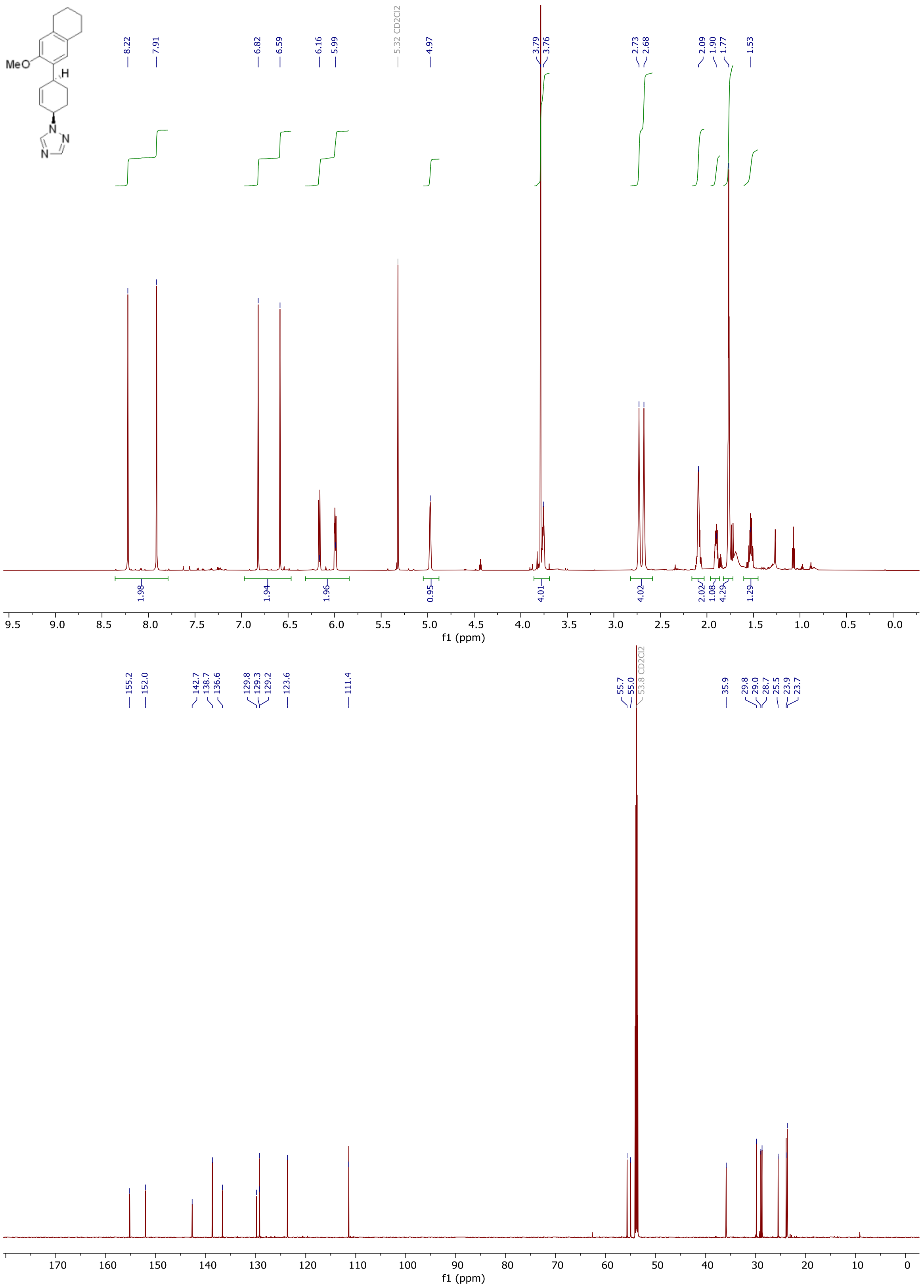


**Fig. S56.** Compound 66 $^{1}$H NMR (800 MHz, $CD_2Cl_2$, 25 °C, top) and $^{13}$C NMR (201 MHz, $CD_2Cl_2$, 25 °C, bottom).

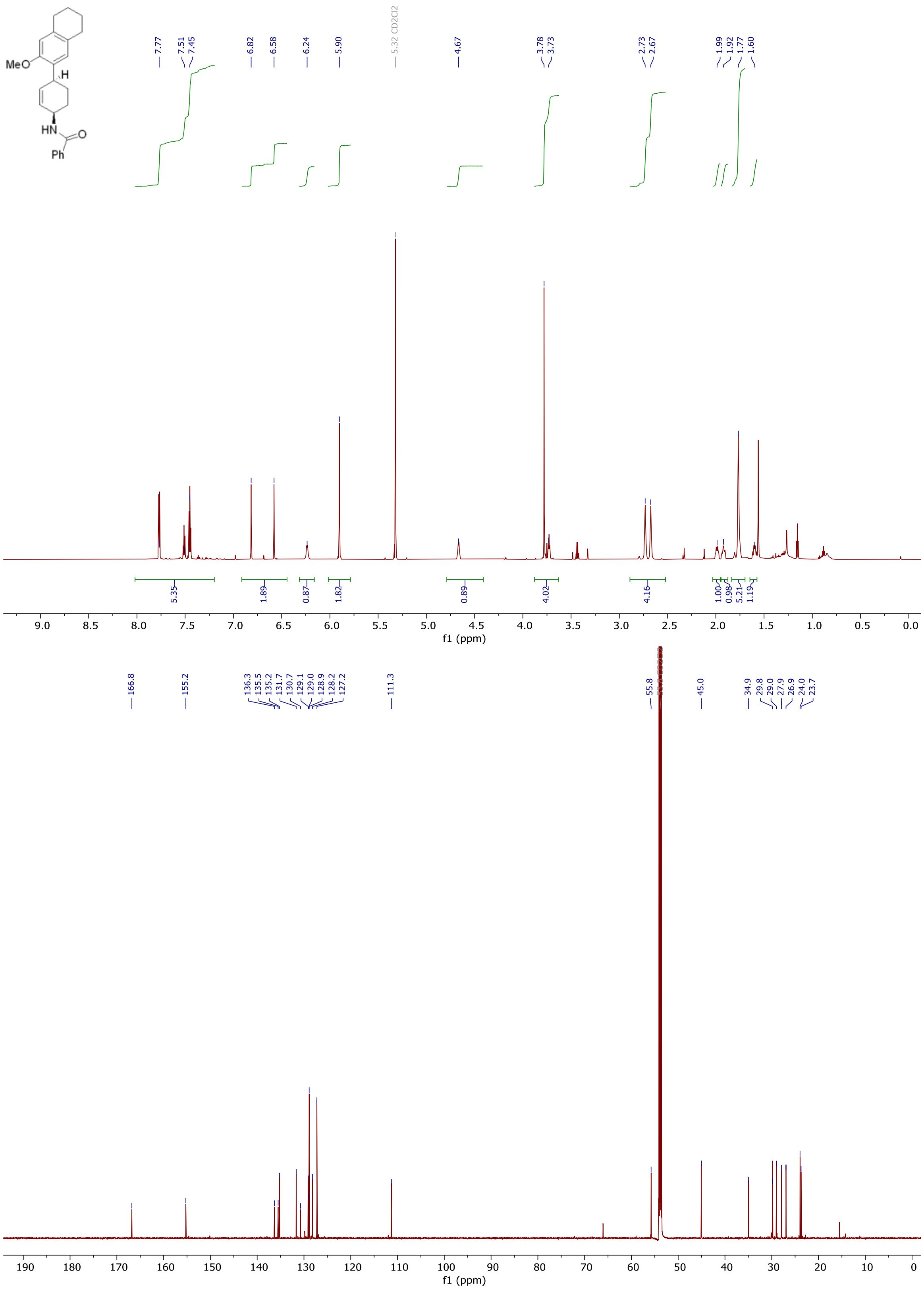

**Fig. S57.** Compound 67 $^{1}H$ NMR (800 MHz, $CD_2Cl_2$, 25 °C, top) and $^{13}C$ NMR (201 MHz, $CD_2Cl_2$, 25 °C, bottom).

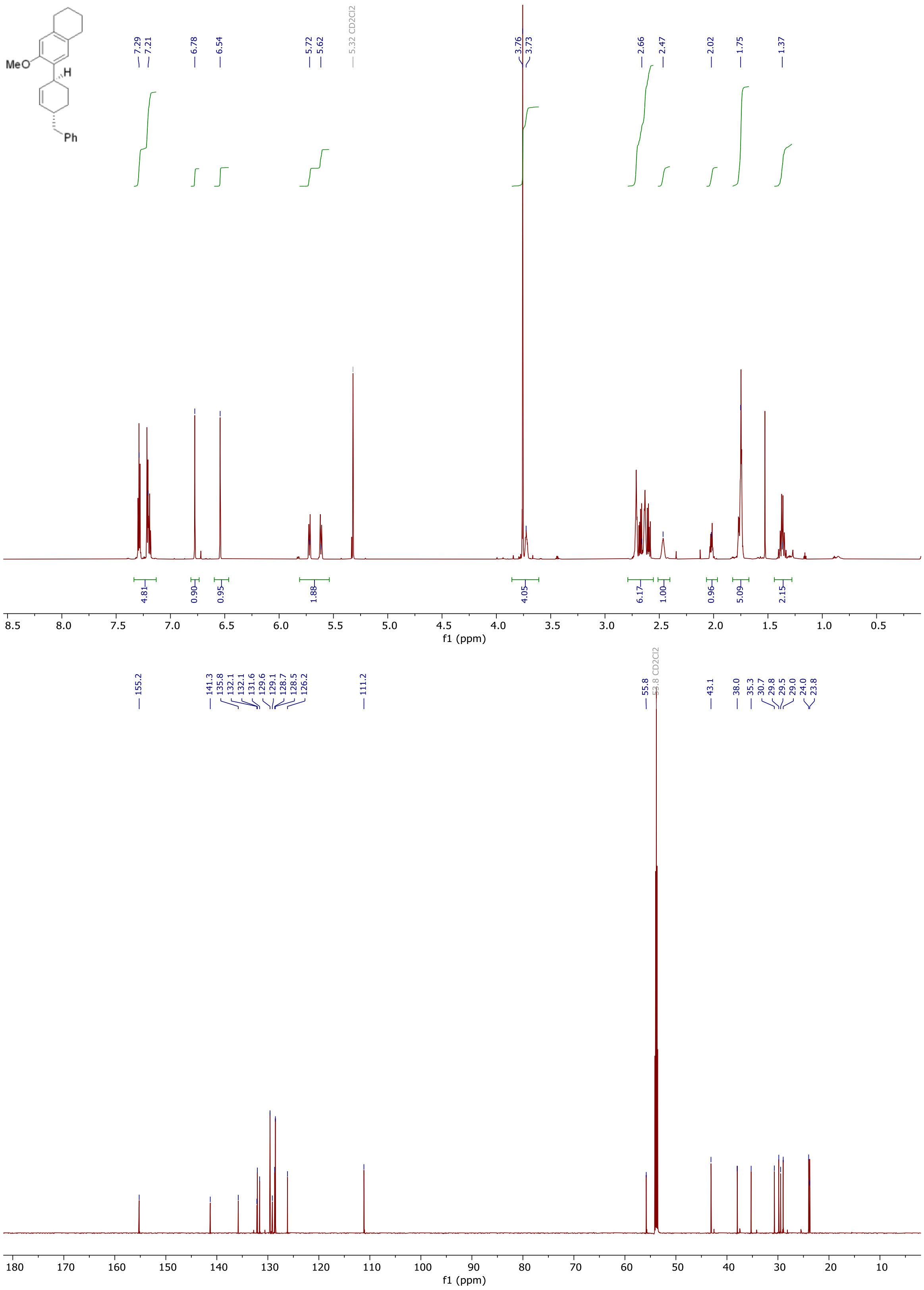


**Fig. S58.** Compound 69 $^{1}H$ NMR (800 MHz, $CD_2Cl_2$, 25 °C, top) and $^{13}C$ NMR (201 MHz, $CD_2Cl_2$, 25 °C, bottom).

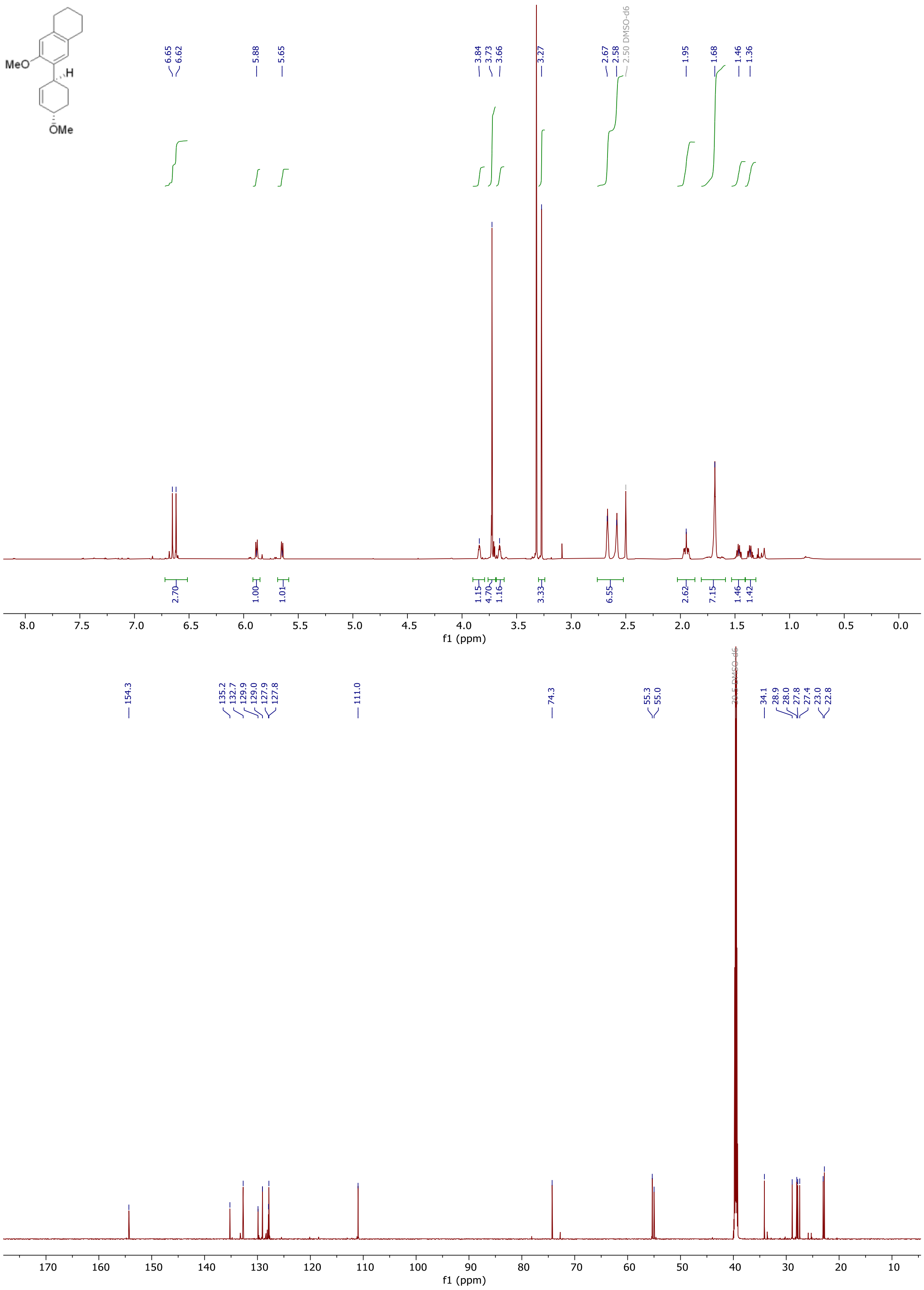

**Fig. S59.** Compound 71 $^{1}$H NMR (800 MHz, $(CD_3)_2SO$, 25 °C, top) and $^{13}$C NMR (201 MHz, $(CD_3)_2SO$, 25 °C, bottom).

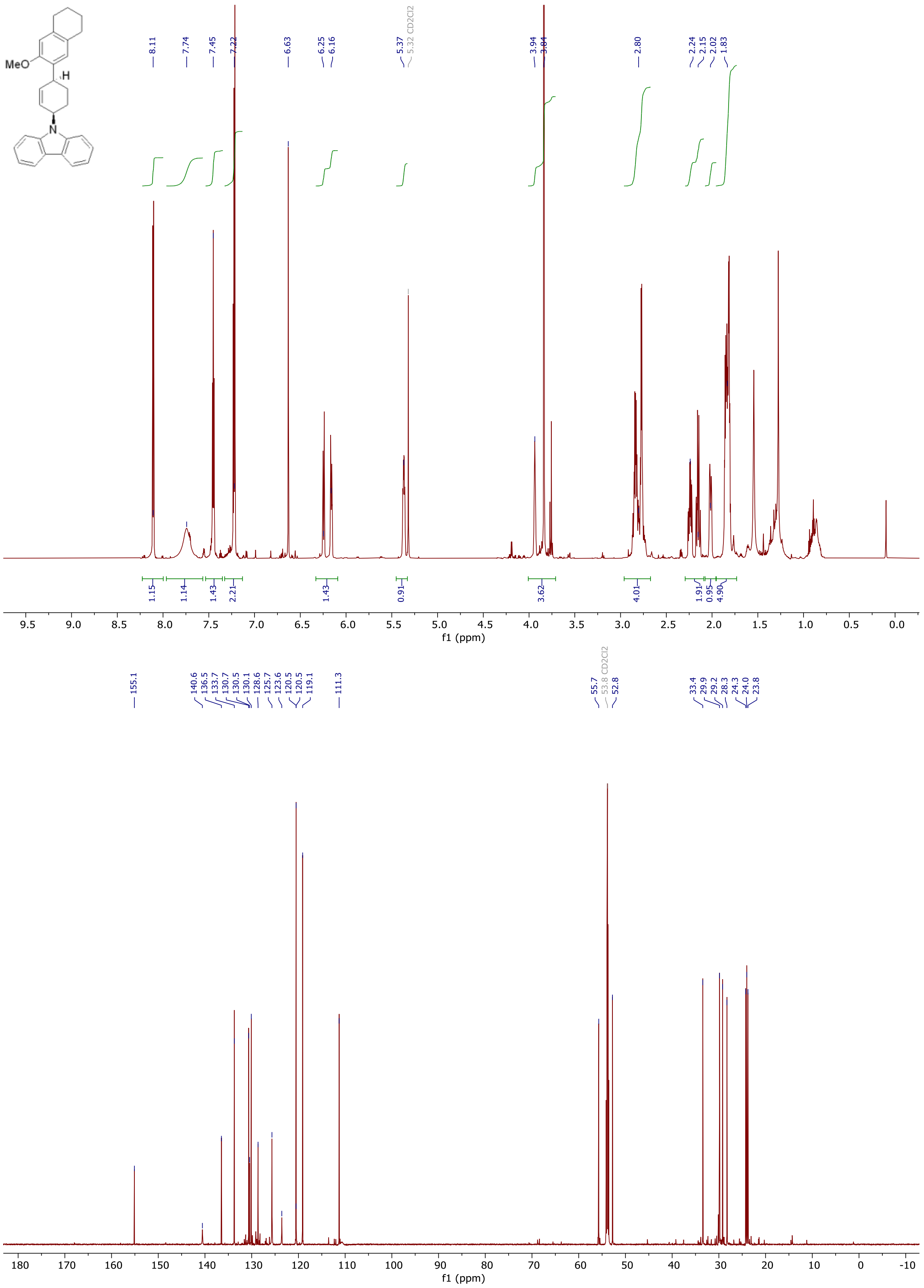


**Fig. S60.** Compound 75 $^{1}H$ NMR (800 MHz, $CD_2Cl_2$, 25 °C, top) and $^{13}C$ NMR (201 MHz, $CD_2Cl_2$, 25 °C, bottom).

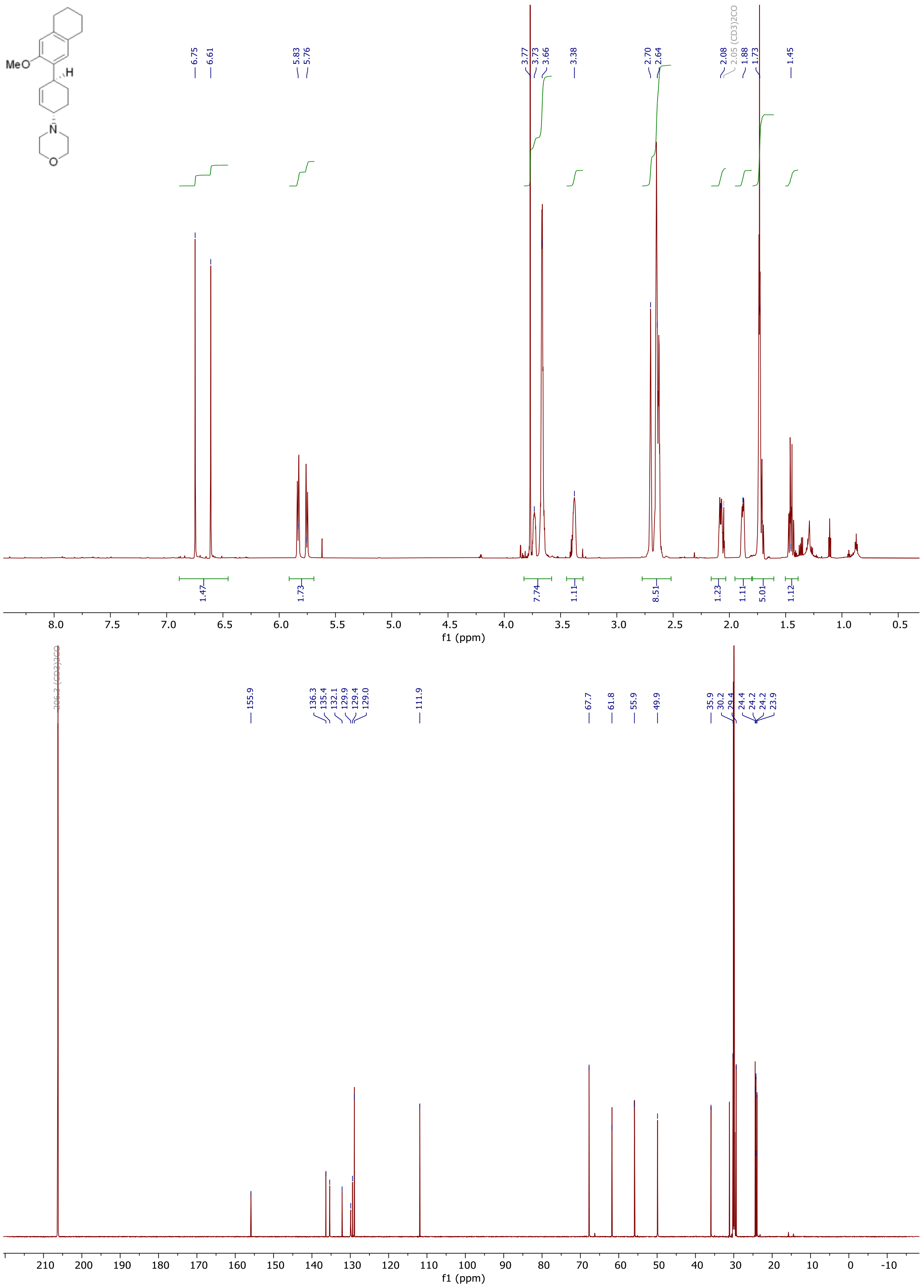


**Fig. S61.** Compound 76 $^{1}H$ NMR (800 MHz, $(CD_3)_2CO$, 25 °C, top) and $^{13}C$ NMR (201 MHz, $(CD_3)_2CO$, 25 °C, bottom).

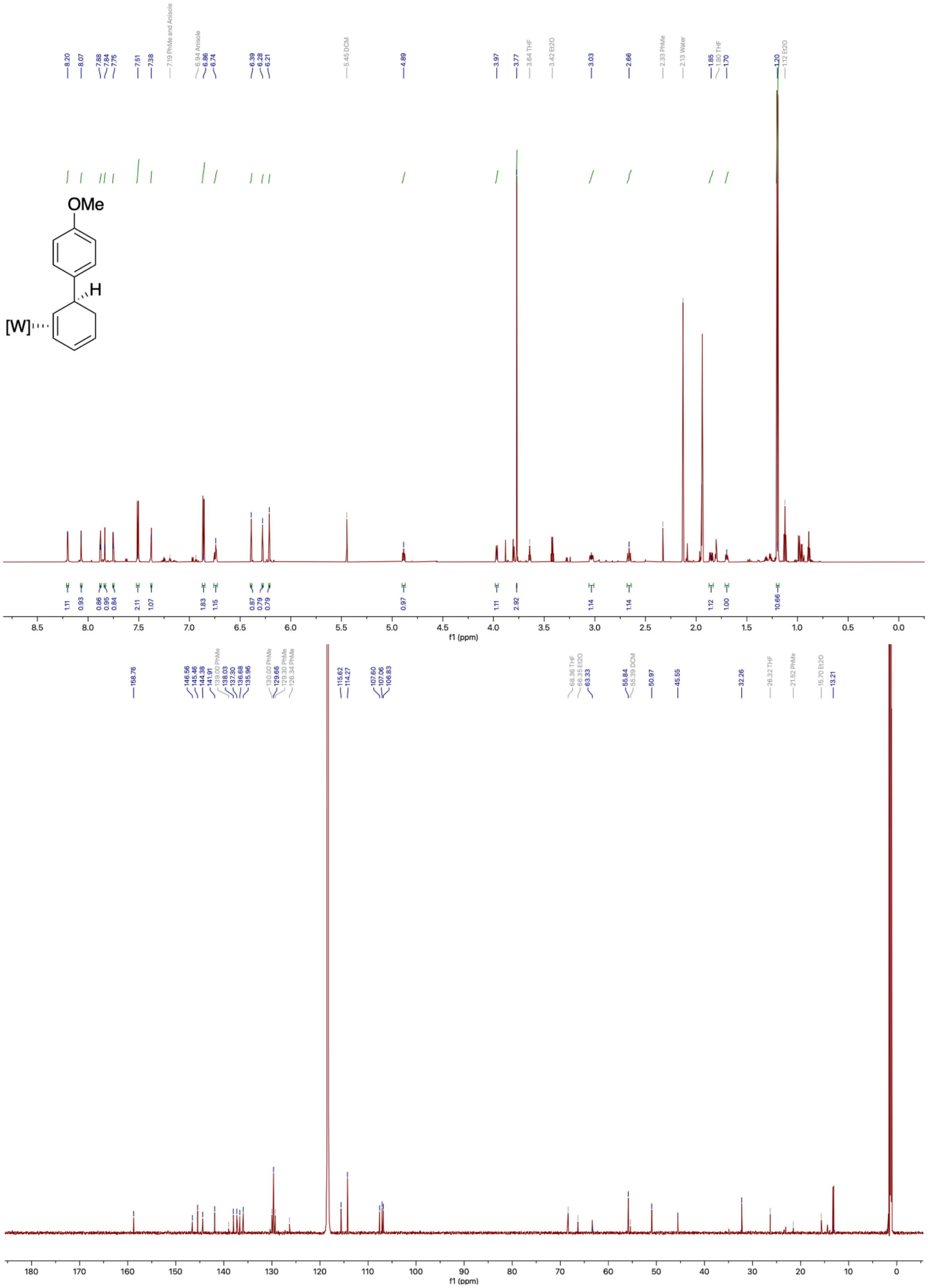

**Fig. S62.** Compound 87D $^{1}H$ NMR (800 MHz, $CD_3CN$, 25 °C, top) and $^{13}C$ NMR (201 MHz, $CD_3CN$, 25 °C, bottom).

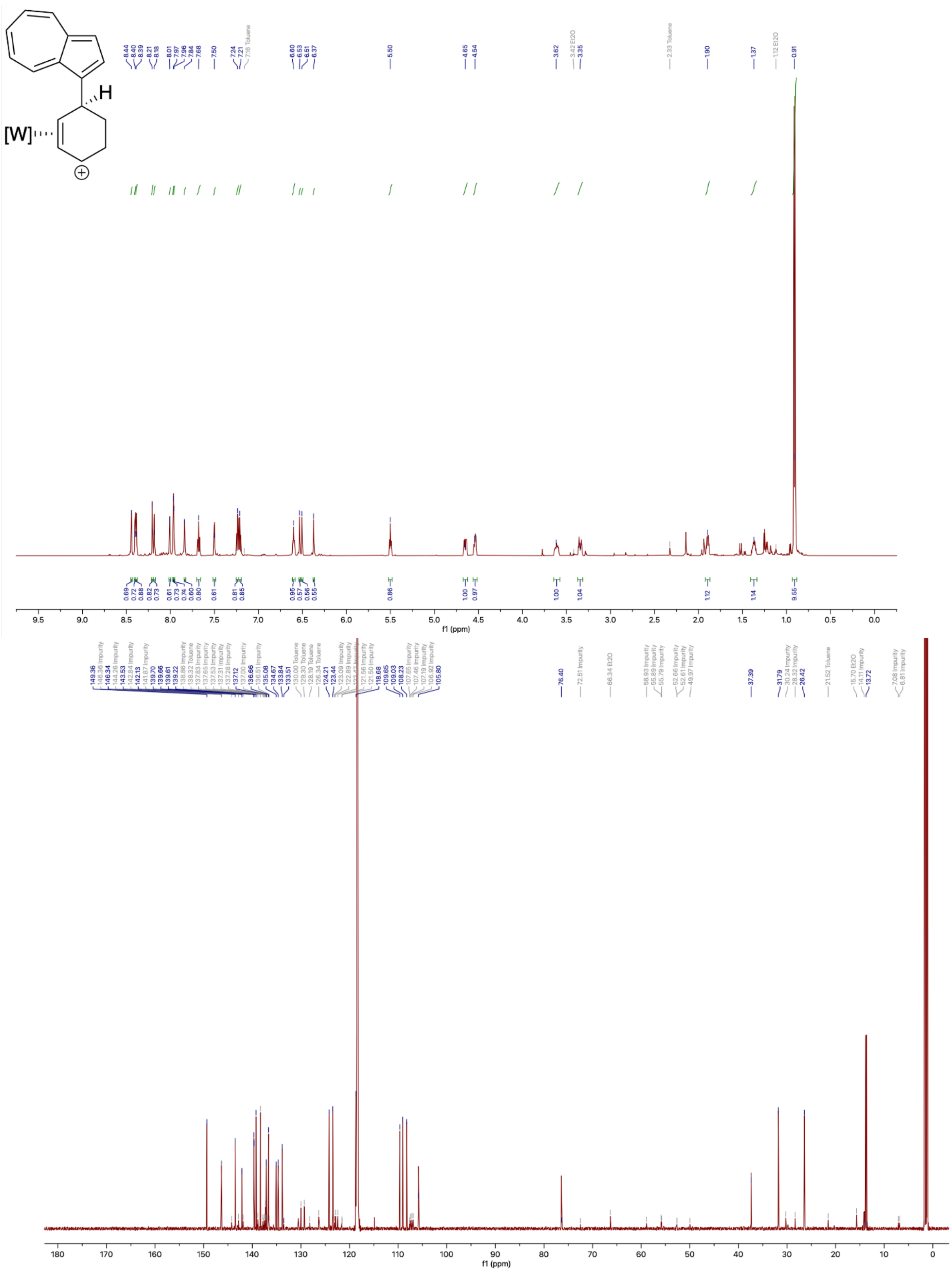

**Fig. S63.** Compound 79D $^{1}$H NMR (800 MHz, $CD_3CN$, 25 °C, top) and $^{13}$C NMR (201 MHz, $CD_3CN$, 25 °C, bottom).

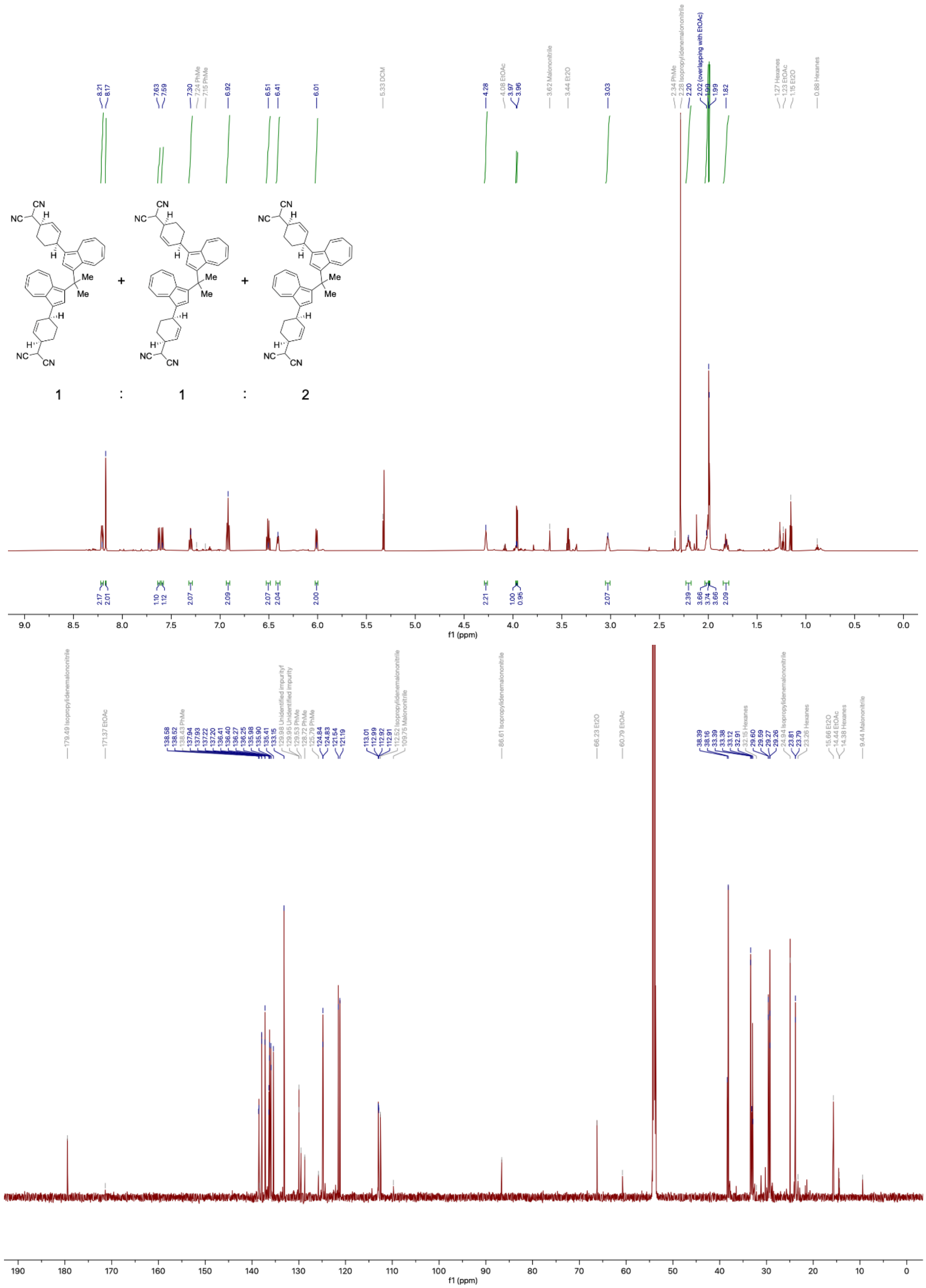


**Fig. S64.** Compound 80 $^{1}H$ NMR (800 MHz, $CD_2Cl_2$, 25 °C, top) and $^{13}C$ NMR (201 MHz, $CD_2Cl_2$, 25 °C, bottom).

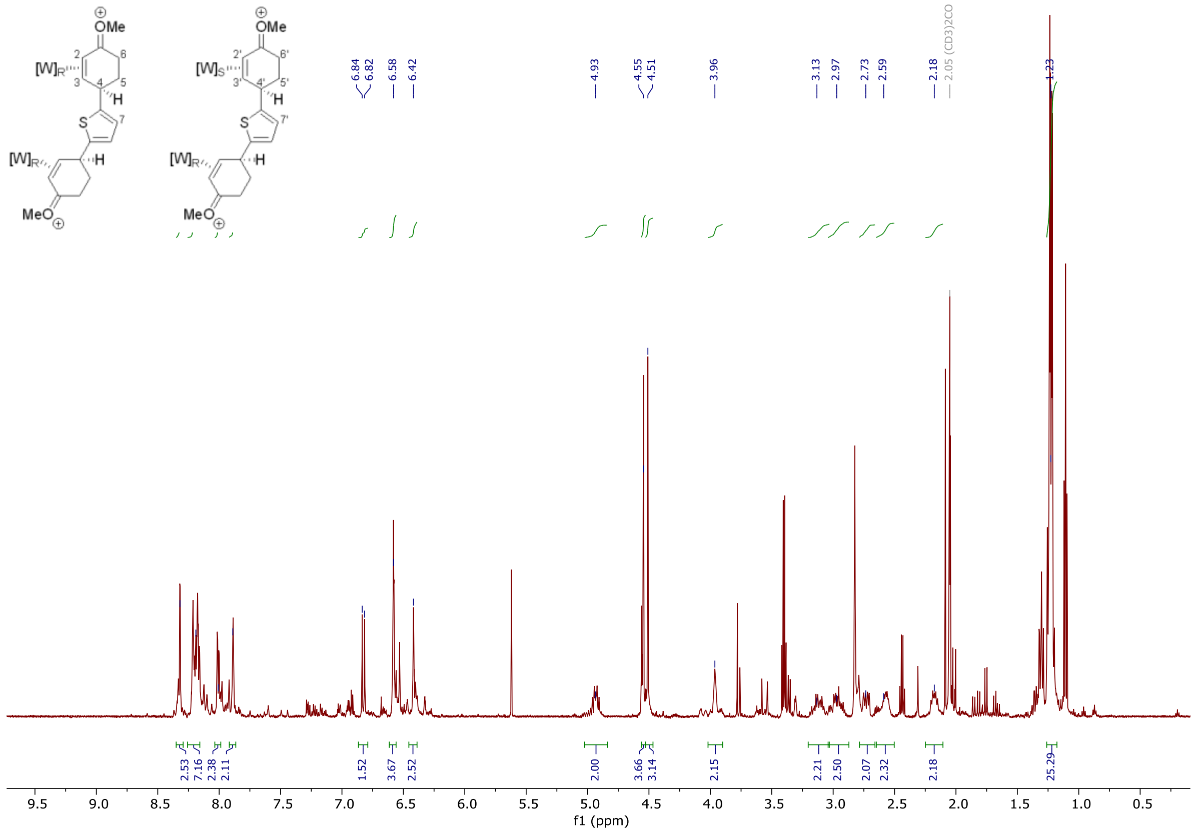


**Fig. S65.** Compound 78 $^{1}$H NMR (600 MHz, $(CD_3)_2CO$, 25 °C).

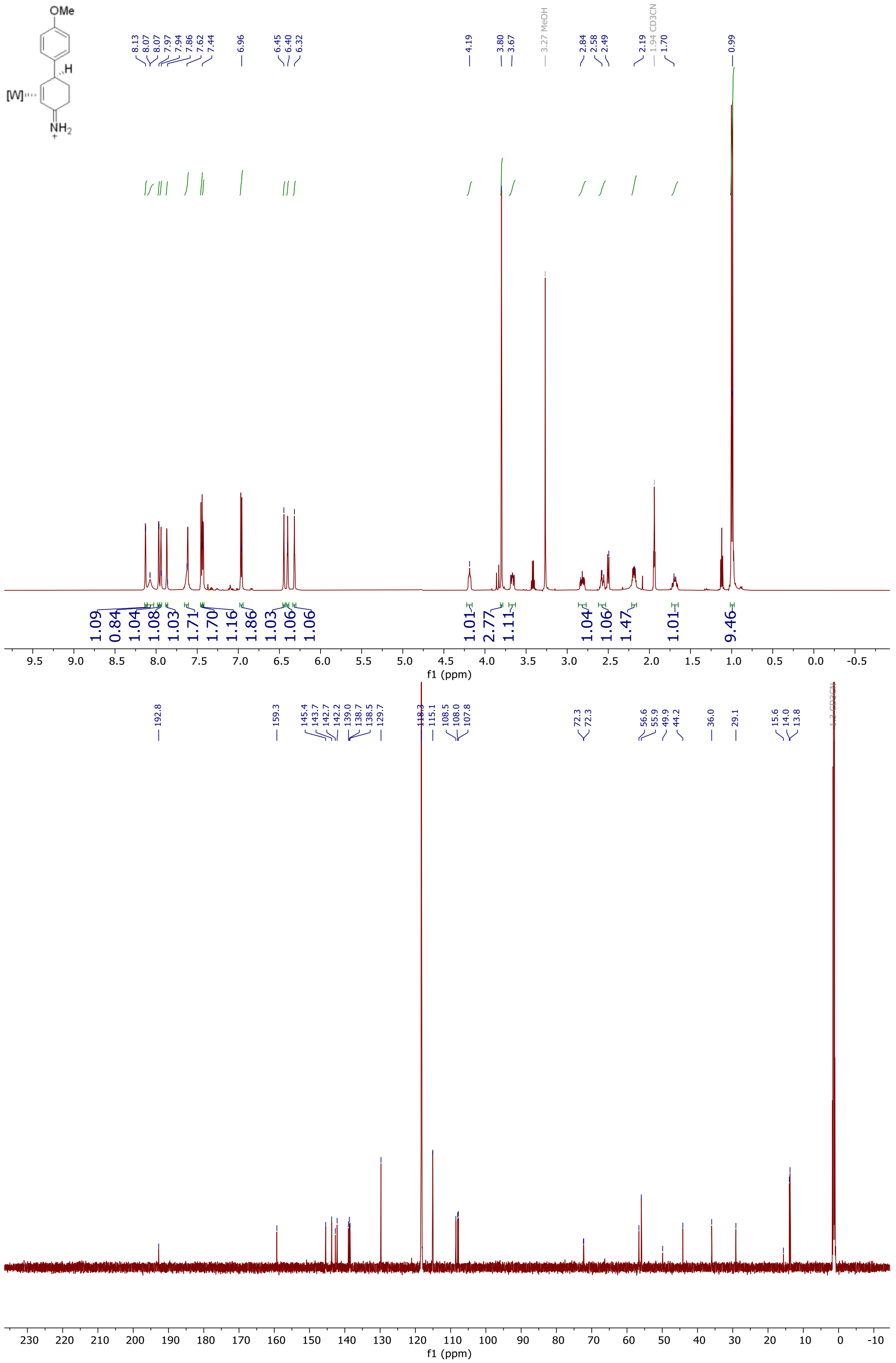

**Fig. S66.** Compound 84D $^{1}H$ NMR (600 MHz, $CD_3CN$, 25 °C, top) and $^{13}C$ NMR (201 MHz, $CD_3CN$, 25 °C, bottom).

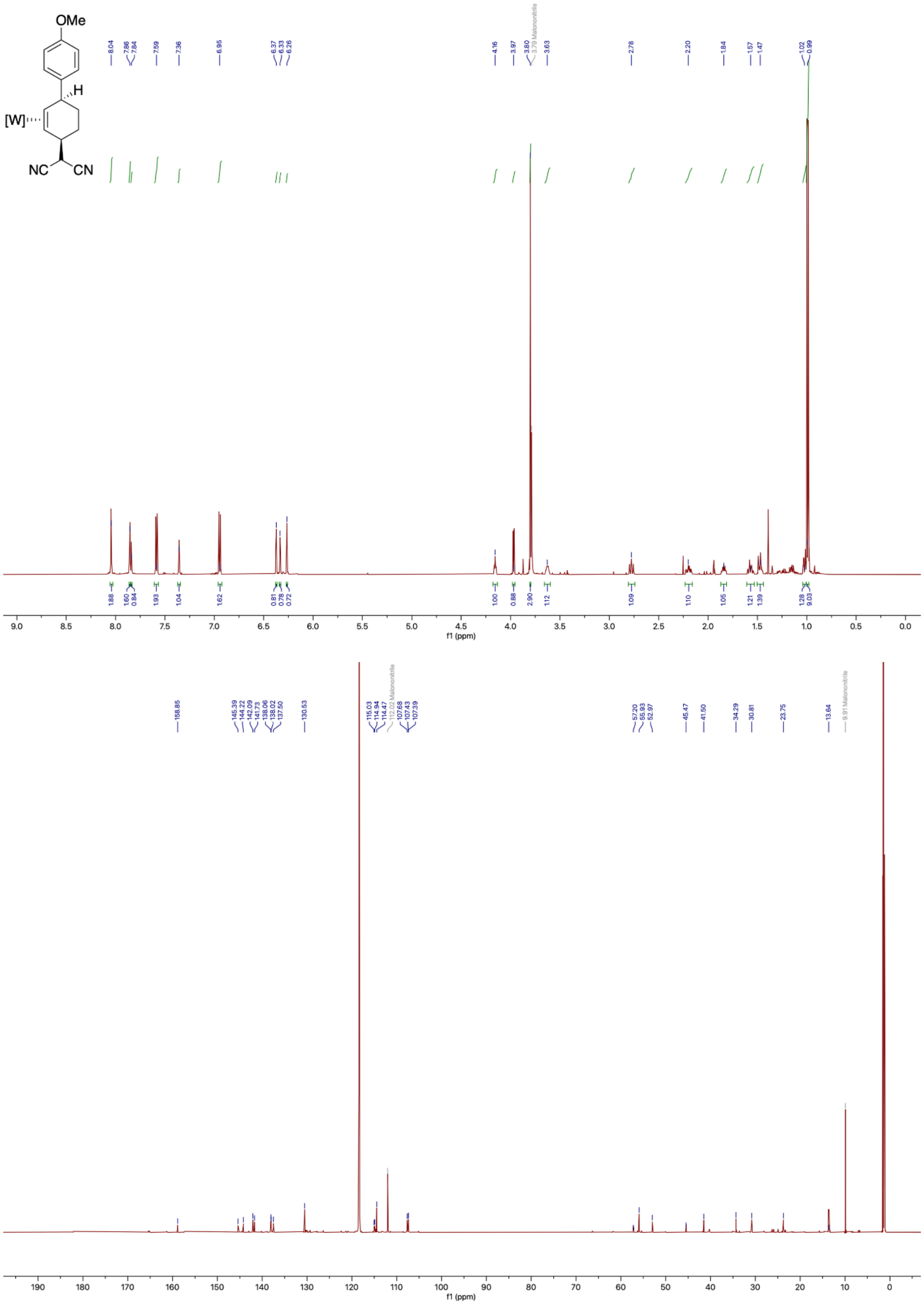


**Fig. S67.** Compound 88D $^{1}H$ NMR (800 MHz, $CD_3CN$, 25 °C, top) and $^{13}C$ NMR (201 MHz, $CD_3CN$, 25 °C, bottom).

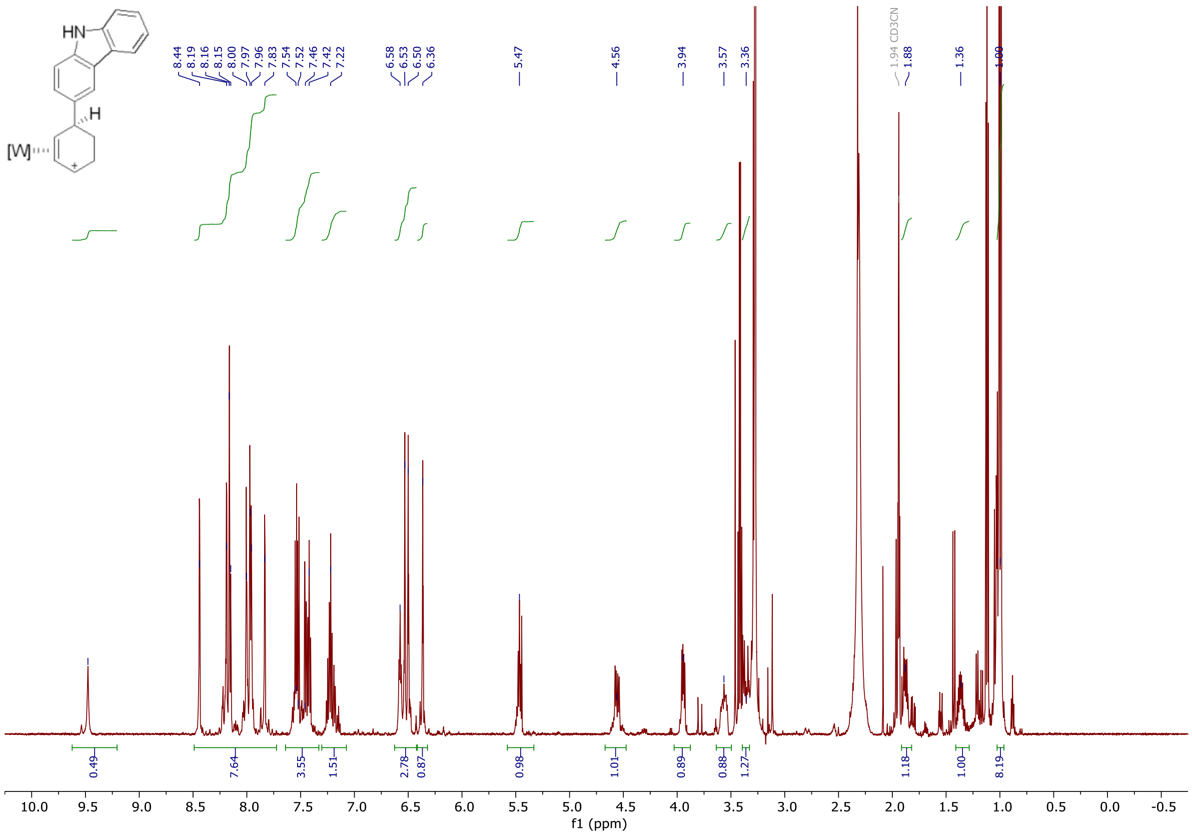


**Fig. S68.** Compound 91D $^{1}H$ NMR (600 MHz, $CD_3CN$, 25 °C).

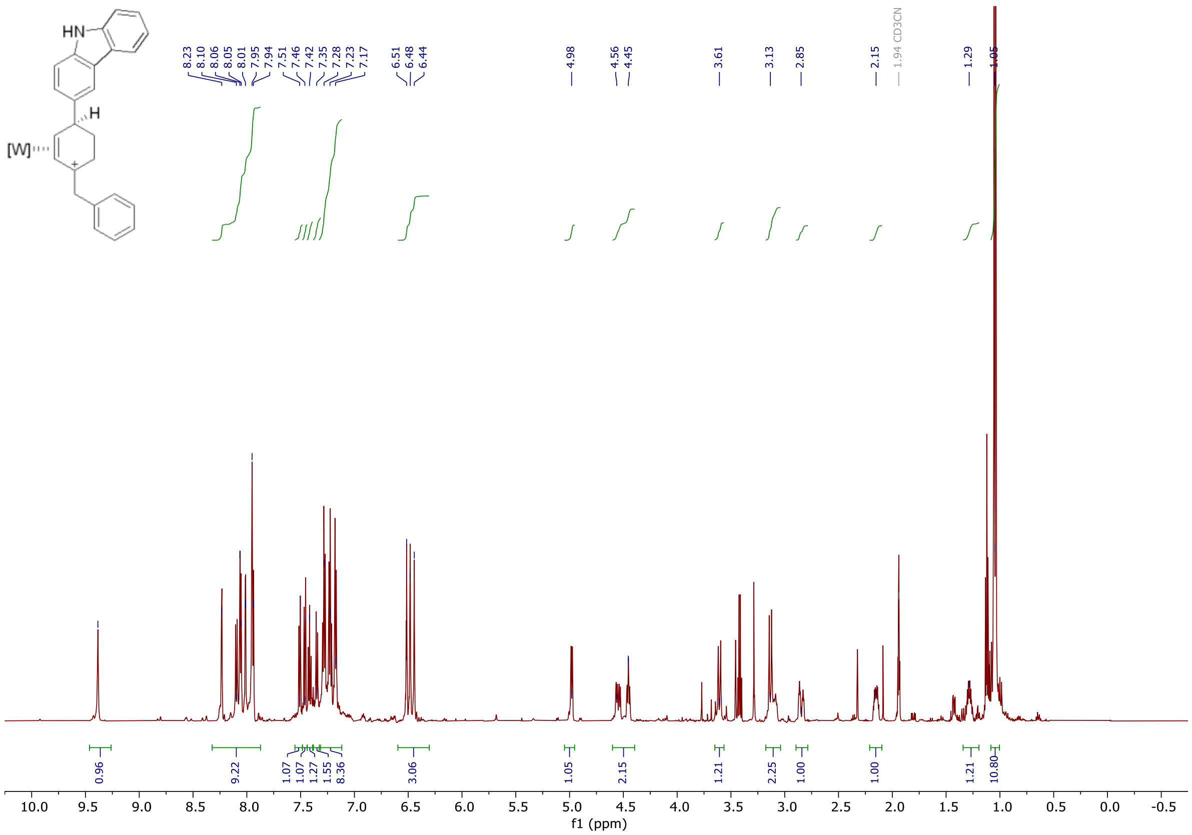

**Fig. S69.** Compound 90D $^1H$ NMR (600 MHz, $CD_3CN$, 25 °C).

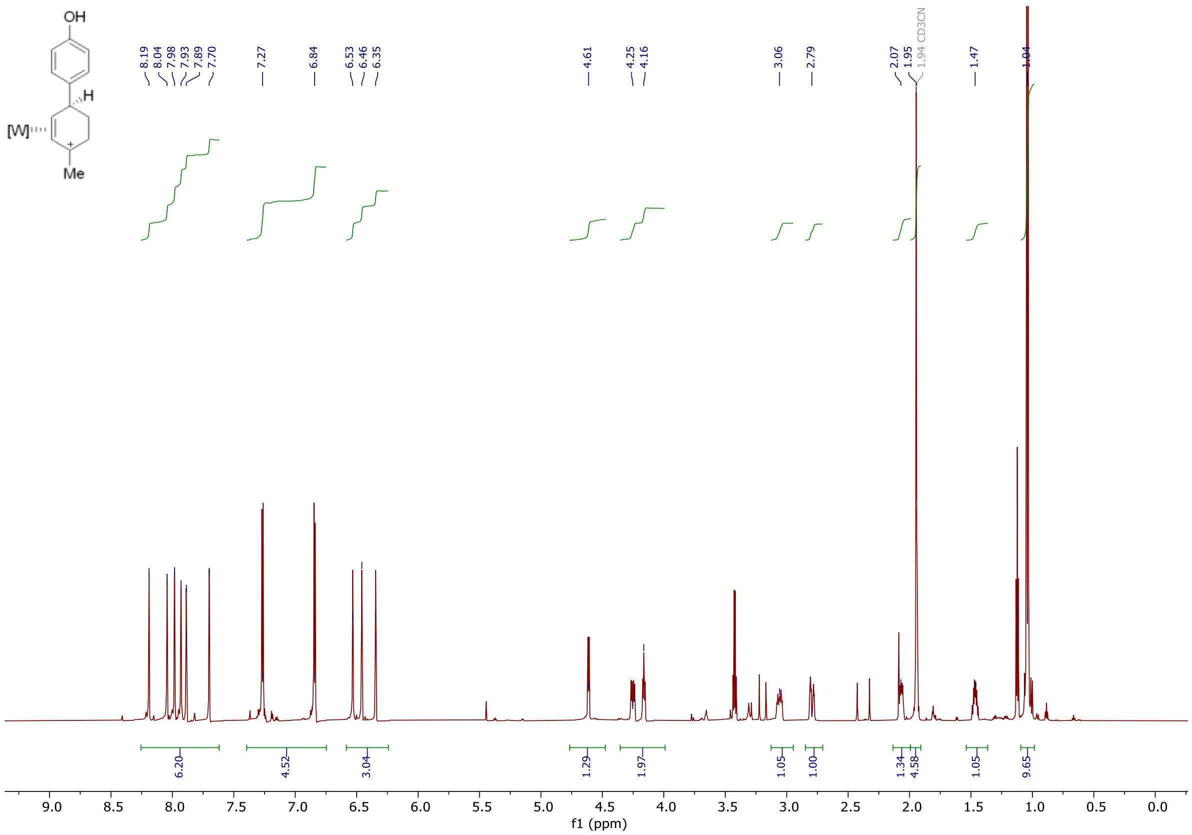


**Fig. S70.** Compound 89D $^{1}H$ NMR (600 MHz, $CD_3CN$, 25 °C).

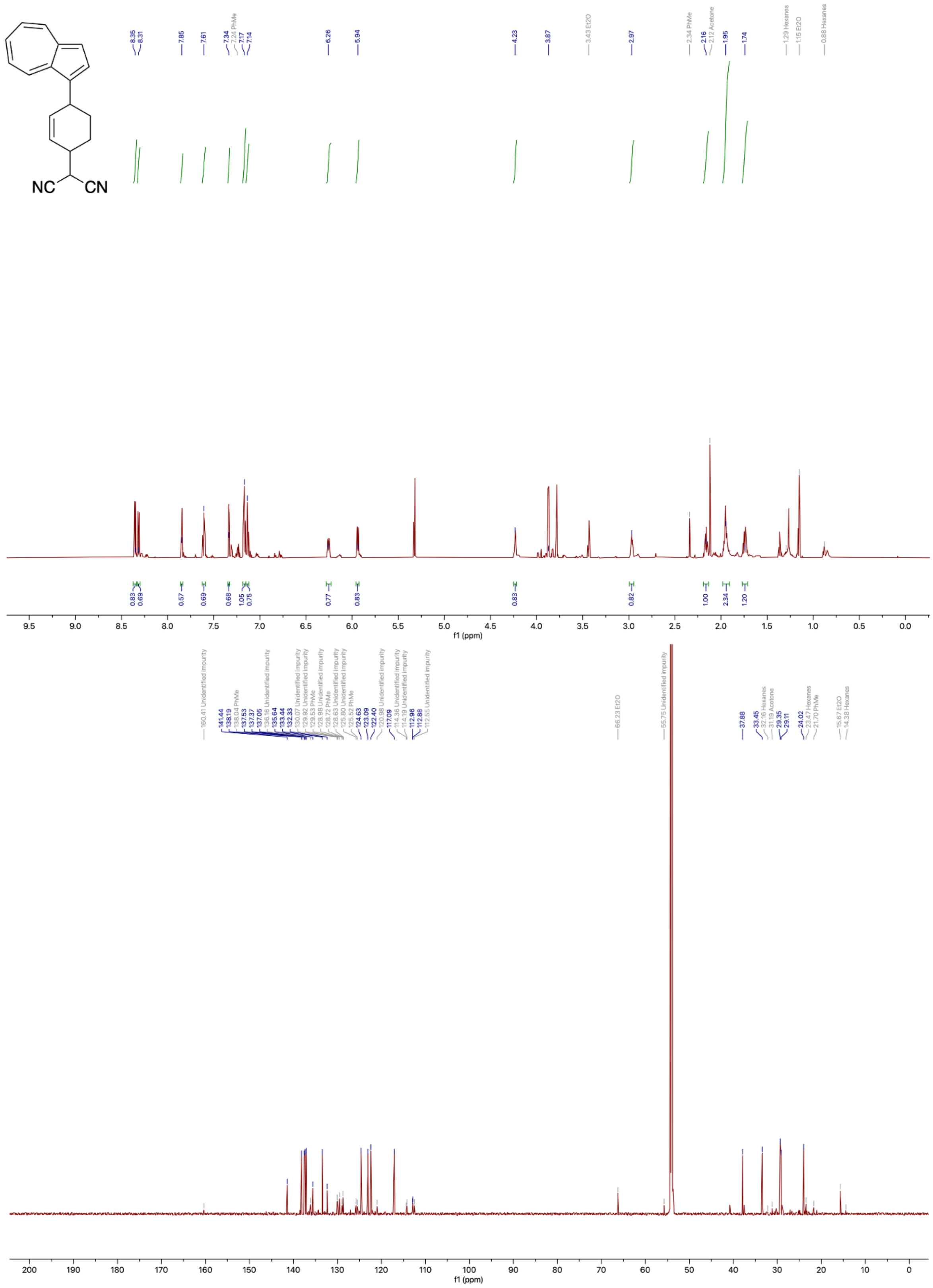


**Fig. S71.** Compound 81 $^{1}$H NMR (800 MHz, $CD_2Cl_2$, 25 °C, top) and $^{13}$C NMR (201 MHz, $CD_2Cl_2$, 25 °C, bottom).

## DFT Calculations

Computational Methods: Ground-state structures were optimized at the M06 level of theory using the 6-31G** [LANL2DZ for W] basis set in Gaussian 16. Previous literature demonstrates that this functional and basis set choice accurately corroborates experimental results.[17] Solvent effects of dichloromethane were modeled using SMD. Gaussian's default criteria was used for optimization, vibrational frequency analysis verified that structures were minima and thermal free energy corrections were applied.

| Compound | Structure | Electronic Energy (Hartree) | Free Energy (Hartree) | Relative Free Energy (kcal/mol) |
|---|---|---|---|---|
| 2H-Anisolium | 2P | -1707.672751 | -1707.259281 | 4.30 |
| | 2D | -1707.680460 | -1707.266138 | 0.00 |
| | | | | |
| Anisole Double Protonation | 3P | -1708.107304 | -1707.677004 | 2.71 |
| | 3D | -1708.109359 | -1707.681321 | 0.00 |

**Table S1.** Electronic and Free Energies for the optimized structures of 2H-Anisolium (2P/2D) and the double protonation of Anisole (3P/3D) in $CH_2Cl_2$.

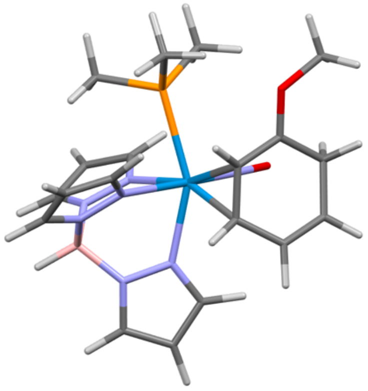

**Fig. S72.** DFT geometry optimization and coordinates for 2P.

| | | | |
|---|---|---|---|
| O | -1.462700 | 1.275200 | -2.526000 |
| N | -0.939600 | 0.872300 | -1.516600 |
| W | -0.132900 | 0.244900 | -0.071700 |
| C | -3.413200 | 1.872100 | -0.176100 |
| C | -3.053600 | 0.667200 | 0.615500 |
| C | -1.876800 | 0.568600 | 1.373100 |
| C | -0.941000 | 1.695500 | 1.355700 |
| C | -1.452000 | 2.979900 | 0.855500 |
| H | -1.884700 | -0.168900 | 2.173800 |
| H | -0.284900 | 1.755400 | 2.226700 |
| H | -0.877500 | 3.876000 | 1.084800 |
| H | -3.339400 | 1.623800 | -1.253100 |

| | | | |
|---|---|---|---|
| H | -4.481100 | 2.080100 | -0.013000 |
| O | -3.892400 | -0.337500 | 0.694900 |
| C | -5.105500 | -0.365700 | -0.074700 |
| H | -4.910300 | -0.145300 | -1.128400 |
| H | -5.487800 | -1.382100 | 0.026500 |
| H | -5.830500 | 0.343700 | 0.336100 |
| C | -2.580100 | 3.073900 | 0.144800 |
| H | -2.937700 | 4.028400 | -0.232800 |
| N | 1.546300 | 1.715000 | -0.038800 |
| N | 2.815900 | 1.323500 | 0.237600 |
| N | 1.033200 | -0.644800 | 1.661900 |
| N | 2.384100 | -0.753600 | 1.584900 |
| N | 1.437300 | -0.642800 | -1.344500 |
| N | 2.703600 | -0.854800 | -0.915300 |
| C | 3.645900 | 2.377300 | 0.120800 |
| C | 2.904300 | 3.487800 | -0.238900 |
| C | 1.592800 | 3.017600 | -0.333100 |
| C | 2.858900 | -1.364600 | 2.683300 |
| C | 1.794400 | -1.670700 | 3.513500 |
| C | 0.675300 | -1.197000 | 2.830200 |
| C | 3.432900 | -1.382500 | -1.914600 |
| C | 2.620200 | -1.531800 | -3.026600 |
| C | 1.379200 | -1.038800 | -2.622400 |
| B | 3.170200 | -0.173900 | 0.395200 |
| H | 4.348600 | -0.322800 | 0.556900 |
| H | 4.706900 | 2.254800 | 0.299800 |
| H | 3.260400 | 4.493600 | -0.411000 |
| H | 0.684600 | 3.546100 | -0.598000 |
| H | 3.922600 | -1.532900 | 2.796800 |
| H | 1.825100 | -2.159600 | 4.476700 |
| H | -0.365500 | -1.229300 | 3.130600 |
| H | 4.483300 | -1.600300 | -1.766200 |
| H | 2.891700 | -1.929100 | -3.994300 |
| H | 0.449500 | -0.955300 | -3.175100 |
| C | 0.258400 | -3.399600 | -0.482700 |
| H | -0.200000 | -4.395500 | -0.501500 |
| H | 0.822900 | -3.248900 | -1.407300 |
| H | 0.948100 | -3.341300 | 0.368100 |
| C | -2.119500 | -2.371500 | -1.751200 |
| H | -1.545500 | -2.142800 | -2.656600 |
| H | -2.486400 | -3.403000 | -1.809300 |
| H | -2.971900 | -1.683400 | -1.702600 |
| P | -1.056400 | -2.142700 | -0.286200 |
| C | -2.011600 | -2.897300 | 1.077900 |
| H | -2.318300 | -3.903900 | 0.769100 |
| H | -1.373300 | -2.985100 | 1.963600 |

| | | | |
|---|---|---|---|
| H | -2.898800 | -2.311400 | 1.325200 |

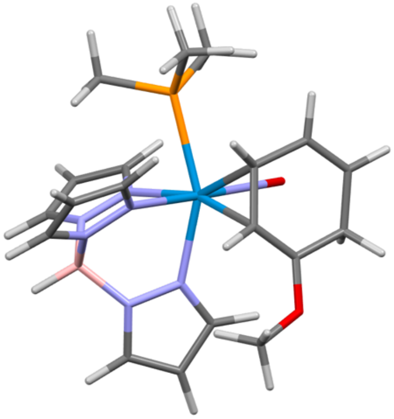

**Fig. S73.** DFT geometry optimization and coordinates for 2D.

| | | | |
|---|---|---|---|
| O | -1.396200 | -1.177600 | -2.724200 |
| N | -0.918900 | -0.830300 | -1.678100 |
| W | -0.195800 | -0.299100 | -0.151200 |
| N | -0.470700 | 1.853100 | -0.551500 |
| N | 0.455900 | 2.758200 | -0.138800 |
| N | 0.850200 | 0.402100 | 1.726100 |
| N | 1.661700 | 1.487500 | 1.683200 |
| N | 1.662100 | 0.305800 | -1.170600 |
| N | 2.407000 | 1.344900 | -0.715200 |
| C | 0.115200 | 3.978900 | -0.594600 |
| C | -1.061100 | 3.875900 | -1.316100 |
| C | -1.378700 | 2.517600 | -1.275400 |
| C | 2.225800 | 1.687400 | 2.886400 |
| C | 1.772500 | 0.707500 | 3.753200 |
| C | 0.913000 | -0.070800 | 2.978800 |
| C | 3.420600 | 1.573700 | -1.569700 |
| C | 3.346100 | 0.659300 | -2.606900 |
| C | 2.215500 | -0.104500 | -2.318000 |
| B | 1.840400 | 2.284200 | 0.383200 |
| H | 2.569300 | 3.219700 | 0.555300 |
| H | 0.745800 | 4.833100 | -0.381300 |
| H | -1.604000 | 4.668100 | -1.811500 |
| H | -2.215400 | 1.989500 | -1.719200 |
| H | 2.907600 | 2.514800 | 3.038600 |
| H | 2.025300 | 0.578500 | 4.795900 |
| H | 0.338500 | -0.944500 | 3.264600 |
| H | 4.114700 | 2.384100 | -1.384400 |
| H | 4.007700 | 0.567900 | -3.456400 |
| H | 1.773400 | -0.924400 | -2.872200 |
| C | 3.019900 | -2.062900 | 0.304300 |

| | | | |
|---|---|---|---|
| H | 3.548000 | -3.000100 | 0.515800 |
| H | 3.435100 | -1.617600 | -0.604400 |
| H | 3.174700 | -1.368300 | 1.139400 |
| C | 1.108800 | -3.543800 | -1.294700 |
| H | 1.410700 | -3.039700 | -2.218600 |
| H | 1.750500 | -4.418700 | -1.136800 |
| H | 0.068300 | -3.871100 | -1.403100 |
| P | 1.230800 | -2.404500 | 0.124900 |
| C | 0.962200 | -3.519300 | 1.548900 |
| H | 1.696000 | -4.332200 | 1.492900 |
| H | 1.111200 | -2.979900 | 2.490500 |
| H | -0.041700 | -3.953500 | 1.536000 |
| C | -1.504400 | -1.564800 | 1.114600 |
| C | -2.049300 | -0.208400 | 1.197800 |
| C | -3.097600 | 0.163700 | 0.366000 |
| C | -3.757500 | -0.777300 | -0.584100 |
| H | -1.063500 | -1.915600 | 2.048800 |
| H | -1.788500 | 0.429300 | 2.039700 |
| H | -4.842300 | -0.629300 | -0.479700 |
| H | -3.530300 | -0.468700 | -1.619800 |
| C | -2.336000 | -2.556100 | 0.406500 |
| H | -2.081800 | -3.609700 | 0.526000 |
| O | -3.658800 | 1.352300 | 0.374700 |
| C | -3.260700 | 2.324900 | 1.352100 |
| H | -3.801400 | 3.236900 | 1.096800 |
| H | -3.551100 | 1.985800 | 2.352000 |
| H | -2.181800 | 2.511100 | 1.314100 |
| C | -3.368400 | -2.207100 | -0.368200 |
| H | -3.954500 | -2.957800 | -0.893000 |

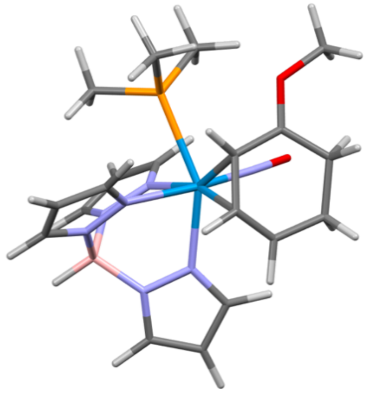

**Fig. S74.** DFT geometry optimization and coordinates for 3P.

| | | | |
|---|---|---|---|
| O | -1.642900 | 1.315900 | -2.331000 |
| N | -1.055600 | 0.929800 | -1.370000 |

| | | | |
|---|---|---|---|
| W | -0.150800 | 0.278900 | 0.022300 |
| N | 1.516900 | 1.712900 | -0.034000 |
| N | 2.792800 | 1.308100 | 0.206800 |
| N | 1.075900 | -0.725000 | 1.620900 |
| N | 2.421600 | -0.833400 | 1.474600 |
| N | 1.288000 | -0.567700 | -1.377200 |
| N | 2.581000 | -0.801300 | -1.033400 |
| C | 3.630900 | 2.334700 | -0.013500 |
| C | 2.894900 | 3.441400 | -0.402800 |
| C | 1.576400 | 2.997500 | -0.417000 |
| C | 2.943700 | -1.517500 | 2.504000 |
| C | 1.917500 | -1.874900 | 3.362000 |
| C | 0.771300 | -1.355100 | 2.769300 |
| C | 3.260200 | -1.203000 | -2.119800 |
| C | 2.395200 | -1.246700 | -3.201800 |
| C | 1.173500 | -0.820800 | -2.691300 |
| B | 3.142100 | -0.201000 | 0.281300 |
| H | 4.324400 | -0.364700 | 0.355300 |
| H | 4.697600 | 2.198700 | 0.114400 |
| H | 3.262600 | 4.427600 | -0.647000 |
| H | 0.672700 | 3.535100 | -0.681300 |
| H | 4.010400 | -1.696800 | 2.553300 |
| H | 1.990100 | -2.426700 | 4.288000 |
| H | -0.249500 | -1.407800 | 3.127600 |
| H | 4.320000 | -1.414100 | -2.048000 |
| H | 2.621600 | -1.532900 | -4.218600 |
| H | 0.223500 | -0.692600 | -3.196200 |
| C | 0.380400 | -3.353500 | -0.533200 |
| H | -0.056700 | -4.357000 | -0.593800 |
| H | 0.960500 | -3.165600 | -1.440400 |
| H | 1.041600 | -3.306300 | 0.339800 |
| C | -2.013200 | -2.363600 | -1.832100 |
| H | -1.453300 | -2.100900 | -2.734800 |
| H | -2.336800 | -3.408400 | -1.906700 |
| H | -2.895700 | -1.718000 | -1.766100 |
| P | -0.984600 | -2.160100 | -0.342900 |
| C | -1.975800 | -2.965000 | 0.960500 |
| H | -2.146000 | -4.001900 | 0.646800 |
| H | -1.431300 | -2.974100 | 1.909700 |
| H | -2.943900 | -2.475900 | 1.092300 |
| C | -3.327500 | 1.983500 | 0.184300 |
| C | -3.037000 | 0.607000 | 0.645800 |
| C | -1.928900 | 0.269700 | 1.474100 |
| C | -1.054900 | 1.349100 | 1.889200 |
| C | -1.080100 | 2.544200 | 1.218300 |
| C | -2.210000 | 3.008700 | 0.378200 |

| | | | |
|---|---|---|---|
| H | -2.041500 | -0.607500 | 2.105900 |
| H | -0.348700 | 1.187700 | 2.700000 |
| H | -0.307300 | 3.274000 | 1.450400 |
| H | -2.605800 | 3.906600 | 0.872400 |
| H | -3.646800 | 1.959200 | -0.864900 |
| H | -1.843500 | 3.365900 | -0.592900 |
| H | -4.231000 | 2.269700 | 0.749000 |
| O | -3.851000 | -0.359400 | 0.367800 |
| C | -4.982900 | -0.177100 | -0.517400 |
| H | -4.631500 | 0.121600 | -1.509700 |
| H | -5.463600 | -1.153500 | -0.563500 |
| H | -5.671400 | 0.563400 | -0.102600 |

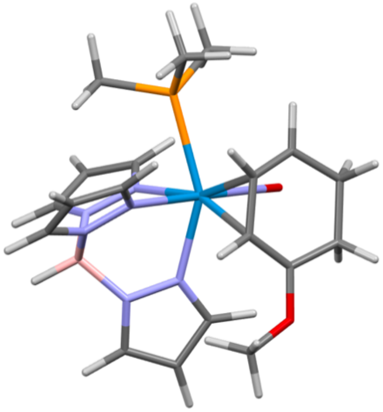

**Fig. S75.** DFT geometry optimization and coordinates for 3D.

| | | | |
|---|---|---|---|
| O | -0.919500 | -1.457300 | -2.772700 |
| N | -0.608800 | -1.076300 | -1.693900 |
| W | -0.097000 | -0.400500 | -0.120500 |
| N | -1.074700 | 1.510400 | -0.540000 |
| N | -0.520800 | 2.692100 | -0.158800 |
| N | 0.686400 | 0.652200 | 1.707000 |
| N | 1.087900 | 1.947400 | 1.624200 |
| N | 1.385500 | 0.781700 | -1.203700 |
| N | 1.763300 | 2.018700 | -0.791100 |
| C | -1.266000 | 3.702000 | -0.642100 |
| C | -2.334800 | 3.176500 | -1.347600 |
| C | -2.160500 | 1.797000 | -1.274500 |
| C | 1.548500 | 2.364000 | 2.813000 |
| C | 1.449700 | 1.318900 | 3.716000 |
| C | 0.903100 | 0.273600 | 2.979300 |
| C | 2.591600 | 2.553600 | -1.702900 |
| C | 2.772400 | 1.646100 | -2.734400 |
| C | 1.980500 | 0.556300 | -2.386700 |
| B | 0.955500 | 2.738200 | 0.319700 |

| | | | |
|---|---|---|---|
| H | 1.327700 | 3.865900 | 0.458500 |
| H | -0.970200 | 4.727600 | -0.459700 |
| H | -3.122300 | 3.715300 | -1.854600 |
| H | -2.765900 | 1.006400 | -1.702900 |
| H | 1.910500 | 3.377600 | 2.930900 |
| H | 1.729300 | 1.316600 | 4.759600 |
| H | 0.658400 | -0.725300 | 3.317200 |
| H | 2.982100 | 3.553400 | -1.559900 |
| H | 3.380500 | 1.763500 | -3.619600 |
| H | 1.817500 | -0.372800 | -2.919800 |
| C | 3.570300 | -0.727300 | 0.314500 |
| H | 4.422200 | -1.380800 | 0.536900 |
| H | 3.776000 | -0.176800 | -0.607400 |
| H | 3.440900 | -0.018200 | 1.140500 |
| C | 2.403500 | -2.908700 | -1.224700 |
| H | 2.456800 | -2.365700 | -2.173300 |
| H | 3.351100 | -3.434300 | -1.058800 |
| H | 1.588400 | -3.639500 | -1.281000 |
| P | 2.085900 | -1.771500 | 0.159800 |
| C | 2.246000 | -2.844700 | 1.628300 |
| H | 3.228700 | -3.327500 | 1.571500 |
| H | 2.205500 | -2.250300 | 2.546300 |
| H | 1.483100 | -3.628200 | 1.661400 |
| C | -2.253800 | -3.052500 | -0.540200 |
| C | -0.965400 | -1.923800 | 1.320700 |
| C | -1.885900 | -0.799600 | 1.304300 |
| C | -3.002700 | -0.829800 | 0.461000 |
| C | -3.286100 | -1.923900 | -0.506900 |
| H | -0.307300 | -2.095900 | 2.167600 |
| H | -1.856900 | -0.094700 | 2.130200 |
| H | -4.281700 | -2.307400 | -0.248600 |
| H | -1.971000 | -3.296800 | -1.571400 |
| H | -3.408700 | -1.469400 | -1.499800 |
| H | -2.689700 | -3.985500 | -0.149200 |
| C | -1.050400 | -2.857100 | 0.299100 |
| H | -0.301000 | -3.647300 | 0.242400 |
| O | -3.905200 | 0.105700 | 0.442100 |
| C | -3.881100 | 1.180300 | 1.408000 |
| H | -4.710500 | 1.831000 | 1.133400 |
| H | -4.035200 | 0.770500 | 2.410500 |
| H | -2.936900 | 1.732200 | 1.355500 |

Crystallographic Data

Each single crystal was coated with Paratone oil and mounted on a MiTeGen MicroLoop. The X-ray intensity data for **5D, 14D, 17D, 56D, 5P, 14P, 15P, 30P, 31P, 84D, 85D,** and **88D** were

measured on a Bruker Kappa APEXII Duo system equipped with both a fine-focus sealed tube (Mo $K_\alpha$, $\lambda$ = 0.71073 Å) and a graphite monochromator, and an Incoatec Microfocus IµS (Cu $K_\alpha$, $\lambda$ = 1.54178 Å) and a multi-layer mirror monochromator. The X-ray intensity data for **9D, 10D, 11D, 18D, 35D, 37D, 39D, 41D, 43D, 44D, 45D, 54D, 55D, 59D, 28P, 65, 80, 81, 86D,** and **87D** were measured on a Bruker D8 Venture Kappa Photon III four-circle diffractometer system equipped with both an Incoatec IµS 3.0 micro-focus sealed X-ray tube (Mo Kα, $\lambda$ = 0.71073 Å) and a HELIOS double bounce multilayer mirror monochromator, and an Incoatec IµS 3.0 micro-focus sealed X-ray tube (Cu Kα, $\lambda$ = 1.54178 Å) and a HELIOS EF double bounce multilayer mirror monochromator.

The frames were integrated with the Bruker SAINT software package using a narrow-frame algorithm. Data were corrected for absorption effects using the Multi-Scan method SADABS[64] or TWINABS[65] Each structure was solved and refined using the Bruker SHELXTL Software Package[66] within APEX3/4 and OLEX2.[67] Non-hydrogen atoms were refined anisotropically. Most hydrogen atoms bound to a heteroatom (B-H, N-H, O-H) were located in the electron density map and refined isotropically, as were the H10 and H11 hydrogens in most of the structures. All other hydrogen atoms were placed in geometrically calculated positions with $U_{iso} = 1.2U_{equiv}$ of the parent atom ($U_{iso} = 1.5U_{equiv}$ for methyl).

For **5D, 15P, 35D, 43D, 55D**, **65**, and **80** the relative occupancy of the disordered atoms was freely refined. Constraints and/or restraints were used on either the anisotropic displacement parameters of the disordered atoms, or on the disordered bonds. For **14P, 41D, 54D**, **59D**, **81**, and **86D** the relative occupancy of the disordered atoms was freely refined without constraints or restraints. No disorder was modeled in **18D**, but restraints were used on the anisotropic displacement parameters of the atoms in the triflate anion.

For **10D**, the relative occupancy of the disordered atoms was freely refined, with constraints on the anisotropic displacement parameters of the disordered atoms and restraints on the disordered bonds. The occupancy of the acetone solvent as set at 50% to be consistent with the disorder of the neighboring groups. For **11D**, the site with a mixture of solvent and anion was modeled at 50% occupancy for each part. The relative occupancy of the disorder at site that was just anion was freely refined with the total occupancy reduced to account for the site symmetry. Constraints were used on the anisotropic displacement parameters of the disordered F and O atoms. For **14D**, the disordered triflate anions and acetonitrile solvent molecules were refined at half-occupancy, with constraints on the anisotropic displacement parameters of selected atoms and restraints on selected bonds.

For **5P**, the relative occupancy of the disordered atoms was freely refined, with restraints on the anisotropic displacement parameters of the disordered atoms. A partially occupied, severely disordered acetone molecule could not be adequately modeled with or without restraints was located in the crystal lattice. The solvent mask routine of OLEX2[65] was used to account for this disordered solvent. It found 22 electrons in a volume of 84 Å$^3$ in 1 void per unit cell. This is consistent with the presence of 0.33 acetone molecules per formula unit. Severely disordered solvent that could not be adequately modeled with or without restraints was also located in the crystal lattices of **17D, 37D** and **56D**. Thus, the structure factors were modified using the PLATON

SQUEEZE[68] technique, in order to produce a "solvate-free" structure factor set. PLATON reported a total electron density of 1204 $e^-$ and total solvent accessible volume of 3523 Å$^3$ for **17D**, corresponding to approximately 1.5 molecules of $CH_2Cl_2$ in the asymmetric unit. For **37D**, PLATON reported a total electron density of 172 $e^-$ and total solvent accessible volume of 602 Å$^3$ corresponding to one molecule of $CH_2Cl_2$ per ASU. For **56D,** PLATON reported a total electron density of 24 $e^-$ and total solvent accessible volume of 360 Å$^3$, corresponding approximately 0.5 exta molecules of $CH_2Cl_2$ in the ASU. In addition, the overall weak diffraction of the crystal of **56D** required a global RIGU restraint.

For **28P** and **39D**, the "domains" feature of APEX4 was used to identify the twin domains of each crystal. The BASF for the twin domain of **28P** refined to 0.45679. For **39D**, BASF parameters for the twin domains refined to 0.38770, 0.14970 and 0.14970, and global RIGU restraint was used to account for the overall poor crystal quality.

| | **5P** | **14P** | **15P** | **28P** | **30P** | **31P** |
|---|---|---|---|---|---|---|
| CCDC number | 2280153 | 2280154 | 2280155 | 2280156 | 2280157 | 2280158 |
| Formula | $C_{32}H_{44}BF_3N_7O_{6.33}PSW$ * | $C_{33}H_{44}BF_6N_{10}O_8PS_2W$ | $C_{26}H_{36}BF_6N_8O_9PS_2W$ | $C_{25}H_{32}BF_3N_7O_5PSW$ | $C_{30}H_{40}BF_3N_7O_5PSW$ | $C_{34}H_{47}BF_6N_9O_8PS_2W$ |
| FW (g/mol) | 942.57 * | 1112.53 | 1008.38 | 825.26 | 893.38 | 1113.55 |
| Temp (K) | 100 | 100 | 100 | 100(2) | 100 | 100 |
| λ (Å) | 0.71073 | 0.71073 | 0.71073 | 1.54178 | 0.71073 | 0.71073 |
| Size (mm) | 0.070 x 0.081 x 0.102 | 0.164 x 0.182 x 0.426 | 0.168 x 0.292 x 0.309 | 0.042 x 0.072 x 0.111 | 0.203 x 0.240 x 0.381 | 0.190 x 0.201 x 0.333 |
| Crystal habit | orange rod | orange block | orange block | yellow block | yellow block | orange block |
| Crystal system | triclinic | monoclinic | monoclinic | triclinic | monoclinic | triclinic |
| Space group | P -1 | P $2_1$/c | P $2_1$/c | P -1 | P $2_1$/c | P -1 |
| a (Å) | 9.9454(6) | 15.4248(14) | 9.7047(11) | 13.3047(7) | 13.7113(8) | 10.9676(9) |
| b(Å) | 10.6249(7) | 13.7297(13) | 11.8127(13) | 14.9097(8) | 18.7184(11) | 13.1199(10) |
| c (Å) | 18.6431(12) | 21.1271(19) | 33.303(4) | 16.6425(10) | 14.1770(9) | 15.8804(12) |
| α (°) | 95.863(2) | 90 | 90 | 87.057(4) | 90 | 97.080(2) |
| β (°) | 102.067(2) | 95.188(2) | 93.044(3) | 86.120(4) | 107.890(2) | 92.958(2) |
| γ (°) | 94.393(2) | 90 | 90 | 70.801(4) | 90 | 93.390(2) |
| Volume (Å$^3$) | 1906.6(2) | 4455.9(7) | 3812.4(7) | 3109.1(3) | 3462.6(4) | 2259.7(3) |
| Z | 2 | 4 | 4 | 4 | 4 | 2 |
| Density (g/cm$^3$) | 1.642 * | 1.658 | 1.757 | 1.763 | 1.714 | 1.637 |
| μ (mm$^{-1}$) | 3.191 | 2.805 | 3.269 | 8.579 | 3.509 | 2.766 |
| F(000) | 945 | 2224 | 2000 | 1632 | 1784 | 1116 |
| θ range (°) | 1.94 to 28.34 | 1.33 to 35.00 | 1.83 to 28.32 | 2.66 to 68.31 | 1.56 to 31.55 | 1.29 to 27.58 |
| Index ranges | $-13 \leq h \leq 13$ $-14 \leq k \leq 14$ $-24 \leq l \leq 24$ | $-24 \leq h \leq 24$ $-22 \leq k \leq 22$ $-34 \leq l \leq 34$ | $-12 \leq h \leq 11$ $-15 \leq k \leq 15$ $-44 \leq l \leq 44$ | $-15 \leq h \leq 15$ $-17 \leq k \leq 17$ $0 \leq l \leq 19$ | $-19 \leq h \leq 20$ $-27 \leq k \leq 27$ $-20 \leq l \leq 20$ | $-14 \leq h \leq 14$ $-17 \leq k \leq 17$ $-20 \leq l \leq 20$ |
| Reflns collected | 89284 | 188252 | 65332 | 34049 | 107805 | 112741 |

| Independent reflns | 9483 [$R_{int}$ = 0.0769] | 19588 [$R_{int}$ = 0.0305] | 9485 [$R_{int}$ = 0.0375] | 10353 [$R_{int}$ = 0.1077] | 11565 [$R_{int}$ = 0.0326] | 10435 [$R_{int}$ = 0.0321] |
|---|---|---|---|---|---|---|
| Data / restraints /parameters | 9483 / 518 / 630 | 19588 / 0 / 699 | 9485 / 21 / 528 | 10353 / 0 / 802 | 11565 / 0 / 462 | 10435 / 0 / 584 |
| GOF on $F^2$ | 1.050 | 1.080 | 1.174 | 1.040 | 1.036 | 1.081 |
| $R_1$ (I>2σ(I)) | 0.0297 | 0.0270 | 0.0427 | 0.0936 | 0.0161 | 0.0250 |
| $wR_2$ (all data) | 0.0587 | 0.0673 | 0.0907 | 0.2854 | 0.0366 | 0.0663 |

**Table S2.** Crystallographic data for 5P, 14P, 15P, 28P, 30P, and 31P.

* A solvent mask was used on this crystal, but the solvent was not included in the chemical formula, molecular weight, or density calculation.

| | **5D** | **9D** | **10D** | **11D** | **14D** | **17D** |
|---|---|---|---|---|---|---|
| CCDC number | 2254578 | 2280138 | 2280139 | 2280140 | 2280141 | 2280142 |
| Formula | $C_{35}H_{50}BF_3N_7O_7PSW$ | $C_{27}H_{36}BF_3N_7O_6PSW$ | $C_{63}H_{78}B_2F_6N_{14}O_{15}P_2S_2W_2$ | $C_{55}H_{70}B_2Cl_2F_6N_{14}O_{16}P_2S_2W_2$ | $C_{68}H_{95}B_2F_{12}N_{19}O_{17}P_2S_4W_2$ | $C_{26}H_{34}BF_3N_7O_7PSW$ * |
| FW (g/mol) | 995.51 | 869.32 | 1900.77 | 1883.53 | 2258.12 | 871.29 * |
| Temp (K) | 100(2) | 100(2) | 100(2) | 100(2) | 100(2) | 100(2) |
| λ (Å) | 0.71073 | 0.71073 | 0.71073 | 0.71073 | 1.54178 | 1.54178 |
| Size (mm) | 0.106 x 0.119 x 0.124 | 0.026 x 0.100 x 0.152 | 0.031 x 0.122 x 0.188 | 0.066 x 0.177 x 0.259 | 0.065 x 0.100 x 0.144 | 0.039 x 0.058 x 0.071 |
| Crystal habit | yellow block | yellow needle | yellow plate | yellow block | yellow block | yellow block |
| Crystal system | monoclinic | monoclinic | orthorhombic | monoclinic | monoclinic | trigonal |
| Space group | P $2_1$/n | P $2_1$/c | A ba2 | C 2/c | P $2_1$/n | R -3 |
| a (Å) | 10.5853(8) | 18.1635(7) | 27.7967(10) | 26.2328(9) | 12.8404(6) | 23.0734(3) |
| b(Å) | 13.7626(12) | 12.1003(4) | 16.4233(6) | 12.5641(4) | 30.1753(11) | 23.0734(3) |
| c (Å) | 26.901(2) | 16.4320(6) | 16.3512(6) | 22.4818(6) | 24.1161(10) | 39.1030(9) |
| α (°) | 90 | 90 | 90 | 90 | 90 | α = 90 |
| β (°) | 95.804(2) | 112.0710(10) | 90 | 112.3130(10) | 104.455(3) | 90 |
| γ (°) | 90 | 90 | 90 | 90 | 90 | 120 |
| Volume ($Å^3$) | 3898.9(5) | 3346.8(2) | 7464.5(5) | 6855.0(4) | 9048.3(7) | 18028.7(6) |
| Z | 4 | 4 | 4 | 4 | 4 | 18 |
| Density ($g/cm^3$) | 1.696 | 1.725 | 1.691 | 1.825 | 1.658 | 1.445 * |
| μ ($mm^{-1}$) | 3.129 | 3.629 | 3.266 | 3.631 | 6.672 | 6.728 |
| F(000) | 2008 | 1728 | 3792 | 3736 | 4528 | 7776 |
| θ range (°) | 1.52 to 28.34 | 2.07 to 26.37 | 1.90 to 28.29 | 1.92 to 30.54 | 2.39 to 68.38 | 2.48 to 68.40 |
| Index ranges | $-13 \le h \le 14$<br>$-18 \le k \le 18$<br>$-35 \le l \le 35$ | $-22 \le h \le 18$<br>$-15 \le k \le 15$<br>$-20 \le l \le 20$ | $-36 \le h \le 3$<br>$-21 \le k \le 1$<br>$-21 \le l \le 21$ | $-37 \le h \le 37$<br>$-17 \le k \le 17$<br>$-32 \le l \le 22$ | $-15 \le h \le 15$<br>$-36 \le k \le 33$<br>$-29 \le l \le 29$ | $-27 \le h \le 26$<br>$-27 \le k \le 27$<br>$-34 \le l \le 46$ |

| | | | | | | |
|---|---|---|---|---|---|---|
| Reflns collected | 46211 | 43550 | 44270 | 59861 | 114581 | 48306 |
| Independent reflns | 9721 [$R_{int}$ = 0.0795] | 6851 [$R_{int}$ = 0.0803] | 9223 [$R_{int}$ = 0.0546] | 10457 [$R_{int}$ = 0.0330] | 16614 [$R_{int}$ = 0.1154] | 7360 [$R_{int}$ = 0.0851] |
| Data / restraints /parameters | 9721 / 0 / 575 | 6851 / 0 / 436 | 9223 / 33 / 500 | 10457 / 0 / 574 | 16614 / 3 / 1244 | 7360 / 2 / 471 |
| GOF on $F^2$ | 1.029 | 1.052 | 1.060 | 1.043 | 1.049 | 1.050 |
| $R_1$ ($I>2\sigma(I)$) | 0.0397 | 0.0419 | 0.0497 | 0.0231 | 0.0550 | 0.0523 |
| $wR_2$ (all data) | 0.0843 | 0.1024 | 0.1064 | 0.0546 | 0.1496 | 0.1408 |

**Table S3.** Crystallographic Data for 5D, 9D, 10D, 11D, 14D, and 17D.

* A solvent mask was used on this crystal, but the solvent was not included in the chemical formula or density calculation.

| | **18D** | **35D** | **37D** | **39D** | **41D** | **43D** |
|---|---|---|---|---|---|---|
| CCDC number | 2280143 | 2254580 | 2254581 | 2280144 | 2280145 | 2280146 |
| Formula | $C_{28}H_{38}BF_3N_7O_6P$ SW | $C_{30}H_{40}BF_3N_7O_2$ PW | $C_{34}H_{49}BN_7O_4$ PW * | $C_{31}H_{35}BN_9O$ PW | $C_{35}H_{50}BCl_2N_8O_3$ PW | $C_{38}H_{46}BCl_2N_8O_4$ PW |
| FW (g/mol) | 883.34 | 813.32 | 845.43 * | 775.31 | 927.36 | 975.36 |
| Temp (K) | 100(2) | 100(2) | 100(2) | 100(2) | 100(2) | 100(2) |
| λ (Å) | 1.54178 | 0.71073 | 0.71073 | 1.54178 | 0.71073 | 0.71073 |
| Size (mm) | 0.043 x 0.056 x 0.088 | 0.045 x 0.060 x 0.132 | 0.103 x 0.144 x 0.221 | 0.024 x 0.027 x 0.046 | 0.059 x 0.107 x 0.133 | 0.033 x 0.049 x 0.073 |
| Crystal habit | yellow plate | yellow needle | colorless block | yellow-green plate | colorless plate | yellow plate |
| Crystal system | monoclinic | orthorhombic | monoclinic | monoclinic | monoclinic | triclinic |
| Space group | P $2_1$/c | P $2_12_12_1$ | P $2_1$/c | P c | P $2_1$/c | P -1 |
| a (Å) | 18.6500(11) | 8.7222(4) | 15.9401(5) | 18.9391(7) | 18.0447(7) | 9.3471(4) |
| b(Å) | 11.8837(8) | 15.2574(6) | 13.3600(4) | 8.3285(2) | 13.0380(5) | 11.5702(5) |
| c (Å) | 15.7013(10) | 24.0871(8) | 19.6480(6) | 20.9098(6) | 18.4661(8) | 19.0169(7) |
| α (°) | α = 90 | 90 | 90 | 90 | 90 | 80.3750(10) |
| β (°) | 100.539(4) | 90 | 104.7470(10) | 106.213(2) | 114.4240(10) | 80.8130(10) |
| γ (°) | 90 | 90 | 90 | 90 | 90 | 87.3350(10) |
| Volume ($Å^3$) | 3421.2(4) | 3205.5(2) | 4046.4(2) | 3167.02(17) | 3955.7(3) | 2001.26(14) |
| Z | 4 | 4 | 4 | 4 | 4 | 2 |
| Density ($g/cm^3$) | 1.715 | 1.685 | 1.388 * | 1.626 | 1.557 | 1.619 |
| μ ($mm^{-1}$) | 7.864 | 3.712 | 2.936 | 7.564 | 3.141 | 3.111 |
| F(000) | 1760 | 1624 | 1712 | 1544 | 1872 | 980 |
| θ range (°) | 4.43 to 68.72 | 2.15 to 28.29 | 2.02 to 29.58 | 2.43 to 66.66 | 2.05 to 30.05 | 1.94 to 26.41 |

| | | | | | | |
|---|---|---|---|---|---|---|
| Index ranges | $-22 \le h \le 21$<br>$-14 \le k \le 13$<br>$-18 \le l \le 16$ | $-11 \le h \le 9$<br>$-20 \le k \le 20$<br>$-27 \le l \le 32$ | $-22 \le h \le 22$<br>$-17 \le k \le 18$<br>$-26 \le l \le 27$ | $-22 \le h \le 21$<br>$0 \le k \le 9$<br>$0 \le l \le 24$ | $-25 \le h \le 25$<br>$-15 \le k \le 18$<br>$-25 \le l \le 25$ | $-11 \le h \le 11$<br>$-14 \le k \le 14$<br>$-23 \le l \le 20$ |
| Reflns collected | 19262 | 40453 | 79390 | 107755 | 74466 | 46834 |
| Independent reflns | 6204 [$R_{int}$ = 0.0855] | 7954 [$R_{int}$ = 0.0939] | 11346 [$R_{int}$ = 0.0480] | 5618 [$R_{int}$ = 0.1368] | 11551 [$R_{int}$ = 0.0416] | 8200 [$R_{int}$ = 0.0557] |
| Data / restraints /parameters | 6204 / 42 / 443 | 7954 / 2 / 420 | 11346 / 168 / 495 | 5618 / 798 / 789 | 11551 / 0 / 486 | 8200 / 1 / 540 |
| GOF on $F^2$ | 1.108 | 1.057 | 1.013 | 1.041 | 1.036 | 1.083 |
| $R_1$ ($I>2\sigma(I)$) | 0.0780 | 0.0420 | 0.0240 | 0.0699 | 0.0236 | 0.0385 |
| $wR_2$ (all data) | 0.2422 | 0.0639 | 0.0487 | 0.1924 | 0.0510 | 0.0971 |

**Table S4.** Crystallographic data for 18D, 35D, 37D, 39D, 41D, and 43D.

* A solvent mask was used on this crystal, but the solvent was not included in the chemical formula or density calculation.

| | **44D** | **45D** | **54D** | **55D** | **56D** | **59D** | **65** |
|---|---|---|---|---|---|---|---|
| CCDC number | 2280147 | 2280148 | 2280149 | 2280150 | 2280151 | 2280152 | 2280164 |
| Formula | $C_{41}H_{48}BN_8O_2PW$ | $C_{32}H_{44}BCl_2N_{10}O_2PW$ | $C_{27}H_{37}BN_7O_3PSW$ | $C_{37}H_{46}BF_3N_7O_5PSW$ | $C_{35}H_{49}BCl_2F_3N_8O_6PSW$ * | $C_{37}H_{49}BCl_2N_7O_2PW$ | $C_{25}H_{25}NO_3$ |
| FW (g/mol) | 910.50 | 897.30 | 765.32 | 983.50 | 1063.41 * | 920.36 | 387.46 |
| Temp (K) | 100(2) | 100(2) | 100(2) | 100(2) | 100(2) | 100(2) | 100(2) |
| λ (Å) | 1.54178 | 1.54178 | 0.71073 | 0.71073 | 1.54178 | 0.71073 | 1.54178 |
| Size (mm) | 0.044 x 0.069 x 0.077 | 0.017 x 0.035 x 0.055 | 0.092 x 0.114 x 0.155 | 0.066 x 0.267 x 0.329 | 0.038 x 0.060 x 0.110 | 0.074 x 0.158 x 0.232 | 0.043 x 0.046 x 0.103 |
| Crystal habit | colorless plate | colorless plate | colorless block | yellow plate | yellow plate | colorless block | colorless needle |
| Crystal system | monoclinic | monoclinic | triclinic | monoclinic | monoclinic | monoclinic | monoclinic |
| Space group | P $2_1$/n | P $2_1$/n | P -1 | P $2_1$/n | P $2_1$/n | P $2_1$/n | P $2_1$/n |
| a (Å) | 15.8872(3) | 10.3256(2) | 8.8869(5) | 10.7219(3) | 10.2467(8) | 10.4721(5) | 15.3236(3) |
| b(Å) | 14.1044(3) | 12.7169(3) | 11.8725(6) | 13.5723(5) | 11.9956(8) | 13.7216(5) | 11.9139(2) |
| c (Å) | 18.3258(4) | 27.5053(6) | 15.7808(8) | 26.4365(9) | 35.573(3) | 26.8281(10) | 22.3480(5) |
| α (°) | 90 | 90 | 70.979(2) | 90 | 90 | 90 | 90 |
| β (°) | 109.9300(10) | 93.258(2) | 75.008(2) | 94.6220(10) | 92.057(7) | 93.564(2) | 100.6730(10) |
| γ (°) | 90 | 90 | 87.460(2) | 90 | 90 | 90 | 90 |
| Volume ($Å^3$) | 3860.50(14) | 3605.87(14) | 1519.08(14) | 3834.6(2) [3] | 4369.6(6) | 3847.6(3) | 4009.35(14) |
| Z | 4 | 4 | 2 | 4 | 4 | 4 | 8 |

| | | | | | | | |
|---|---|---|---|---|---|---|---|
| Density (g/cm$^3$) | 1.567 | 1.653 | 1.673 | 1.704 | 1.616 * | 1.589 | 1.284 |
| μ (mm$^{-1}$) | 6.313 | 8.092 | 3.965 | 3.177 | 7.374 | 3.226 | 0.668 |
| F(000) | 1840 | 1800 | 764 | 1976 | 2136 | 1856 | 1648 |
| θ range (°) | 3.19 to 68.37 | 3.22 to 68.37 | 1.90 to 30.52 | 2.00 to 28.32 | 2.49 to 66.82 | 2.05 to 30.53 | 3.24 to 68.30 |
| Index ranges | -19 ≤ h ≤ 18<br>-16 ≤ k ≤ 16<br>-22 ≤ l ≤ 21 | -12 ≤ h ≤ 12<br>-15 ≤ k ≤ 13<br>-33 ≤ l ≤ 32 | -12 ≤ h ≤ 12<br>-16 ≤ k ≤ 16<br>-22 ≤ l ≤ 20 | -13 ≤ h ≤ 14<br>-18 ≤ k ≤ 18<br>-35 ≤ l ≤ 35 | -9 ≤ h ≤ 12<br>-14 ≤ k ≤ 11<br>-42 ≤ l ≤ 42 | -14 ≤ h ≤ 14<br>-18 ≤ k ≤ 19<br>-38 ≤ l ≤ 32 | -18 ≤ h ≤ 18<br>-14 ≤ k ≤ 1<br>-26 ≤ l ≤ 26 |
| Reflns collected | 40264 | 34749 | 47432 | 74417 | 31798 | 80300 | 52716 |
| Independent reflns | 7055 [$R_{int}$ = 0.0590] | 6602 [$R_{int}$ = 0.1534] | 9238 [$R_{int}$ = 0.0376] | 9516 [$R_{int}$ = 0.0481] | 7700 [$R_{int}$ = 0.1319] | 11716 [$R_{int}$ = 0.0499] | 7342 [$R_{int}$ = 0.0446] |
| Data / restraints /parameters | 7055 / 0 / 503 | 6602 / 0 / 446 | 9238 / 0 / 404 | 9516 / 6 / 595 | 7700 / 488 / 497 | 11716 / 0 / 504 | 7342 / 2 / 539 |
| GOF on $F^2$ | 1.012 | 1.022 | 1.026 | 1.091 | 1.130 | 1.065 | 1.011 |
| $R_1$ (I>2σ(I)) | 0.0250 | 0.0647 | 0.0170 | 0.0285 | 0.1073 | 0.0254 | 0.0379 |
| $wR_2$ (all data) | 0.0611 | 0.1796 | 0.0400 | 0.0722 | 0.2663 | 0.0613 | 0.1006 |

**Table S5.** Crystallographic data for 44D, 45D, 54D-56D, 59D, and 65.

* A solvent mask was used on this crystal, but the solvent was not included in the chemical formula or density calculation.

| | **80** | **81** | **84D** | **85D** | **86D** | **87D** | **88D** |
|---|---|---|---|---|---|---|---|
| CCDC number | 2283551 | 2314278 | 2280161 | 2280162 | 2280159 | 2280160 | 2280163 |
| Formula | $C_{42}H_{38}Cl_2N_4$ | $C_{19}H_{16}N_2$ | $C_{27}H_{39}BF_3N_8O_6$ PSW | $C_{36}H_{44}BF_3N_9O_5$ PSW | $C_{30}H_{41}BCl_2N_7O_2$PW | $C_{25}H_{33}BN_7O_2$PW | $C_{28}H_{35}BN_9O_2$PW |
| FW (g/mol) | 668.81 | 272.34 | 886.35 | 997.49 | 828.23 | 689.21 | 755.28 |
| Temp (K) | 100(2) | 100(2) | 100(2) | 100(2) | 100(2) | 100(2) | 100(2) |
| λ (Å) | 1.54178 | 0.71073 | 0.71073 | 0.71073 | 0.71073 | 0.71073 | 0.71073 |
| Size (mm) | 0.035 x 0.054 x 0.090 | 0.090 x 0.145 x 0.742 | 0.031 x 0.212 x 0.213 | 0.109 x 0.228 x 0.282 | 0.068 x 0.140 x 0.287 | 0.080 x 0.102 x 0.111 | 0.038 x 0.101 x 0.162 |
| Crystal habit | green-blue plate | blue needle | colorless plate | orange plate | colorless plate | colorless plate | colorless Plates |
| Crystal system | monoclinic | monoclinic | monoclinic | monoclinic | monoclinic | monoclinic | monoclinic |
| Space group | P $2_1$/n | P $2_1$/n | P $2_1$/c | P $2_1$/n | P $2_1$/n | P $2_1$/n | P $2_1$/n |

| | | | | | | | |
|---|---|---|---|---|---|---|---|
| a (Å) | 10.4781(4) | 7.6353(3) | 7.9137(6) | 16.5998(13) | 10.1692(3) | 10.2013(3) | 11.6812(8) |
| b(Å) | 17.6935(7) | 18.2561(10) | 20.0549(15) | 14.7979(12) | 14.2343(5) | 18.9174(6) | 12.6730(10) |
| c (Å) | 19.2979(9) | 10.5852(5) | 22.0340(16) | 17.2268(15) | 23.2178(7) | 14.7994(5) | 20.9074(18) |
| α (°) | 90 | 90 | 90 | 90 | 90 | 90 | 90 |
| β (°) | 104.626(3) | 104.932( | 93.262(2) | 102.409(2) | 93.9130(10) | 106.7650(10) | 92.612(2) |
| γ (°) | 90 | 90 | 90 | 90 | 90 | 90 | 90 |
| Volume (Å$^3$) | 3461.8(3) | 1425.66(12) | 3491.3(5) | 4132.8(6) | 3352.98(18) | 2734.63(15) | 3091.8(4) |
| Z | 4 | 4 | 4 | 4 | 4 | 4 | 4 |
| Density (g/cm$^3$) | 1.283 | 1.269 | 1.686 | 1.603 | 1.641 | 1.674 | 1.623 |
| μ (mm$^{-1}$) | 1.947 | 0.075 | 3.482 | 2.950 | 3.692 | 4.319 | 3.830 |
| F(000) | 1406 | 576 | 1768 | 2000 | 1656 | 1368 | 1504 |
| θ range (°) | 3.44 to 68.39 | 2.23 to 28.28 | 1.37 to 28.33 | 1.55 to 26.44 | 2.27 to 28.29 | 2.35 to 27.49 | 1.88 to 25.38 |
| Index ranges | -12 ≤ h ≤ 12<br>-21 ≤ k ≤ 21<br>-23 ≤ l ≤ 23 | -8 ≤ h ≤ 10<br>-24 ≤ k ≤ 22<br>-14 ≤ l ≤ 14 | -10 ≤ h ≤ 10<br>-26 ≤ k ≤ 26<br>-29 ≤ l ≤ 29 | -20 ≤ h ≤ 20<br>-18 ≤ k ≤ 16<br>-21 ≤ l ≤ 21 | -13 ≤ h ≤ 12<br>-18 ≤ k ≤ 18<br>-30 ≤ l ≤ 30 | -12 ≤ h ≤ 13<br>-24 ≤ k ≤ 24<br>-19 ≤ l ≤ 19 | -14 ≤ h ≤ 13<br>-15 ≤ k ≤ 15<br>-25 ≤ l ≤ 25 |
| Reflns collected | 40342 | 19007 | 71208 | 41904 | 57661 | 45585 | 35667 |
| Independent reflns | 6348 [$R_{int}$ = 0.0910] | 3535 [$R_{int}$ = 0.0538] | 8708 [$R_{int}$ = 0.0774] | 8514 [$R_{int}$ = 0.0686] | 8312 [$R_{int}$ = 0.0395] | 6260 [$R_{int}$ = 0.0495] | 5671 [$R_{int}$ = 0.1179] |
| Data / restraints /parameters | 6348 / 16 / 456 | 3535 / 0 / 245 | 8708 / 1 / 460 | 8514 / 0 / 527 | 8312 / 0 / 441 | 6260 / 0 / 346 | 5671 / 0 / 394 |
| GOF on $F^2$ | 1.096 | 1.033 | 1.022 | 1.043 | 1.161 | 1.050 | 0.998 |
| $R_1$ (I>2σ(I)) | 0.0661 | 0.0435 | 0.0337 | 0.0471 | 0.0315 | 0.0284 | 0.0387 |
| $wR_2$ (all data) | 0.1601 | 0.1079 | 0.0694 | 0.1313 | 0.0688 | 0.0693 | 0.0730 |

**Table S6.** Crystallographic data for 80-81 and 84D-88D.

## External Data Sets associated with manuscript:

## Data S1: CIF files for all crystal structures.

## Data S2: XYZ Files for DFT calculations.

## REFERENCES AND NOTES


1. J.-L. Reymond, M. Awale, Exploring chemical space for drug discovery using the chemical universe database. *ACS Chem. Nerosci.* **3**, 649–657 (2012).

2. D. C. Blakemore , L. Castro, I. Churcher, D. C. Rees, A. W. Thomas, D. M. Wilson, A. Wood, Organic synthesis provides opportunities to transform drug discovery. *Nat. Chem.* **10**, 383–394 (2018).

3. D. G. Brown, J. Boström, Analysis of past and present synthetic methodologies on medicinal chemistry: Where have all the new reactions gone? *J. Med. Chem.* **59**, 4443–4458 (2016).

4. F. Lovering, J. Bikker, C. Humblet, Escape from flatland: Increasing saturation as an approach to improving clinical success. *J. Med. Chem.* **52**, 6752–6756 (2009).

5. W. C. Wertjes, E. H. Southgate, D. Sarlah, Recent advances in chemical dearomatization of nonactivated arenes. *Chem. Soc. Rev.* **47**, 7996–8017 (2018).

6. A. R. Pape, K. P. Kaliappan, E. P. Kündig, Transition-metal-mediated dearomatization reactions. *Chem. Rev.* **100**, 2917–2940 (2000).

7. L. N. Mander, Exploitation of aryl synthons in the synthesis of polycyclic natural products. *Synlett*, **1991**, 134–144 (1991).

8. T. Bach, Additions to functionalized arenes with concurrent dearomatization. *Angew. Chem. Int. Ed. Engl.* **35**, 729–730 (1996).

9. J. T. Weatherford-Pratt, J. A. Smith, J. M. Bloch, M. N. Ericson, J. T. Myers, K. S. Westendorff, D. A. Dickie, W. D. Harman, The double protonation of dihapto-coordinated benzene complexes enables dearomatization using aromatic nucleophiles. *Nat. Commun.* **14**, 3145 (2023).

10. K. D. Welch, D. P. Harrison, E. C. Lis, W. Liu, R. J. Salomon, W. D. Harman, W. H. Myers, Large-scale syntheses of several synthons to the dearomatization agent {TpW(NO)(PMe3)} and convenient spectroscopic tools for product analysis. *Organometallics* **26**, 2791–2794 (2007).

11. J. M. Keane, W. D. Harman, A new generation of π-basic dearomatization agents. *Organometallics* **24**, 1786–1798 (2005).

12. D. P. Harrison, W. D. Harman, Opening new chemical space through novel dearomatization reactions. *Aldrichimica Acta* **45**, 45–55 (2012).

13. J. M. Keane, M. D. Chordia, C. J. Mocella, M. Sabat, C. O. Trindle, W. D. Harman, Transition metal-stabilized arenium cations: Protonation of arenes dihapto-coordinated to π-basic metal fragments. *J. Am. Chem. Soc.* **126**, 6806–6815 (2004).

14. E. C. Lis, R. J. Salomon, M. Sabat, W. H. Myers, W. D. Harman, Synthesis of 1-oxadecalins from anisole promoted by tungsten. *J. Am. Chem. Soc.* **130**, 12472–12476 (2008).

15. D. P. Harrison, A. C. Nichols-Nielander, V. E. Zottig, L. Strausberg, R. J. Salomon, C. O. Trindle, M. Sabat, T. B. Gunnoe, D. A. Iovan, W. H. Myers, W. D. Harman, Hyperdistorted tungsten allyl complexes and their stereoselective deprotonation to form dihapto-coordinated dienes. *Organometallics* **30**, 2587–2597 (2011).

16. K. B. Wilson, J. A. Smith, H. S. Nedzbala, E. K. Pert, S. J. Dakermanji, D. A. Dickie, W. D. Harman, Highly functionalized cyclohexenes derived from benzene: Sequential tandem addition reactions promoted by tungsten. *J. Org. Chem.* **84**, 6094–6116 (2019).

17. H. Mayr, B. Kempf, A. R. Ofial, Pi-nucleophilicity in carbon-carbon bond-forming reactions. *Acc. Chem. Res.* **36**, 66–77 (2003).

18. M. Kędziorek, P. Mayer, H. Mayr, Nucleophilic reactivities of azulene and fulvenes. *Eur. JOC* **2009**, 1202–1206 (2009).

19. S. H. Meiere, W. D. Harman, Binding selectivity of dihapto-coordinated olefins, ketones, and aldehydes utilizing the asymmetric π-basic metal fragment {TpRe(CO)(1-methylimidazole)} (Tp = hydridotris(pyrazolyl)borate). *Organometallics* **20**, 3876–3883 (2001).

20. T. C. Coombs, W. Huang, E. C. Garnier-Amblard, L. S. Liebeskind, Novel substitutions of 1-alkoxy- and 1-arylsulfonyloxy-η3-allylmolybdenum complexes. A case for η1-alkenyl carbene complexes as intermediates. *Organometallics* **29**, 5083–5097 (2010).

21. Y. D. Ward, L. A. Villanueva, G. D. Allred, L. S. Liebeskind, Preparation of dicarbonyl[hydrotris(1-pyrazolyl)borato](η3-allyl)molybdenum complexes bearing electron-donating substituents (1-((tert-Butyldimethylsilyl)oxy), 1-alkoxy, and 1-acetoxy) via the nucleophilic addition of Mo(CO)3(DMF)3to enals and enones. *Organometallics* **15**, 4201–4210 (1996).

22. J. M. Keane, F. Ding, M. Sabat, W. D. Harman, Solid-state induced control of kinetically unstable stereoisomers. *J. Am. Chem. Soc.* **126**, 785–789 (2004).

23. K. B. Wilson, "*Functionalization of Benzene and its Derivatives by Coordination to a Tungsten Dearomatization Agent"*, thesis, University of Virginia, Charlottesville, VA (2018).

24. P. J. Canfield, I. M. Blake, Z.-L. Cai, I. J. Luck, E. Krausz, R. Kobayashi, J. R. Reimers, M. J. Crossley, A new fundamental type of conformational isomerism. *Nat. Chem.***10**, 615–624 (2018).

25. R. A. Baillie, R. W. Y. Man, M. V. Shree, C. Chow, M. E. Thibault, W. S. McNeil, P. Legzdins, Intermolecular C–H activations of hydrocarbons initiated by Cp*M(NO)(CH2CMe3)(η3-CH2CHCHPh) complexes (M = Mo, W). *Organometallics* **30**, 6201–6217 (2011).

26. M. F. Semmelhack, in *Comprehensive Organic Synthesis*, B. M. Trost, I. Fleming, Eds. (Pergamon, 1992), vol. 4.

27. L. Wei, Intermolecular dearomative 1,2-amination/carbonylation via nucleophilic addition of simple amines to arene π-bonds. *Chem. A Eur. J.*, **29**, e202300776, (2023).

28. K. B. Wilson, J. T. Myers, H. S. Nedzbala, L. A. Combee, M. Sabat, W. D. Harman, Sequential tandem addition to a tungsten–trifluorotoluene complex: A versatile method for the preparation of highly functionalized trifluoromethylated cyclohexenes. *J. Am. Chem. Soc.* **139**, 11401–11412 (2017).

29. H. Pang, D. Wu, H. Cong, G. Yin, Stereoselective palladium-catalyzed 1,3-arylboration of unconjugated dienes for expedient synthesis of 1,3-disubstituted cyclohexanes. *ACS Catal.* **9**, 8555–8560 (2019).

30. S. Di Micco, R. Vitale, M. Pellecchia, M. F. Rega, R. Riva, A. Basso, G. Bifulco, Identification of lead compounds as antagonists of protein Bcl-xL with a diversity-oriented multidisciplinary approach. *J. Med. Chem.* **52**, 7856–7867 (2009).

31. M. E. Hoke, M.-R. Brescia, S. Bogaczyk, P. DeShong, B. W. King, M. T. Crimmins, Regioselectivity in the palladium-catalyzed addition of carbon nucleophiles to carbocyclic derivatives. *J. Org. Chem.* **67**, 327–335 (2002).

32. P. Ponchai, K. Adpakpang, S. Bureekaew, Selective cyclohexene oxidation to allylic compounds over a Cu-triazole framework via homolytic activation of hydrogen peroxide. *Dalton Trans.* **50**, 7917–7921 (2021).

33. X. Zhang, R. C. Larock, Synthesis of highly substituted 1,3-dienes, 1,3,5-trienes, and 3,6-disubstituted cyclohexenes by the palladium-catalyzed coupling of organic halides, internal alkynes or 1,3-cyclohexadienes, and organoboranes. *Tetrahedron* **66**, 4265–4277 (2010).

34. M. Takimoto, M. Mori, Cross-coupling reaction of oxo-π-allylnickel complex generated from 1,3-diene under an atmosphere of carbon dioxide. *J. Am. Chem. Soc.* **123**, 2895–2896 (2001).

35. R. C. Larock, Y. Wang, X. Dong, T. Yao, Synthesis of C-5 substituted nucleosides via palladium-catalyzed coupling of dienes and amines. *Tetrahedron* **61**, 11427–11439 (2005).

36. A. Basso, L. Banfi, G. Guanti, R. Riva, Straightforward stereoselective synthesis of polyfunctionalised cyclohexenols using a multicomponent approach. *Tetrahedron* **66**, 2390–2397 (2010).

37. M. S. Sell, R. D. Rieke, Carbocyclization of E,E-1,4-Diphenyl-1,3-butadiene with dichloroalkanes mediated by Rieke metals. *Synth. Commun.* **25**, 4107–4113 (1995).

38. A. Izumiseki, H. Yamamoto, Intermolecular/intramolecular sequential aldol reaction. *J. Am. Chem. Soc.* **136**, 1308–1311 (2014).

39. M. Yu, S. Lou, F. Gonzalez-Bobes, Ring-closing metathesis in pharmaceutical development: Fundamentals, applications, and future directions. *OPR&D* **22**, 918–946 (2018).

40. X. Jiang, R. Wang, Recent developments in catalytic asymmetric inverse-electron-demand Diels–Alder reaction. *Chem. Rev.* **113**, 5515–5546 (2013).

41. H. Xie, G. M. Sammis, E. M. Flamme, C. M. Kraml, E. J. Sorensen, The catalytic asymmetric Diels–Alder reactions and post-cycloaddition reductive transpositions of 1-hydrazinodienes. *Chem. A Eur. J.* **17**, 11131–11134 (2011).

42. R. Giovannini, P. Knochel, Ni(II)-catalyzed cross-coupling between polyfunctional arylzinc derivatives and primary alkyl iodides. *J. Am. Chem. Soc.* **120**, 11186–11187 (1998).

43. C. Cordovilla, C. Bartolomé, J. M. Martínez-Ilarduya, P. Espinet, The Stille reaction, 38 years later. *ACS Catal.* **5**, 3040–3053 (2015).

44. N. Miyaura, A. Suzuki, Palladium-catalyzed cross-coupling reactions of organoboron compounds. *Chem. Rev.* **95**, 2457–2483 (1995).

45. Y. Hatanaka, T. Hiyama, Cross-coupling of organosilanes with organic halides mediated by a palladium catalyst and tris(diethylamino)sulfonium difluorotrimethylsilicate. *J. Org. Chem.* **53**, 918–920 (1988).

46. E. J. Corey, G. H. Posner, Selective formation of carbon-carbon bonds between unlike groups using organocopper reagents. *J. Am. Chem. Soc.* **89**, 3911–3912 (1967).

47. H. O. House, W. L. Respess, G. M. Whitesides, The chemistry of carbanions. XII. The role of copper in the conjugate addition of organometallic reagents1. *J. Org. Chem.* **31**, 3128–3141 (1966).

48. G. Fouquet, M. Schlosser, Improved carbon-carbon linking by controlled copper catalysis. *Angew. Chem. Int. Ed. Engl.* **13**, 82–83 (1974).

49. M. Rueping, B. J. Nachtsheim, A review of new developments in the Friedel–Crafts alkylation – From green chemistry to asymmetric catalysis. *Beilstein J.Org. Chem.* **6**, 6 (2010).

50. T.-Y. Lee, Y. Kang, Y. Chung, R. Pike, D. Sweigart, Manganese-mediated synthesis of cis-disubstituted cyclohexadienes via double nucleophilic addition to coordinated arenes. *Inorganica Chim. Acta* **214**, 125–134 (1993).

51. F. Rose-Munch, E. Rose, Planar chiral (η5-cyclohexadienyl)- and (η6-arene)-tricarbonylmanganese complexes: Synthetic routes and application. *Org. Biomol. Chem.* **9**, 4725–4735 (2011).

52. H.-J. Knölker, Iron-mediated synthesis of heterocyclic ring systems and applications in alkaloid chemistry. *Synlett* **1992**, 371–387 (1992).

53. A. J. Pearson, Tricarbonyl(diene)iron complexes: Synthetically useful properties. *Acc. Chem. Res.* **13**, 463–469 (1980).

54. J.-L. Han, M.-C. Liu, C. W. Ong, Diastereoselective synthesis of a highly functionalized angularly substituted cis-perhydroisoquinoline-3,6-dione via organoiron. *J. Org. Chem.* **75**, 1637–1642 (2010).

55. A. J. Pearson, *Iron Compouinds in Organic Synthesis*, Chapter 6. (Academic Press, 1994).

56. L. A. P. Kane-Maguire, E. D. Honig, D. A. Sweigart, Nucleophilic addition to coordinated cyclic .pi.-hydrocarbons: Mechanistic and synthetic studies. *Chem. Rev.* **84**, 525 (1984), 525–543 (1984)

57. L. A. P. Kane-Maguire, C. A. Mansfield, Cationic metal-dienyl complexes as electrophilic reagents on aromatic molecules. *JCS, Chem. Commun.*, **540-541** (1973).

58. K. K. Gruner, T. Hopfmann, K. Matsumoto, A. Jäger, T. Katsuki, H.-J. Knölker, Efficient iron-mediated approach to pyrano[3,2-a]carbazole alkaloids—first total syntheses of O-methylmurrayamine A and 7-methoxymurrayacine, first asymmetric synthesis and assignment of the absolute configuration of (−)-trans-dihydroxygirinimbine. *Org. Biomol. Chem.* **9**, 2057–2061 (2011).

59. P. Dunås, A. J. Paterson, G. Kociok-Köhn, S. E. Lewis, N. Kann, Selective iron-mediated C- and O-addition of phenolic nucleophiles to a cyclohexadiene scaffold using renewable precursors. *ACS Sustain. Chem. Eng.* **7**, 7155–7162 (2019).

60. J. A. Pienkos, V. E. Zottig, D. A. Iovan, M. Li, D. P. Harrison, M. Sabat, R. J. Salomon, L. Strausberg, V. A. Teran, W. H. Myers, W. D. Harman, Friedel–Crafts ring-coupling reactions promoted by tungsten dearomatization agent. *Organometallics* **32**, 691–703 (2013).

61. B. L. MacLeod, J. A. Pienkos, K. B. Wilson, M. Sabat, W. H. Myers, W. D. Harman, Synthesis of novel hexahydroindoles from the dearomatization of indoline. *Organometallics* **35**, 370–387 (2016).

62. A. W. Lankenau, D. A. Iovan, J. A. Pienkos, R. J. Salomon, S. Wang, D. P. Harrison, W. H. Myers, W. D. Harman, Enantioenrichment of a tungsten dearomatization agent utilizing chiral acids, *J. Am. Chem. Soc.* **137**, 3649–3655 (2015).

63. J. A. Smith, A. Schouten, J. H. Wilde, K. S. Westendorff, D. A. Dickie, D. H. Ess, W. D. Harman, Experiments and direct dynamics simulations that probe η2-arene/aryl hydride equilibria of tungsten benzene complexes. *J. Am. Chem. Soc.* **142**, 16437–16454 (2020).

64. L. Krause, R. Herbst-Irmer, G. M. Sheldrick, D. Stalke, Comparison of silver and molybdenum microfocus X-ray sources for single-crystal structure determination. *J. Appl. Cryst.* **48**, 3–10 (2015).

65. M. Sevvana, M. Ruf, I. Usón, G. M. Sheldrick, R. Herbst-Irmer, Non-merohedral twinning: From minerals to proteins. *Acta Crystallogr D Struct Biol* **75**, 1040–1050 (2019).

66. G. M. Sheldrick, SHELXT - integrated space-group and crystal-structure determination. *Acta Crystallogr A Found Adv* **71**, 3–8 (2015).

67. O. Dolomanov, L. Bourhis, R. Gildea, J. Howard, H. Puschmann, OLEX2: A complete structure solution, refinement and analysis program. *J. Appl. Cryst. J. Appl. Cryst* **42**, 339–341 (2009).

68. A. L. Spek, PLATON SQUEEZE: A tool for the calculation of the disordered solvent contribution to the calculated structure factors. *Acta Crystallogr C Struct Chem* **71**, 9–18 (2015).
